# Supplementary material for: Engineering Fluoroacetate Dehalogenase by Growth‐Based Selections on Non‐Natural Organofluorides
Source: Angew Chem Int Ed Engl. 2026 Jan 28;65(10):e24234. doi: 10.1002/anie.202524234 (PMC12955510; doi:10.1002/anie.202524234)
Supplement: Supplementary file 1 — Supporting File 1: The manuscript is accompanied by a Supporting Information file, which includes Supporting Tables and Figures, a Supporting Discussion, experimental procedures, and sequences of H1‐variants used in the study. Additionally, all Boltz‐2 predictions are included (.json and. cif files), and a supporting document is provided, which contains all raw 19F‐NMR spectra for kinetic characterization of H1‐variants, determination of defluorination activity in the supernatant of cultures, and relaxation delay tests. The authors have cited additional references within the Supporting Information [51, 52, 53, 54, 55, 56, 57, 58, 59, 60, 61, 62, 63, 64]. [file ANIE-65-e24234-s003.pdf]

Supporting Information

Engineering fluoroacetate dehalogenase by growth-based selections on non-natural organofluorides

Suzanne C. Jansen[1], Pauline van Beers[1], and Clemens Mayer[1*]

Affiliations:

[1] Biomolecular Chemistry & Catalysis, Stratingh Institute, University of Groningen, Nijenborgh 3, 9747 AG Groningen, The Netherlands

*Correspondence to: c.mayer@rug.nl

Table of contents

1. Supporting Figures S2
2. Supporting Tables S10
3. Supporting Discussion S19
4. Experimental S21
5. Sequences S43
6. Supporting References S47

1. Supporting Figures

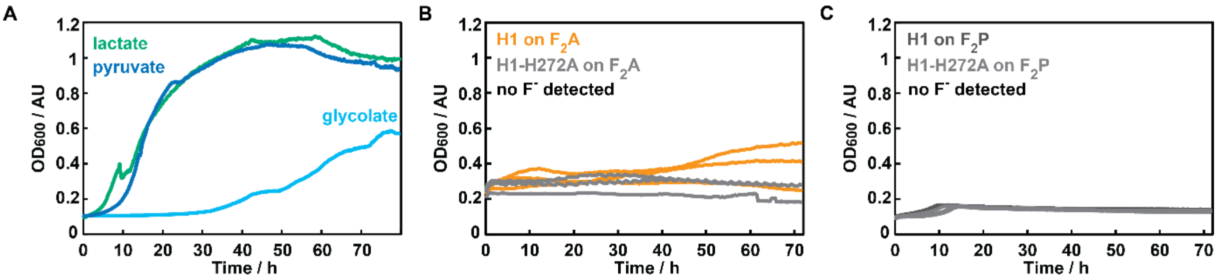

Figure S1: A: Positive control growth phenotypes of cells producing H1, when utilizing lactate (green), pyruvate (dark blue) or glycolate (light blue) as carbon sources. **B-C:** Growth phenotype of H1- or H1-H272A-producing cells failing to utilize F_2A (**B**) or F_2P (**C**) as sole carbon source. No F^- was detected in culture supernatant by ^{19}F-NMR.

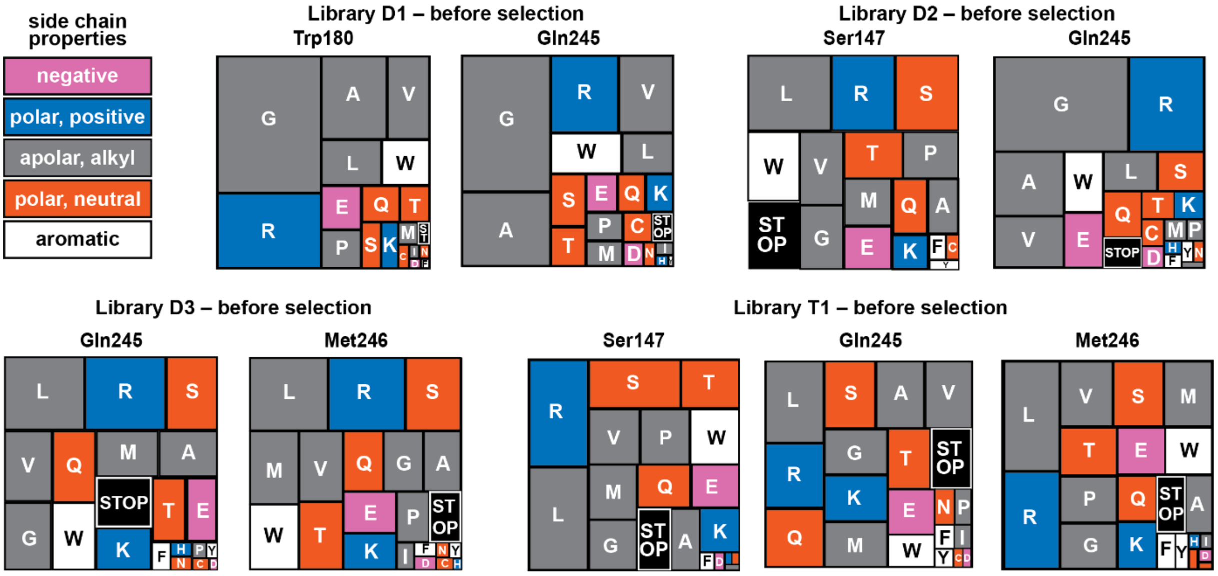

Figure S2: Treemap charts depicting the amino acid distribution across randomized positions for all populations prior to selection. Top left, a legend for the color-code of the properties of amino acid side chains. The data is based on raw reads from whole-plasmid sequencing samples (see *Experimental*).

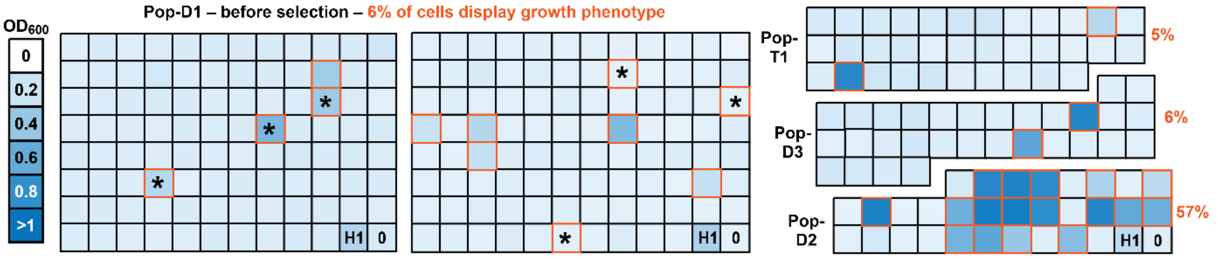

Figure S3: Distribution of H1-variants displaying a growth phenotype across populations when utilizing FP. Each block depicts the OD_{600} for a host producing a randomized H1-variant after 50 hours of growth on FP. Values represent single measurements. Blocks marked with orange lines were considered to have a positive growth phenotype. Blocks marked with asterisks showed a positive growth phenotype within 24 hours of growth, and were subjected to ^{19}F-NMR analysis.

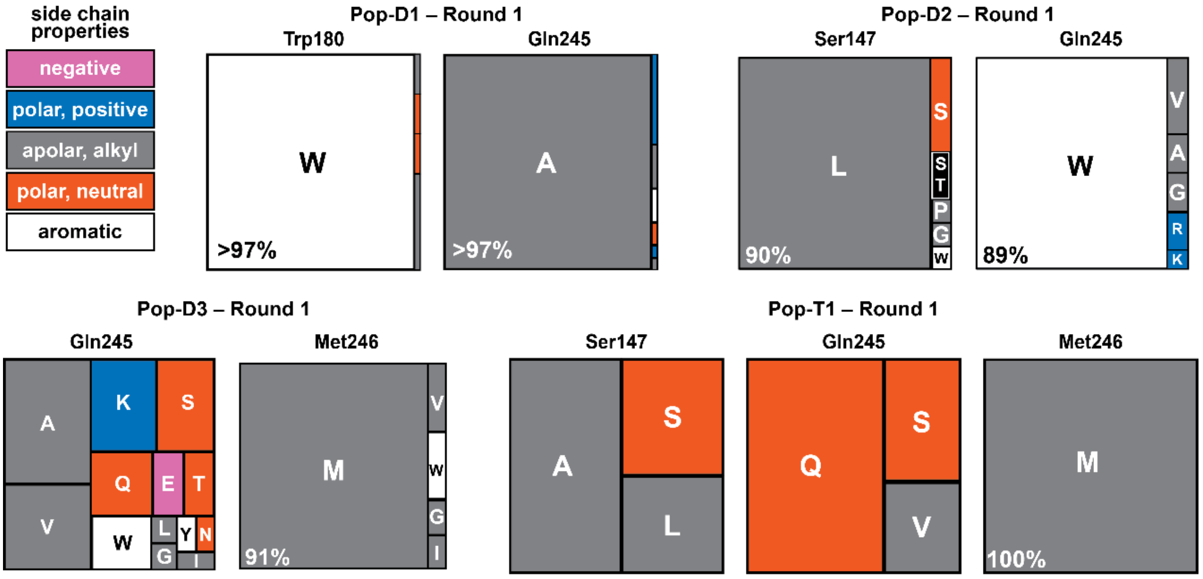

Figure S4: Treemap charts depicting the enrichment of amino acids across randomized positions in populations after one passage on FP as sole carbon source. Top left, a legend for the color-code of the properties of amino acid side chains. The data is based on raw reads from whole-plasmid sequencing samples (see *Experimental*).

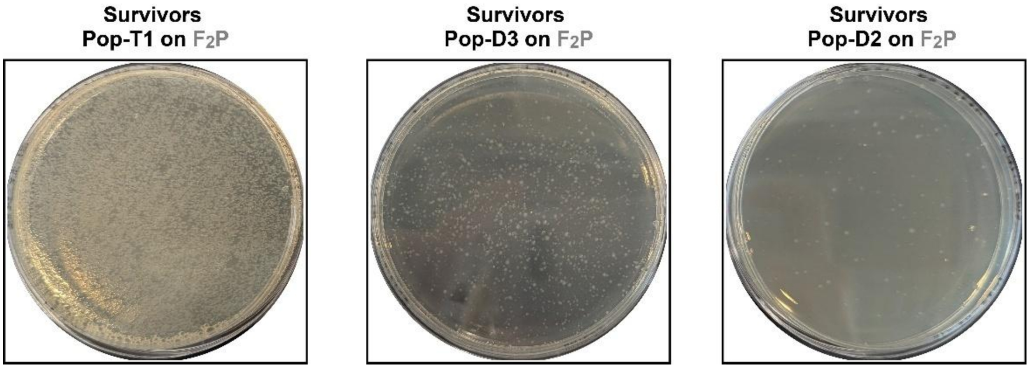

Figure S5: Comparison of surviving bacteria following a 14-day incubation with F_2P as the sole carbon source.

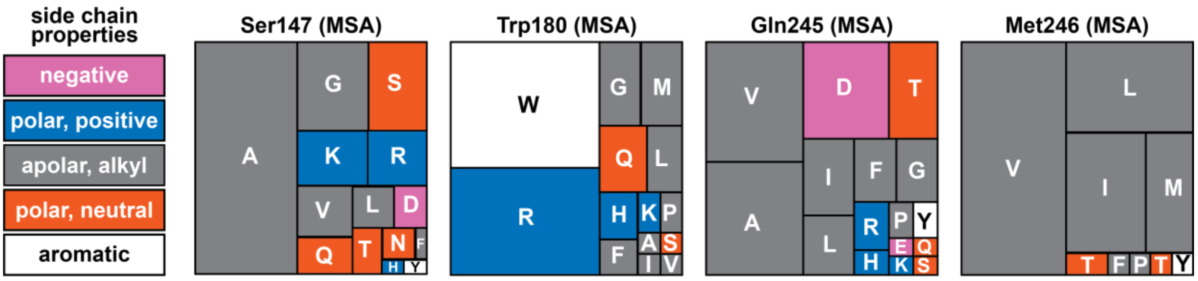

Figure S6: Treemap charts depicting the amino acid distribution from HotSpotWizard's multiple sequence alignment (MSA) across targeted positions (legend for properties of amino acids on the left). The distribution is based on 200 sequences with gaps in the alignment being left out for clarity.

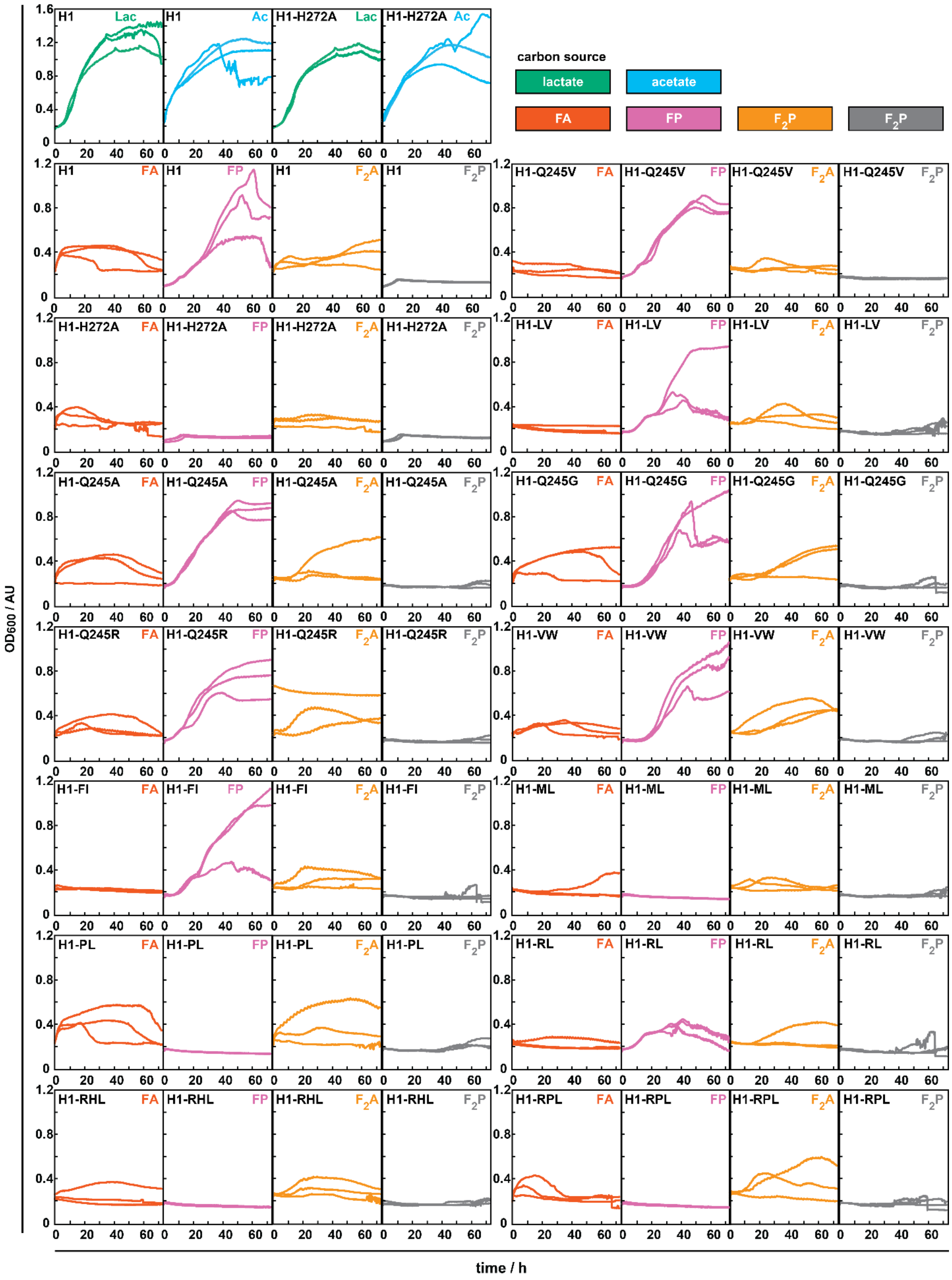

Figure S7: Growth curves (in triplicate) of H1-producing *E. coli* in presence of 100 mM FA (red), FP (purple), F_2A (orange), and F_2P (grey). Additionally, the growth curves for H1 and H1-H272A for lactate (green) and acetate (blue) are shown at the top. These growth curves are representative for all H1-variants. Top right, a legend for the color-code of the carbon sources used.

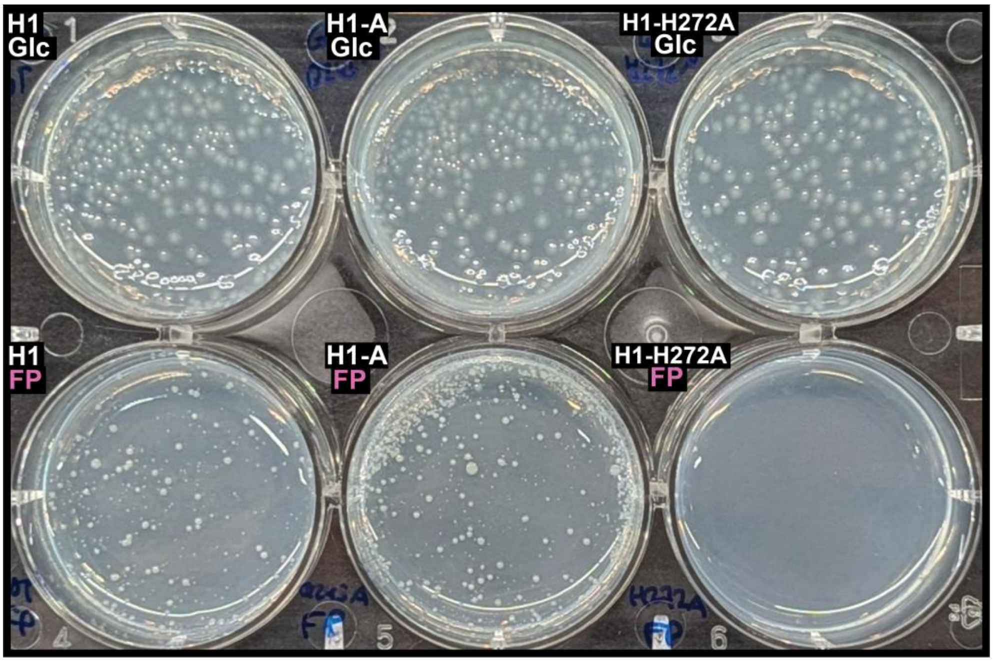

Figure S8: Selective MMV agar plate containing glucose (Glc) (top row, positive control) or FP (bottom row) as sole carbon source. Appropriate dilutions are plated of cells harboring the wildtype FAcD-H1 (left), an improved variant H1-A (middle), or the inactive variant H1-H272A (right). Plates contain 17 µg/mL chloramphenicol for plasmid maintenance, 0.1 mM IPTG for protein production, and were incubated at 30 ºC for 7 days. The picture was taken after 4 days of incubation.

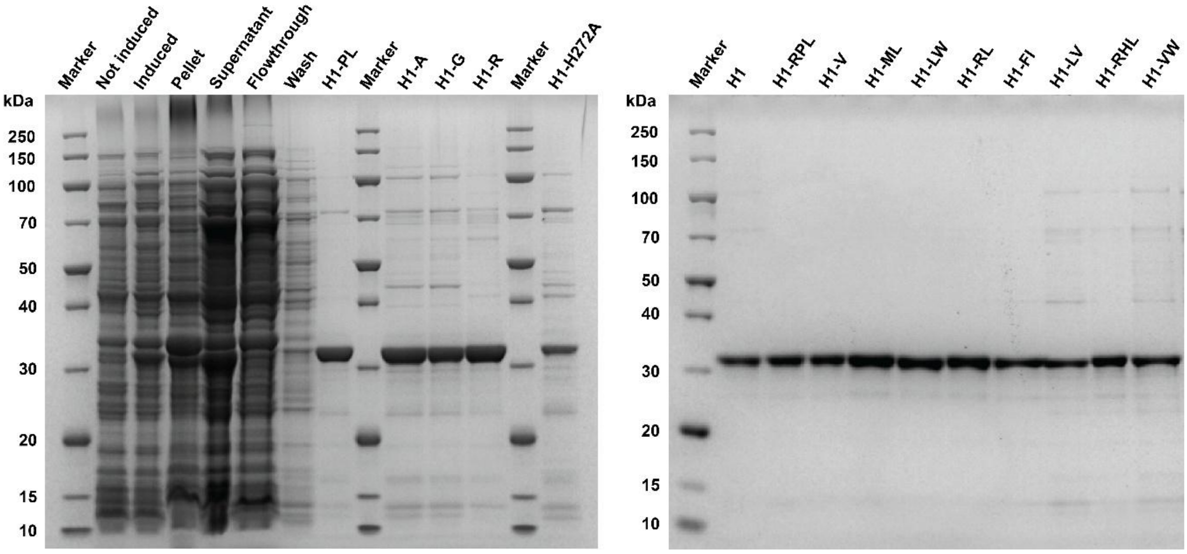

Figure S9: SDS-PAGE analyses of production and purification of H1-variants**.** The gels depict representative fractions throughout the production and purification process for H1-PL and the elution fractions of all H1-variants after purification.

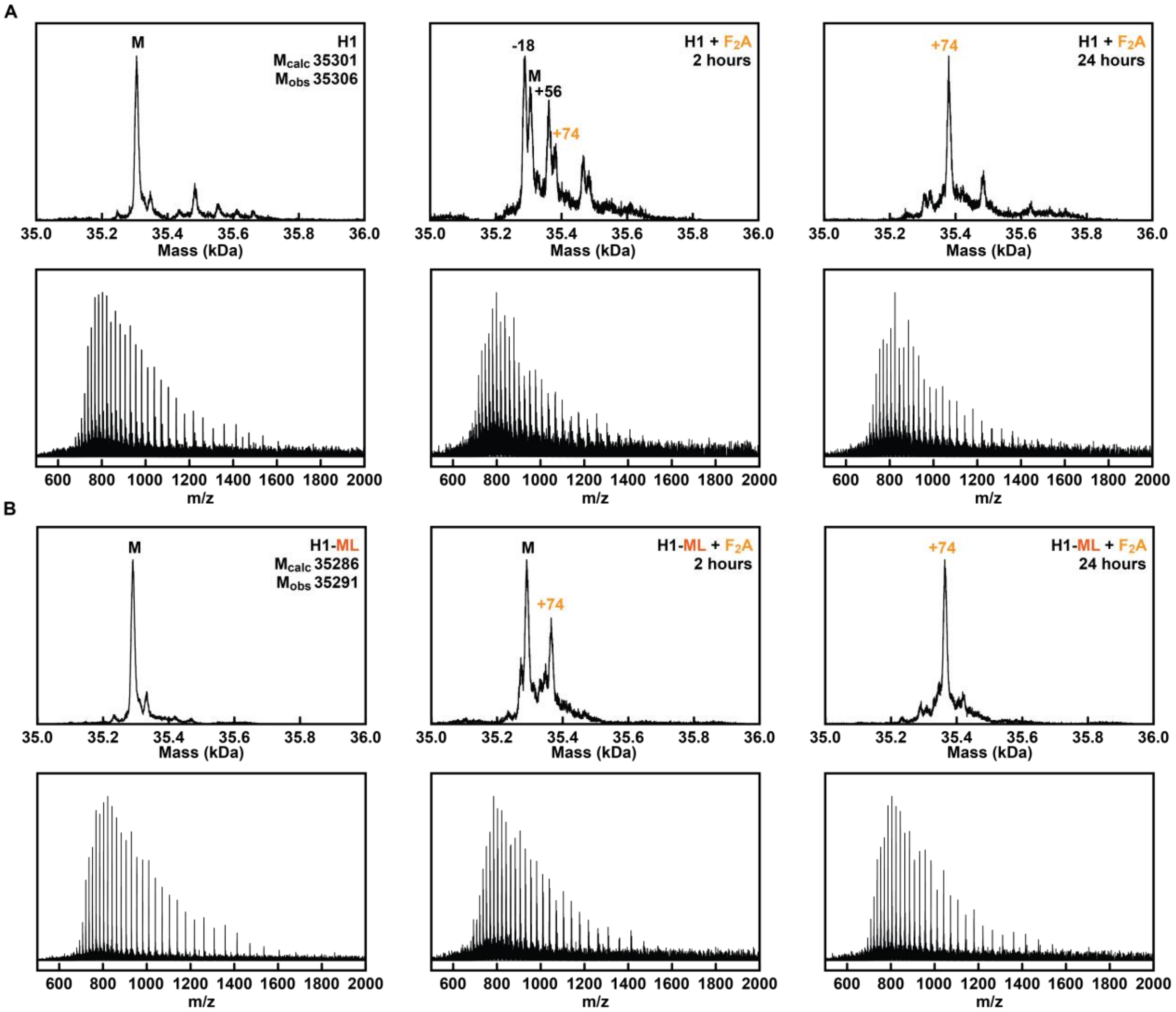

Figure S10: Raw and deconvoluted masses from UPLC-MS spectra before and during F_2A incubation for H1 (**A**) or H1-ML (**B**).

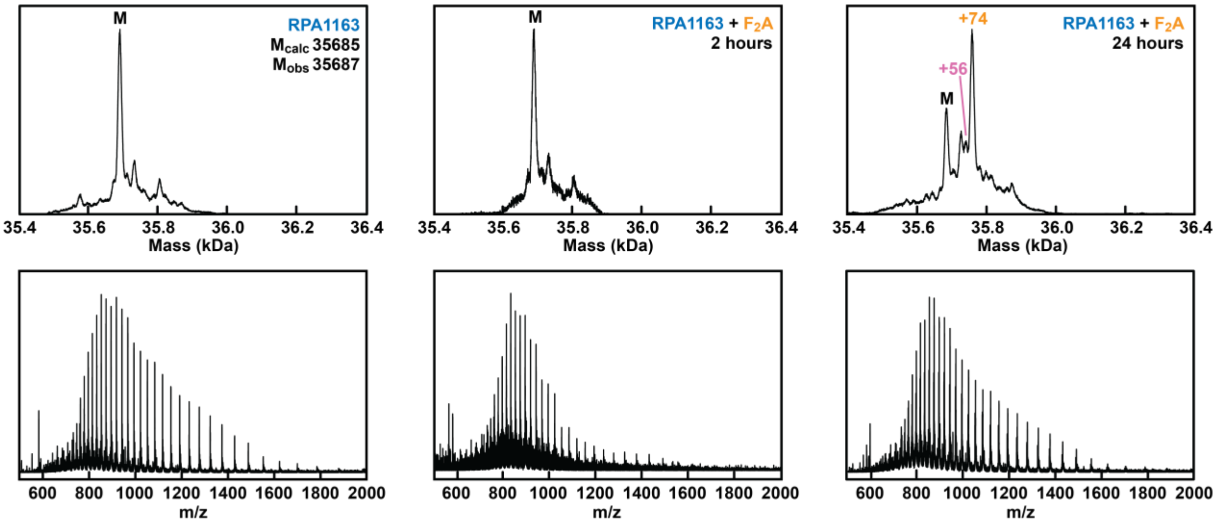

Figure S11: Raw and deconvoluted masses from UPLC-MS spectra for RPA1163 before and during F_2A incubation.

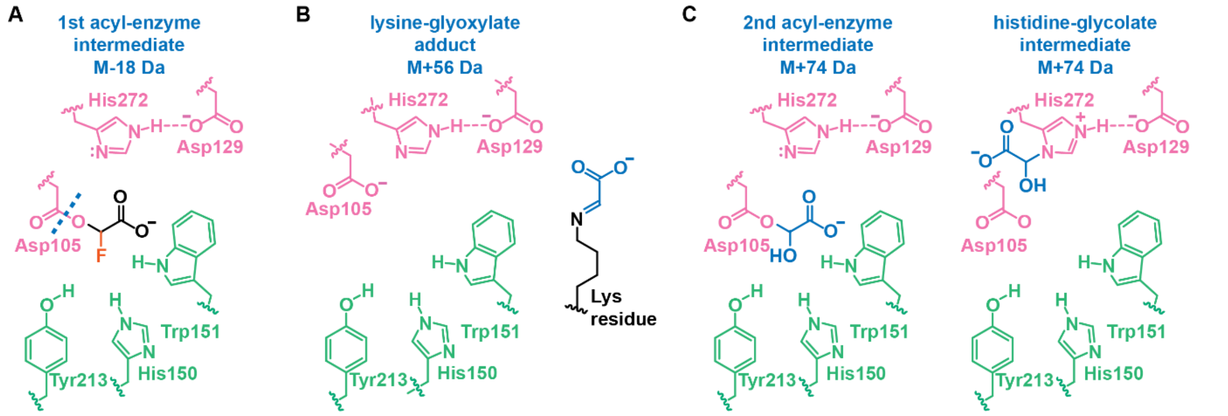

Figure S12: A-C: Potential structures of tentatively assigned species observed in UPLC-MS studies following the incubation of H1-variants with F_2A. **A:** The M-18 Da species arises from the fragmentation of the ester bond in the initial acyl-enzyme intermediate. **B:** The M+56 Da species results from the condensation of an unknown lysine residue (or N-terminal amine) with glyoxylate. **C:** The M+74 species could indicate the presence of the second acyl-enzyme intermediate or the glycolation of a nucleophilic residue in the active site. As representative example, the structure for the glycolated His272 is depicted.

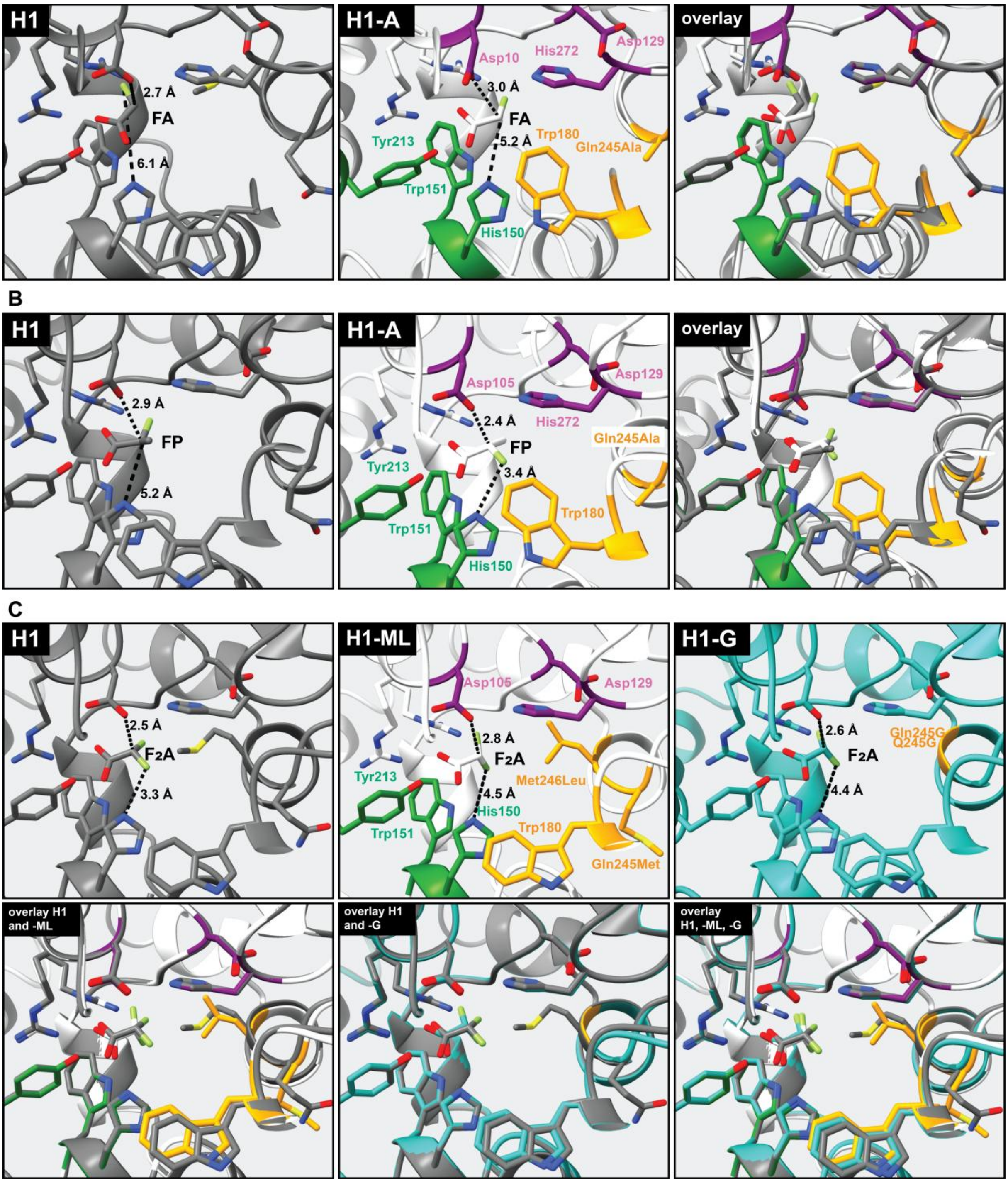

Figure S13: Predicted Boltz-2 structures of H1 and improved H1-variants with FA (**A**), FP (**B**), or F_2A (**C**) docked into the active sites. The respective H1-variants are indicated in the figure. The wildtype H1 model is always depicted in grey. Residues in regions of interest are colored, i.e. green is the halide pocket (H150, W151, Y213), purple is the catalytic triad (D105, D129, H272), and in orange are targeted positions of interest (W180, Q245, M246). Ser147 is not included, as this residue was not substituted in any of the depicted H1-variants. Various overlays are included, as well as distances (in Å) to allow comparison of the orientation of critical residues. Specifically, the distance between the closest oxygen of D105 and the substrate's C_α is shown, as well as the distance between the closest fluorine and N_ε of H150. Models must be interpreted with caution, as the ligand is not in an orientation suitable for nucleophilic attack (see main text). The models are available for download in the Supporting Data.

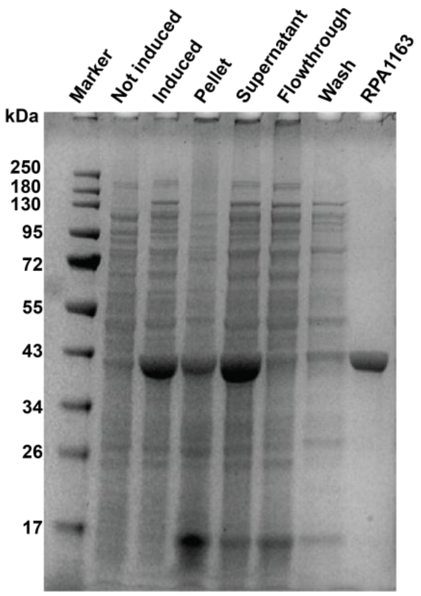

Figure S14: SDS-PAGE analysis of production and purification of RPA1163.

2. Supporting Tables

Supporting Table S1: Comparison of existing approaches to assay FAcD activity.

Ref.	Journal, year, volume, page nr.	Screening/selection method	# of FAcD variants	purified enzyme / cell lysate / in vivo	Notes
[1]	J. Bacteriol. **2009**, 191:2630	colorimetric glycolate detection	**18**	purified enzymes	pinpointed role of active site residues of FAcDEX-FA1
[2]	Microb. Biotechnol. **2010**, 3:107-	96-well pH indicator assay (phenol red)	**193**	purified enzymes	aimed at identifying novel dehalogenases by elucidating sequence determinants for dehalogenase activity
[3]	J. Am. Chem. Soc. **2011**, 133:7461	96-well pH indicator assay (bromothymol blue)	**6**	purified enzymes	study the role of active site / halide pocket residues
[4]	Lett. Appl. Microbiol. **2011**, 53:417	colorimetric agar screen (zirconium-xylenol orange reagent)	**n.d. (soil sample)**	in vivo	serial passages of soil sample identified defluorinating *Burkholderia* bacteria.
[5]	Environ. Intern. **2019** 131:104999.	Gas chromatography or GC-MS	**1**	purified enzyme	investigate FAcD-catalyzed defluorination of various substrates
[6]	ACS Catal **2020,** 10:3143	Gas chromatography	**5**	purified enzymes	semi-rational design of FAcDs active on bulky organofluorides
[7]	Environ. Sci. Technol. **2021**, 55:9817	HRMS / fluoride ion electrode	**1**	purified enzyme	study the mechanism FAcD-catalyzed defluorination
[8]	Appl. Environ. Microbiol. **2022**, 88:e00288-22	96-well colorimetric assay (lathanum-alizarin reagent)	**65**	whole cells	assay development to assess defluorination by the organism *Pseudomonas putida* F1
[9]	FEBS J. **2023**, 290:4966	96-well pH indicator assay (phenol red)	**19**	purified enzymes	probing role of active side residues
[10]	Small **2023**, 19:2205232	fluorogenic RNA-based biosensor coupled to droplet sorting microfluidics	**2**	in vivo	Development of F^- biosensor. Model screening of two *E. coli* carrying either FAcD (positive control) or DhlA (a dechlorinase, negative control)
[11]	ACS Omega **2024**, 9:28546	^{19}F-NMR, mass spectrometry, fluoride ion electrode	**5**	purified enzyme	characterization of recently identified *Delftia acidovorans* defluorinases
[12]	PNAS **2025**, 122:e2504122122	96-well colorimetric assay (zirconium-xylenol orange reagent)	**495**	cell supernatants	alanine scanning to determine critical residues for defluorination activity, domain swap to unlock weak defluorinase activity
[13]	mBio **2025**, 16:e0179825	various pH-indicator screens in 96-well plates, liquid/solid media, and hydrogels	**nd. (single variants and consortia)**	purified enzymes / in vivo	Various applications of pH-based assays to identify new substrates and estimate relative rates

Supporting Table S2: Apparent initial rates for FAcD-H1 and H1-H272A. Numbers corresponding to **Fig. 2A** are given here. Wildtype H1 rates were determined from biological duplicates. Rates are based on F^- release measured by ^{19}F-NMR spectroscopy (see *Experimental* for details). Standard deviations are given. Traces of F^- (<1%) detected in F_2P assays are ascribed to substrate or buffer impurities. Note: n.d. stands for 'not detected'.

**Enzyme**	**FA**	**FP**	**F_2A**	**F_2P**
	$v_{0,app}$ (s^{-1})	$v_{0,app}$ (s^{-1})	$v_{0,app}$ (s^{-1})	$v_{0,app}$ (s^{-1})
**H1**	32 ± 4.5	1.9 ± 0.36	$3.5 \times 10^{-3} \pm 9.4 \times 10^{-4}$	n.d.
**H1-H272A**	n.d.	n.d.	n.d.	n.d.

Supporting Table S3: The transformation efficiency was based on the colony-forming units (cfus) and the total culture volume. For transformations into NEB 10-beta, a negative control was included in the Golden Gate restriction-ligations which contained no insert DNA. This control gave an estimate of background, i.e. colonies harboring an empty vector. Note: n.a. stands for 'not applicable'.

	**Host**	**Method**	**# cfus on diluted plate**	**# transformants (corrected for dilution)**	**Coverage of theoretical genetic diversity**	**Background**
**Lib-D1**	NEB 10-beta	Chemical transformation	50	2.0×10^4	$2.0 \times 10^4 / 32^2 =$ 20	4.0×10^2 (2%)
**Lib-D2**	NEB 10-beta	Chemical transformation	36	4.0×10^3	$4.0 \times 10^3 / 32^2 =$ 3.9	1.1×10^2 (3%)
**Lib-D3**	NEB 10-beta	Chemical transformation	71	7.9×10^3	$7.9 \times 10^3 / 32^2 =$ 7.7	1.1×10^2 (1%)
**Lib-T1**	NEB 10-beta	Electroporation	392	4.4×10^5	$4.4 \times 10^5 / 32^3 =$ 13	4.4×10^2 (0.1%)
**Pop-D1**	BL21(DE3)	Chemical transformation	17	3.4×10^2	n.a.	n.a.
**Pop-D2**	BL21(DE3)	Electroporation	42	4.2×10^6	n.a.	n.a.
**Pop-D3**	BL21(DE3)	Electroporation	143	1.4×10^7	n.a.	n.a.
**Pop-T1**	BL21(DE3)	Electroporation	79	7.9×10^6	n.a.	n.a.

Supporting Table S4: Sanger sequencing results of 101 random single colonies throughout the selection campaigns. Variants chosen for characterization are marked in green. Codons that were not randomized are blocked off in gray. See also the results from whole-plasmid sequencing (**Figs. 3C-D** and **Supporting Fig. S4**).

Population	Clone no.	S147 codon	S147	W180 codon	W180	Q245 codon	Q245	M246 codon	M246	Notes, additional substitutions/mutations
**Pop-D1** FP plate reader screening, random clones with growth phenotype	1			TGG	W	AGT	S			
	2			TGG	W	GCG	A			
	3			TGG	W	GCG	A			R230H (C**G**C->C**A**C), F83F (TT**C**->TT**T**)
	4-5			TGG	**W**	GGG	**G**			
	6			TGG	W	TGG	W			A146T (**G**CA->**A**CA), Q191K (**C**AG->**A**AG), A280A (GC**T**->GC**C**)
**Pop-D2** FP plate reader screening, random clones with growth phenotype	1	TGG	W			GCG	A			
	2	TCG	S			GCG	A			
	3	GTG	**V**			TGG	**W**			
	4	TCG	S			GAG	E			
**Pop-D1 Round 2** FP selection	1-6			TGG	**W**	GCG	**A**			
**Pop-D1 Round 1** FA selection	1-6			TGG	**W**	CGG	**R**			
**Pop-D2 Round 1** FP selection	1-11	TTG	L			TGG	W			
	12	TCG	S			AAG	K			
	13	GGG	G			TGT	C			
	2 clones are removed due to ambiguous or failed sequencing results									
**Pop-D2 Round 2** FP selection	1-7	TTG	**L**			TGG	**W**			
	3 clones are removed due to ambiguous or failed sequencing results									
**Pop-D3 Round 1** FP selection	1-3					CAG	Q	ATG	M	
	4					ACG	T	ATG	M	
	5					AAG	K	ATG	M	
	6-7					GTG	V	ATG	M	V232V (GT**T**-> GT**G**)
	8					AGG	R	ATG	M	C176C (TG**C**->TG**T**), S79S (TC**A**->TC**C**)
	9					GCG	A	ATG	M	Q229Q (CA**G**-> CA**A**)
	10					GAG	E	ATG	M	
	11					ATG	**M**	CTG	**L**	
	12					TTT	**F**	ATT	**I**	
	13					TTG	L	TTG	L	Poor seq. quality
	14					ACG	T	GGG	G	
	1 clone is removed due to ambiguous or failed sequencing results									
**Pop-D3 Round 2** FP selection	1-10					GTG	**V**	ATG	**M**	V232V (GT**T**-> GTG)
**Pop-T1 Round 1** FP selection	1	TCG	S			AGT	S	ATG	M	
	2	CCG	P			TTG	L	ATG	M	
	3	CGG	**R**			CCG	**P**	CTG	**L**	A46V (G**C**C -> G**T**C)
	4	TAG	STOP			CCG	P	TTG	L	R81H (C**G**C->C**A**C)
	5	TAG	STOP			TTG	L	CTG	L	Base (G) insertion at G247, causing frameshift
	6	GCG	A			CAG	Q	ATG	M	Poor seq. quality
	7-13	Partial (2 out of 7) or complete (5 out of 7) gene deletion								
	7 clones are removed due to ambiguous or failed sequencing results									
**Pop-T1 Round 2** FP selection	1-5	TTG	**L**			GTG	**V**	ATG	**M**	
	6	GCG	A			CAG	Q	ATG	M	Poor seq. quality
	7-18	Gene deletion								
**Pop-T1 Round 1** F_2P selection	1	CCG	**P**			TTG	**L**	ATG	**M**	
	2-3	CGG	**R**			CAT	**H**	CTG	**L**	
	4	CGG	**R**			TTG	**L**	ATG	**M**	

Supporting Table S5: Panel of H1 variants chosen for characterization and their origins. The carbon source that was used during selections is given in brackets**.**

**Defluorinase shorthand**	**Substitutions in H1 enzyme**	**Library source and rationale**
**H1-A**	Q245A	Pop-D1 round 2 (FP), most common variant. Also found in initial plate reader assessment.
**H1-LW**	S147L-Q245W	Pop-D2 round 1 and 2, most common variant.
**H1-V**	Q245V	Pop-D3 round 2 (FP), most common variant.
**H1-LV**	S147L-Q245V	Pop-T1 round 2 (FP), most common variant after wildtype.
**H1-R**	Q245R	Pop-D1 round 1 (FA), sole surviving variant after FA selection.
**H1-G**	Q245G	Pop-D1 (FP), found twice in initial plate reader assessment with growth phenotype.
**H1-VW**	S147V-Q245W	Pop-D2 (FP), found in initial plate reader assessment with growth phenotype.
**H1-FI**	Q245F-M246I	Single colony from Pop-D3 round 1 (FP) that had an uncommon Met246 substitution.
**H1-ML**	Q245M-M246L	Single colony from Pop-D3 round 1 (FP) that had a Met246 substitution to Leu, a substitution that we also identified in the F_2P selection.
**H1-RPL (+A46V)**	A46V-S147R-Q245P-M246L	Pop-T1 round 1 (FP), survivor with similar substitutions to those identified in F_2P selections.
**H1-RL**	S147R-Q245L	Pop-T1 round 1 (F_2P) survivor.
**H1-RHL**	S147R-Q245H-M246L	Pop-T1 round 1 (F_2P) survivor.
**H1-PL**	S147P-Q245L	Pop-T1 round 1 (F_2P) survivor. Also present in Pop-T1 round 1 (FP).

Supporting Table S6: Mass analysis of purified enzymes. Q-ToF MS results confirm the identity of several purified H1 variants in 50 mM Na_2HPO_4 buffer and/or MilliQ. The H272A and PL proteins are present with the expected mass, but also reveal a peak with a mass difference of +176 to 181 Da. We ascribe this to a possible N-terminal gluconoylation of the His-tag[14], although this was not investigated in detail. It is worth noting that these two variants were produced at 18 ˚C instead of the usual 37 ˚C.

Defluorinase variant	Expected (M-Met, Da)	Observed (M-Met, Da)
**H1**	35300.75	**35305.2**
**H1-H272A**	35234.69	**35240.6**, 35417.0 (+176)
**H1-A**	35243.70	**35247.0**
**H1-LW**	35384.91	**35387.6**
**H1-V**	35271.75	**35275.4**
**H1-LV**	35297.83	**35302.7**
**H1-R**	35328.80	**35335.0**
**H1-G**	35229.67	**35233.4**
**H1-VW**	35370.89	**35374.1**
**H1-FI**	35301.76	**35306.4**
**H1-ML**	35285.78	**35288.7**
**H1-RPL (+A46V)**	35348.86	**35353.5**
**H1-RL**	35354.89	**35359.1**
**H1-RHL**	35360.83	**35366.1**
**H1-PL**	35295.82	**35301.2**, 35482.0 (+181)
**RPA1163**	35685.46	**35686.5**

Supporting Table S7: Apparent initial rates and turnover numbers (TON) of purified FAcD-H1 variants. Numbers corresponding to **Fig. 4B** are given here. Wildtype H1 parameters were determined from biological duplicates. H1 variant parameters were determined from technical duplicates unless indicated otherwise. All parameters are based on F^- release measured by ^{19}F-NMR spectroscopy (see *Experimental* for details). Standard deviations are given. Note: n.d. stands for 'not detected'.

	**FA**	**FP**	**F_2A**
**Enzyme**	$v_{0,app}$ **(s^{-1})**	$v_{0,app}$ **(s^{-1})**	**TON**
**H1**	32 ± 4.5	1.9 ± 0.36	307 ± 81 (bio. dup. with tech. replicate)
**H1-H272A**	n.d.	n.d.	n.d.
**H1-A**	63 ± 0.82	3.2 ± 0.21	267 ± 37
**H1-LW**	12 ± 3.8	1.0 ± 0.090	75 ± 2
**H1-V**	47 ± 0.16	2.0 ± 0.093	174 ± 19
**H1-LV**	18 ± 3.5	1.2 ± 0.080	213 ± 50
**H1-R**	38 ± 5.3	2.1 ± 0.33	147 ± 71
**H1-G**	26 ± 2.1	1.1 ± 0.17	783 ± 21
**H1-VW**	5.7 ± 1.6	0.72 ± 0.079	116 ± 2
**H1-FI**	38 ± 5.8	2.1 ± 0.11	256 ± 86
**H1-ML**	5.9 ± 0.76	0.047 ± 0.0029	1900 ± 161 (tech. tripl)
**H1-RPL (+A46V)**	1.3 ± 0.20	0.022 ± 0.00028	723 ± 27
**H1-RL**	25 ± 0.05	1.3 ± 0.071	232 ± 53
**H1-RHL**	4.7 ± 0.25	0.039 ± 0.0044	1140 ± 95
**H1-PL**	2.1 ± 0.54	0.052 ± 0.0065	134 ± 22 (tech. tripl)

Supporting Table S8: Conversion of FP was determined by measuring fluoride (F^-) release by ^{19}F-NMR spectroscopy over an extended time using purified H1 wildtype (biological duplicates) and H1-A (technical duplicates). In all cases, enzyme concentration was 0.5 µM and FP concentration was 10 mM. Standard deviations are given.

Enzyme	Reaction time	% of F^-
**H1**	5 hours	46.2 ± 2.2
	24 hours	50.9 ± 0.51
**H1-A**	5 hours	50.4 ± 0.2
	24 hours	50.5 ± 0.03

Supporting Table S9: Residue confidence scores (predicted local distance difference test, pLDDT values) of residue Trp180 from Boltz-2 docking simulations of various H1 variants with FA, F_2A and FP. pLDDT is scaled from 0 to 100 and higher values indicate higher confidence. For completion we include the Boltz-2 confidence score, which evaluates the predicted model as a whole and has a range from 0 to 1 (higher values indicate higher confidence).

Model	pLDDT score of Trp180	Boltz-2 confidence score
**H1 with FA**	64.2	0.92
**H1 with F_2A**	63.6	0.92
**H1 with FP**	64.9	0.92
**H1-A with FA**	65.7	0.93
**H1-A with F_2A**	68.3	0.93
**H1-A with FP**	65.0	0.93
**H1-ML with FA**	68.8	0.93
**H1-ML with F_2A**	70.4	0.93
**H1-ML with FP**	68.6	0.93
**H1-G with FA**	70.6	0.93
**H1-G with F_2A**	69.5	0.93
**H1-G with FP**	67.1	0.93

Supporting Table S10: Contents of Minimal Medium with Vitamins (MMV) used for growth-based screening and selection. The salt solution (adapted from literature[15]) was autoclaved for 30 min at 121 ˚C. Prior to use, filtered vitamin solution (of 1000× stock), filtered trace metals solution (of 200× stock) and various (fluorinated) carbon sources were added.

**Salt solution (1×)**		
**Compound**	**g/L**	**mM**
$Na_2HPO_4 \cdot 7\ H_2O$	4.0	14.8
KH_2PO_4	1.4	10.3
$MgSO_4 \cdot 7\ H_2O$	0.4	1.7
$(NH_4)_2SO_4$	1.0	7.6
**Trace metals solution (200×)**[16]		
**Compound**	**g/L**	**mM**
$Ca(NO_3)_2$	780	4.75
$FeSO_4 \cdot 7\ H_2O$	200	0.72
$ZnSO_4 \cdot 7\ H_2O$	10	0.035
H_3BO_4	10	0.16
$CoCl_2 \cdot 6\ H_2O$	10	0.042
$CuSO_4 \cdot 5\ H_2O$	10	0.040
$MnSO_4 \cdot 1\ H_2O$	4	0.024
$Na_2MoO_4 \cdot 2\ H_2O$	3	0.012
$NiCl_2 \cdot 6\ H_2O$	2	0.008
$Na_2WO_4 \cdot 2\ H_2O$	2	0.006
**Vitamin solution (1000×)** in 50/50 ethanol/demi water[15]		
**Compound**	**g/L**	**mM**
Biotin	2.2	0.01
Folic acid	2.2	0.005
p-aminobenzoic acid	200	1.46
Riboflavin	220	0.58
Panthothenic acid	440	2.01
Niacinamide	440	3.60
Pyridoxine · HCl	440	2.14
Thiamine · HCl	440	1.30
**Carbon sources** (1M - 4M stocks, neutralized to pH ≈ 7 – 8 with NaOH when needed)		
**Compound**	**Final concentration**	**Sterilization**
Glucose	11 mM (= 0.2 w/v %)	Filtered
Sodium acetate	75 mM	Filtered
Sodium fluoroacetate	100 mM	None
Sodium DL-lactate	50 mM (racemic)	Autoclaved
Sodium pyruvate	50 mM	None
Sodium glycolate	100 mM	Autoclaved
Sodium 2-fluoropropionate	100 mM (racemic)	None
Sodium 2,2-difluoropropionate	100 mM	None

Supporting Table S11: DNA fragments required for generating H1 NNK libraries via oePCR. Templates and primer pairs are indicated.

**Lib-D1**	**Template(s)**	**Primer pair**	**Fragment size**
Fragment D1-A	pACYC_dehH1	*dehH1_GG_for_BsaI* + *dehH1_W180_rev*	551 bp
Fragment D1-B	pACYC_dehH1	*dehH1_W180NNK_for* + *dehH1_Q245_rev*	217 bp
Fragment D1-C	pACYC_dehH1	*dehH1_Q245NNK_for* + *dehH1_GG_rev_BsaI*	189 bp
Intermediate fragment D1-B-C	D1-B and D1-C	*dehH1_W180NNK_for* + *dehH1_GG_rev_BsaI*	384 bp
Full-length insert Lib-D1	D1-A and D1-B-C	*dehH1_GG_for_BsaI* + *dehH1_GG_rev_BsaI*	913 bp
**Lib-D2**	**Template(s)**	**Primer pair**	**Fragment size**
Fragment D2-A	dehH1 synthesized gene	*dehH1_GG_for_BsaI* + *dehH1_S147_rev*	452 bp
Fragment D2-B	dehH1 synthesized gene	*dehH1_S147NNK_for* + *dehH1_Q245_rev*	313 bp
Fragment D2-C	is the same as fragment D1-C		
Intermediate fragment D2-A-B	D2-A and D2-B	*dehH1_GG_for_BsaI* + *dehH1_Q245_rev*	746 bp
Full-length insert Lib-D2	D2-A-B and D2-C	*dehH1_GG_for_BsaI* + *dehH1_GG_rev_BsaI*	913 bp
**Lib-D3**	**Template(s)**	**Primer pair**	**Fragment size**
Fragment D3-A	dehH1 synthesized gene	*dehH1_GG_for_BsaI* + *dehH1_Q245_rev*	746 bp
Fragment D3-B	dehH1 synthesized gene	*dehH1_Q245NNK_M246NNK_for* *dehH1_GG_rev_BsaI*	189 bp
Full-length insert Lib-D3	D3-A and D3-B	*dehH1_GG_for_BsaI* + *dehH1_GG_rev_BsaI*	913 bp
**Lib-T1**	**Template(s)**	**Primer pair**	**Fragment size**
Fragment T1-A	is the same as fragment D2-A		
Fragment T1-B	is the same as fragment D2-B		
Fragment T1-C	is the same as fragment D3-B		
Intermediate fragment T1-B-C	T1-B and T1-C	*dehH1_S147NNK_for* + *dehH1_GG_rev_BsaI*	480 bp
Full-length insert Lib-T1	T1-A and T1-B-C	*dehH1_GG_for_BsaI* + *dehH1_GG_rev_BsaI*	913 bp

Supporting Table S12: Datasets highlighting the effect of various ^{19}F-NMR measurement protocols on the relative integration of fluoride ion and substrate. sw = spectral width; pw = pulse angle; nt = number of scans; d1 = relaxation delay.

Sample	Protocol	Measurement time	F- (%) measured	Substrate (%) measured
Mixture of 0.75 mM NaF (7.5%) and 9.25 mM F_2A (92.5%)	sw=100 to -240 ppm, pw=45, nt=128, d1 = 1 sec (standard settings)	4 min and 23 sec	5.66	94.34
	sw=100 to -240 ppm, pw=90, nt=128, d1 = 1 sec	4 min and 23 sec	6.95	93.05
	sw=100 to -240 ppm, pw=45, nt=128, d1 = 5 sec	12 min and 55 sec	4.96	95.04
	sw=100 to -240 ppm, pw=45, nt=128, d1 = 10 sec	23 min and 35 sec	4.68	95.32
	sw=100 to -240 ppm, pw=45, nt=128, d1 = 15 sec	34 min and 15 sec	4.50	95.50
	sw=100 to -240 ppm, pw=45, nt=128, d1 = 20 sec	44 min and 55 sec	4.78	95.22
	sw=100 to -240 ppm, pw=45, nt=64, d1 = 20 sec	22 min and 28 sec	4.26	95.74
	sw=100 to -240 ppm, pw=90, nt=128, d1 = 20 sec	44 min and 55 sec	4.84	95.16
Mixture of 1.5 mM NaF (15%) and 8.5 mM F_2A (85%)	sw=100 to -240 ppm, pw=45, nt=128, d1 = 1 sec (standard settings)	4 min and 23 sec	10.84	89.16
	sw=100 to -240 ppm, pw=90, nt=128, d1 = 1 sec	4 min and 23 sec	12.72	87.28
	sw=100 to -240 ppm, pw=45, nt=128, d1 = 5 sec	12 min and 55 sec	9.45	90.55
	sw=100 to -240 ppm, pw=45, nt=128, d1 = 10 sec	23 min and 35 sec	9.36	90.64
	sw=100 to -240 ppm, pw=45, nt=128, d1 = 15 sec	34 min and 15 sec	9.35	90.65
	sw=100 to -240 ppm, pw=45, nt=128, d1 = 20 sec	44 min and 55 sec	9.16	90.84
	sw=100 to -240 ppm, pw=45, nt=64, d1 = 20 sec	22 min and 28 sec	9.0	91.0
	sw=100 to -240 ppm, pw=90, nt=128, d1 = 20 sec	44 min and 55 sec	9.23	90.77
Reaction of 0.5 µM H1 with 10 mM FP, quenched after 5 h	sw=100 to -240 ppm, pw=45, nt=128, d1 = 1 sec (standard settings)	4 min and 23 sec	53.67	46.33
	sw= -20 to -240 ppm, pw=45, nt=128, d1 = 20 sec	44 min and 21 sec	47.75	52.25
Reaction of 0.5 µM H1 with 10 mM FP, quenched after 24 h	sw=100 to -240 ppm, pw=45, nt=128, d1 = 1 sec (standard settings)	4 min and 23 sec	57.50	42.50
	sw= -20 to -240 ppm, pw=45, nt=128, d1 = 20 sec	44 min and 21 sec	50.57	49.43
Reaction of 0.5 µM H1-A with 10 mM FP, quenched after 5 h	sw=100 to -240 ppm, pw=45, nt=128, d1 = 1 sec (standard settings)	4 min and 23 sec	56.43	43.57
	sw= -20 to -240 ppm, pw=45, nt=128, d1 = 20 sec	44 min and 21 sec	50.31	49.69
Reaction of 0.5 µM H1-A with 10 mM FP, quenched after 24 h	sw=100 to -240 ppm, pw=45, nt=128, d1 = 1 sec (standard settings)	4 min and 23 sec	57.94	42.06
	sw= -20 to -240 ppm, pw=45, nt=128, d1 = 20 sec	44 min and 21 sec	50.52	49.48

3. Supporting Discussion

During growth-based screening to validate FAcD-H1 variants, we observed inconsistent growth curves when we utilized FA and F_2A as sole carbon source. In general, growing precultures of bacteria on the poor carbon source acetate resulted in less predictable growth speeds in these experiments. We found this inconsistency only when pre/main cultures were cultivated in 96-well plates with MMV and acetate, while culturing those in tubes (5 mL) under otherwise similar conditions gave the expected phenotype that correlated well with enzyme activity (see *Experimental*). This inconsistency is illustrated well when comparing growth behavior on FA of hosts producing wildtype (H1) or inactive (H1-H272A) variants (**Supporting Fig. S15** below). We also found significant differences in F^- release in culture supernatant of H1-producing hosts depending on which pre/main culturing conditions were applied. This result is relevant, since growth should provide consistent readout of enzyme activity. We did not observe this inconsistency between protocols when pre/main cultures were grown on MMV with glucose (and subsequently, on FP or F_2P in the final plate).

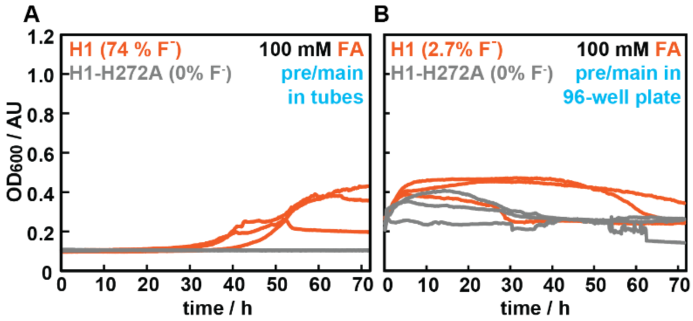

Figure S15: Growth curves of cells producing H1 or H1-H272A when utilizing FA as sole carbon source, with F^- release indicated. A: Growth phenotype when pre/main cultures were grown in tubes. **B:** Growth phenotype when pre/main cultures were grown in 96-well plates.

With these considerations in mind, the growth curves on FA/F_2A observed after cultivating the pre/main cultures in 96-well plates could have the following explanations: (1) The inducer medium added to the 96-well main culture contains 75 mM acetate. Carryover of this carbon source could explain 'growth' on F_2A, since no F_2A consumption was detected (F^-

release, see also **Supporting Fig. S15**). (2) The OD_{600} of the main cultures from MMV with acetate is likely higher than we anticipate (>0.6) upon inoculating the final plate, causing a higher starting OD_{600} at t = 0 h. Due to the low culture volume in the 96-well plates used for the main culture (500 µL), measuring OD_{600} is not straightforward whereas in tubes (5 mL) this is easily monitored using a portable cell density meter. On occasion we, by eye, observed variable OD_{600} across different wells of the pre/main culture. (3) High FA toxicity kills the high number of stationary cells, but the turbidity of dying cells can obscure (poor) growth from survivors. Low F^- release further suggests that cells are no longer in exponential phase, and are struggling to produce sufficient FAcDs.

Due to these issues, growth curves on FA and F_2A for engineered FAcD-H1 variants are left largely out of consideration, and we decided to assess them based on more reliable in vitro characterization instead of optimizing the pre/main culturing conditions.

4. Experimental

Safety Statement: No unexpected or unusually high safety hazards were encountered. Sodium fluoroacetate was handled with extra attention to safety (often in dilute, pH-neutralized aqueous solutions) and stored away when unused.

Materials: Chemicals were purchased from *abcr*, *BLD-Pharm*, *TCI Europe*, and *Sigma-Aldrich*, and used without purification unless noted otherwise. In particular, sodium monofluoroacetate (99%) (FA), sodium 2,2-difluoroacetate (97%) (F_2A), sodium 2,2-difluoropropionate (95%) (F_2P), and 2,3,3,3-tetrafluoropropanoic acid (97%) (4F) were purchased from *abcr*, and 2-fluoropropionic acid (97%) (FP) was purchased from *BLD-Pharm*. *Escherichia coli* strains NEB 10-beta and BL21(DE3) (*New England Biolabs*) were used for cloning and expression/selection experiments, respectively. Bacteria were cultured in Lysogeny broth (LB) medium during non-selective conditions, or Super Optimal Broth (with Catabolite repression) (SOB, SOC) medium during bacterial cell transformation. During selections, bacteria were cultured in a chemically defined Minimal Medium with Vitamins (MMV), supplemented with trace metals, vitamin solution, and various (fluorinated) carbon sources (**Supporting Table S10**). Standard cloning primers and sequencing primers were synthesized by *Eurofins Genomics* (Germany). Primers with degenerate codons (NNK) were synthesized by *Biolegio* (The Netherlands) and *Eurofins Genomics* (Germany). The synthetic genes for FAcD-H1 (dehH1) and RPA1163 were ordered from *Twist Bioscience* (USA). Plasmid isolation kits (QIAprep Spin Miniprep Kits) and PCR and gel clean-up kits (QIAquick PCR Purification Kit, QIAquick Gel Extraction Kit) were purchased from *QIAGEN* (Germany) and used according to the manufacturer's instructions. Sanger sequencing of plasmids and PCR products as well as whole plasmid sequencing (WPS, by *Oxford Nanopore* technology) was

carried out by *Eurofins Genomics* (Germany). In silico cloning and sequence analysis was performed using *Snapgene* (V1.1.3) and *Geneious* (V8.1.9) software. Phusion® High Fidelity DNA Polymerase, GC buffer, deoxynucleotide (dNTP) solution mix, Hi-T4 DNA ligase, T4 DNA ligase reaction buffer, dimethyl sulfoxide (DMSO), BsaI-HFv2, DpnI and CutSmart® buffer were purchased from *New England Biolabs*. Ni-NTA resin (Ni Sepharose 6 Fast Flow) and PD MiniTrap™ G-25 columns were purchased from *Cytiva* (Germany). Precast SDS PAGE gels (SurePAGE™, Bis-Tris, 10x8, 12%, 15 wells) and Tris-MOPS-SDS Running Buffer Powder were purchased from *GenScript* (USA). InstantBlue® Coomassie Protein Stain was purchased from *Abcam* (United Kingdom). Electroporation cuvettes (2 mm) were purchased from *Fisher Scientific* (The Netherlands).

Methods: The concentration of DNA in solutions was determined based on the absorption at 260 nm on a Thermo Scientific Nanodrop 2000 UV-Vis spectrophotometer. The concentration of protein in solutions was determined based on the absorption at 280 nm on the same apparatus. Molar extinction coefficients at 280 nm and molecular weight of proteins were calculated using the ProtParam Expasy web server (https://web.expasy.org/protparam/).

Cellular density by means of optical density at 600 nm (OD_{600}) was measured on an Ultrospec 10 Cell Density Meter (*Biochrom*) in cuvettes or directly from 5 mL cultures. Plate reader growth assays were recorded on a Synergy H1 microplate reader (*BioTek*). ^{19}F-NMR spectra were recorded on a Bruker 400 in 10% D_2O (400 MHz). ^{19}F-NMR spectra were processed and integrated using MestReNova (V12.0.0) software.

UPLC-MS analysis was performed on an Acquity UPLC system (*Waters*) coupled to a quadrupole/time-of-flight (Q-ToF) mass spectrometer (*Waters*) equipped with a PDA detector. The protein samples were injected on a reversed phase ACQUITY UPLC BEH300 C4 1.7 µm column (2.1 mm×150 mm) and the eluent system employed a combination of 0.1% formic acid

in MilliQ (A) and 0.1% formic acid in acetonitrile (B) at a flow rate of 0.3 mL/min. The gradient started with 90% A and 10% B for 2 minutes, then varied linearly from 10 to 50% B (v/v) from 2-10 minutes, 50 to 95% B from 10-11 minutes, kept at 95% B from 11-13 minutes, returning to 5% B from 13-13.1 min, re-equilibration to 5% B from 13.1-20 minutes. The sample injection volume was 3 μL of ca. 3 μM protein. Mass spectra were obtained in the ESI-positive ion mode over a mass range between 500 to 2000 Da.

LC-MS/MS analysis was performed on digested, reconstituted peptides on an Ultimate 3000 RSLC chromatography system (*Thermo Scientific*) coupled to an Exploris 480 Mass Analyser (*Thermo Scientific*). Peptides were loaded into a C18 Trap cartridge (Acclaim Pepmap C18 Reversed Phase Trap Cartridge, 5 μm, 0.3 mm I.D. × 5 mm L., *Thermo Scientific*) and then separated on a reversed phase 1.9 μm nano-LC column (PepSep C18 Repro-Sil AQ 75 μm I.D.× 40 cm, *Bruker*). The eluent system employed a combination of 0.1% formic acid in MilliQ (A) and 0.1% formic acid in 80% acetonitrile (B). The gradient started from 95% A and 5% B, to 35% solvent B over 60 minutes at a flow rate of 0.3 mL/min. Sample injection volume was 2 μL. Eluted peptides were ionized by online nano-electrospray. Data dependent acquisition (DDA) mode was used to obtain high resolution master scans from 385 m/z to 1540 m/z or 250 m/z to 1000 m/z, at a resolution of 120,000 at m/z 200. The ions at +2 to +6 charge states were then selected according to their abundance and fragmented by HCD at 30% normalized collision energy. The resulting fragment ions were measured by data-dependent (dd) MS2 scans at a resolution of 15,000.

LC-MS/MS data was analyzed using PEAKS Studio 12.5 (*Bioinformatics Solutions*) with a MS1 error tolerance of 10 ppm for precursor ions and a fragment ion error tolerance of 0.02 Da. Charge density spectra from UPLC-MS were obtained using MassLynx (V4.1) software and subsequently deconvoluted using MagTran (V1.03)[17] software, for H1 variants using a mass range of 35,000-36,000 (35,200-35,500 when zoomed in), a charge range of 1-

100, a S/N threshold of 5 and a max. no of species of 5. The same settings were applied for RPA1163, except using a mass range of 35,400-36,400.

Competent cells: Chemically competent cells were made using the Inoue method[18]. Electrocompetent cells were made using glycerol/mannitol density step centrifugation[19] and were electroporated on the same day to maximize transformation efficiency.

Assembly of pACYC_dehH1 and pACYC_RPA1163: All cloning and growth experiments were performed using the selection plasmid pACYC_GG, our in-house expression plasmid based on the commercially available pACYCDuet-1 whose design and construction has been described in our earlier work.[20] In brief, this vector harbors a p15A origin of replication, encodes the chloramphenicol resistance gene *cat*, and features two multiple cloning sites (MCSs) specific for type IIS restriction enzymes BsaI or Esp3I, enabling the modular exchange of two target genes via Golden Gate Assembly. Both positions are under IPTG-inducible promoters (T7), and the vector also introduces an N-terminal His-tag on the gene cloned into MCS1. In this work, MCS1 was used for all dehH1 variants, libraries, and RPA1163, and MCS2 was always left empty.

The amino acid sequence for our target enzyme, FAcD-H1 (DehH1 as given on Uniprot Q01398), was converted to a DNA sequence and codon optimized for *E. coli* using the online Codon Optimization Tool from Integrated DNA Technologies. The same was performed for RPA1163 from *Rhodopseudomonas palustris* CGA009 (Q6NAM1 DEHA_RHOPA on UniProt). The sequence of interest was flanked by BsaI recognition sites and was purchased as a synthetic gene (see *Sequences*). This *dehH1* or *RPA1163* gene was then cloned into MCS1 of pACYC_GG by Golden Gate Assembly with Hi-T4 DNA ligase and BsaI-HFv2. The following thermocycler program was used for the assembly: (1) 30 cycles alternating between 37 ˚C and

16 ˚C for 5 and 10 min respectively, (2) a final digestion step at 55 ˚C for 20 min, and (3) an enzyme inactivation step at 65 ˚C for 20 min. The assembly reactions were transformed into chemically competent *E. coli* NEB10-beta cells. A single colony was picked from LB plates containing chloramphenicol (34 µg/mL) and used to inoculate 5 mL LB medium with chloramphenicol (34 µg/mL). Bacteria were grown overnight, plasmids isolated, and successful assembly of pACYC_dehH1 or pACYC_RPA1163 was confirmed by Sanger sequencing with *MCS1_Up* and *DuetDOWN1*. For protein expression and/or growth-based selections (described later), the plasmid was transformed into chemically competent *E. coli* BL21(DE3).

Site-directed mutagenesis for making pACYC_dehH1_H272A: Starting from plasmid pACYC_dehH1, mutagenic primers *dehH1_H272A_fw* and *dehH1_H272_rv* were used to generate a control plasmid that would encode an inactive H1 variant in which the histidine base of the catalytic triad was substituted by an alanine (H272A). The PCR reaction was performed in MilliQ water with Phusion-HF DNA polymerase, 200 µM dNTPs, 0.5 µM forward and 0.5 µM reverse primer, 1X GC buffer, 3% DMSO, and ~1 ng template DNA in a 50 µL reaction. The following thermocycler settings for this QuikChange were used: (1) initial denaturation at 95 ˚C for 3 min, (2) 16 cycles of denaturation at 95 ˚C for 30 s, annealing at 63 ˚C for 30 s, and extension at 72 ˚C for 1:30 min; (3) a final extension at 72 ˚C for 10 min. PCR product formation was confirmed by agarose gel electrophoresis, and the resulting PCR product was digested with DpnI for 1 hour at 37 ˚C to remove remaining template DNA. Next, the DNA was transformed into chemically competent *E. coli* NEB10-beta cells. A single colony was picked from LB plates containing chloramphenicol (34 µg/mL) and used to inoculate 5 mL LB medium with chloramphenicol (34 µg/mL). Bacteria were grown overnight, plasmids isolated, and successful generation of pACYC_dehH1-H272A was confirmed by Sanger sequencing

with *DuetDOWN1*. For protein expression and/or growth-based selections (described later), the plasmid was transformed into chemically competent *E. coli* BL21(DE3).

AlphaFold model and residue selection mutagenesis: Currently, no crystal structure is available for H1. To allow visual inspection of the enzyme structure, an AlphaFold2-Multimer model[21] was generated of the wildtype sequence of H1, which encodes a homodimer. To do so, the open source ColabFold platform was used.[22] In total, four residues were targeted for NNK randomization. Trp180 was inspired by a work from the Wang group where, in a homologous fluoroacetate dehalogenase (RPA1163 from *Rhodopseudomonas palustris* CGA009), its substitution for smaller residues made room for bulkier α-fluorocarboxylic acids.[6] After submitting our model to the online webserver HotSpot Wizard[23], Gln245 and Ser147 were identified as functional hot spots (i.e. mutable residues not involved in catalysis, but positioned near the catalytic pocket) and thus also chosen for mutagenesis. Met246 was chosen following visual inspection of the model, as it was pointing towards the active site and was conveniently positioned next to Gln245. With epistatic interactions in mind, it was only randomized in combination with Gln245. All libraries were generated as double or triple NNK libraries, in order to search for potential epistatic effects and increase library size. The combinations of randomized residues were Lib-D1 (W180NNK + Q245NNK), Lib-D2 (S147NNK + Q245NNK), Lib-D3 (Q245NNK + M246NNK), and Lib-T1 (S147NNK + Q245NNK+ M246NNK). At an early stage of the project we solely randomized the equivalent residue of Trp180 in a homologous enzyme, and this resulted in full reversion to W180W, or to the weakly active W180F (data not shown). Because the H1 double library Lib-D1 again fully reverted to W180W, we did not target Trp180 again, nor construct a library in which all four residues were simultaneously targeted.

Generation of NNK libraries: Overlap extension PCR (oePCR) with primers bearing degenerate NNK codons was employed to randomize the targeted positions. Starting from pACYC_dehH1 or the ordered dehH1 gene, two or three DNA fragments with partially overlapping ends were generated using PCR with various *NNK_for* mutagenic primers in combination with their respective *_rev* primers (see **Supporting Table S11**). The partially overlapping fragments were subsequently used as templates and were merged together by oePCR. Some fragments were interchangeable between libraries as they randomized the same parts of the sequence. In our hands, coupling of more than two fragments by oePCR directly did not produce sufficient product. Therefore, an intermediate fragment was generated that merged two fragments together when required. In the final oePCR, amplification of an (intermediate) fragment together with its complementary partially overlapping fragment in the presence of primers *dehH1_GG_for_BsaI* and *dehH1_GG_rev_BsaI* yielded the full-length 913 bp construct (**Supporting Table S11**).

In total, three (oe)PCR reactions were required per library to create the full-length insert. The following PCR protocol was used: (1) initial denaturation at 95 ˚C for 3 min, (2) (5+)25 cycles of denaturation at 95 ˚C for 30 s, annealing at 64˚C for 30 s and extension at 72 ˚C for 15 s; (3) a final extension at 72 ˚C for 10 min. For oePCR reactions, the primers were added to the PCR reaction mixture after 5 cycles. All PCR products were separated on a 0.8 % agarose gel and excised from the gel to remove unspecific amplification products and remove template DNA. All PCR reactions were performed in MilliQ water with Phusion-HF DNA polymerase, 200 µM dNTPs, 0.5 µM forward and 0.5 µM reverse primer, 1X GC buffer, 3% DMSO, and ~1 ng template DNA in 50 µL reactions. In case of poor DNA yields, up to 8 reactions of 50 µL were pooled together prior to gel extraction, rather than increasing the number of cycles so as not to introduce PCR bias.

To assemble the selection plasmids, the partially randomized CDS was cloned into pACYC_GG using Golden Gate Assembly with Hi-T4 DNA ligase and BsaI-HFv2 as described earlier. A negative control was included that did not contain insert DNA but solely the target vector, to determine the number of background transformants which harbored empty vectors, which was generally very low (≤3%, see **Supporting Table S3**). The entirety of the restriction-ligation reactions was transformed into NEB 10-beta cells, either by heat shock into chemically competent cells for the double libraries Lib-D1, Lib-D2 and Lib-D3, or by electroporation for the triple library Lib-T1. The number of transformants was calculated based on the number of colony-forming units and final culture volume (**Supporting Table S3**). All colonies were scraped from large selective LB agar plates (~200 mL) containing chloramphenicol (34 µg/ml). Library plasmid DNA was isolated and sequenced with *DuetDOWN1*, *MCS1_Up* and whole-plasmid sequencing to verify library quality and confirm successful randomization (see also the section on *Sequencing analysis*). The isolated plasmids were stored at -20 ˚C until the start of a selection or screening experiment.

Growth-based screening of *E. coli* producing H1 or H272A in the plate reader with preculturing in tubes: This protocol had some minor adaptations based on the carbon source used (*vide infra*) and whether or not the pre- and main cultures were incubated in tubes or in 96-deep well plates (see next section). The overall workflow is the same, however.

Precultures of 5 mL Minimal Medium with Vitamins (MMV, see **Supporting Table S10**) with 34 µg/mL chloramphenicol and 11 mM glucose (= 0.2 w/v %) as sole carbon source were inoculated with single colonies from fresh agar plates with *E. coli* BL21(DE3) cells harboring pACYC_dehH1 or pACYC_dehH1-H272A. The precultures were incubated at 37 ˚C with moderate shaking (135 rpm) for ≈ 20 h. The following morning, main cultures of 5 mL MMV with 17 µg/mL chloramphenicol and 11 mM glucose were inoculated with 50 µL of the

corresponding preculture and grown at 37 ˚C, 135 rpm, until the optical density at 600 nm (OD_{600}) was approximately 0.3. Then, expression of the dehH1 or dehH1-H272A coding sequence was induced by addition of IPTG (final concentration 1 mM), and expression was allowed for 3 hours at 37 ˚C, 135 rpm. During this step, the final plate assay plate was prepared, i.e. a flat-bottom 96-well plate suitable for a plate reader. In each well was added: (1) 180 µL of MMV containing 17 µg/mL chloramphenicol, 1 mM IPTG, and a variable (fluorinated) carbon source (e.g. 111 mM FP, making the final concentration 100 mM) and (2) 20 µL of the induced main culture ($OD_{600} \approx 0.6$). The following final concentrations were used for each tested carbon source: 100 mM FA, 100 mM racemic FP, 100 mM F_2A, 100 mM F_2P, 100 mM glycolate, 50 mM pyruvate, 50 mM racemic lactate, 75 mM acetate. Assay plates were closed with a transparent plastic lid and transferred into a Synergy H1 microplate reader that had been preheated to 30 °C. While continuously shaking (double orbital, 425 c.p.m.) at 30 °C, growth was monitored by measuring OD_{600} every 10 minutes from the bottom of the wells for a period up to 110 hours (generally around 72 hours was sufficient).

Depending on the carbon source under study in the plate reader, the proceedings regarding the preculture and main culture are slightly different. When growth on C3 carbon sources (FP, F_2P, pyruvate, or lactate) was assessed, precultures and main cultures were supplemented with 11 mM glucose as sole carbon source, and followed the proceedings above. However, when growth on C2 carbon sources (FA, F_2A, glycolate, or acetate) was assessed, precultures and main cultures were supplied with 75 mM acetate as sole carbon source instead, in order already upregulate the expression of genes involved in the glyoxylate shunt.[24] In this case, incubation times also had to be increased due to slower growth rates: the precultures were incubated for at least 26 hours (up to 48 hours), the main culture was incubated overnight (≈ 16 hours) after inoculation, and the induced main culture was allowed to express for 3 – 4 hours.

Additionally, the volume used from the preculture to inoculate the main culture was increased from 50 µL to 100 µL if necessary.

Growth-based screening of *E. coli* producing FAcD-H1 variants in the plate reader with preculturing in 96-well plates: When investigating the growth phenotype of the panel with enriched FAcD-H1 variants after selections, the abovementioned screening protocol was also applied, with some modifications. To exclude any changes to host fitness that could have been the result of spontaneous background mutations in the *E. coli* genome or the selection plasmid backbone, the H1 CDS of the hits was recloned prior to growth screening. Specifically, they were amplified by routine PCR with primers *dehH1_GG_for_BsaI* and *dehH1_GG_rev_BsaI*, gel extracted, and cloned into fresh pACYC_GG vectors by Golden Gate Assembly (as described earlier). These products were transformed into NEB10-beta, isolated plasmids were confirmed by Sanger sequencing with *DuetDOWN1* or whole-plasmid sequencing, and subsequently transformed into BL21(DE3). Then, recloned variants were cultured in 96-well plates (instead of tubes) and assessed in the plate reader for their growth on 75 mM acetate, 100 mM FA, 100 mM F_2A, 50 mM racemic lactate, 100 mM racemic FP, and 100 mM F_2P.

The following growth-screening protocol was applied to check the growth characteristics of single colonies harboring FAcD-H1 variants before and after selection. A 96-deep well plate (pre-plate) filled with 500 µL Minimal Medium with Vitamins (MMV, **Supporting Table S10**) containing 34 µg/mL chloramphenicol and 11 mM glucose (= 0.2 w/v %) was inoculated with single colonies from freshly streaked or transformed *E. coli* BL21(DE3) cells, harboring a selection plasmid (pACYC_dehH1) or a mutated gene variant thereof. For FAcD-H1 variants after selection, colonies were picked in triplicate (technical replicates). The 96-deep well plate was incubated overnight (≈ 18 hours) at 37 °C while shaking at 750 rpm (*Titramax 1000 & Incubator 1000, Heidolph*). The next morning, 25 µL of the

densely grown overnight cultures was used to inoculate a new 96-deep well plate (main plate) containing 500 µL MMV, 17 µg/mL chloramphenicol and 11 mM glucose. After incubating the main culture for 3 hours at 37 °C, 750 rpm, expression was induced by addition of 16.5 µL MMV with 17 µg/mL chloramphenicol, 11 mM glucose, and 30 mM IPTG (final concentration 1 mM IPTG). The plate was incubated at 37 °C, 750 rpm for another 3 hours to allow expression. Then, transparent 96-well assay plates (flat-bottom) were set up as before, by adding 20 µL of the induced main culture (OD ≈ 0.6) to 180 µL of MMV containing 17 µg/mL chloramphenicol, 1 mM IPTG, and a variable (fluorinated) carbon source. Growth was monitored in the plate reader as described earlier.

Also in this case, the protocol has a number of adaptations when growth on C2 carbon sources (FA, F_2A, or acetate) was assessed. In those cases, the pre-plate and main plate were supplied with 75 mM acetate as sole carbon source instead of glucose. The pre-plate was incubated for 40 – 48 hours, the main culture was incubated overnight (≈ 16h) after inoculation, and the induced main culture (induced by addition of 16.5 µL MMV with 17 µg/mL chloramphenicol, 75 mM acetate, and 30 mM IPTG, giving a final concentration of 1 mM IPTG) was allowed to express for 4 hours.

Growth phenotype categorization: To depict the growth characteristics of each variant in a comprehensive manner (i.e. a single figure), OD_{600} after 50 hours of growth was depicted for each well with a white ($OD_{600} = 0$) to blue ($OD_{600} \geq 1$) color gradient. Cells were considered to have a positive growth phenotype on FP when $OD_{600} > 0.2$ at this time, and double-checked by visual inspection of each growth curve relative to its corresponding wildtype and inactive (H1-H272A) control. It should be noted that some clones in **Supporting Fig. S3** showed a growth phenotype early on and were subjected to ^{19}F-NMR analysis after 24 hours of growth (F^- release ranged between 14-30%); therefore, these clones did not have an OD_{600} value after 50 hours of

growth. Nevertheless, these clones were counted towards the number of clones with a positive growth phenotype, and their last measured OD_{600} value (after 24 hours) was depicted with an asterisk.

OD_{600} values depicted in **Supporting Fig. S3** are from single measurements; values in **Fig. 4A** are averaged from technical triplicates (corresponding growth curves are given in **Supporting Fig. S7**).

Preparation of ^{19}F-NMR samples from cultures: During culturing, fluoride release from successful fluorinated substrate conversion was periodically measured by ^{19}F-NMR spectroscopy. For selection cultures, 110 µL of a 5 mL culture was removed by pipetting. For wells of interest in a 96-well plate, the plate reader was paused after 24, 72, or 110 hours of incubation, 110 – 200 µL was removed from wells of interest by pipetting, and the plate reader was resumed. To prepare the samples for ^{19}F-NMR, cells were spun down by centrifugation (10 min, 13,000 rpm) and 100 µL of supernatant was mixed with 350 µL MilliQ and 50 µL D_2O. The mixture was transferred to an NMR tube and F^- release was determined by ^{19}F-NMR (see *^{19}F-NMR analysis*).

Growth-based selections of FAcD-H1 variants from libraries in liquid media: Chemically competent or freshly prepared electrocompetent *E. coli* BL21(DE3) were transformed with library plasmids of Lib-D1, Lib-D2, Lib-D3, or Lib-T1. Following recovery, a fraction of the cells was plated on LB agar plates with 34 µg/mL chloramphenicol to calculate the number of transformants (**Supporting Table S3**). All transformants were grown directly from the recovering cells by topping the medium up to 4 mL SOB, adding 34 µg/mL chloramphenicol to kill cells devoid of a plasmid, and by growing the cultures for ~20h at 30 °C, 135 rpm. Precultures of controls BL21(DE3) pACYC_dehH1 or pACYC_dehH1-H272A were

inoculated in 4 mL LB with 34 μg/mL chloramphenicol and grown overnight at 37 °C, 135 rpm. These controls were grown alongside the selection cultures under the same conditions. Following incubation of the precultures, main cultures of 5 mL MMV and 17 μg/mL chloramphenicol, supplemented either with 11 mM glucose (Glc) or with 50 mM acetate (Ac) as carbon source, were inoculated with 50 μL – 200 μL of the corresponding preculture. Main cultures were incubated at 37 °C, 135 rpm until an OD ≈ 0.2 was reached. For the Glc cultures, this was after approximately 3 – 4 hours; for the Ac cultures, this required approximately 7 – 8 hours. Gene expression was induced by addition of 1 mM IPTG and main cultures were incubated for 3 more hours at 37 °C, 135 rpm. During this incubation step, selection medium was freshly prepared, containing 5 mL MMV, 1 mM IPTG, 17 μg/mL chloramphenicol, and a variable fluorinated carbon source: 100 mM FA, 100 mM F_2A, 100 mM FP, or 100 mM F_2P.

To start the selections, the induced cultures were diluted 1:100 in selection medium with FP or F_2P from Glc main cultures, and were diluted 1:50 in selection medium with FA or F_2A from Ac main cultures. Selection cultures were grown at 30 °C (for Lib-D2, Lib-D3, or Lib-T1) or 37 °C (for Lib-D1), 135 rpm while routinely measuring OD_{600} using a portable cell density meter. Since the carbon sources are poor, growth of libraries required at least 3–4 days for FP and >1 week for FA. Therefore, OD_{600} values of 0.3–0.4 were generally considered to be sufficient to continue to the next passage, which would be started by diluting 1:100 in fresh selection medium. In this way, one (on FA) or two (on FP) selection rounds were performed.

During the selections, ^{19}F-NMR samples of the supernatant would occasionally be measured (as described earlier) to check either if traces of defluorination could be detected prior to observable measurable cell density, or to confirm that observable growth was the result of active defluorination. Furthermore, selection survivors were routinely plated from the selection cultures on LB agar plates with 34 μg/mL chloramphenicol using appropriate dilutions. A 25%

glycerol stock was made of the mixed populations prior to selection and after passage, to enable long-term storage.

For the selections on F_2P, no measurable growth was observed after 14 days incubation, so 200 µL of supernatant was plated without dilution on LB agar plates containing 34 µg/mL chloramphenicol to assess cell viability. Surviving colonies of the most promising library were collected, pooled plasmids were isolated, and sent for whole plasmid sequencing (WPS).

Growth-based assay of FAcD variants on solid media: Single colonies of BL21(DE3) cells harboring pACYC_H1, pACYC_H1-A, or pACYC_H1-H272A were picked from freshly streaked plates and used to inoculate 5 mL MMV precultures containing 11 mM Glc as sole carbon source and 34 µg/mL chloramphenicol. Following overnight incubation at 37 °C, 135 rpm, 30 µL of culture was used to start 3 mL main cultures (MMV with 11 mM Glc and 17 µg/mL chloramphenicol). Main cultures were incubated at 37 °C, 135 rpm, until OD was ~0.4 and then induced (1 mM IPTG). Expression was allowed at 37 °C, 135 rpm for 3 hours, during which a fresh selective plate was prepared in a sterile 6 well cell culture plate. The salt solution for MMV (**Supporting Table S10**) and agar solution were autoclaved separately as a 2× stock and mixed 1:1 prior to use. For the final composition, the plates contained agar (15 g/L), MMV as described (**Supporting Table S10**) supplied either with 11 mM Glc or 100 mM FP (racemic) as sole carbon source, 17 µg/mL chloramphenicol and 0.1 mM IPTG. Each well contained 4 mL of volume. The main cultures were diluted 6×10^5-fold using MMV salt solution without carbon source, 50 µL was plated on each well and spread with beads. Plates were incubated at 30 °C for 7 days and routinely inspected for colony formation.

Sequencing analysis throughout selections: The change in composition of the population during the selections was monitored by Sanger sequencing (with *DuetDOWN1* or *MCS1_Up*)

of single colonies, and by whole plasmid sequencing (WPS) of pooled plasmids from mixed populations. To increase plasmid yields, LB medium with 34 µg/mL chloramphenicol was used to start cultures from mixed populations, prior to selection and after each passage. After overnight growth their plasmids were isolated and sent for sequencing.

To analyze WPS data, raw reads were assembled to the reference plasmid (pACYC_dehH1) in *Geneious*, and mutated codons across randomized positions were extracted. Before quantification, invalid data was removed: as some reads sequenced the area outside of the CDS, these invalid codons ('---') were trimmed from the dataset using an Excel script. Furthermore, during one Pop-T1 selection campaign, hitchhiker cells had excised the dehH1 CDS, in which case the reads missing the CDS (49% of reads) were removed manually. After curating the data, all extracted NNK codons were translated to the respective amino acids and their ratios were determined using another Excel script. Once the composition of each population did not change significantly anymore, they were considered fully enriched.

Comparison of sequence data to evolutionary diversity via HotSpotWizard's multiple sequence alignment (MSA): During the routine workflow of HotSpotWizard, the tool generates an MSA of the query sequence with 199 similar sequences (in our case fluoroacetate dehalogenases, haloacetate dehalogenases, alpha-beta hydrolases, etc). The amino acid frequencies for each aligned residue can be inspected in the web interface. Across these 200 sequences, the occurrence (%) of each amino acid at our targeted positions (S147, W180, Q245 and M246) was extracted from this dataset and the distribution was used to make a treemap chart. Gaps were not included.

Protein production and purification: Flasks containing 250 mL or 500 mL LB with 34 µg/mL chloramphenicol were inoculated with 250 µL or 500 µL, respectively, of a densely grown

overnight culture of *E. coli* BL21(DE3) cells harboring the appropriate pACYC_RPA1163 or pACYC_dehH1 (or a variant thereof) plasmid. Cells were grown at 37 °C, 135 rpm until an OD of ~0.3-0.6 was reached, and gene expression was induced by adding 1 mM IPTG. Enzymes were produced overnight (~20h) at 37 °C (with the exception of H1-H272A and H1-PL, which were produced at 18 °C), 135 rpm, after which the cells were harvested by centrifugation (3,700 rpm for 20 min, 4 °C). When needed, cell pellets were stored at -20 °C until purification. Next, cell pellets were resuspended in buffer (20 mL, 50 mM Na_2HPO_4, pH 8, containing 1 mg/mL egg white lysozyme). The cells were then lysed by sonication for 10 min, with 5 s pulse and 5 s pause cycles at 70% amplitude**,** and cellular debris was removed by centrifugation (12,000 rcf for 45 min, 4 °C). The supernatant was loaded onto a Ni-NTA resin and purified according to the manufacturer's specifications. Elution fractions containing protein were pooled, concentrated, and finally stored in 50 mM Na_2HPO_4, pH 8 with 5% glycerol at -20 °C. The purity and identity of dehH1 variants/RPA1163 was confirmed by SDS-PAGE (**Supporting Figs. S9, S14**) and mass spectrometry, respectively (Q-ToF UPLC-MS, **Supporting Table S6**).

Reaction assays for in vitro characterization of defluorination activity: To investigate enzyme activity on FA, FP and F_2A, standard enzymatic reactions were performed in 1.5 mL Eppendorf tubes at 25 ˚C without shaking with a volume of 450 µL in 50 mM Na_2HPO_4 buffer (pH 8.0) with 10 mM substrate. Reactions with wildtype H1 were performed as biological duplicates, all other reactions were performed as technical duplicates (or in a few indicated cases, technical triplicates). Substrates were prepared as 20× stocks and adjusted to pH ~8 by addition of NaOH. 20 µM stock solutions of the FAcD-H1 variants were prepared in the same buffer. To initiate the reactions, enzyme was added: the final concentration was 0.5 µM enzyme for reactions with FA, 0.5 µM or 5 µM enzyme for reactions with FP (5 µM for poorly performing enzymes) and 1 µM enzyme for reactions with F_2A. Samples of 100 µL

were taken at several time points (for F_2A after 24 hours only) and quenched by addition to 1 volume of ice cold MeCN. Next, 250 µL MilliQ was added to increase the final volume and suppress noise, and 50 µL D_2O was added for shimming. For a number of samples, instead of pure D_2O 50 µL of a freshly prepared 10× stock of $NaBF_4$ in D_2O was added as a qualitative internal standard (0.5 mM final concentration). Samples were mixed by vortexing, transferred to an NMR tube and analyzed by ^{19}F-NMR immediately. For assaying activity on 2,3,3,3-tetrafluoropropanoic acid (4F), the same procedure was performed, except with 5 µM enzyme, and quenching only after 2 and 19 hours. Only trace activity was detected on this substrate (See Supporting Document with ^{19}F-NMR spectra).

For assaying activity on F_2P, 450 µL reactions were set up in 50 mM Na_2HPO_4 buffer (pH 8.0), containing 10 mM F_2P, 45 µL D_2O, and 20 µM enzyme. Samples were mixed briefly by pipetting, transferred to an NMR tube, and incubated at 25 °C in a non-shaking incubator. Reaction progress was monitored by ^{19}F-NMR, measuring after at least 24h incubation.

Time-course with F_2A: Enzymatic reactions were performed as described before (see *Reaction assays for in vitro characterization of defluorination activity*) with some minor adjustments. Specifically, reaction volume was increased to 850 µL in order to be able to quench at more timepoints. Reactions with H1 wildtype and H1-ML were incubated at 25 °C as before, and reactions with RPA1163 were incubated at 45 °C. The enzyme concentration was 1 µM and F_2A concentration was 10 mM. Samples were quenched and analyzed as previously described.

Extended reaction assay to pinpoint enantioselectivity of H1-variants: To investigate whether our engineering had affected enantioselectivity for FP, enzymatic reactions were performed as described before (see *Reaction assays for in vitro characterization of defluorination activity*) with some minor adjustments. In brief, reactions were performed in the

same buffer and same temperature with 0.5 µM H1 (biological duplicates) or 0.5 µM H1-A (technical replicates) with 10 mM FP. Samples were taken and quenched after extended times, namely 5 hours and 24 hours. An extended ^{19}F-NMR protocol was used with a relaxation delay of 20 seconds, with 128 scans, a pulse angle of 45 degrees, and a spectral width of -20 to -240 ppm (see *^{19}F-NMR analysis*).

^{19}F-NMR analysis: Using ^{19}F-NMR, substrate (FA, F_2A, FP, or F_2P) consumption and product (F^-) formation could be monitored simultaneously, both in cellular supernatant and in reaction assays. The apparent initial rate of each enzyme was calculated based on the formation of F^- after a suitable timepoint, at which 5-10% conversion had occurred. F^- concentration was determined based on the quantitative analysis of F^- and substrate signal. The same ppm ranges were used for signal integration to prevent bias: -119.400 to -120.200 ppm for F^-; -216.100 to -217.000 ppm for FA; -123.500 to -124.800 ppm for F_2A; -172.600 to -173.400 ppm for FP; -96.500 to -98.900 ppm for F_2P. The spectra were referenced to the F^- signal at -119.800 ppm or to the internal standard $NaBF_4$ signal at -150.200 ppm if used. Auto baseline correction (Whittaker Smoother) and auto phase correction were applied when necessary. Controls containing no enzyme or an inactive enzyme variant (FAcD-H1-H272A) provided insight into background trace levels of F^- that sometimes followed from impurities in the buffer, internal standard, or substrate stocks. Corrections based on these controls were applied when necessary.

Regarding the ^{19}F-NMR protocol, a relaxation delay (d1) of 1 second was used, with 128 scans, a pulse angle of 45 degrees and a spectral width of 100 to -240 ppm, unless indicated otherwise. Because of the long relaxation time of F^-, we observed a slight (~10%) systematic overestimation of F^- with this method, and proceeded to compare of several ^{19}F-NMR protocols (see **Supporting Table S12**). We deemed our fast protocol (4 min 23 sec per sample)

sufficiently accurate when measuring conversion within the 5-10%, but employed a specialized protocol with an extended relaxation delay when the error needed to be mitigated, for example when assessing enantioselectivity of H1-variants (described earlier). In those cases, a relaxation delay of 20 seconds was used, with 128 scans, a pulse angle of 45 degrees, and a spectral width of -20 to -240 ppm (**see Supporting Table S12**).

An example of calculating apparent initial rate ($v_{0,app}$) is given. The processed ^{19}F-NMR spectrum below (**Supporting Fig. S16**) indicates the conversion of FP (10 mM) with enzyme variant VW (0.5 μM) after 30 minutes. Signals for F^- at -119.80 ppm and FP at -172.95 ppm are both integrated, and total integrals are set to 100. From these values, we find 6.02% F^- release and 93.98% FP, corresponding to 0.602 mM F^- and 9.398 mM FP. The following formula was applied to determine the apparent initial rate: $v_{0,app}$ = [F^- formed in mM] / ([enzyme concentration in mM] × time in seconds) = 0.602 mM / (0.0005 mM × 1800 seconds) = 0.7 s^{-1}. Duplicates were used to calculate an average and standard deviation for each entry. Turnover number (TON) corresponds to the number of moles of a substrate that a mole of catalyst can convert before becoming inactivated, so to determine those values, the following formula was applied: TON = (product in moles) / (catalyst in moles). The product glyoxylate relates to half of the fluoride produced as determined by ^{19}F-NMR. Duplicates (or in a few indicated entries, triplicates) were used to calculate an average TON and standard deviation for each entry. All TONs were determined based on 24-hour time samples.

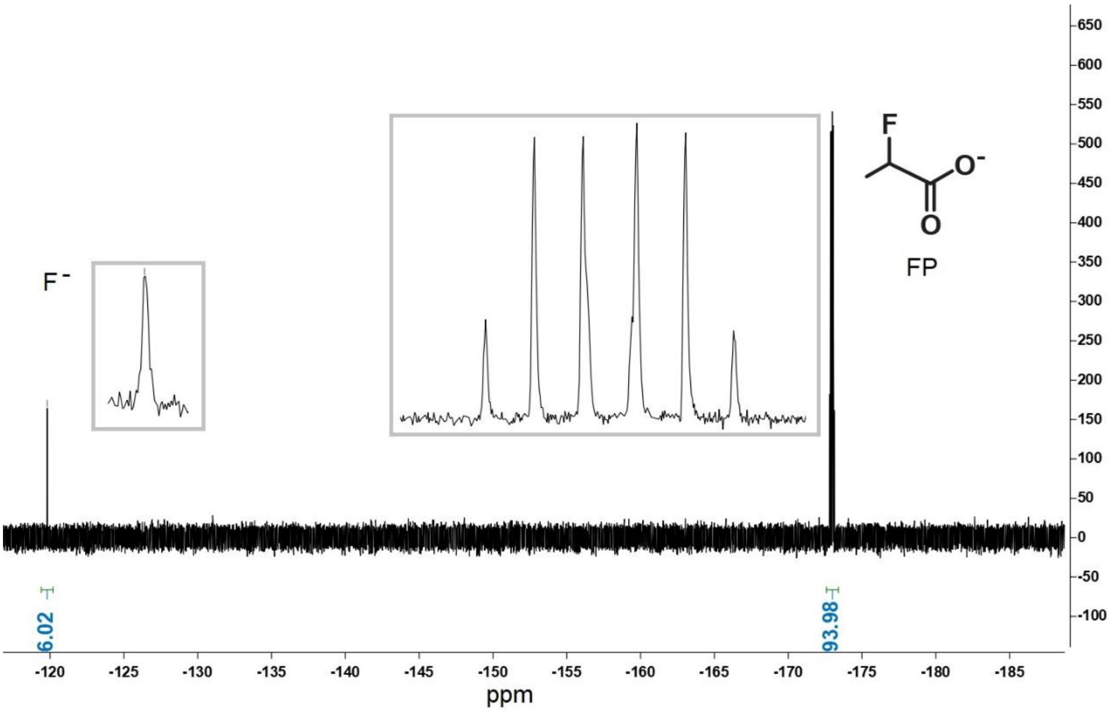

Figure S16: ^{19}F-NMR spectrum of enzyme variant H1-VW (0.5 µM) converting FP (10 mM). The reaction was quenched after 30 minutes. Close-ups of the signals are given in grey boxes; they show the expected splitting.

Enzyme modification studies upon F_2A incubation: Please refer to *Methods* for a detailed description of mass analyzer equipment. Protein samples containing 9 µM H1, H1-ML, or RPA1163 in 50 mM Na_2HPO_4 buffer (pH 8.0) were incubated with 10 mM F_2A at room temperature (25 ˚C) without shaking. Negative controls included the same proteins and buffer, but were incubated without substrate. Aliquots were taken at t = 2h and t = 24h, diluted with MilliQ to 3 µM protein, and immediately subjected to UPLC-MS analysis. In a repeat experiment, an aliquot of the t = 24h sample of H1 was denatured for 1 hour with at 37 ˚C with urea and DTT (final concentration 2M urea, 100 mM DTT), and then subjected to UPLC-MS analysis; this did not remove the modification. Aliquots of the 9 µM samples of H1 and H1-ML were also used for LC-MS/MS analysis by (chymo)trypsin digest, described below.

(Chymo)trypsin digest: Solutions with 9 µM enzyme, incubated with and without F_2A for 24 hours, were denatured with 2 M urea and reduced with 10 mM TCEP for 1 h at 37 °C, and then

alkylated with 20 mM iodoacetamide at room temperature in the dark for 45 minutes. Samples were diluted with 67 mM NH_4HCO_3 until a urea concentration of 1 M was reached. These diluted samples were digested with 1:25 (w/w) sequencing grade modified trypsin (*Promega*) at 37 °C overnight with agitation. Alternatively, they were supplemented with 10 mM $CaCl_2$ and digested with 1:20 (w/w) sequencing grade chymotrypsin (*Promega*) and incubated at 25 °C overnight. After digestion, trifluoroacetic acid (TFA) was added to reach 1% (v/v) final concentration and any precipitated detergent was removed by centrifugation. To purify the sample by solid phase extraction, the digested peptides were cleaned with C18 SPE spin tips (*Pierce*) according to the manufacturer's instructions, dried by centrifugation under vacuum, and reconstituted in 20 µL 2% MeCN and 0.1% formic acid for injection. Digested peptides were measured on an Ultimate 3000 RSLC chromatography system coupled to an Exploris 480 Mass Analyser (MS1 resolution of 120,000 and MS2 resolution of 15,000, see *Methods*). Using PEAKS Studio 12.5 software, raw files were analyzed by searching against the forward and reverse peptide sequences of the *E. coli* proteome and the target dehH1 sequences. Identified proteins were reported if the false discovery rate of the protein identifications were less than 1%. Carbamidomethylation at cysteines was set as a fixed modification; variable modifications included acetylation at N-term, deamination, and modification of Asp/His/Tyr by $C_2H_1O_3$ (+73), $C_2H_2O_3$ (+74), and $C_2H_3O_3$ (+75).

Predictions with Boltz-2: Docking studies and corresponding structure predictions of H1, H1-A, H1-G and H1-ML were performed using Boltz-2, version 2.2.1.[22, 25] Predictions were performed on the GPU partition with 40 GB memory (1 node, 1 GPU per node) of the Hábrók High-Performance computing cluster of the University of Groningen. Default Boltz-2 settings were used, unless noted otherwise. Structures were predicted using 10 recycling steps and 25 samples, and the prediction ranked with the highest confidence score was used for analysis. The

flag `--use_msa_server` was added to generate MSA's automatically via the mmseqs2 server. The input format was described in a .yaml format with the H1 protein sequence as a dimer, i.e. with protein id `[A, B]`. The H1 protein sequence started at the N-terminal His-tag and linker, minus Met. A substrate of interest was described as ligand id `C` using the SMILES for fluoroacetate anion `C(C(=O)[O-])F`, difluoroacetate anion `C(C(=O)[O-])(F)F`, or 2-fluoropropionate anion `CC(C(=O)[O-])F`. A pocket constraint was included and enforced with a potential, to specify the following active site residues: His150, Trp151, Tyr213 (fluoride pocket), Asp105 (nucleophile), and Arg106 and Arg109 (carboxylate pocket), setting the maximum distance between any ligand atom and any atom of these active site residues to 4 Å (the lowest possible setting supported in the script). Despite this, we observed the ligand in an orientation different to what is expected from mechanistic considerations: this is likely the most energetically favorable orientation (see main text).[3] Without constraints, this orientation is also found (data not shown). In the .yaml file, `properties: affinity` was included for binder [C] to predict binding affinities. Predicted affinity values and confidence scores were extracted from the corresponding .json output files. The output .cif models were visualized using Chimera 1.10.1 and overlaid using the matchmaker command. Predicted local distance difference test (pLDDT) values were extracted from the .cif files. All predictions are included as Supporting Data.

5. Sequences

Primers: Sequences of primers used in this work, with mutations or randomizations shown underlined and bold.

**Name**	**Sequence (5' → 3')**
DuetDOWN1	GATTATGCGGCCGTGTACAA
MCS1_Up	GGAGATATACCATGGGCAGC
dehH1_H272A_fw	TTACCTGGTGGA**<u>GC</u>**CTTCTTCGTGGACCAGTTTCC
dehH1_H272_rv	TCCACCAGGTAAGGATGCGTTCGTTG
dehH1_GG_for_BsaI	ACCGGTCTCCTGGTATGGACTTTCCGGGGT
dehH1_GG_rev_BsaI	GTTGGTCTCCCAAGTCACCCATTGCGTGCTAAG
dehH1_S147NNK_for	GAATCGCTTAGTCGCCGCA**<u>NNK</u>**TATTGGCATTGGTATTTC
dehH1_S147_rev	TGCGGCGACTAAGCGATTCGTGTTCATAAAC
dehH1_W180NNK_for	TTATGAAACATGCTTGTTTGGT**<u>NNK</u>**GGAGCCACTAAAGTGTCTG
dehH1_W180_rev	ACCAAACAAGCATGTTTCATAAAAAAAGTCGGGG
dehH1_Q245NNK_for	GGTATTTTATGGGTCTAAGGGC**<u>NNK</u>**ATGGGCCAGCTTTTCGATA
dehH1_Q245_rev	GCCCTTAGACCCATAAAATACCAATGTAGGAC
dehH1_Q245NNK_M246NNK_fw	GGTATTTTATGGGTCTAAGGGC**<u>NNKNNK</u>**GGCCAGCTTTTCGATA

DNA Sequence (5' →3') of dehH1 synthetic gene (CDS flanked by BsaI sites, codon optimized for *E. coli*)

```
CTAACCGGTCTCCTGGTATGGACTTTCCGGGGTTCAAGAACTCTACGGTGACAGTTGACGGCGTAGATATTGCCT
ATACAGTCTCTGGAGAGGGGCCACCTGTGTTAATGCTGCATGGTTTCCCTCAGAACCGTGCGATGTGGGCGCGTG
TGGCCCCACAACTTGCTGAGCACCACACTGTCGTTTGCGCAGATCTTCGCGGCTACGGTGACAGCGACAAGCCGA
AGTGTTTACCAGATCGCTCAAATTACTCATTTCGCACATTCGCCCACGACCAATTGTGTGTGATGCGTCACCTTG
GGTTCGAGCGTTTTCATCTTGTCGGACATGATCGCGGCGGTCGCACCGGGCATCGCATGGCCTTGGACCACCCCG
AGGCCGTACTGTCCTTGACCGTCATGGATATTGTACCGACTTATGCCATGTTTATGAACACGAATCGCTTAGTCG
CCGCATCATATTGGCATTGGTATTTCTTACAACAACCTGAGCCATTTCCAGAACACATGATTGGGCAAGACCCCG
ACTTTTTTTATGAAACATGCTTGTTTGGTTGGGGAGCCACTAAAGTGTCTGATTTTGACCAACAGATGCTTAACG
CTTACCGCGAATCGTGGCGTAATCCAGCAATGATCCACGGTAGTTGCAGCGATTATCGTGCCGCCGCAACAATCG
ACTTGGAGCATGATTCGGCTGACATTCAGCGCAAGGTTGAATGTCCTACATTGGTATTTTATGGGTCTAAGGGCC
AGATGGGCCAGCTTTTCGATATTCCTGCAGAGTGGGCCAAACGCTGCAACAATACAACGAACGCATCCTTACCTG
GTGGACACTTCTTCGTGGACCAGTTTCCAGCTGAGACTTCGGAGATCTTGCTGAAGTTCTTAGCACGCAATGGGT
GACTTGGGAGACCAACTA
```

DNA Sequence (5' →3') of RPA1163 synthetic gene (CDS flanked by BsaI sites, codon optimized for *E. coli*)

CTAACCGGTCTCCTGGTATGCCGGACCTTGCGGACCTTTTTCCGGGGTTCGGTAGTGAATGGATCAACACTAGCT
CGGGCCGCATTTTTGCCCGTGTTGGAGGGGACGGTCCCCCGTTGCTTCTTTTGCACGGCTTTCCGCAAACCCACG
TAATGTGGCACCGCGTGGCGCCCAAGTTAGCTGAGCGTTTTAAGGTGATTGTGGCCGATTTGCCCGGCTACGGAT
GGTCGGACATGCCAGAAAGCGACGAACAGCATACCCCCTACACAAAGCGCGCCATGGCAAAGCAGCTGATTGAGG
CCATGGAACAGTTAGGCCACGTACATTTCGCTTTGGCCGGGCATGACCGTGGTGCGCGCGTATCGTACCGTTTAG
CGTTGGATTCGCCAGGCCGTTTAAGTAAATTAGCAGTACTGGATATTTTACCAACATACGAATATTGGCAACGTA
TGAATCGCGCTTACGCTCTGAAGATCTATCACTGGAGTTTTTTGGCTCAGCCAGCCCCACTGCCGGAAAATCTTT
TGGGAGGGGACCCGGATTTCTATGTTAAGGCCAAGTTGGCAAGTTGGACACGCGCTGGAGACTTGTCAGCGTTTG
ACCCTCGCGCGGTCGAGCACTATCGCATTGCCTTTGCGGACCCGATGCGCCGCCACGTTATGTGTGAGGACTATC
GCGCGGGTGCGTACGCTGATTTCGAGCATGACAAGATTGACGTAGAGGCAGGTAACAAAATCCCAGTGCCAATGT
TGGCATTGTGGGGCGCCAGTGGTATCGCGCAATCGGCGGCGACGCCGTTGGACGTCTGGCGTAAGTGGGCAAGTG
ACGTTCAGGGAGCACCAATCGAGAGCGGTCATTTCCTTCCAGAGGAGGCCCCAGACCAGACGGCCGAAGCGCTTG
TGCGCTTTTTCTCGGCTGCCCCGTGACTTGGGAGACCAACTA

Protein sequences: Protein sequences of the wildtype FAcD-H1 (DehH1) and the variants further characterized in this work are given below. For clarity, deviations from the wildtype sequence are marked in bold red. The N-terminal His-tag and corresponding linker is included in the sequence and underlined. Residue numbering starts at the Met after this linker.

H1

MGSSHHHHHHGSGLVPRGSAGMDFPGFKNSTVTVDGVDIAYTVSGEGPPVLMLHGFPQNRAMWARVAPQLAEHHT
VVCADLRGYGDSDKPKCLPDRSNYSFRTFAHDQLCVMRHLGFERFHLVGHDRGGRTGHRMALDHPEAVLSLTVMD
IVPTYAMFMNTNRLVAASYWHWYFLQQPEPFPEHMIGQDPDFFYETCLFGWGATKVSDFDQQMLNAYRESWRNPA
MIHGSCSDYRAAATIDLEHDSADIQRKVECPTLVFYGSKGQMGQLFDIPAEWAKRCNNTTNASLPGGHFFVDQFP
AETSEILLKFLARNG*

H1-H272A

MGSSHHHHHHGSGLVPRGSAGMDFPGFKNSTVTVDGVDIAYTVSGEGPPVLMLHGFPQNRAMWARVAPQLAEHHT
VVCADLRGYGDSDKPKCLPDRSNYSFRTFAHDQLCVMRHLGFERFHLVGHDRGGRTGHRMALDHPEAVLSLTVMD
IVPTYAMFMNTNRLVAASYWHWYFLQQPEPFPEHMIGQDPDFFYETCLFGWGATKVSDFDQQMLNAYRESWRNPA
MIHGSCSDYRAAATIDLEHDSADIQRKVECPTLVFYGSKGQMGQLFDIPAEWAKRCNNTTNASLPGG**A**FFVDQFP
AETSEILLKFLARNG*

H1-A (Q245A)

MGSSHHHHHHGSGLVPRGSAGMDFPGFKNSTVTVDGVDIAYTVSGEGPPVLMLHGFPQNRAMWARVAPQLAEHHT
VVCADLRGYGDSDKPKCLPDRSNYSFRTFAHDQLCVMRHLGFERFHLVGHDRGGRTGHRMALDHPEAVLSLTVMD
IVPTYAMFMNTNRLVAASYWHWYFLQQPEPFPEHMIGQDPDFFYETCLFGWGATKVSDFDQQMLNAYRESWRNPA
MIHGSCSDYRAAATIDLEHDSADIQRKVECPTLVFYGSKG**A**MGQLFDIPAEWAKRCNNTTNASLPGGHFFVDQFP
AETSEILLKFLARNG*

H1-LW (S147L-Q245W)

MGSSHHHHHHGSGLVPRGSAGMDFPGFKNSTVTVDGVDIAYTVSGEGPPVLMLHGFPQNRAMWARVAPQLAEHHT
VVCADLRGYGDSDKPKCLPDRSNYSFRTFAHDQLCVMRHLGFERFHLVGHDRGGRTGHRMALDHPEAVLSLTVMD
IVPTYAMFMNTNRLVAA**L**YWHWYFLQQPEPFPEHMIGQDPDFFYETCLFGWGATKVSDFDQQMLNAYRESWRNPA

MIHGSCSDYRAAATIDLEHDSADIQRKVECPTLVFYGSKG**W**MGQLFDIPAEWAKRCNNTTNASLPGGHFFVDQFP
AETSEILLKFLARNG*

H1-V (Q245V)

MGSSHHHHHHGSGLVPRGSAGMDFPGFKNSTVTVDGVDIAYTVSGEGPPVLMLHGFPQNRAMWARVAPQLAEHHT
VVCADLRGYGDSDKPKCLPDRSNYSFRTFAHDQLCVMRHLGFERFHLVGHDRGGRTGHRMALDHPEAVLSLTVMD
IVPTYAMFMNTNRLVAASYWHWYFLQQPEPFPEHMIGQDPDFFYETCLFGWGATKVSDFDQQMLNAYRESWRNPA
MIHGSCSDYRAAATIDLEHDSADIQRKVECPTLVFYGSKG**V**MGQLFDIPAEWAKRCNNTTNASLPGGHFFVDQFP
AETSEILLKFLARNG*

H1-LV (S147L-Q245V)

MGSSHHHHHHGSGLVPRGSAGMDFPGFKNSTVTVDGVDIAYTVSGEGPPVLMLHGFPQNRAMWARVAPQLAEHHT
VVCADLRGYGDSDKPKCLPDRSNYSFRTFAHDQLCVMRHLGFERFHLVGHDRGGRTGHRMALDHPEAVLSLTVMD
IVPTYAMFMNTNRLVAA**L**YWHWYFLQQPEPFPEHMIGQDPDFFYETCLFGWGATKVSDFDQQMLNAYRESWRNPA
MIHGSCSDYRAAATIDLEHDSADIQRKVECPTLVFYGSKG**V**MGQLFDIPAEWAKRCNNTTNASLPGGHFFVDQFP
AETSEILLKFLARNG*

H1-R (Q245R)

MGSSHHHHHHGSGLVPRGSAGMDFPGFKNSTVTVDGVDIAYTVSGEGPPVLMLHGFPQNRAMWARVAPQLAEHHT
VVCADLRGYGDSDKPKCLPDRSNYSFRTFAHDQLCVMRHLGFERFHLVGHDRGGRTGHRMALDHPEAVLSLTVMD
IVPTYAMFMNTNRLVAASYWHWYFLQQPEPFPEHMIGQDPDFFYETCLFGWGATKVSDFDQQMLNAYRESWRNPA
MIHGSCSDYRAAATIDLEHDSADIQRKVECPTLVFYGSKG**R**MGQLFDIPAEWAKRCNNTTNASLPGGHFFVDQFP
AETSEILLKFLARNG*

H1-G (Q245G)

MGSSHHHHHHGSGLVPRGSAGMDFPGFKNSTVTVDGVDIAYTVSGEGPPVLMLHGFPQNRAMWARVAPQLAEHHT
VVCADLRGYGDSDKPKCLPDRSNYSFRTFAHDQLCVMRHLGFERFHLVGHDRGGRTGHRMALDHPEAVLSLTVMD
IVPTYAMFMNTNRLVAASYWHWYFLQQPEPFPEHMIGQDPDFFYETCLFGWGATKVSDFDQQMLNAYRESWRNPA
MIHGSCSDYRAAATIDLEHDSADIQRKVECPTLVFYGSKG**G**MGQLFDIPAEWAKRCNNTTNASLPGGHFFVDQFP
AETSEILLKFLARNG*

H1-VW (S147V-Q245W)

MGSSHHHHHHGSGLVPRGSAGMDFPGFKNSTVTVDGVDIAYTVSGEGPPVLMLHGFPQNRAMWARVAPQLAEHHT
VVCADLRGYGDSDKPKCLPDRSNYSFRTFAHDQLCVMRHLGFERFHLVGHDRGGRTGHRMALDHPEAVLSLTVMD
IVPTYAMFMNTNRLVAA**V**YWHWYFLQQPEPFPEHMIGQDPDFFYETCLFGWGATKVSDFDQQMLNAYRESWRNPA
MIHGSCSDYRAAATIDLEHDSADIQRKVECPTLVFYGSKG**W**MGQLFDIPAEWAKRCNNTTNASLPGGHFFVDQFP
AETSEILLKFLARNG*

H1-FI (Q245F-M246I)

MGSSHHHHHHGSGLVPRGSAGMDFPGFKNSTVTVDGVDIAYTVSGEGPPVLMLHGFPQNRAMWARVAPQLAEHHT
VVCADLRGYGDSDKPKCLPDRSNYSFRTFAHDQLCVMRHLGFERFHLVGHDRGGRTGHRMALDHPEAVLSLTVMD
IVPTYAMFMNTNRLVAASYWHWYFLQQPEPFPEHMIGQDPDFFYETCLFGWGATKVSDFDQQMLNAYRESWRNPA
MIHGSCSDYRAAATIDLEHDSADIQRKVECPTLVFYGSKG**FI**GQLFDIPAEWAKRCNNTTNASLPGGHFFVDQFP
AETSEILLKFLARNG*

H1-ML (Q245M-M246L)

MGSSHHHHHHGSGLVPRGSAGMDFPGFKNSTVTVDGVDIAYTVSGEGPPVLMLHGFPQNRAMWARVAPQLAEHHT
VVCADLRGYGDSDKPKCLPDRSNYSFRTFAHDQLCVMRHLGFERFHLVGHDRGGRTGHRMALDHPEAVLSLTVMD
IVPTYAMFMNTNRLVAASYWHWYFLQQPEPFPEHMIGQDPDFFYETCLFGWGATKVSDFDQQMLNAYRESWRNPA
MIHGSCSDYRAAATIDLEHDSADIQRKVECPTLVFYGSKG**ML**GQLFDIPAEWAKRCNNTTNASLPGGHFFVDQFP
AETSEILLKFLARNG*

H1-RPL (A46V-S147R-Q245P-M246L)

MGSSHHHHHHGSGLVPRGSAGMDFPGFKNSTVTVDGVDIAYTVSGEGPPVLMLHGFPQNRAMWARV**V**PQLAEHHT
VVCADLRGYGDSDKPKCLPDRSNYSFRTFAHDQLCVMRHLGFERFHLVGHDRGGRTGHRMALDHPEAVLSLTVMD
IVPTYAMFMNTNRLVAA**R**YWHWYFLQQPEPFPEHMIGQDPDFFYETCLFGWGATKVSDFDQQMLNAYRESWRNPA
MIHGSCSDYRAAATIDLEHDSADIQRKVECPTLVFYGSKG**PL**GQLFDIPAEWAKRCNNTTNASLPGGHFFVDQFP
AETSEILLKFLARNG*

H1-RL (S147R-Q245L)

MGSSHHHHHHGSGLVPRGSAGMDFPGFKNSTVTVDGVDIAYTVSGEGPPVLMLHGFPQNRAMWARVAPQLAEHHT
VVCADLRGYGDSDKPKCLPDRSNYSFRTFAHDQLCVMRHLGFERFHLVGHDRGGRTGHRMALDHPEAVLSLTVMD
IVPTYAMFMNTNRLVAA**R**YWHWYFLQQPEPFPEHMIGQDPDFFYETCLFGWGATKVSDFDQQMLNAYRESWRNPA
MIHGSCSDYRAAATIDLEHDSADIQRKVECPTLVFYGSKG**L**MGQLFDIPAEWAKRCNNTTNASLPGGHFFVDQFP
AETSEILLKFLARNG*

H1-RHL (S147R-Q245H-M246L)

MGSSHHHHHHGSGLVPRGSAGMDFPGFKNSTVTVDGVDIAYTVSGEGPPVLMLHGFPQNRAMWARVAPQLAEHHT
VVCADLRGYGDSDKPKCLPDRSNYSFRTFAHDQLCVMRHLGFERFHLVGHDRGGRTGHRMALDHPEAVLSLTVMD
IVPTYAMFMNTNRLVAA**R**YWHWYFLQQPEPFPEHMIGQDPDFFYETCLFGWGATKVSDFDQQMLNAYRESWRNPA
MIHGSCSDYRAAATIDLEHDSADIQRKVECPTLVFYGSKG**HL**GQLFDIPAEWAKRCNNTTNASLPGGHFFVDQFP
AETSEILLKFLARNG*

H1-PL (S147P-Q245L)

MGSSHHHHHHGSGLVPRGSAGMDFPGFKNSTVTVDGVDIAYTVSGEGPPVLMLHGFPQNRAMWARVAPQLAEHHT
VVCADLRGYGDSDKPKCLPDRSNYSFRTFAHDQLCVMRHLGFERFHLVGHDRGGRTGHRMALDHPEAVLSLTVMD
IVPTYAMFMNTNRLVAA**P**YWHWYFLQQPEPFPEHMIGQDPDFFYETCLFGWGATKVSDFDQQMLNAYRESWRNPA
MIHGSCSDYRAAATIDLEHDSADIQRKVECPTLVFYGSKG**L**MGQLFDIPAEWAKRCNNTTNASLPGGHFFVDQFP
AETSEILLKFLARNG*

6. Supporting References

[1] K. Jitsumori, R. Omi, T. Kurihara, A. Kurata, H. Mihara, I. Miyahara, K. Hirotsu, N. Esaki, *J Bacteriol* **2009**, *191*, 2630-2637.

[2] W. Y. Chan, M. Wong, J. Guthrie, A. V. Savchenko, A. F. Yakunin, E. F. Pai, E. A. Edwards, *Microbial Biotechnology* **2010**, *3*, 107-120.

[3] P. W. Y. Chan, A. F. Yakunin, E. A. Edwards, E. F. Pai, *Journal of the American Chemical Society* **2011**, *133*, 7461-7468.

[4] C. K. Davis, S. E. Denman, L. I. Sly, C. S. McSweeney, *Lett Appl Microbiol* **2011**, *53*, 417-423.

[5] Y. Li, Y. Yue, H. Zhang, Z. Yang, H. Wang, S. Tian, J. B. Wang, Q. Zhang, W. Wang, *Environ Int* **2019**, *131*, 104999.

[6] H. Zhang, S. Tian, Y. Yue, M. Li, W. Tong, G. Xu, B. Chen, M. Ma, Y. Li, J.-b. Wang, *ACS Catalysis* **2020**, *10*, 3143-3151.

[7] Y. Yue, J. Fan, G. Xin, Q. Huang, J. B. Wang, Y. Li, Q. Zhang, W. Wang, *Environ Sci Technol* **2021**, *55*, 9817-9825.

[8] M. D. Bygd, K. G. Aukema, J. E. Richman, L. P. Wackett, *Appl Environ Microbiol* **2022**, *88*, e0028822.

[9] A. N. Khusnutdinova, K. A. Batyrova, G. Brown, T. Fedorchuk, Y. S. Chai, T. Skarina, R. Flick, A. P. Petit, A. Savchenko, P. Stogios, A. F. Yakunin, *The FEBS Journal* **2023**, *290*, 4966-4983.

[10] C. Husser, S. Vuilleumier, M. Ryckelynck, *Small* **2023**, *19*, 2205232.

[11] S. Farajollahi, N. V. Lombardo, M. D. Crenshaw, H. B. Guo, M. E. Doherty, T. R. Davison, J. J. Steel, E. A. Almand, V. A. Varaljay, C. Suei-Hung, P. A. Mirau, R. J. Berry, N. Kelley-Loughnane, P. B. Dennis, *ACS Omega* **2024**, *9*, 28546-28555.

[12] S. I. Probst, F. D. Felder, V. Poltorak, R. Mewalal, I. K. Blaby, S. L. Robinson, *Proceedings of the National Academy of Sciences* **2025**, *122*, e2504122122.

[13] A. T. Simon, A. G. Dodge, J. Bondy, M. R. O'Connor, A. Aksan, L. P. Wackett, *mBio* **2025**, *16*.

[14] K. F. Geoghegan, H. B. F. Dixon, P. J. Rosner, L. R. Hoth, A. J. Lanzetti, K. A. Borzilleri, E. S. Marr, L. H. Pezzullo, L. B. Martin, P. K. LeMotte, A. S. McColl, A. V. Kamath, J. G. Stroh, *Analytical Biochemistry* **1999**, *267*, 169-184.

[15] M. J. Veen, F. S. Aalbers, H. J. Rozeboom, A. M. W. H. Thunnissen, D. F. Sauer, G. Roelfes, *Angewandte Chemie International Edition* **2025**, *64*.

[16] E. M. Gabor, E. J. De Vries, D. B. Janssen, *Environmental Microbiology* **2004**, *6*, 948-958.

[17] Z. Zhang, A. G. Marshall, *Journal of the American Society for Mass Spectrometry* **1998**, *9*, 225-233.

[18] aH. Im, *Bio-protocol* **2011**, *1*, e143; bH. Inoue, H. Nojima, H. Okayama, *Gene* **1990**, *96*, 23-28.

[19] D. J. Warren, *Anal Biochem* **2011**, *413*, 206-207.

[20] R. Rubini, S. C. Jansen, H. Beekhuis, H. J. Rozeboom, C. Mayer, *Angew Chem Int Ed Engl* **2023**, *62*, e202213942.

[21] aJ. Jumper, R. Evans, A. Pritzel, T. Green, M. Figurnov, O. Ronneberger, K. Tunyasuvunakool, R. Bates, A. Žídek, A. Potapenko, A. Bridgland, C. Meyer, S. A. A. Kohl, A. J. Ballard, A. Cowie, B. Romera-Paredes, S. Nikolov, R. Jain, J. Adler, T. Back, S. Petersen, D. Reiman, E. Clancy, M. Zielinski, M. Steinegger, M. Pacholska, T. Berghammer, S. Bodenstein, D. Silver, O. Vinyals, A. W. Senior, K. Kavukcuoglu, P. Kohli, D. Hassabis, *Nature* **2021**, *596*, 583-589; bR. Evans, M. O'Neill, A. Pritzel, N. Antropova, A. Senior, T. Green, A. Žídek, R. Bates, S. Blackwell, J. Yim, O. Ronneberger, S. Bodenstein, M. Zielinski, A. Bridgland, A. Potapenko, A. Cowie, K. Tunyasuvunakool, R. Jain, E. Clancy, P. Kohli, J. Jumper, D. Hassabis, *bioRxiv* **2022**, 2021.2010.2004.463034.

[22] M. Mirdita, K. Schütze, Y. Moriwaki, L. Heo, S. Ovchinnikov, M. Steinegger, *Nature Methods* **2022**, *19*, 679-682.
[23] L. Sumbalova, J. Stourac, T. Martinek, D. Bednar, J. Damborsky, *Nucleic Acids Res* **2018**, *46*, W356-w362.
[24] M. K. Oh, L. Rohlin, K. C. Kao, J. C. Liao, *J Biol Chem* **2002**, *277*, 13175-13183.
[25] aJ. Wohlwend, G. Corso, S. Passaro, N. Getz, M. Reveiz, K. Leidal, W. Swiderski, L. Atkinson, T. Portnoi, I. Chinn, J. Silterra, T. Jaakkola, R. Barzilay, Cold Spring Harbor Laboratory, **2024**; bS. Passaro, G. Corso, J. Wohlwend, M. Reveiz, S. Thaler, V. R. Somnath, N. Getz, T. Portnoi, J. Roy, H. Stark, D. Kwabi-Addo, D. Beaini, T. Jaakkola, R. Barzilay, Cold Spring Harbor Laboratory, **2025**.
