# Supplementary material for: Engineering Fluoroacetate Dehalogenase by Growth‐Based Selections on Non‐Natural Organofluorides
Source: Angew Chem Int Ed Engl. 2026 Jan 28;65(10):e24234. doi: 10.1002/anie.202524234 (PMC12955510; doi:10.1002/anie.202524234)

P39_E1_WT1_100mMFA_20240924120145
STANDARD FLUORINE PARAMETERS

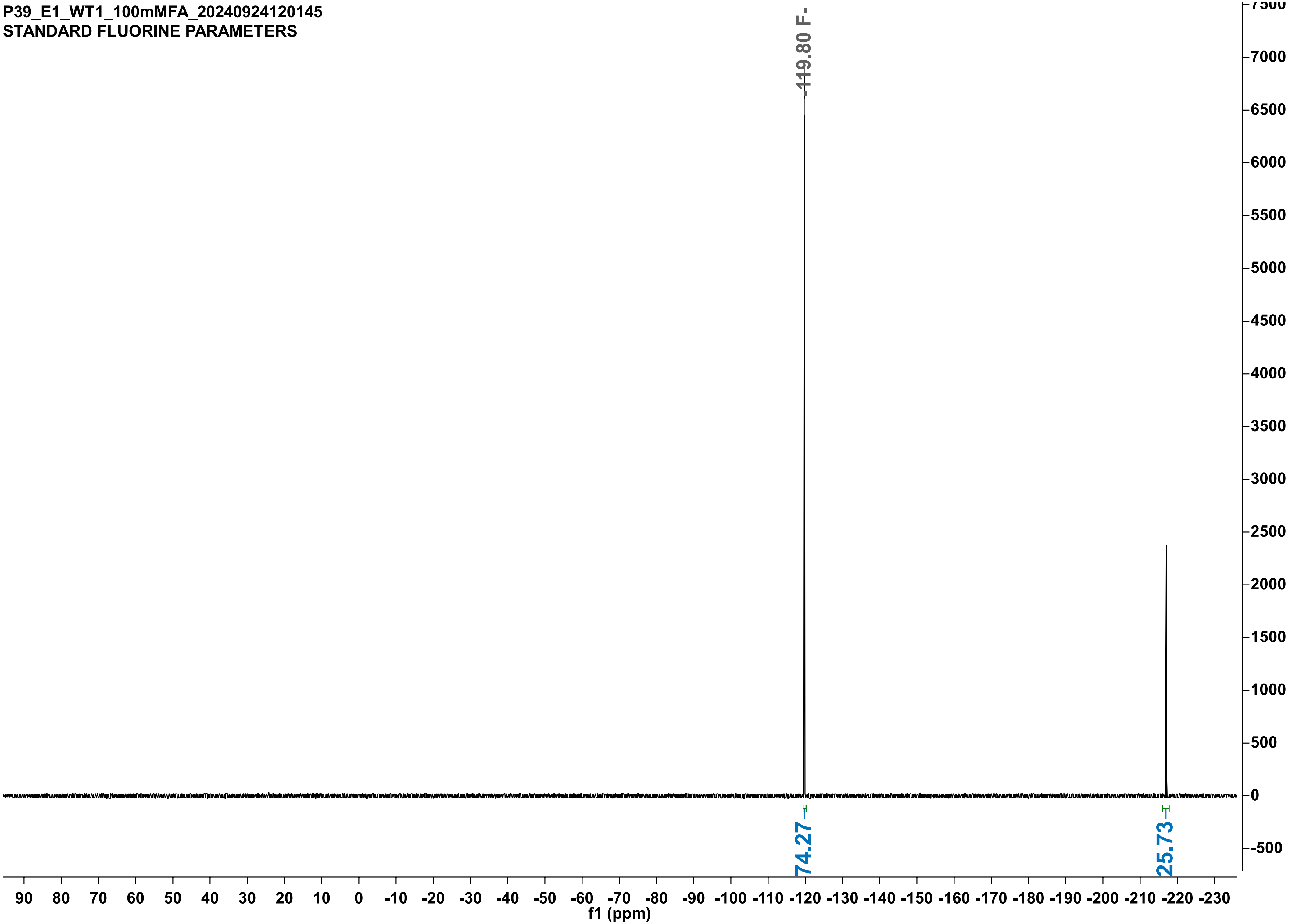

P39_E4_H272A_100mMFA_20240924120239
STANDARD FLUORINE PARAMETERS

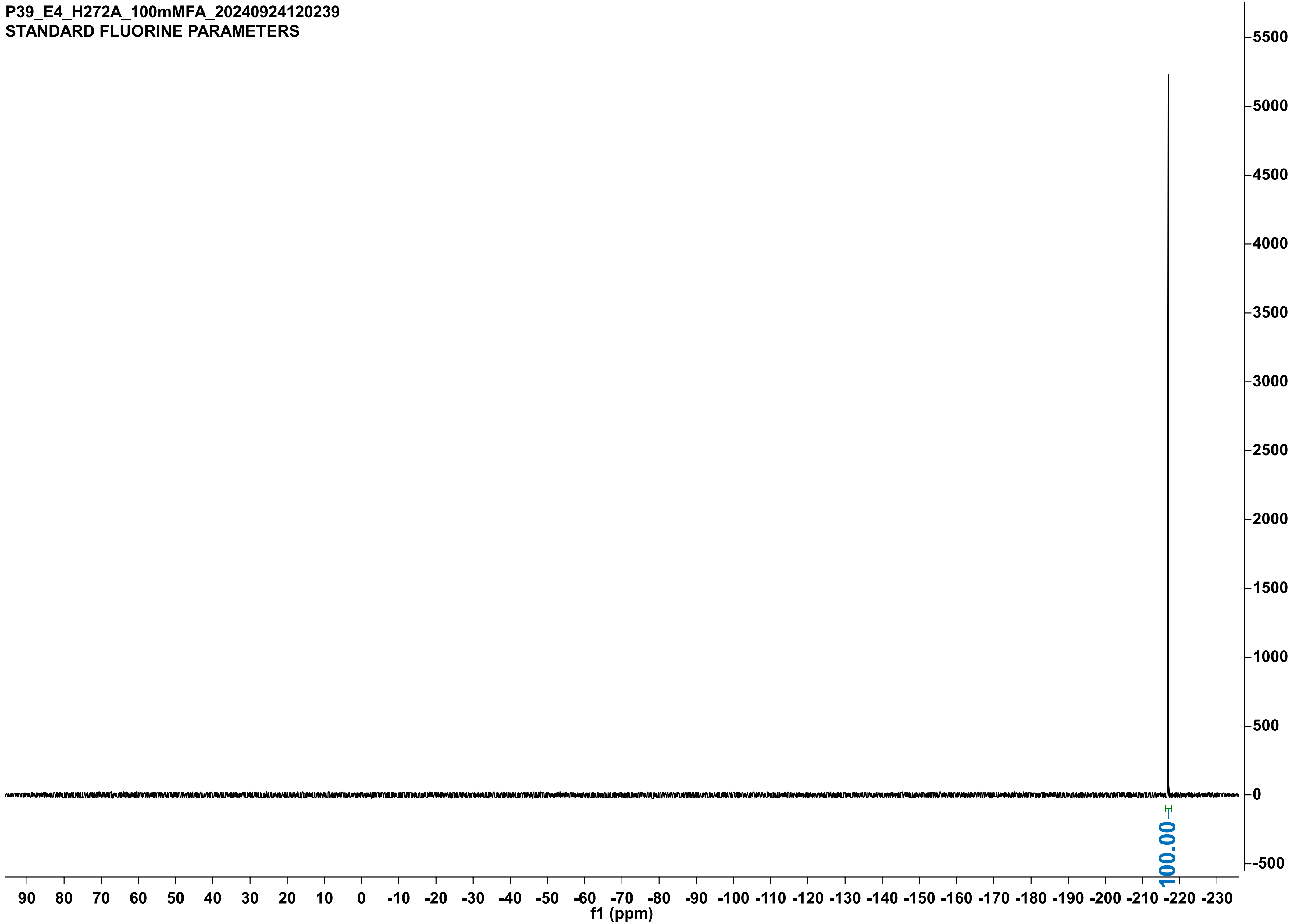

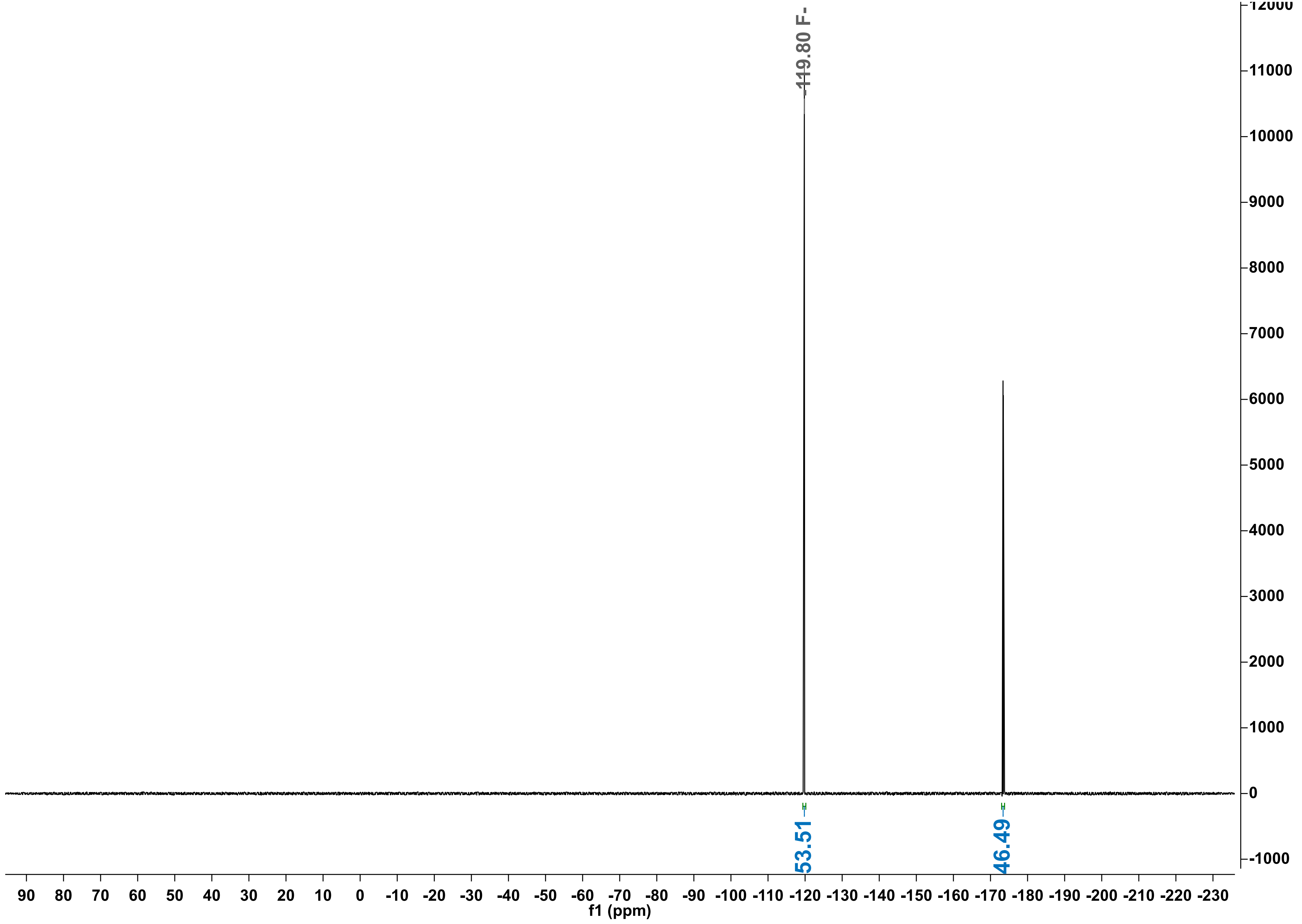

-119.80 F-
53.51
46.49
f1 (ppm)

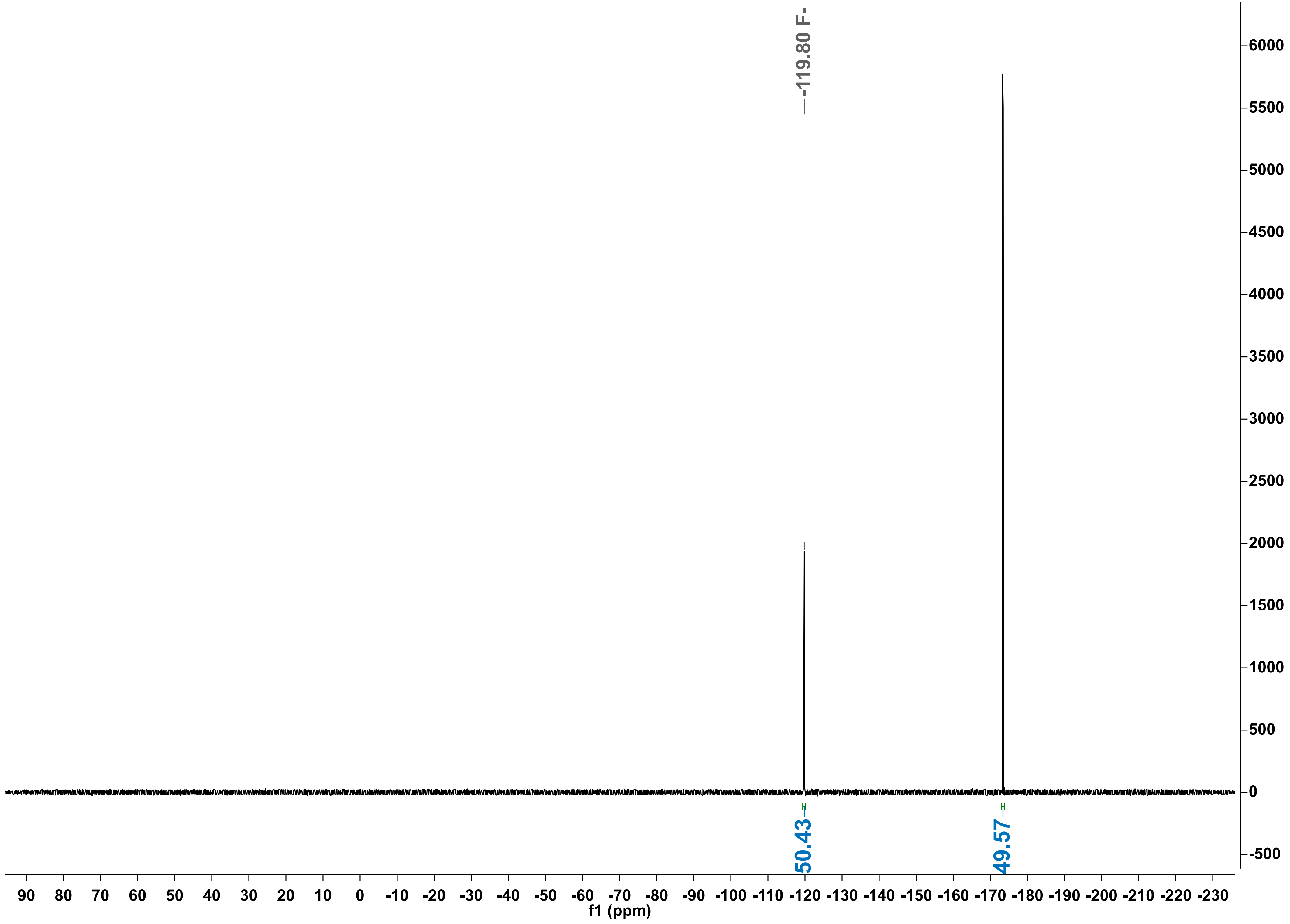

-119.80 F-
50.43
49.57
f1 (ppm)

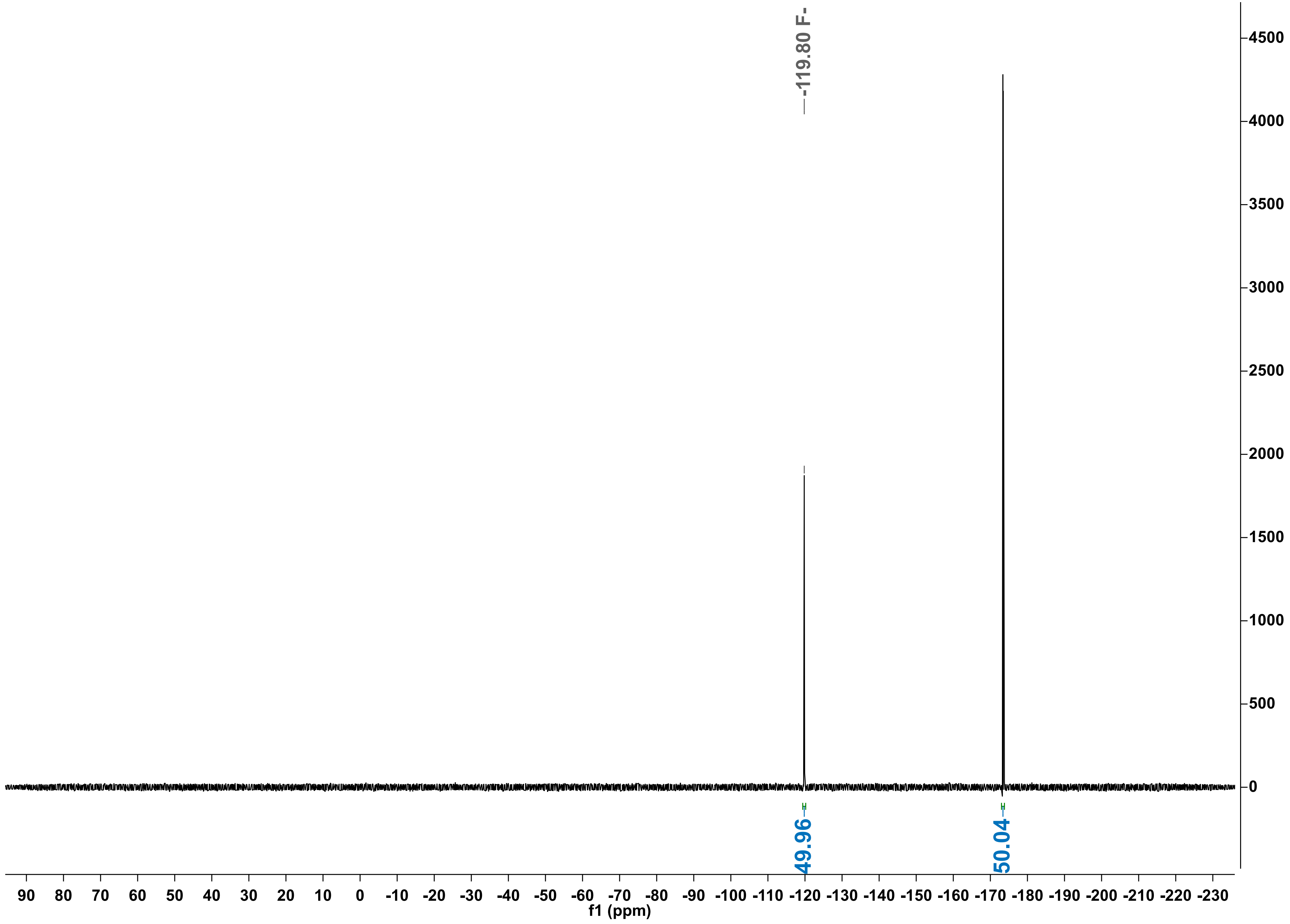

-119.80 F-
49.96
50.04
4500
4000
3500
3000
2500
2000
1500
1000
500
0
90 80 70 60 50 40 30 20 10 0 -10 -20 -30 -40 -50 -60 -70 -80 -90 -100 -110 -120 -130 -140 -150 -160 -170 -180 -190 -200 -210 -220 -230
f1 (ppm)

P50_sample19_null1_FPA_72h_20250511164508
STANDARD FLUORINE PARAMETERS

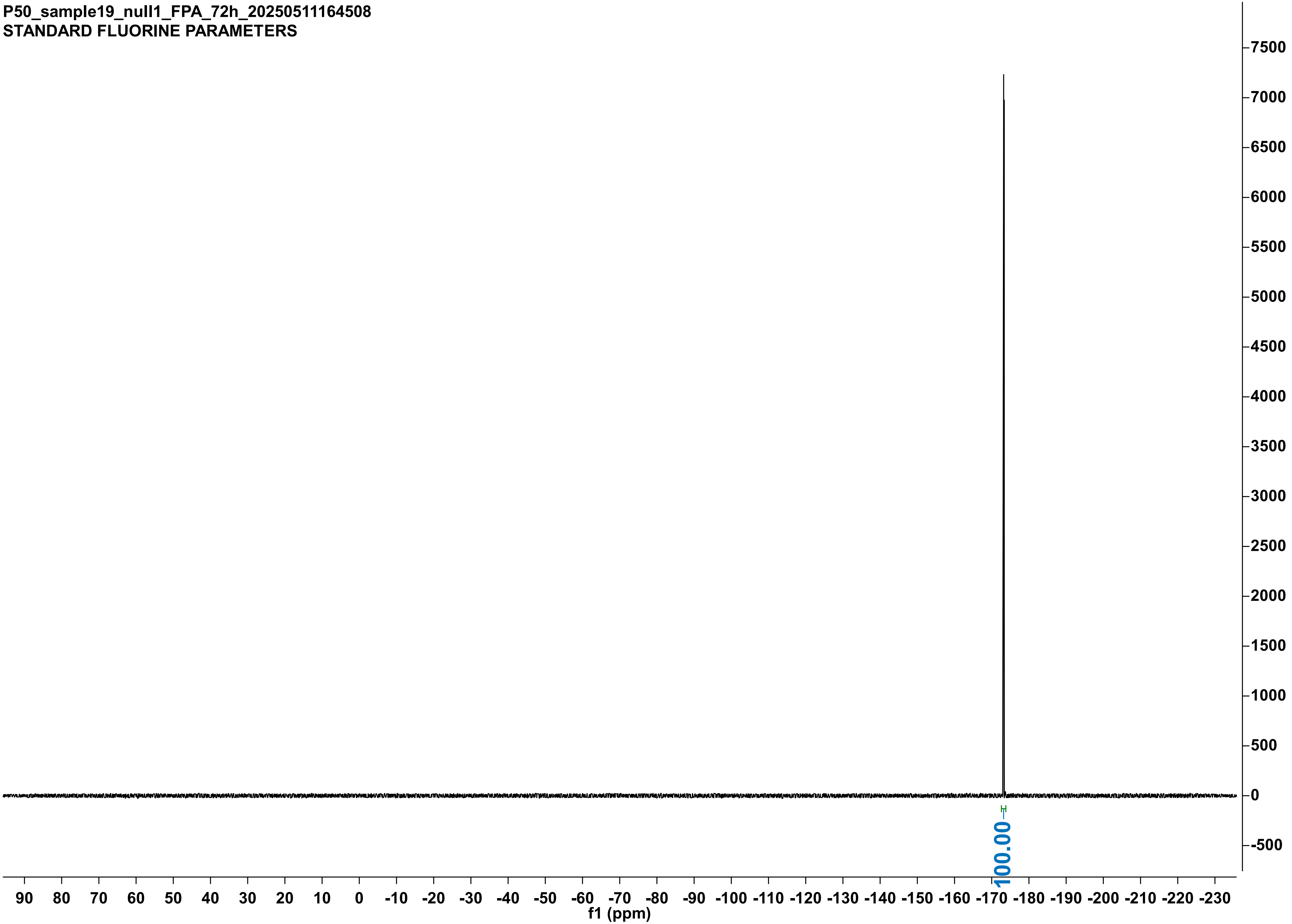

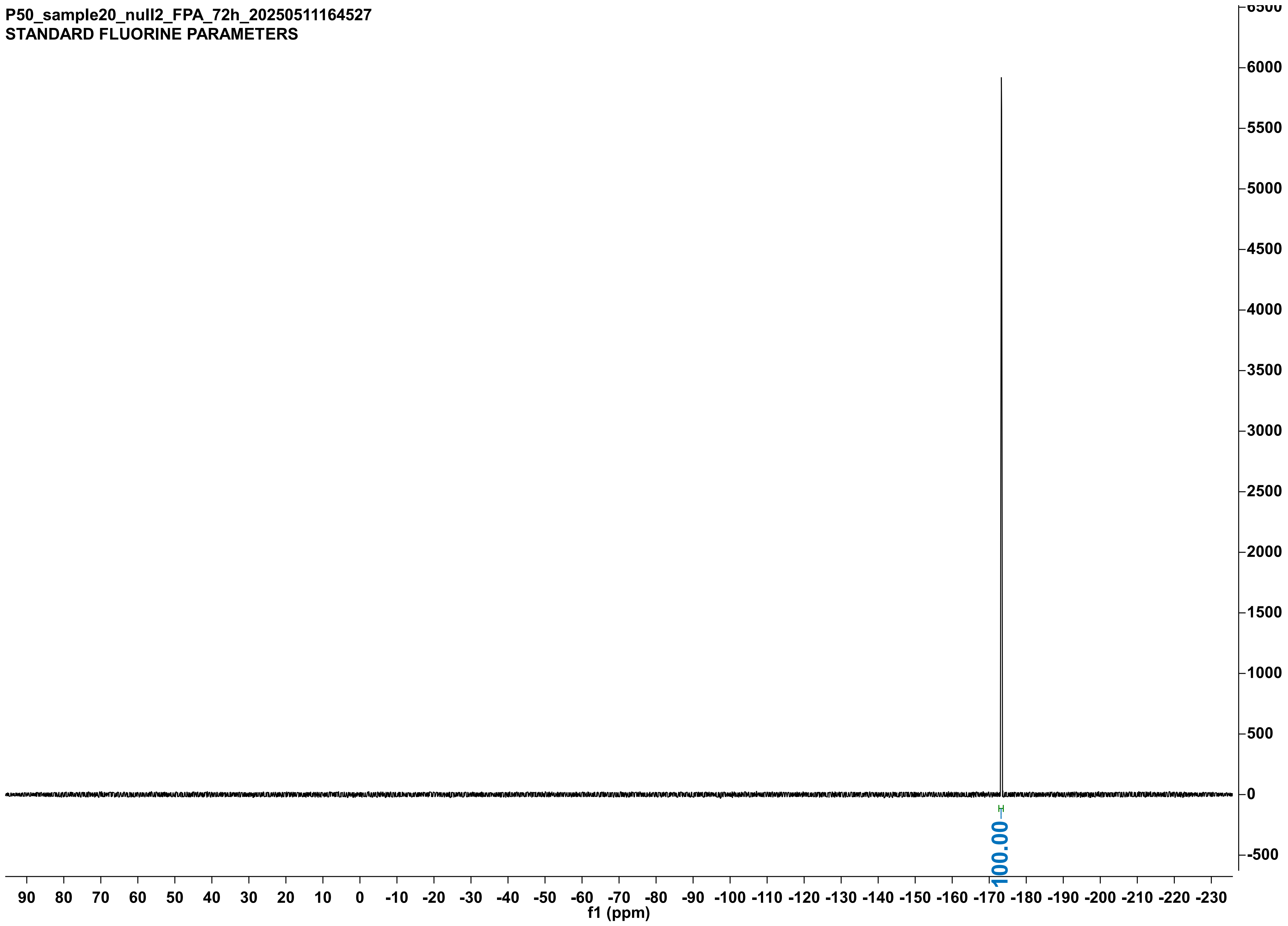

P50_sample20_null2_FPA_72h_20250511164527
STANDARD FLUORINE PARAMETERS
100.00
f1 (ppm)

P50_sample21_null3_FPA_72h_20250511164540
STANDARD FLUORINE PARAMETERS

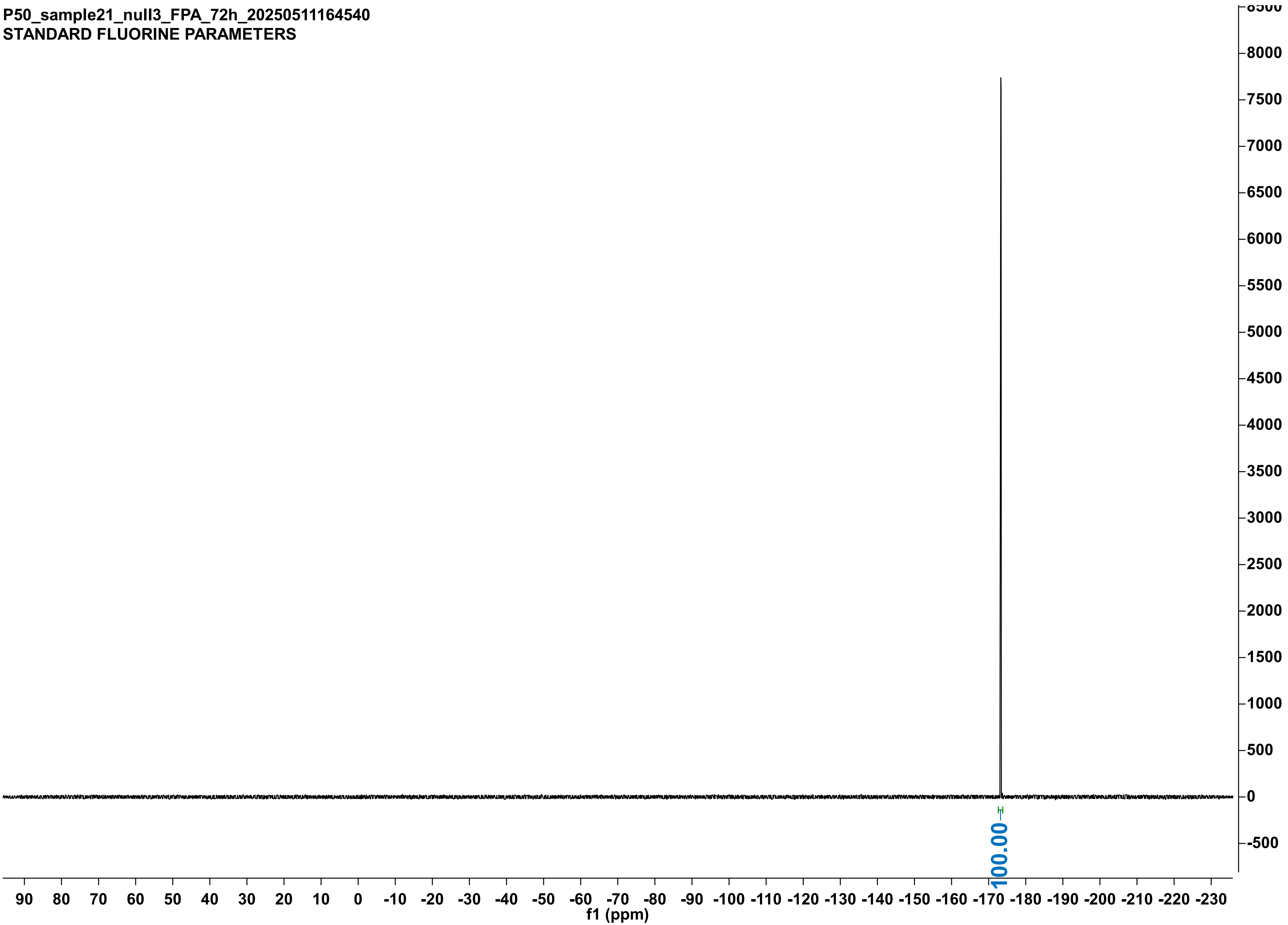

P50_sample10_WT1_2FAc_72h_20250511160442
STANDARD FLUORINE PARAMETERS

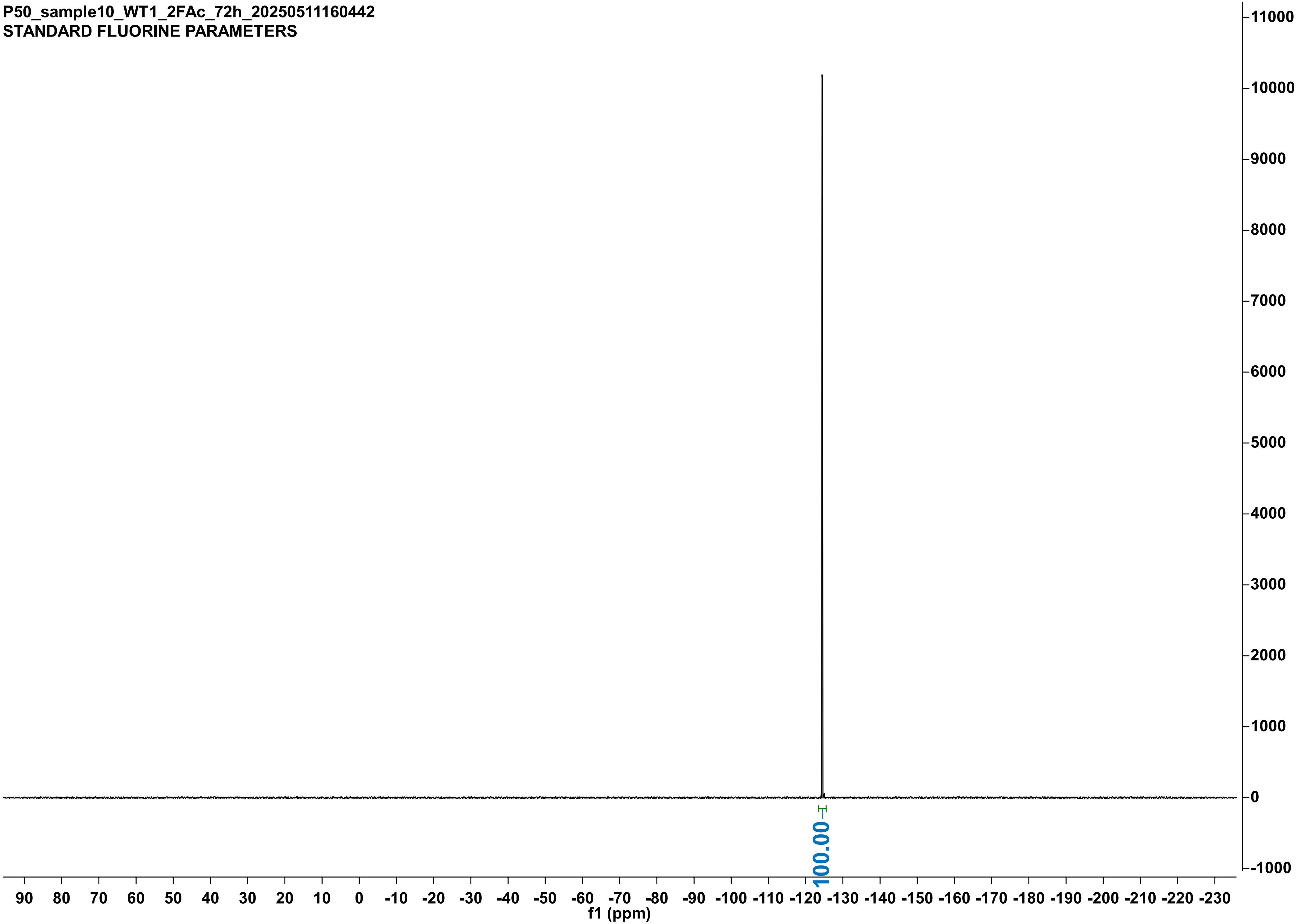

P50_sample11_WT2_2FAc_72h_20250511160453
STANDARD FLUORINE PARAMETERS

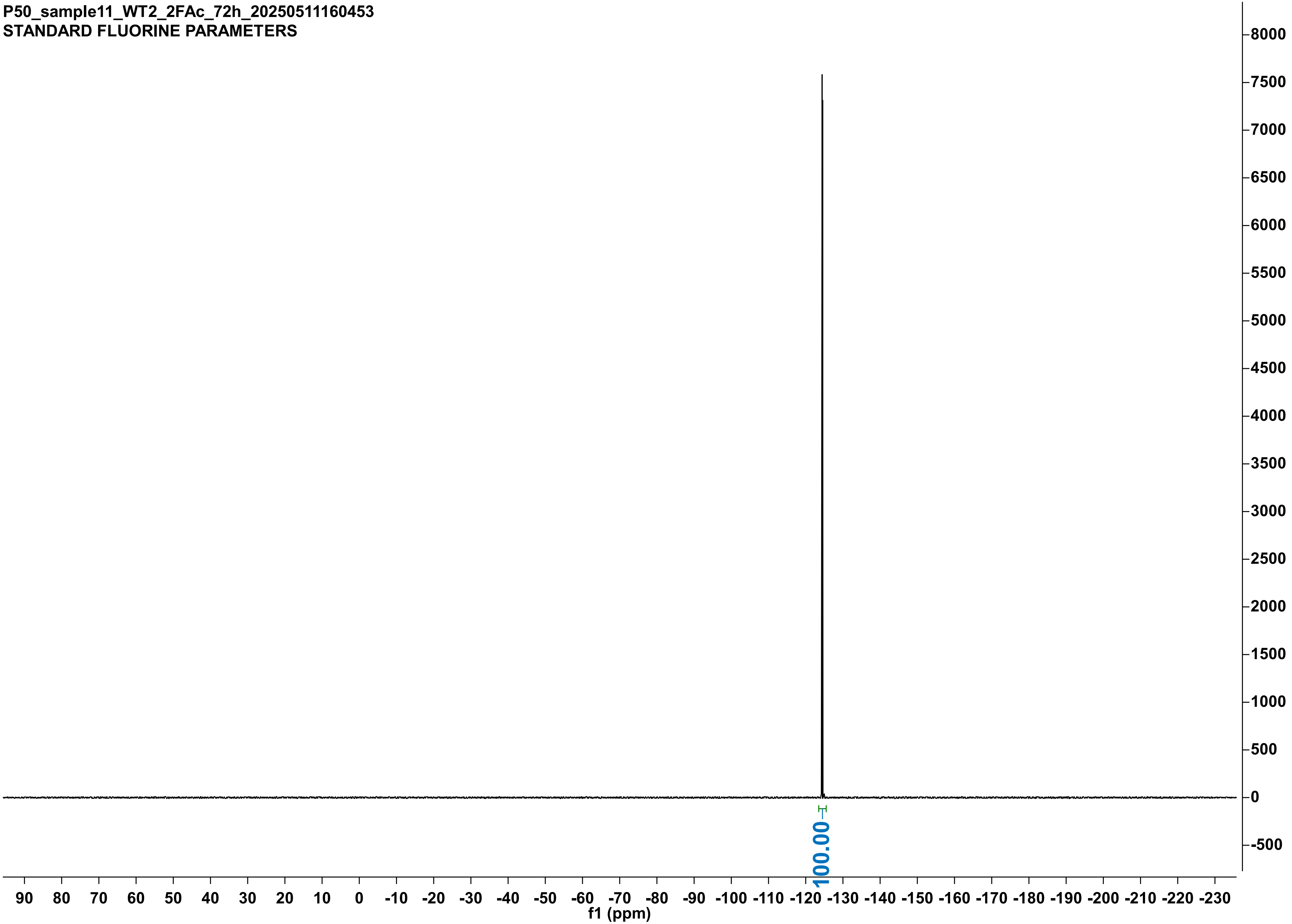

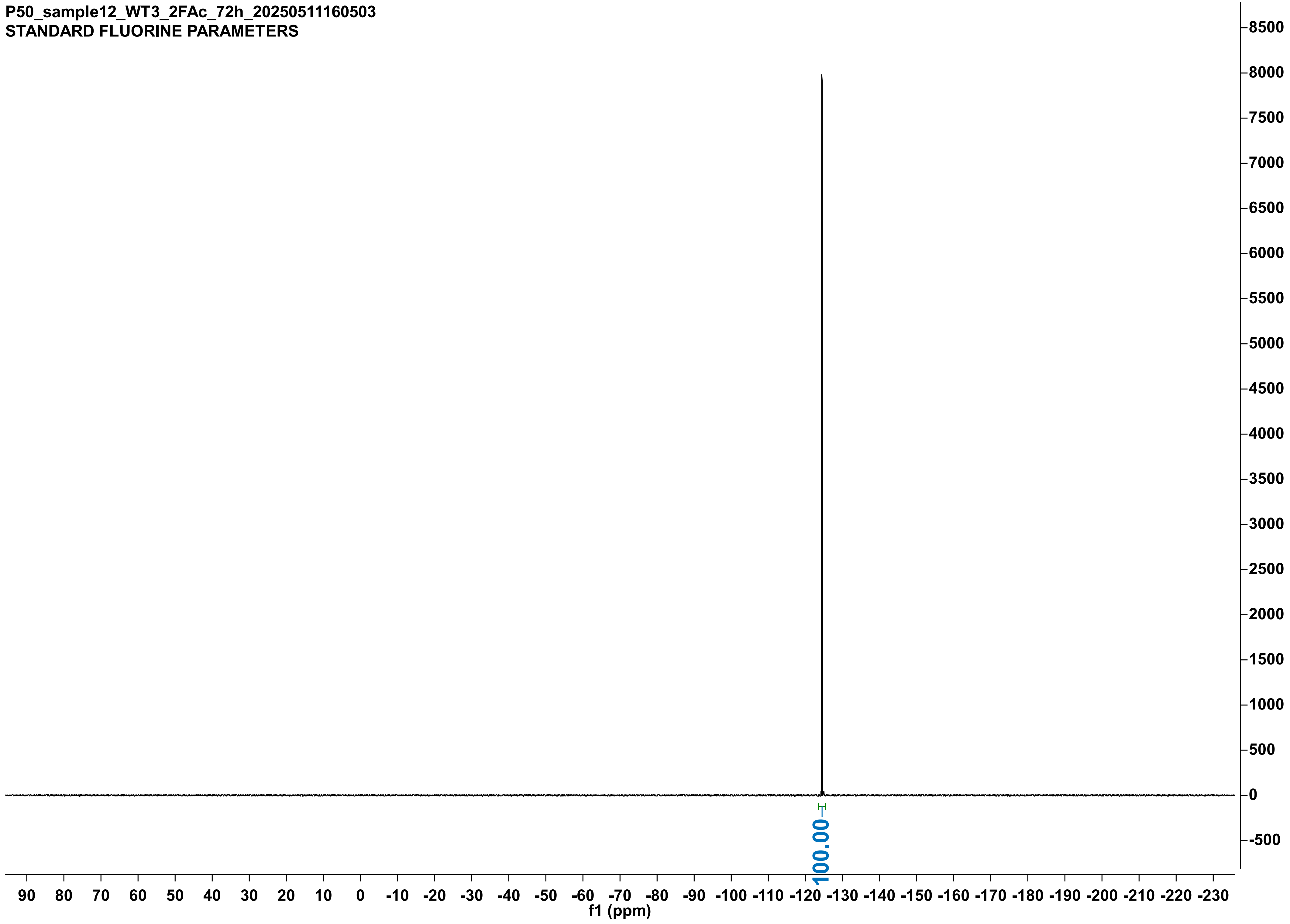
P50_sample12_WT3_2FAc_72h_20250511160503
STANDARD FLUORINE PARAMETERS
100.00
f1 (ppm)

P50_sample7_null1_2FAc_72h_20250511160407
STANDARD FLUORINE PARAMETERS

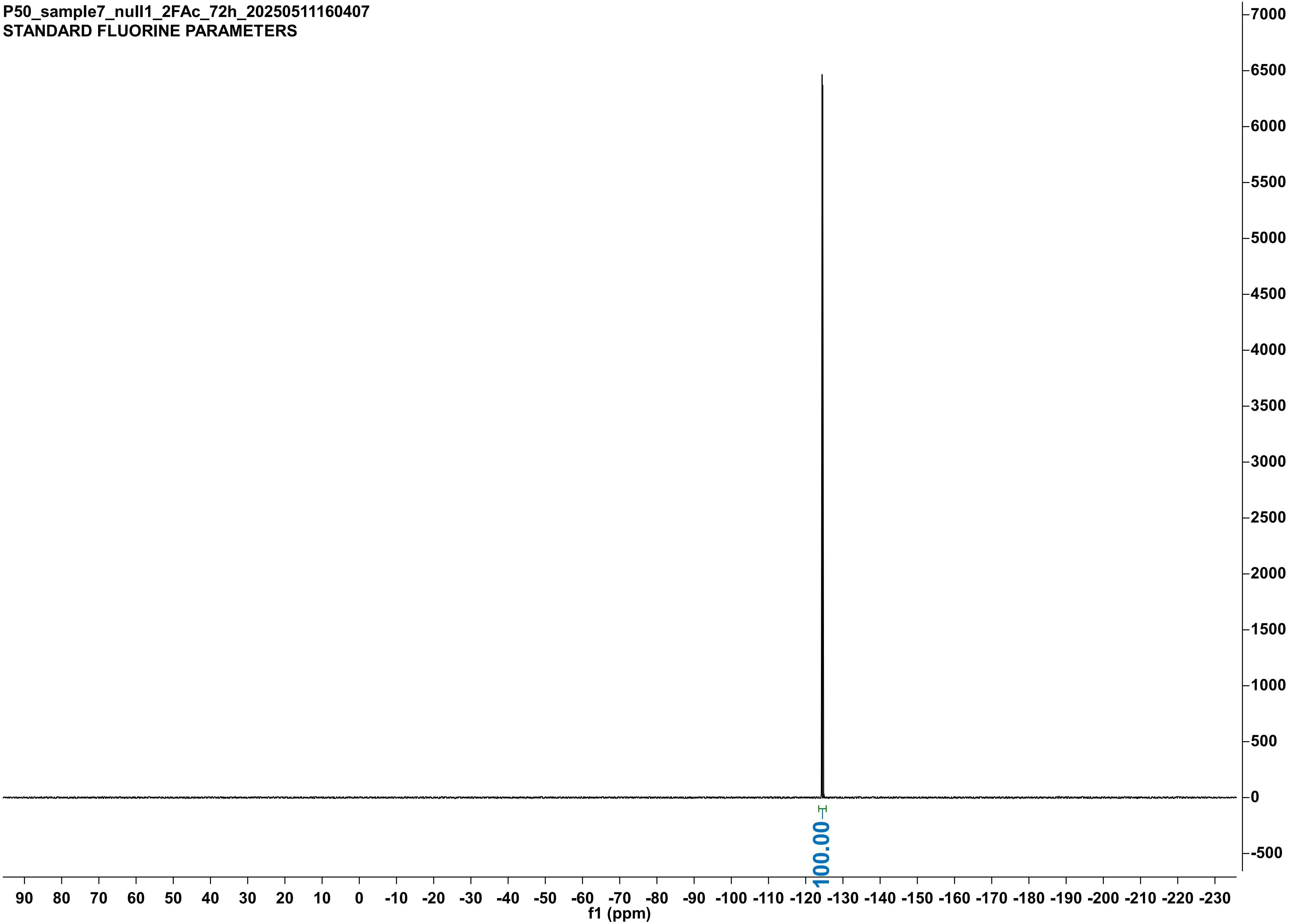

P50_sample8_null2_2FAc_72h_20250511160422
STANDARD FLUORINE PARAMETERS

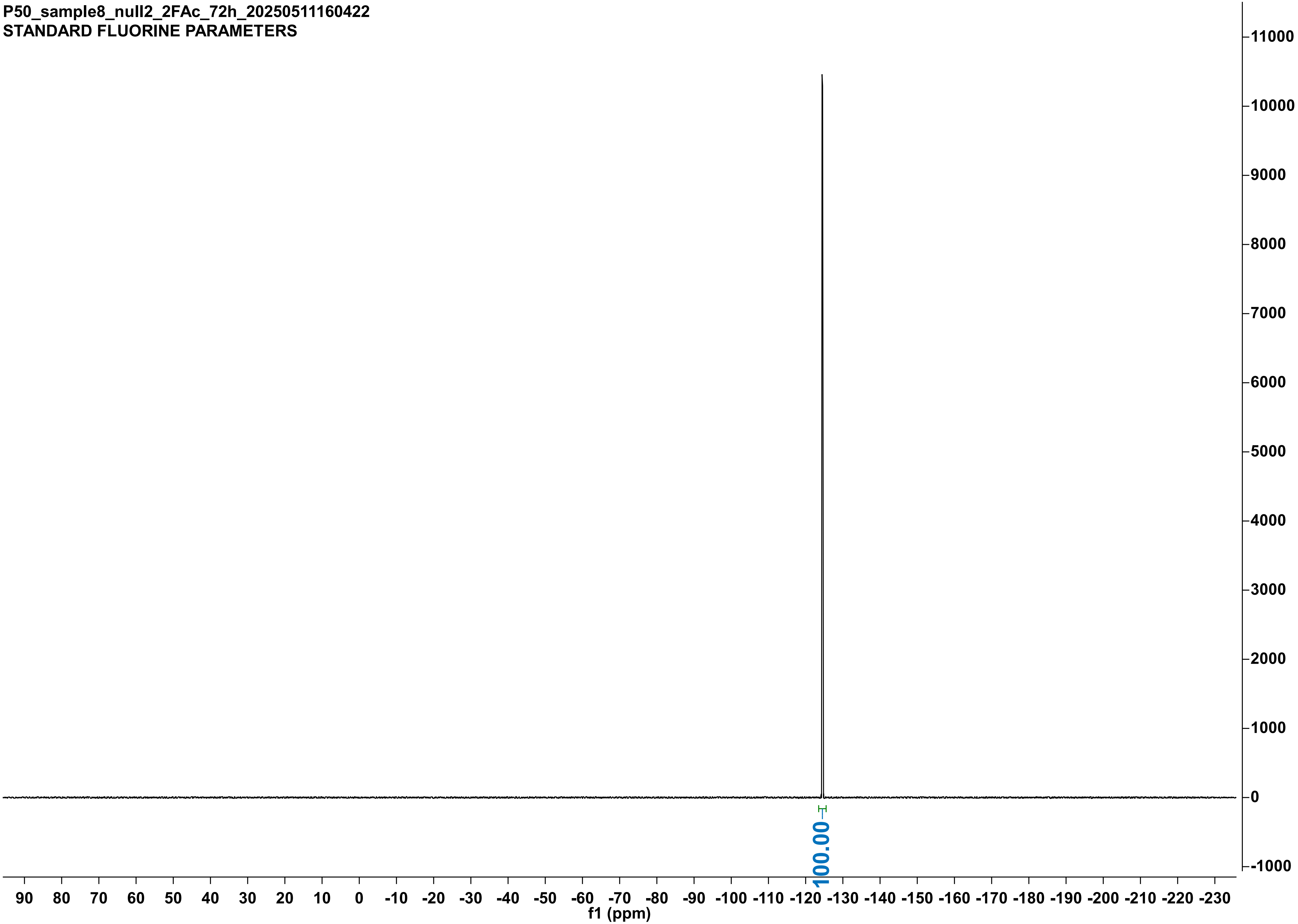

P50_sample9_null3_2FAc_72h_20250511160434
STANDARD FLUORINE PARAMETERS

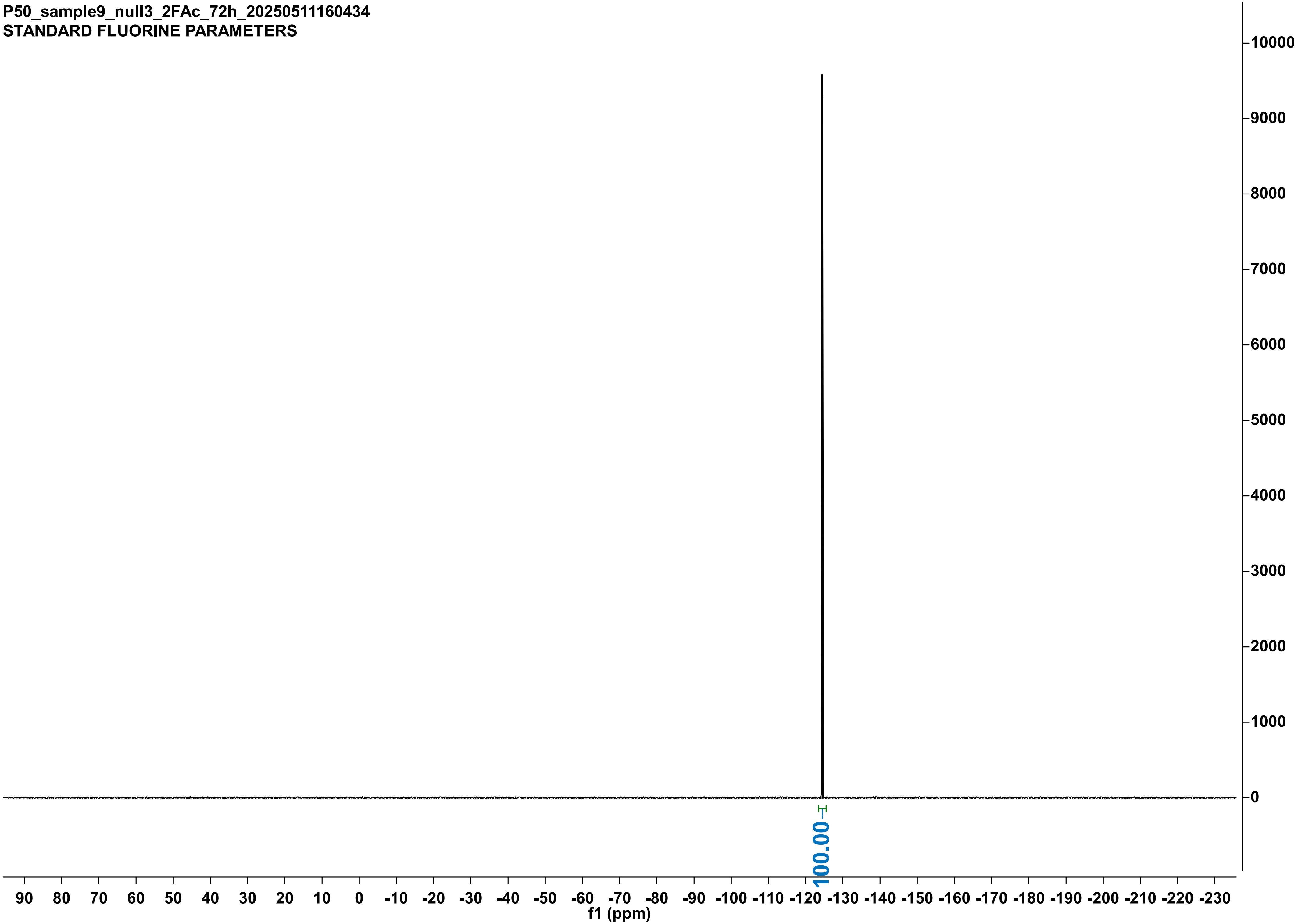

P50_sample16_WT1_2FPA_72h_20250511160616
STANDARD FLUORINE PARAMETERS

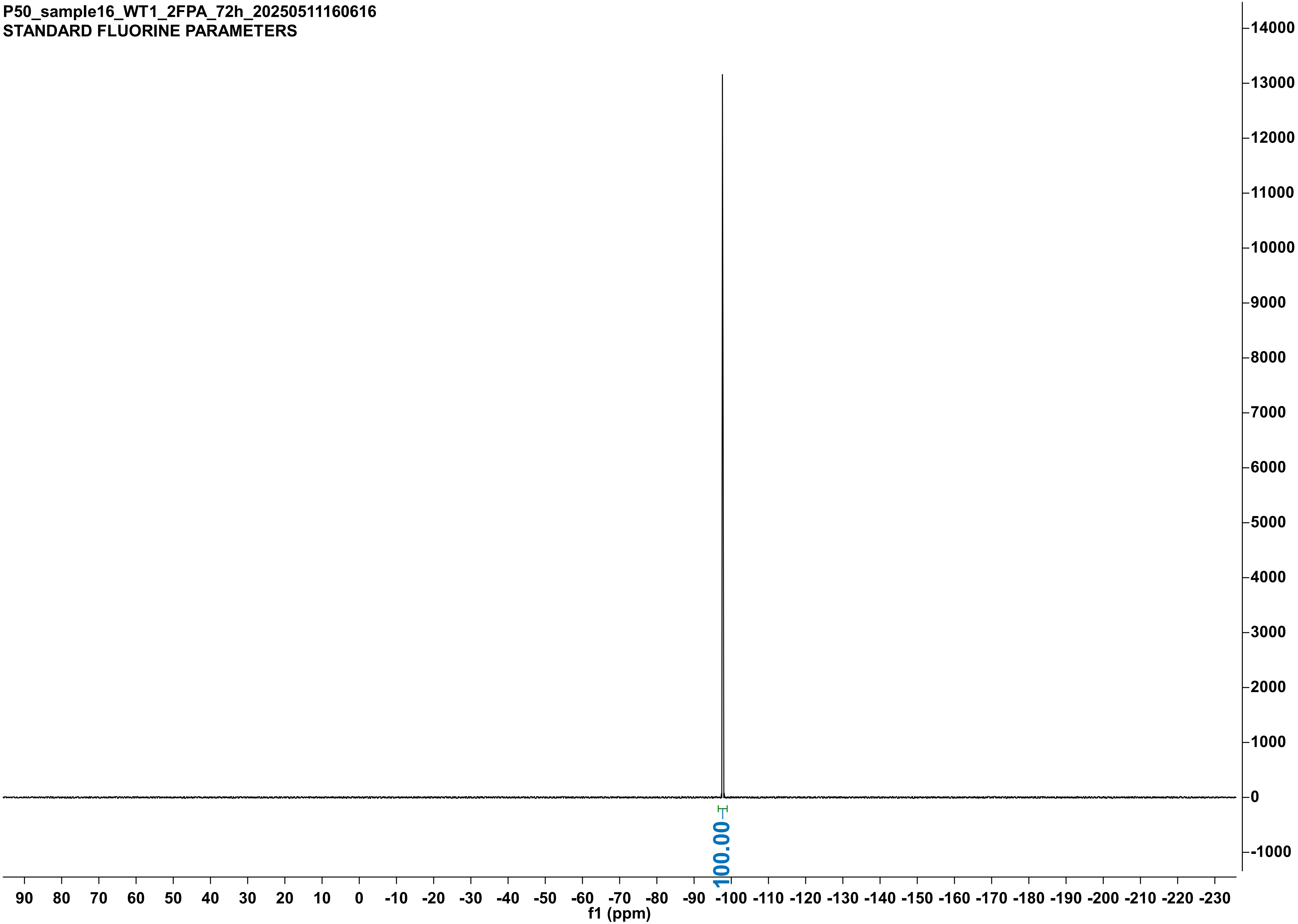

P50_sample17_WT2_2FPA_72h_20250511164442
STANDARD FLUORINE PARAMETERS

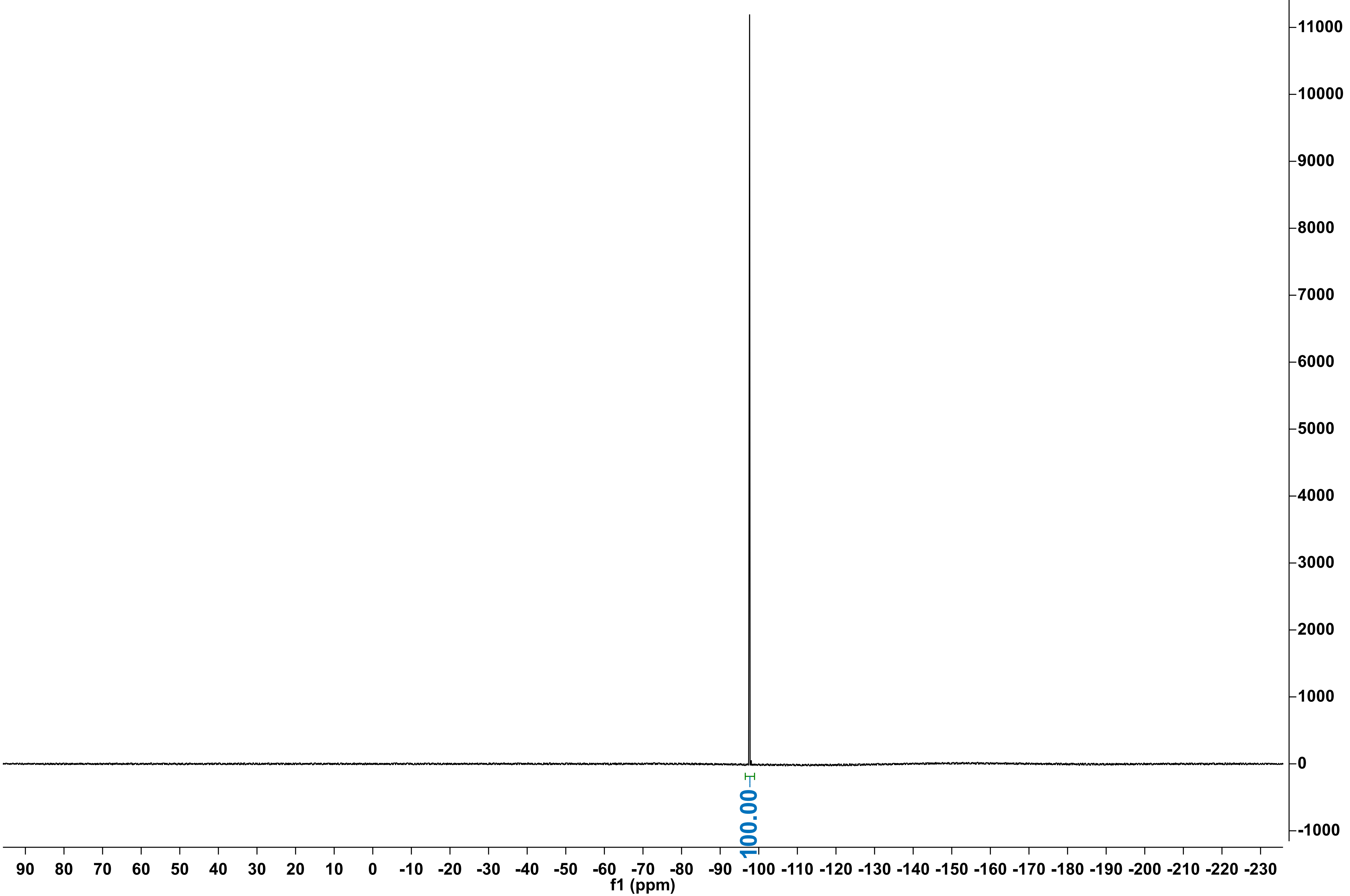

P50_sample18_WT3_2FPA_72h_20250511164455
STANDARD FLUORINE PARAMETERS

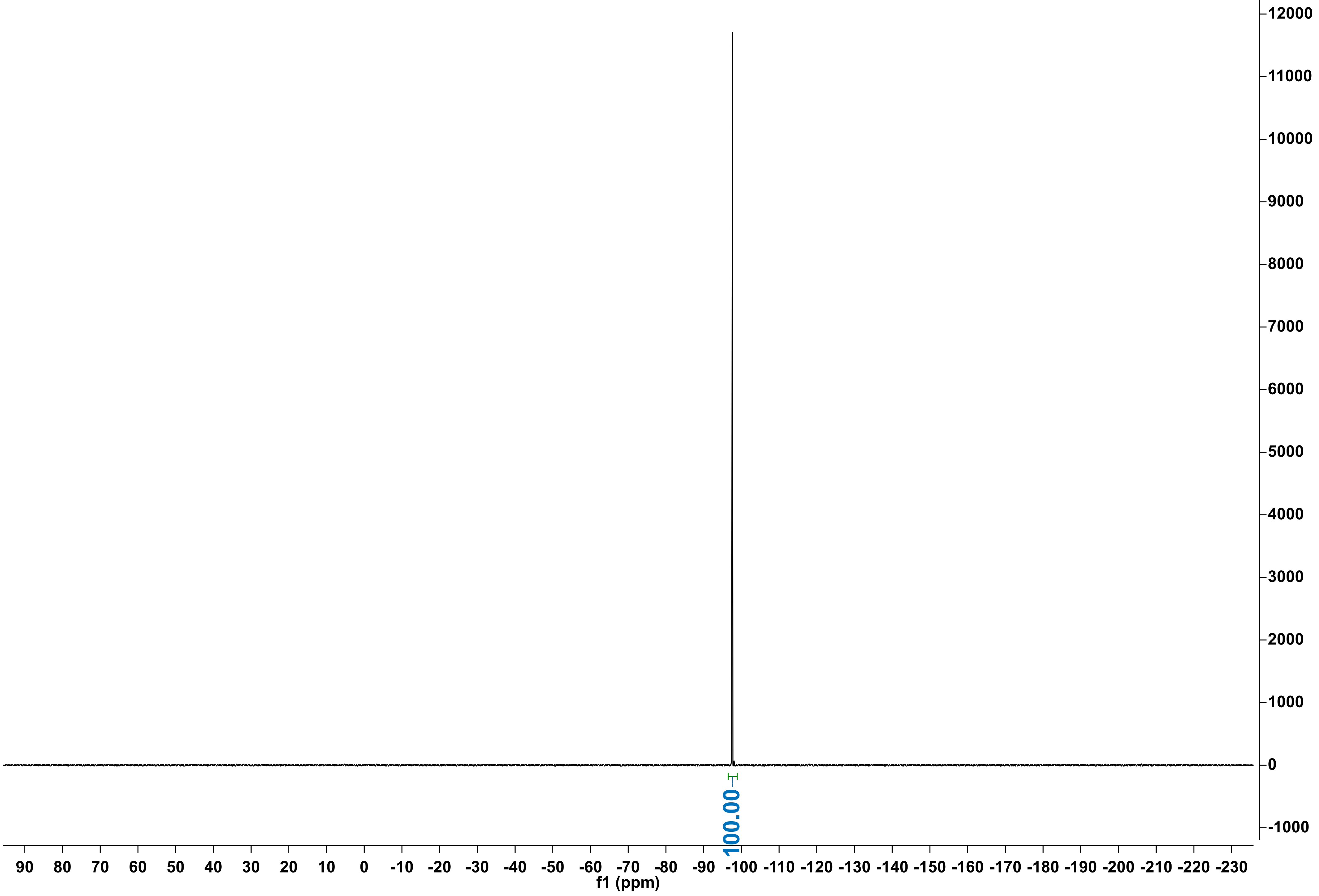

P50_sample22_null1_2FPA_72h_20250511164554
STANDARD FLUORINE PARAMETERS

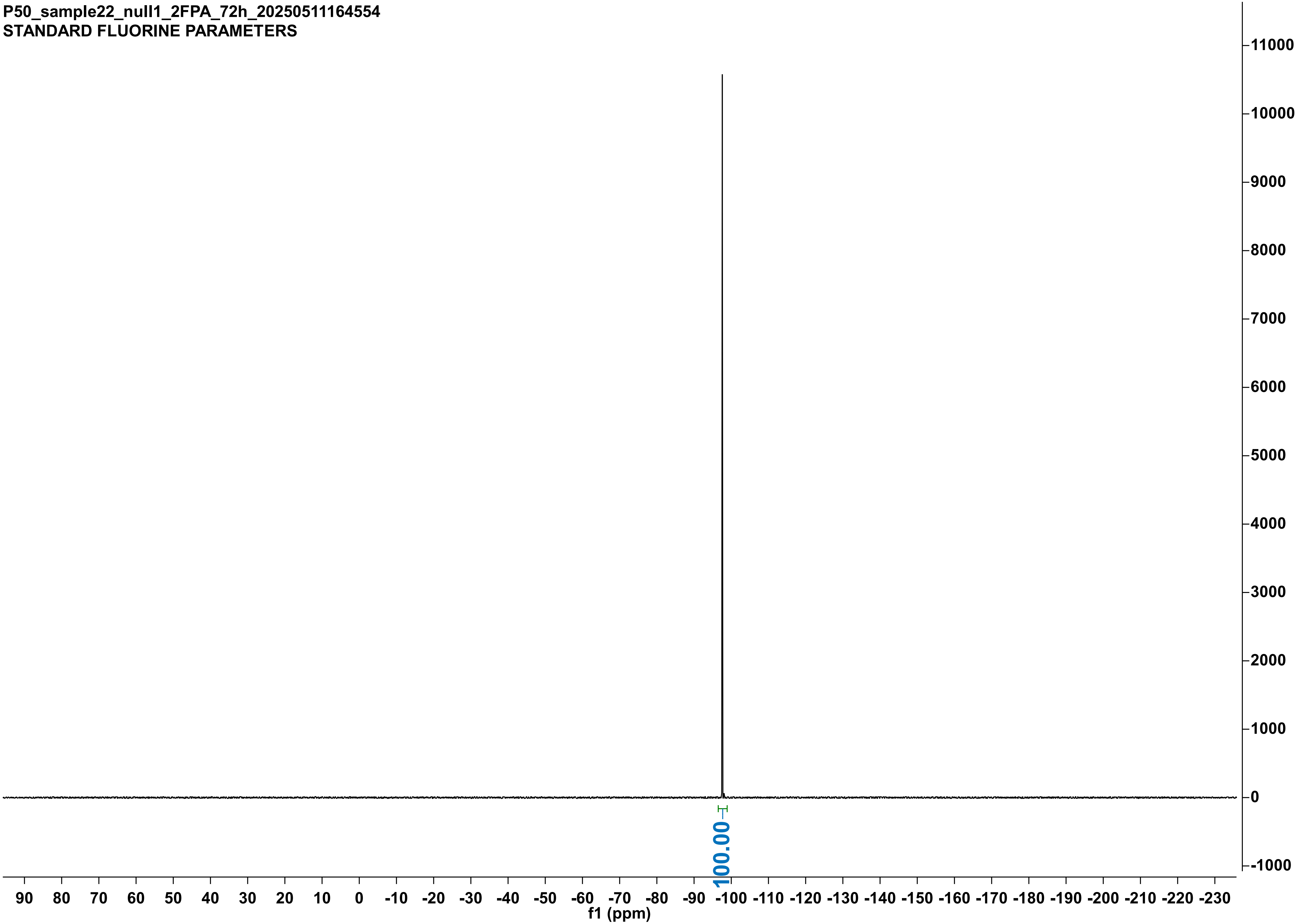

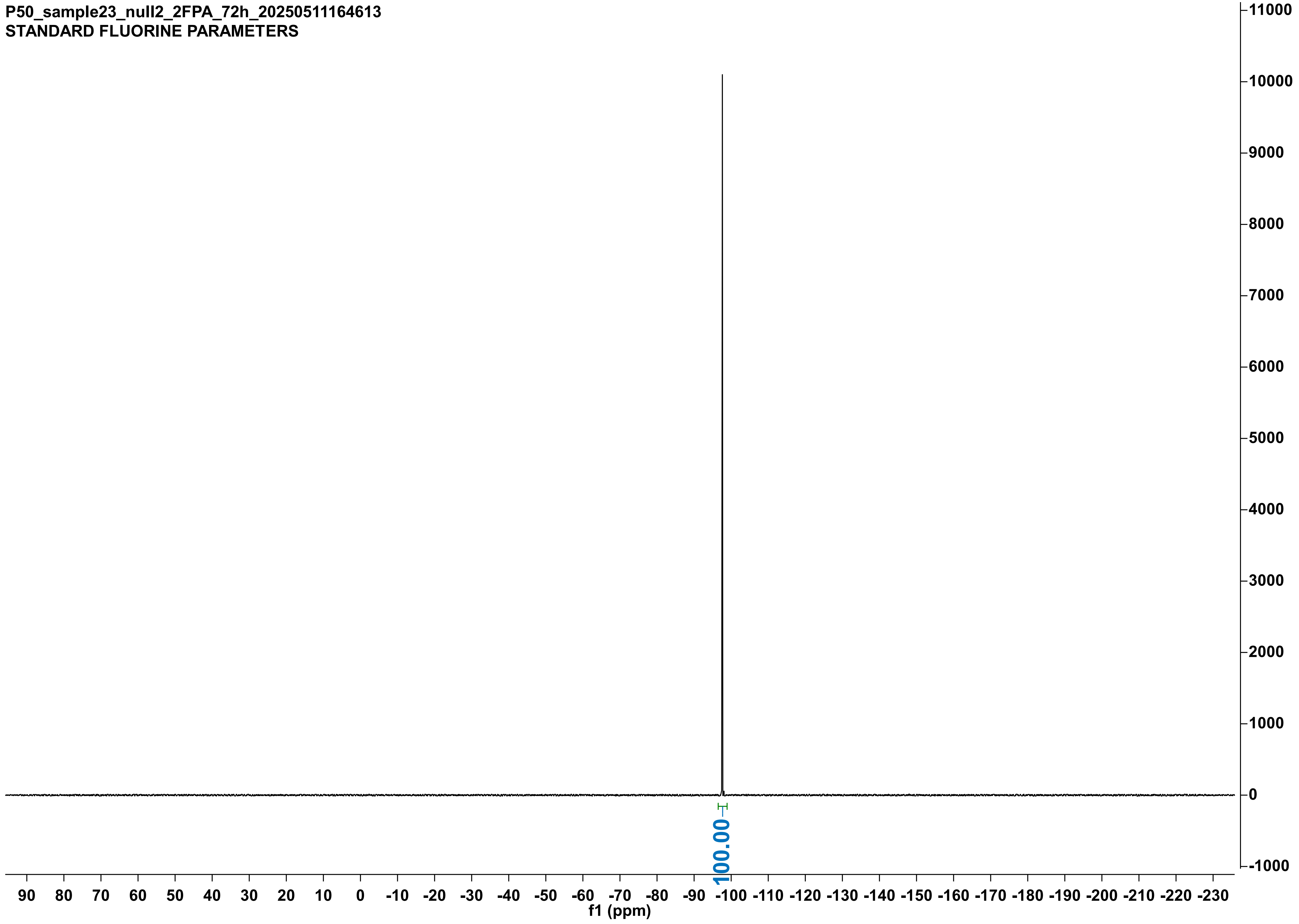

P50_sample23_null2_2FPA_72h_20250511164613
STANDARD FLUORINE PARAMETERS
100.00
f1 (ppm)
90 80 70 60 50 40 30 20 10 0 -10 -20 -30 -40 -50 -60 -70 -80 -90 -100 -110 -120 -130 -140 -150 -160 -170 -180 -190 -200 -210 -220 -230
11000 10000 9000 8000 7000 6000 5000 4000 3000 2000 1000 0 -1000

P50_sample24_null3_2FPA_72h_20250511164624
STANDARD FLUORINE PARAMETERS

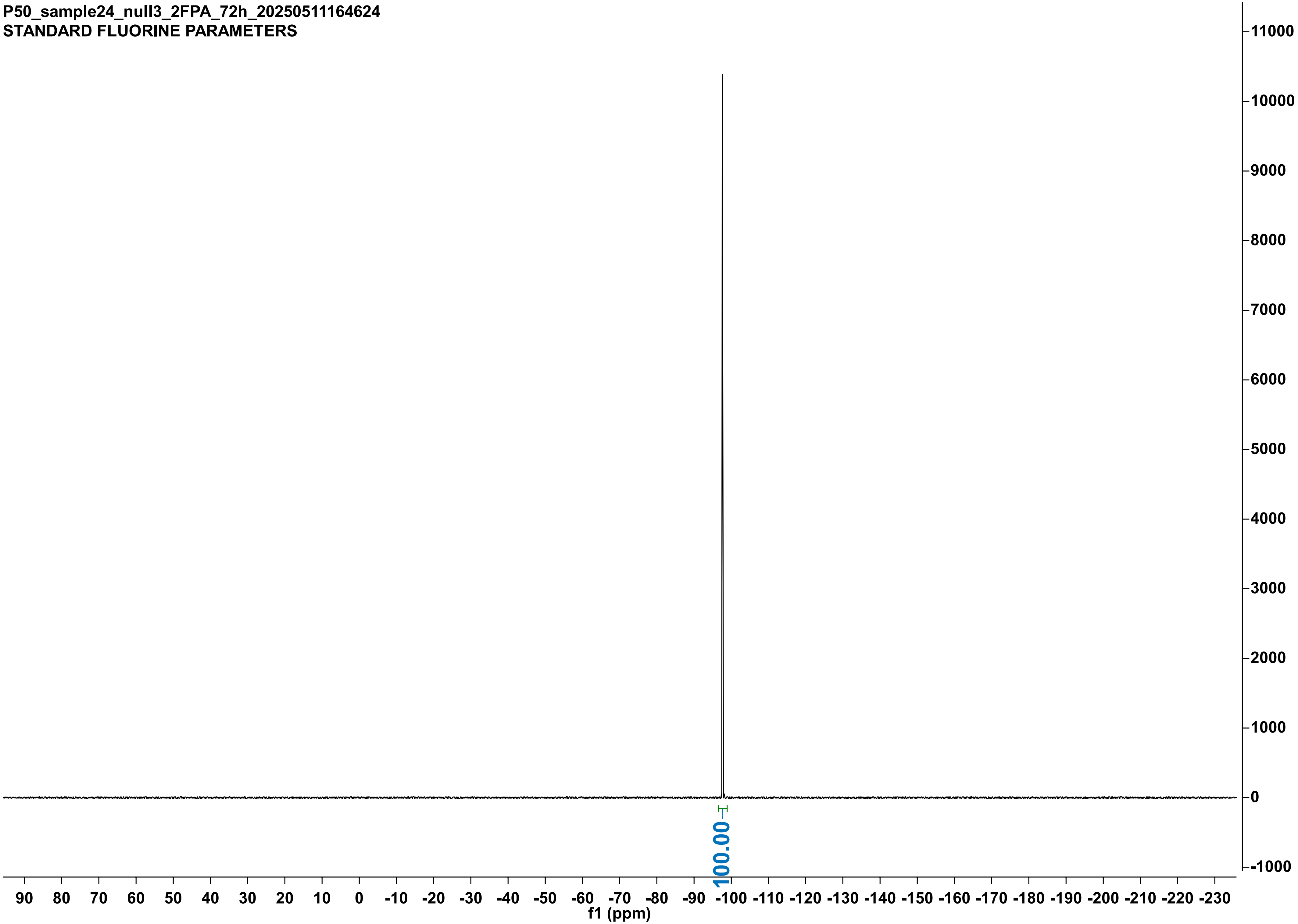

X48-11-4-05uMH272A-2-10mMFAc-20min_20250411112926
STANDARD FLUORINE PARAMETERS

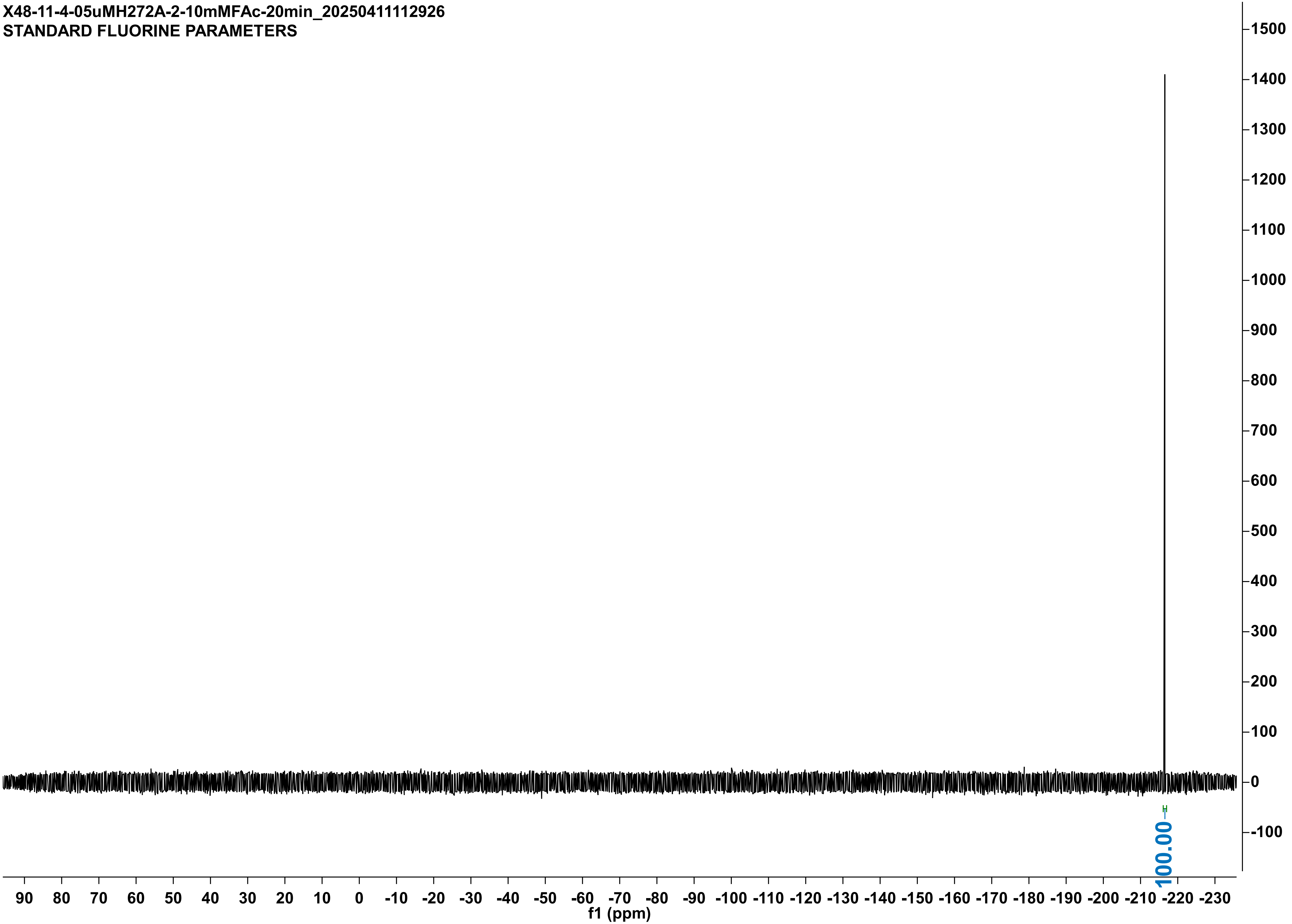

X48-11-4-05uMWT1-remade-10mMFAc-1min_20250411140808
STANDARD FLUORINE PARAMETERS

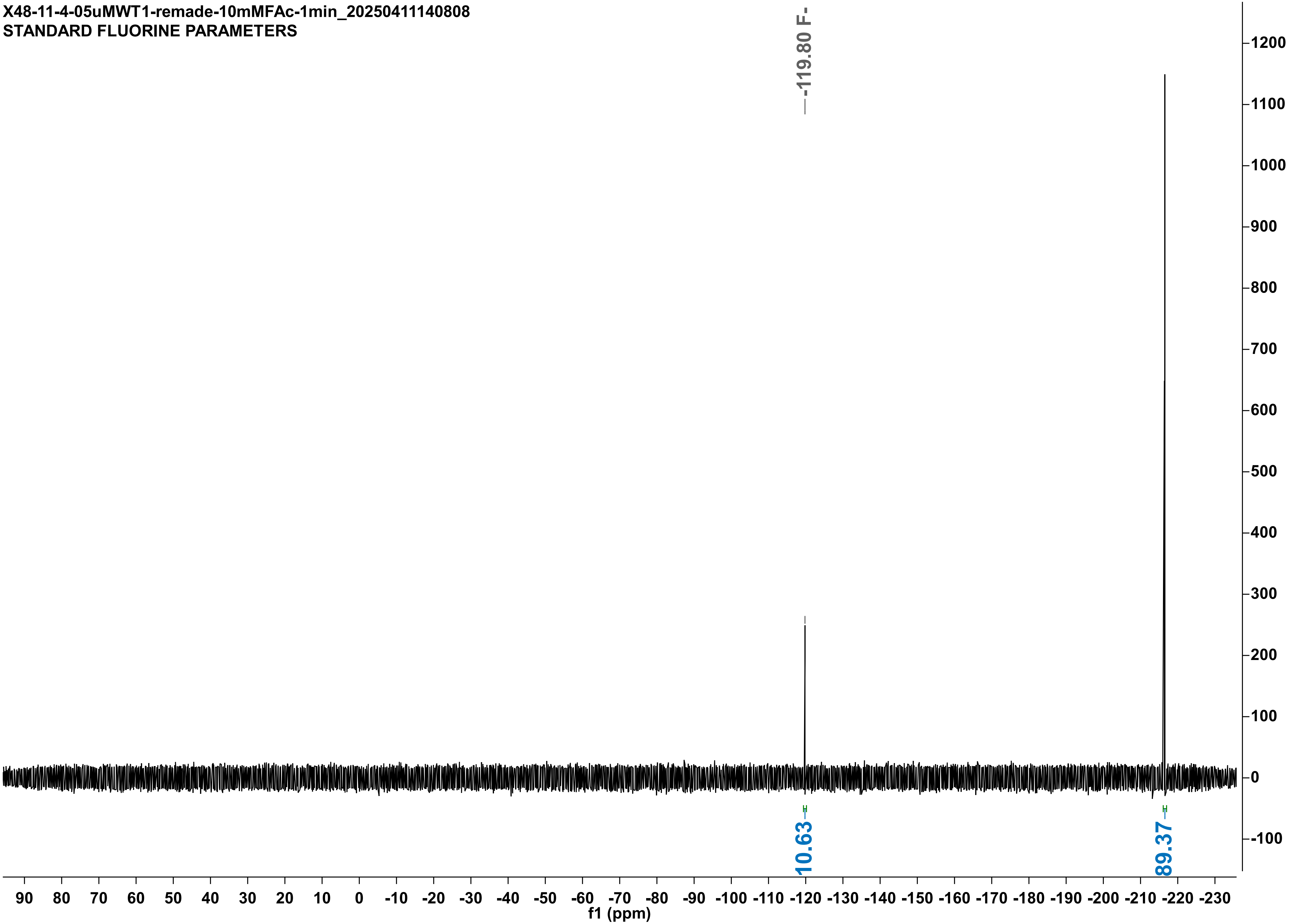

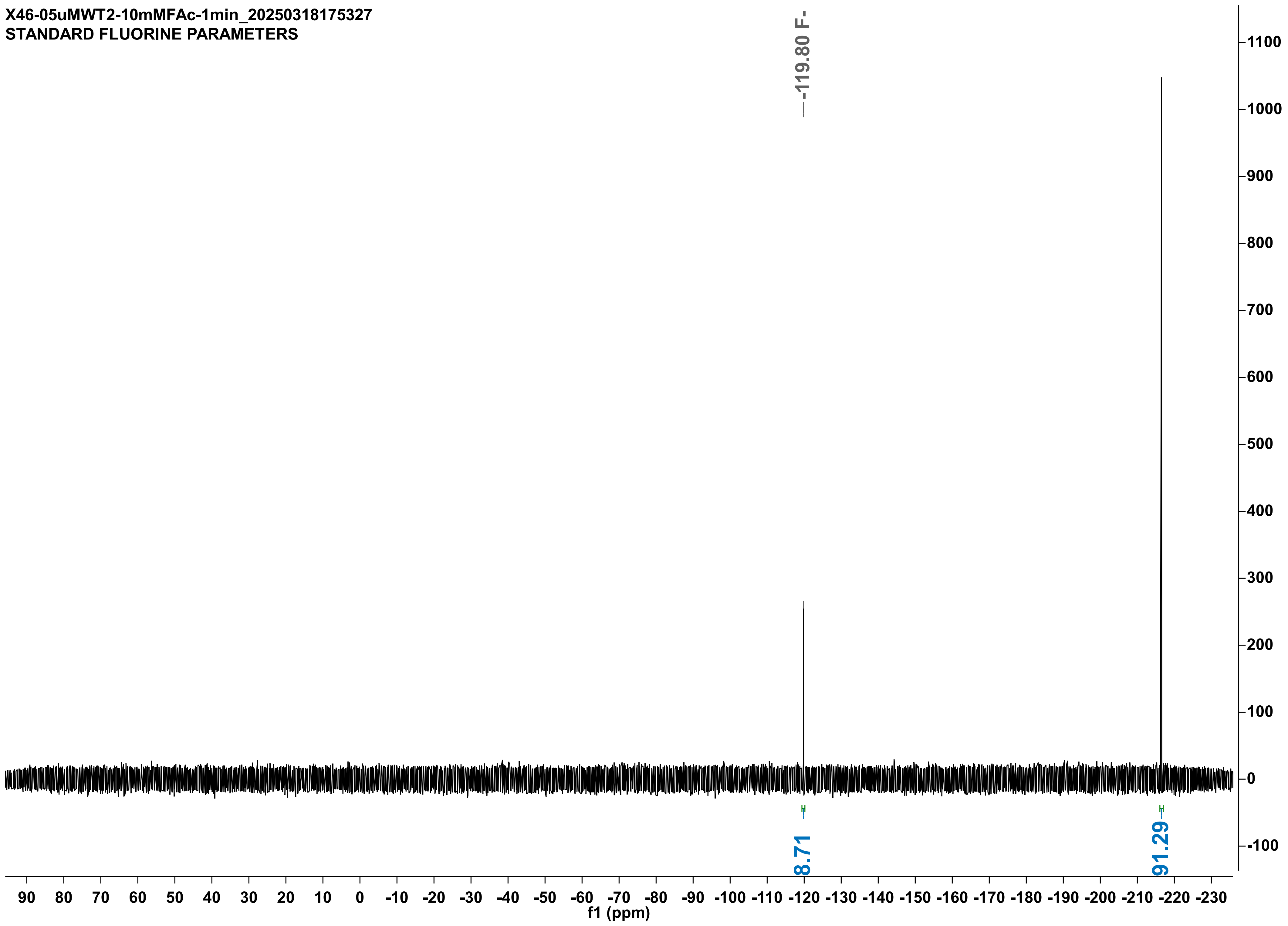

X46-05uMWT2-10mMFAc-1min_20250318175327
STANDARD FLUORINE PARAMETERS
-119.80 F-
8.71
91.29
90 80 70 60 50 40 30 20 10 0 -10 -20 -30 -40 -50 -60 -70 -80 -90 -100 -110 -120 -130 -140 -150 -160 -170 -180 -190 -200 -210 -220 -230
f1 (ppm)
1100
1000
900
800
700
600
500
400
300
200
100
0
-100

X48-11-4-05uMRPL-10mMFAc-20min_20250411161735
STANDARD FLUORINE PARAMETERS

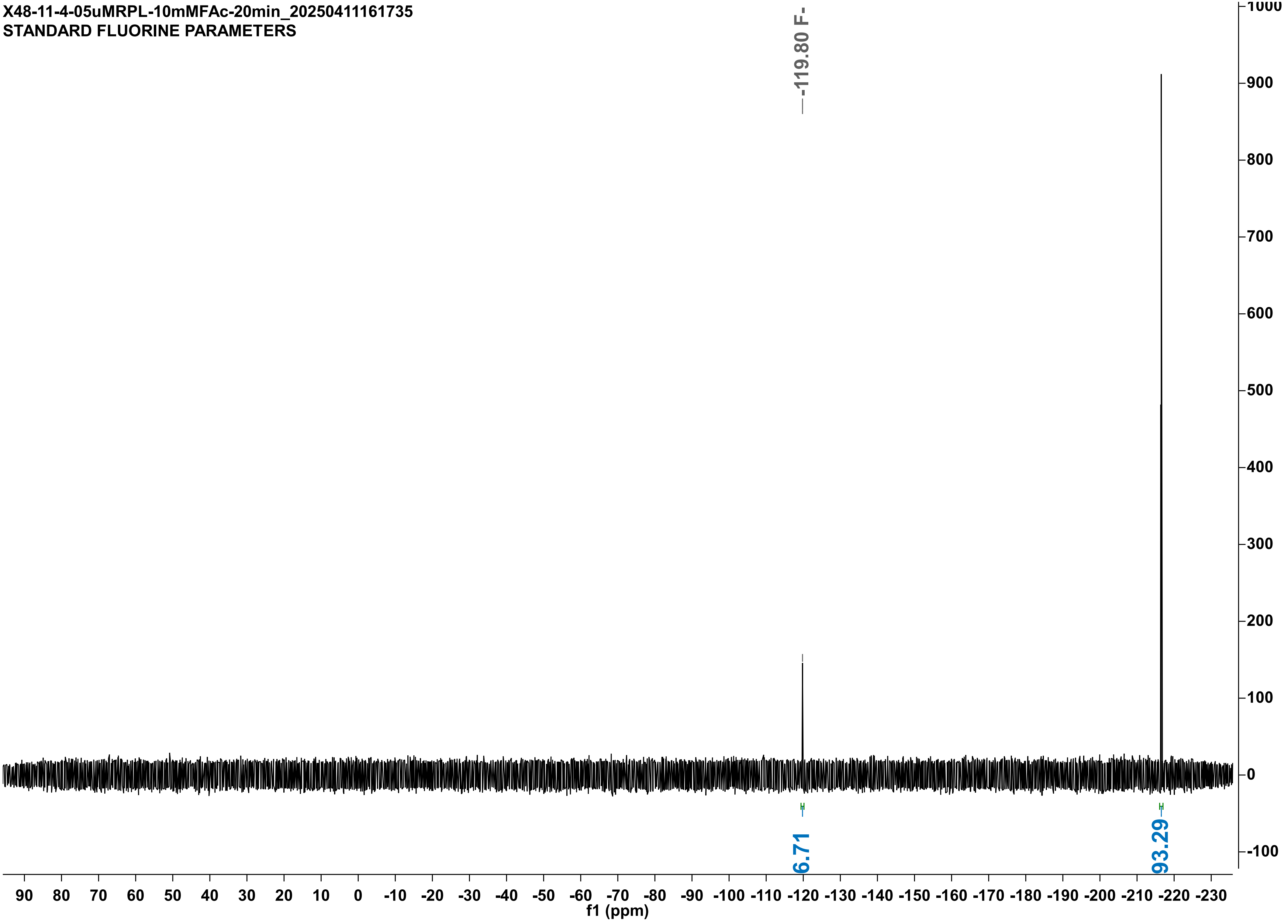

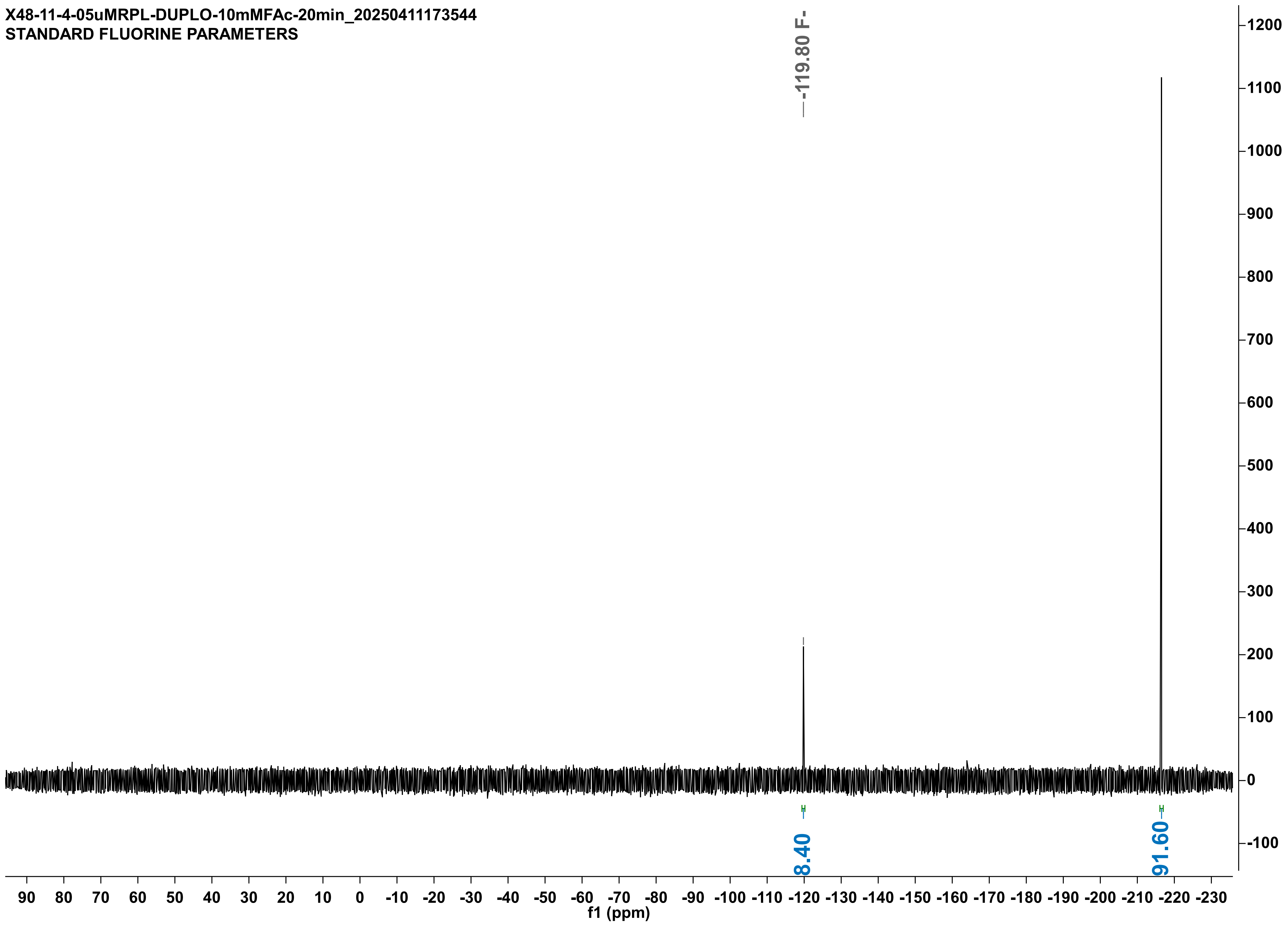
X48-11-4-05uMRPL-DUPLO-10mMFAc-20min_20250411173544
STANDARD FLUORINE PARAMETERS
-119.80 F-
8.40
91.60
f1 (ppm)

X48-11-4-05uMVW--10mMFAc-5min__20250411140616
STANDARD FLUORINE PARAMETERS

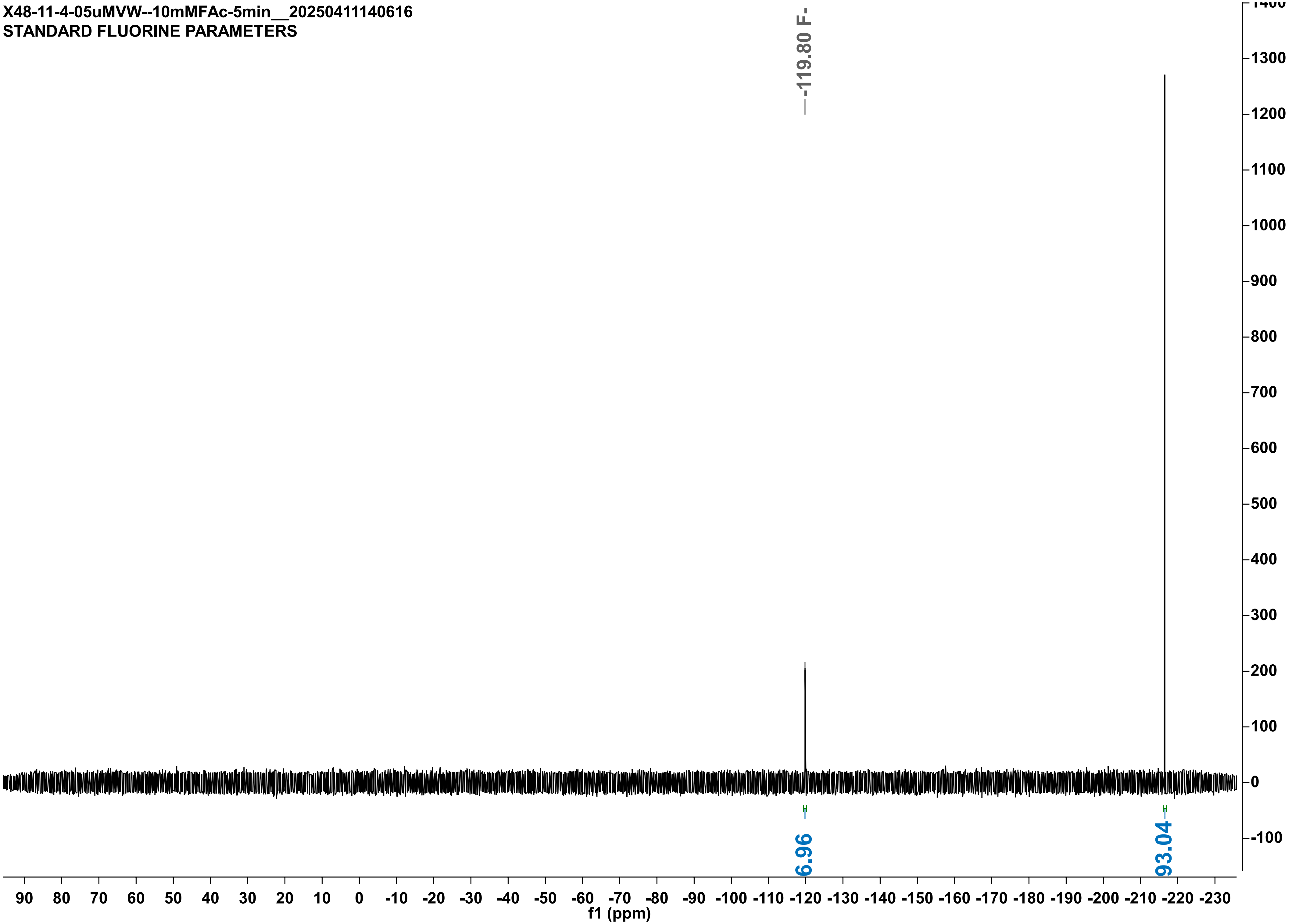

X48-11-4-05uMVW--duplo-10mMFAc-5min_20250411155403
STANDARD FLUORINE PARAMETERS

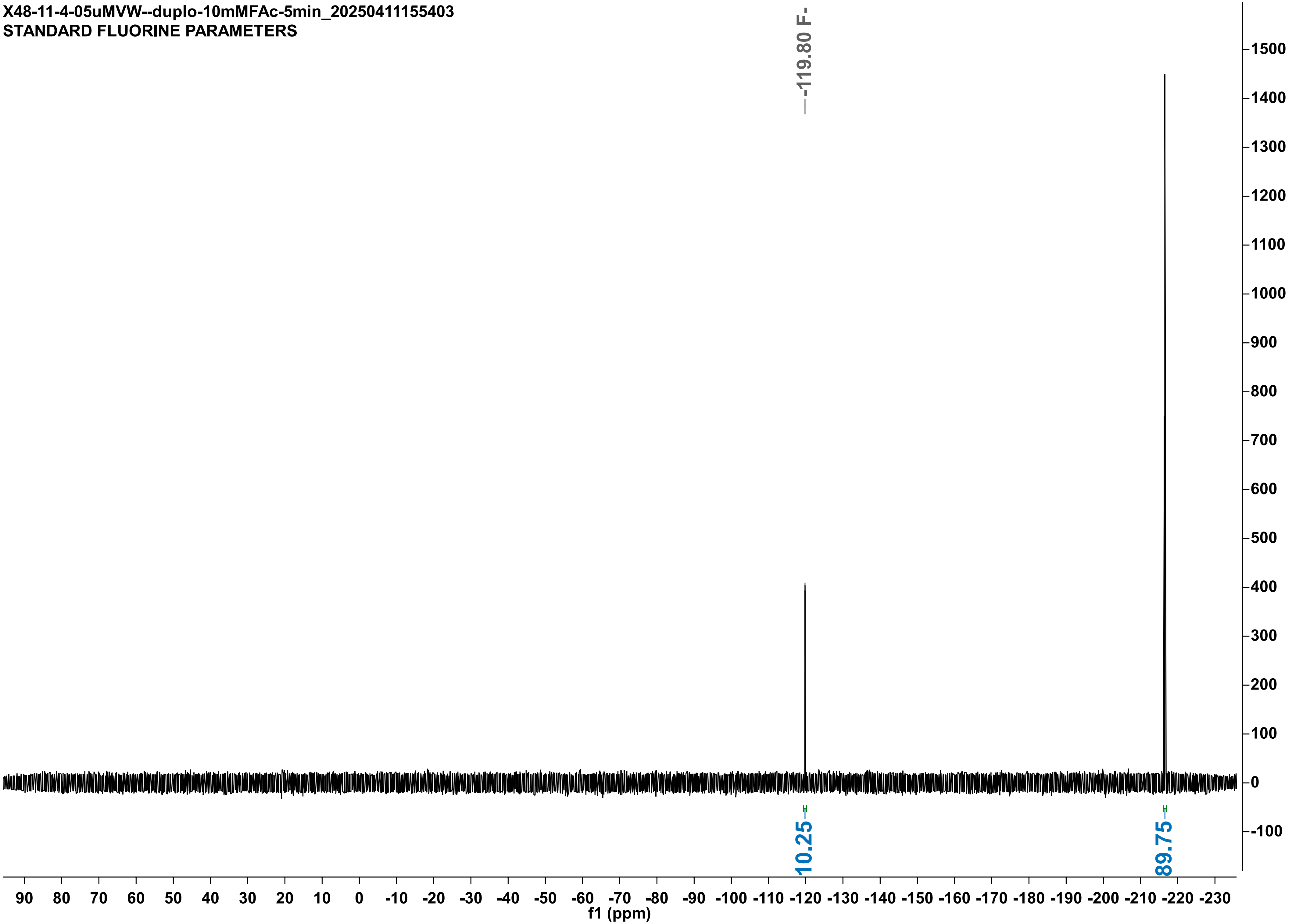

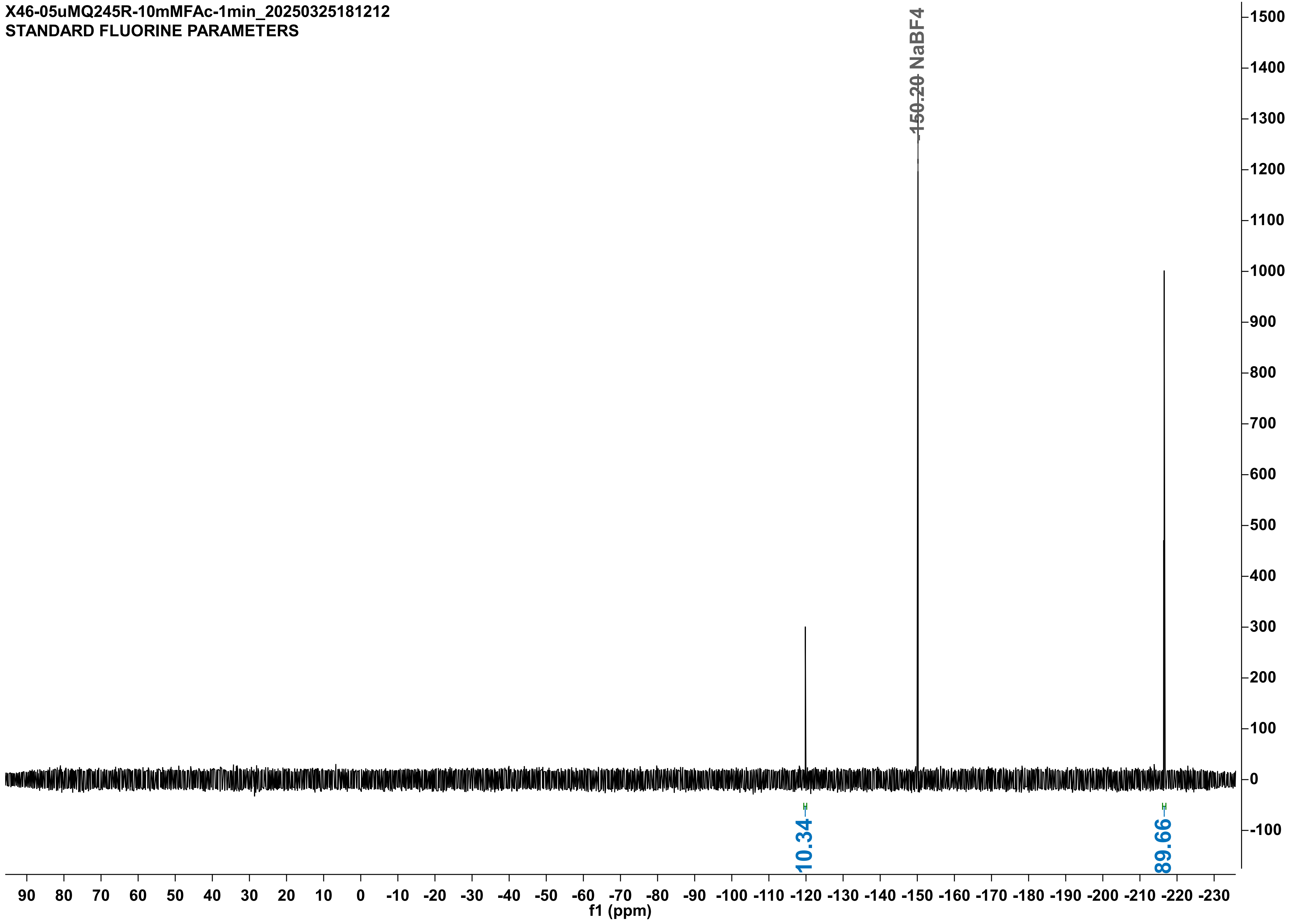

X46-05uMQ245R-10mMFAc-1min_20250325181212
STANDARD FLUORINE PARAMETERS
-150.20 NaBF4
10.34
89.66
f1 (ppm)

X48-16-4-05uMQ245R-10mMFAc-60sec_20250416210642
STANDARD FLUORINE PARAMETERS

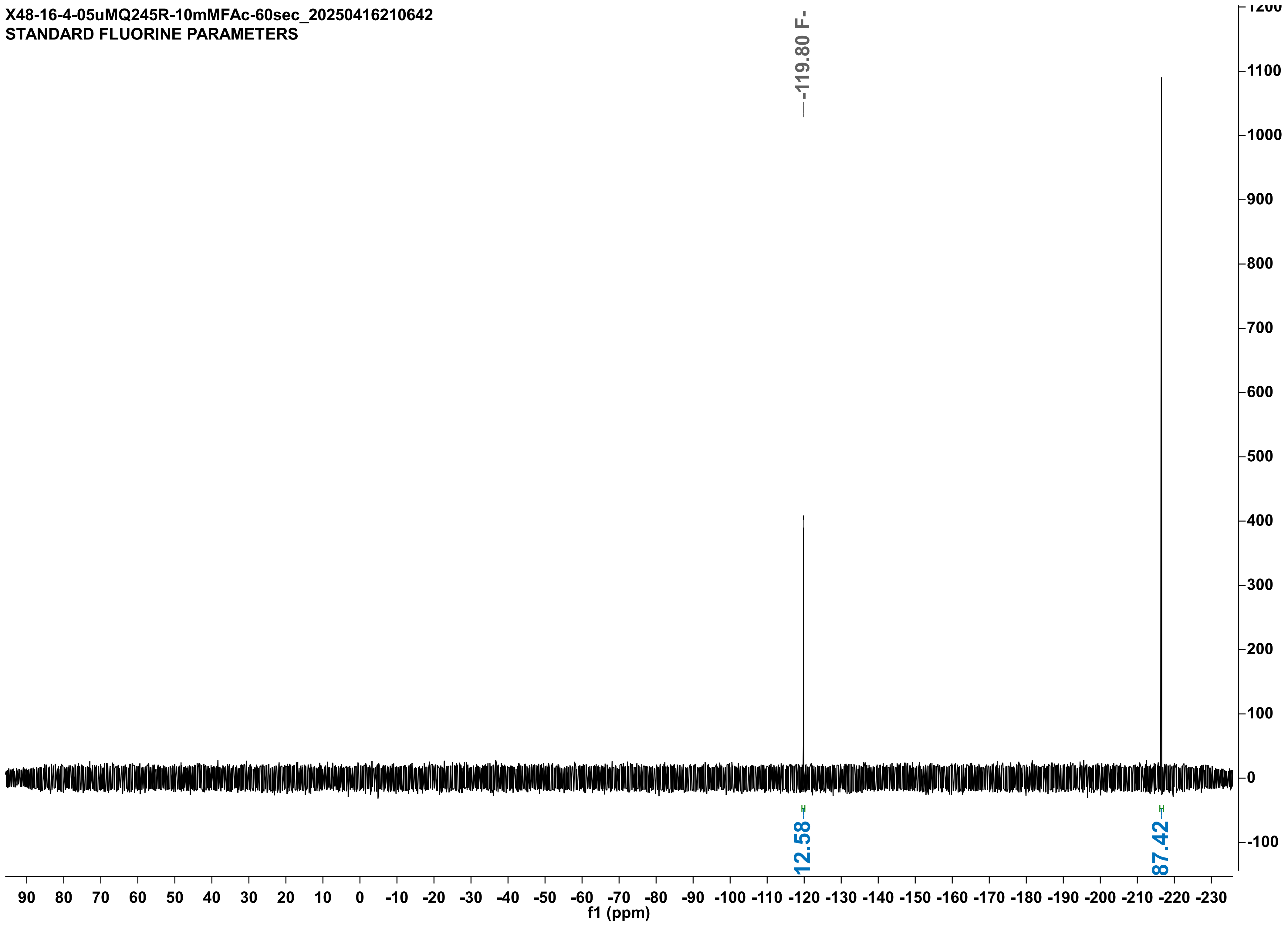

X48-14-4-05uM-ML-10mMFAc-3min_20250414183622
STANDARD FLUORINE PARAMETERS

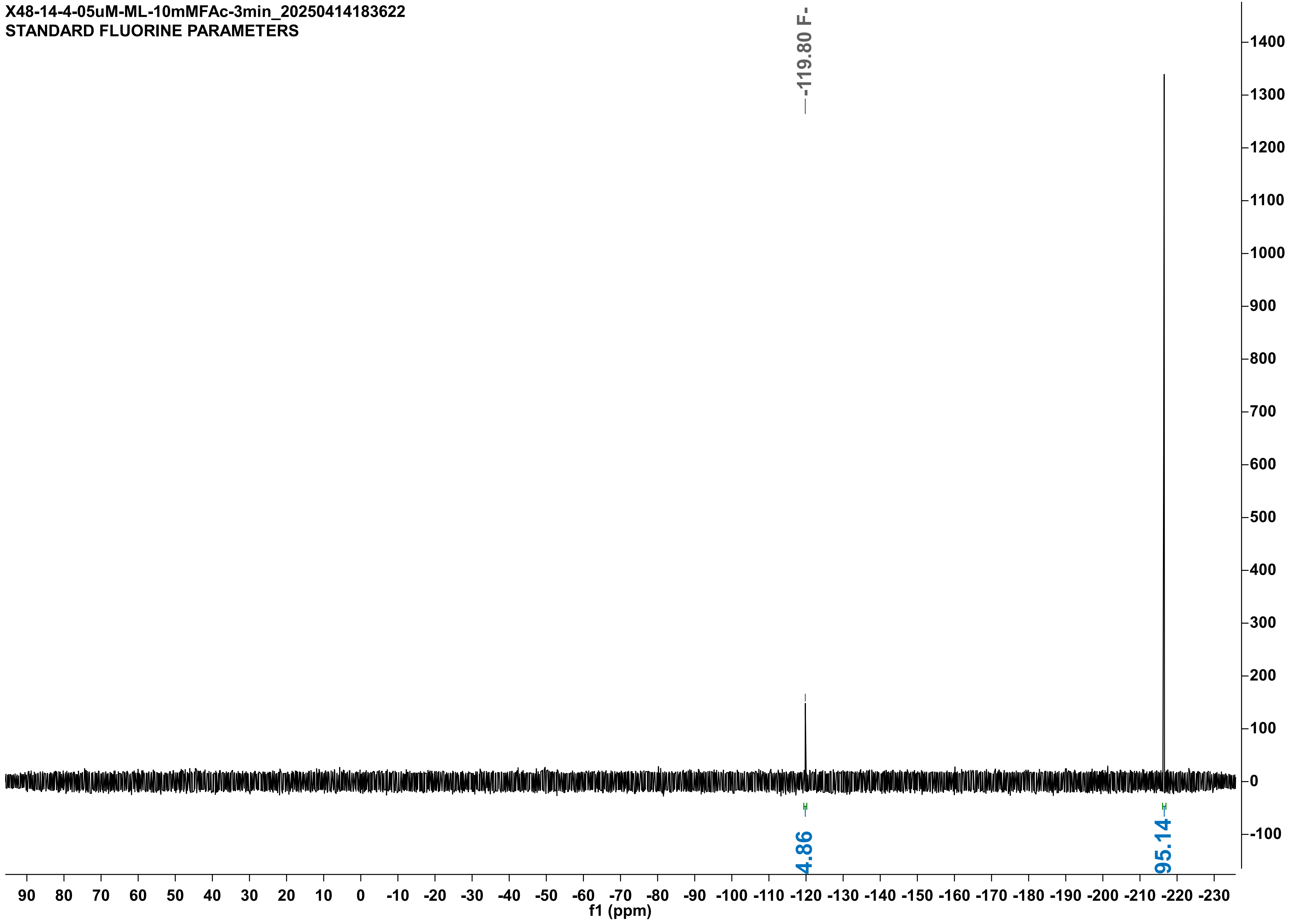

X48-14-4-05uM-ML-DUPLO-10mMFAc-3min_20250414190702
STANDARD FLUORINE PARAMETERS

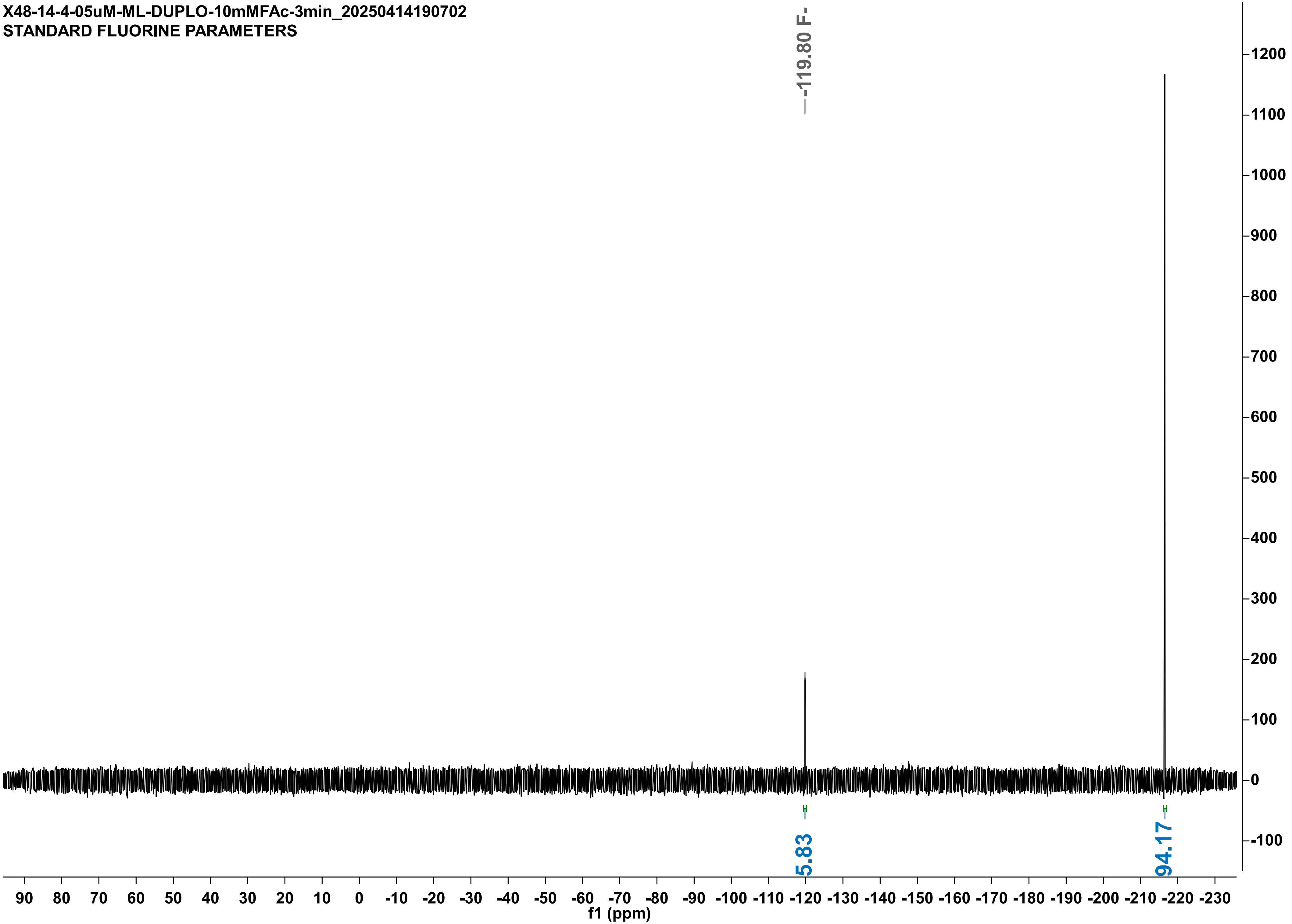

X48-14-4-05uMRHL-10mMFAc-7min_20250414190743
STANDARD FLUORINE PARAMETERS

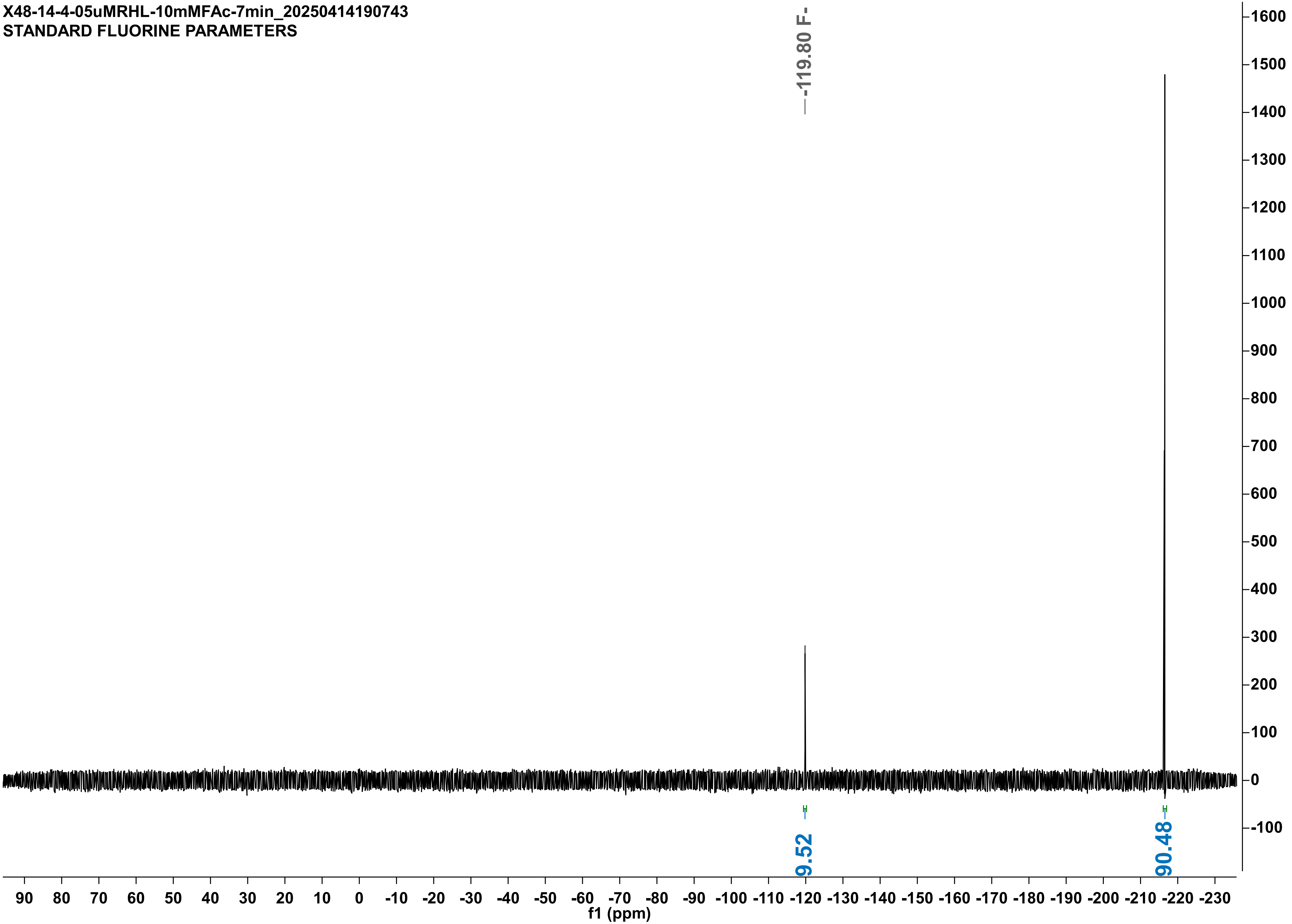

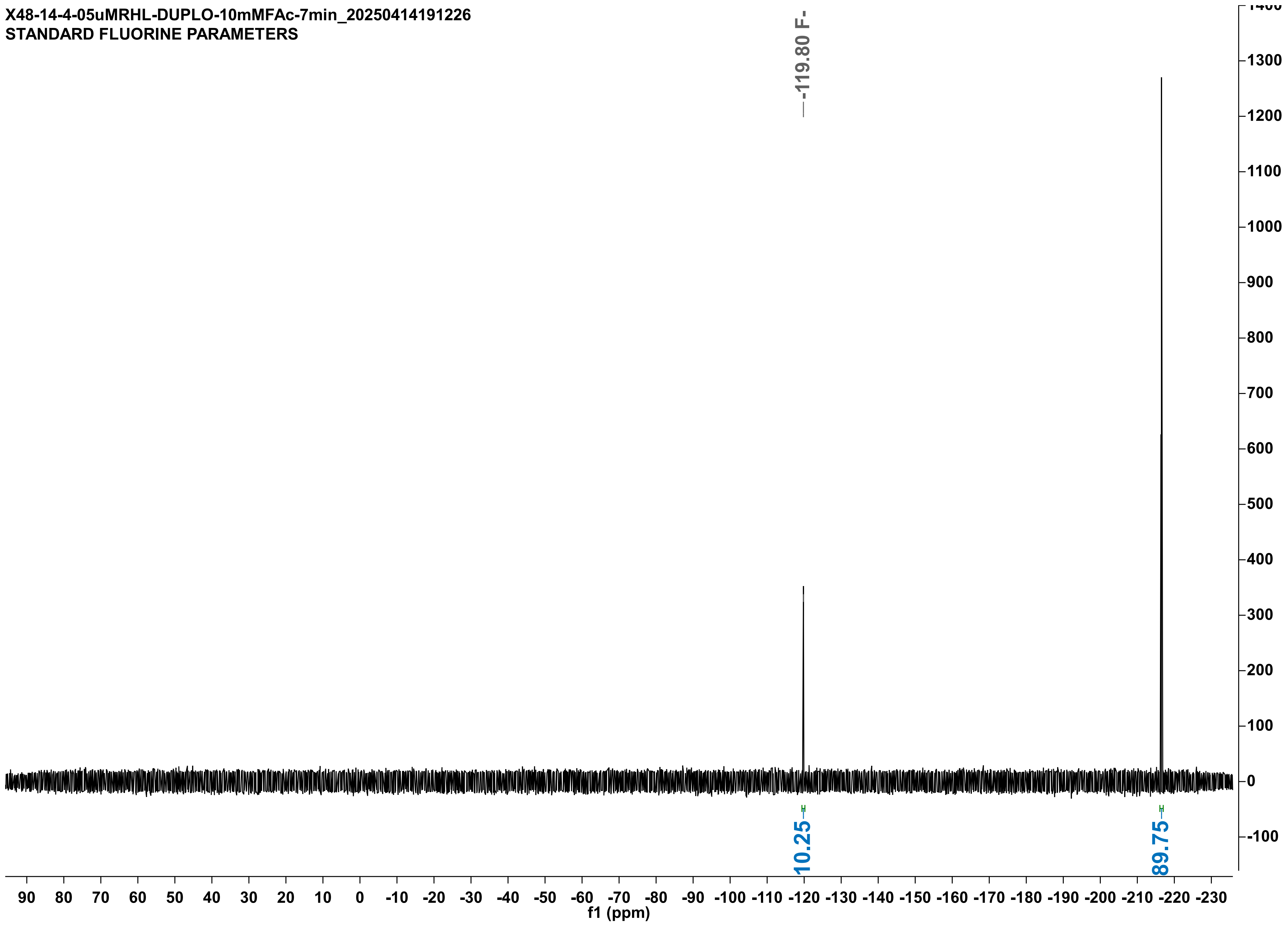

X48-14-4-05uMRHL-DUPLO-10mMFAc-7min_20250414191226
STANDARD FLUORINE PARAMETERS
-119.80 F-
10.25
89.75
f1 (ppm)

X48-11-4-05uMQ245A-10mMFAc-45sec_20250411113955
STANDARD FLUORINE PARAMETERS

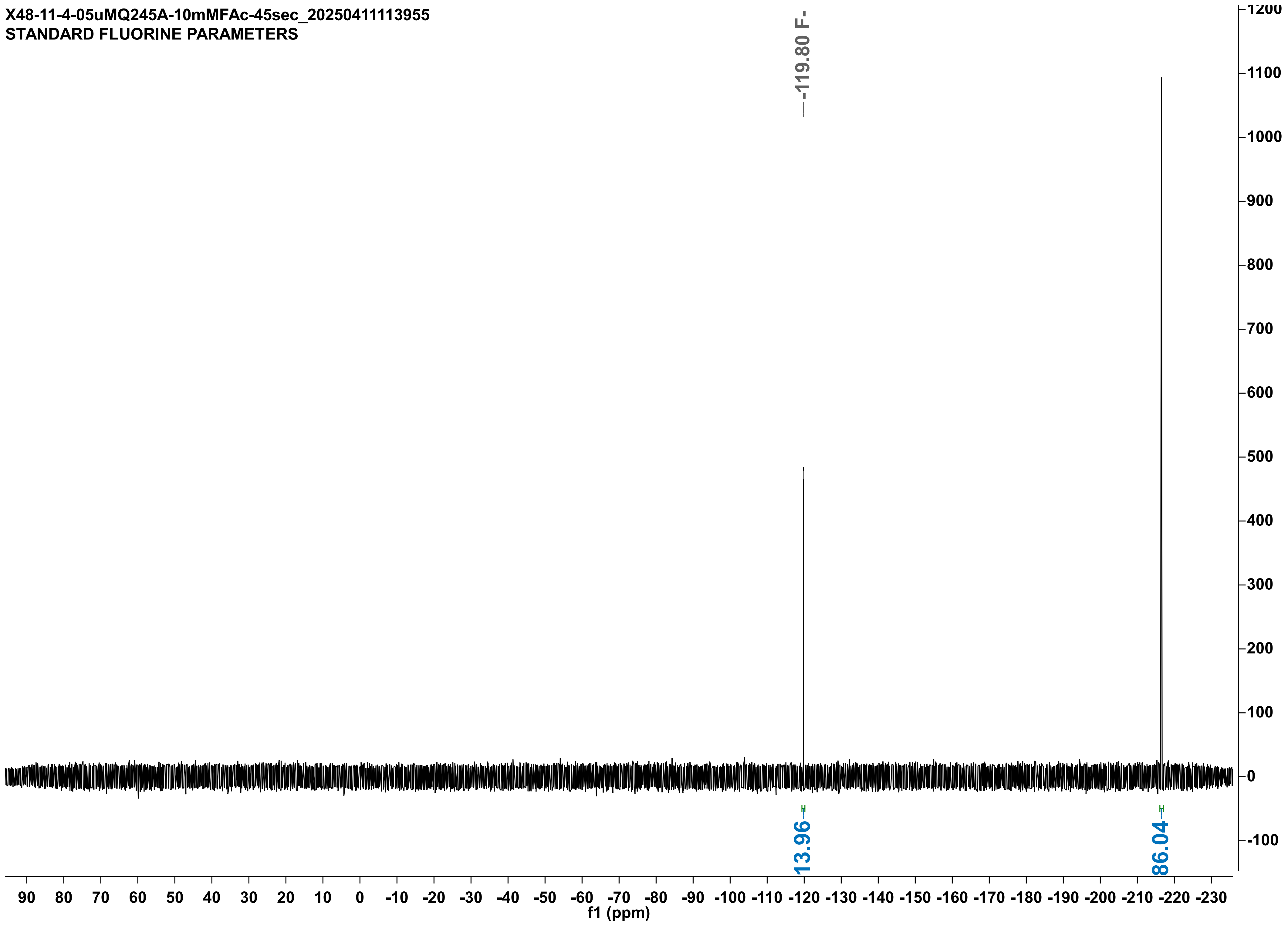

X48-11-4-05uMQ245A-DUPLO-10mMFAc-45sec_20250411132637
STANDARD FLUORINE PARAMETERS

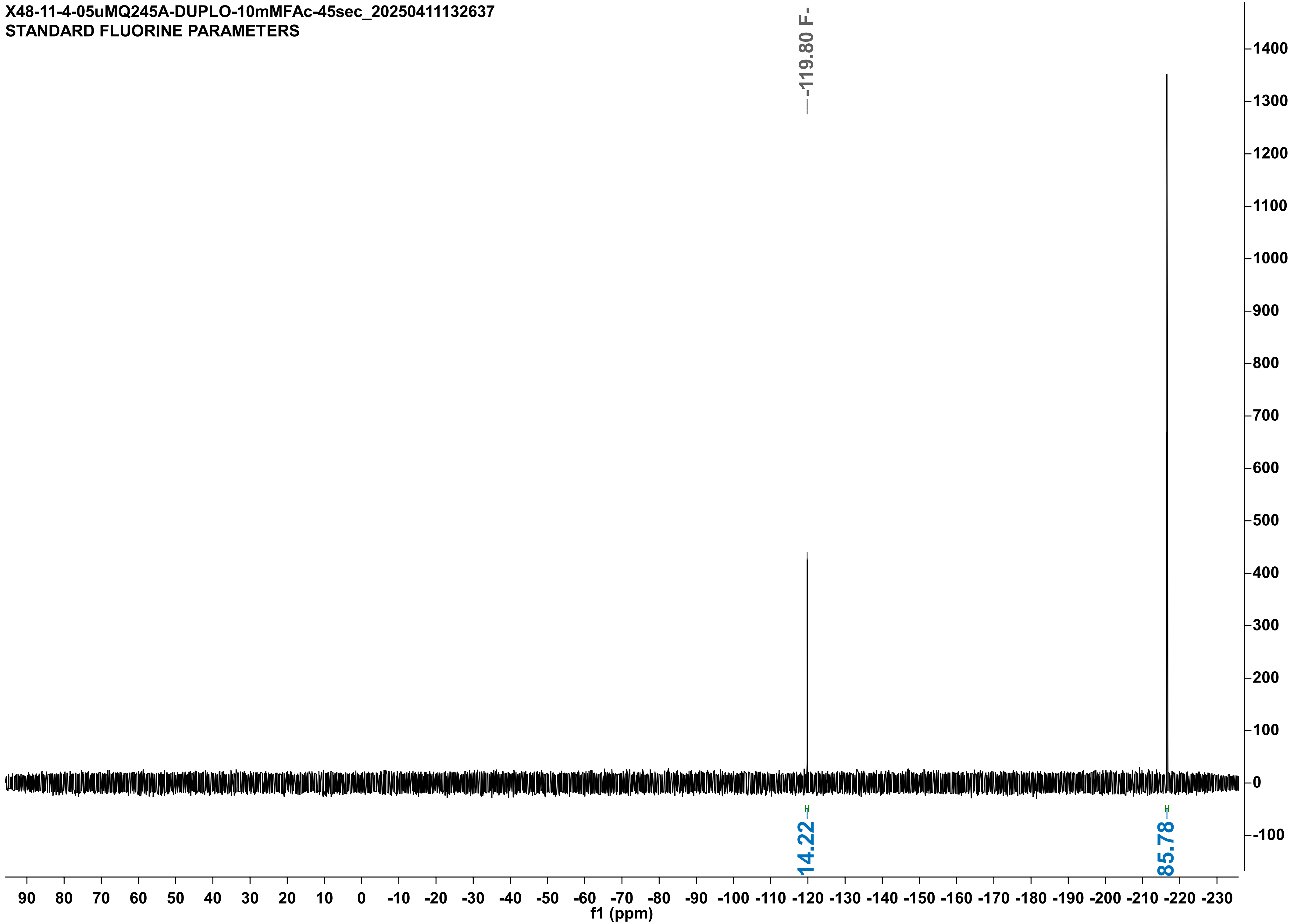

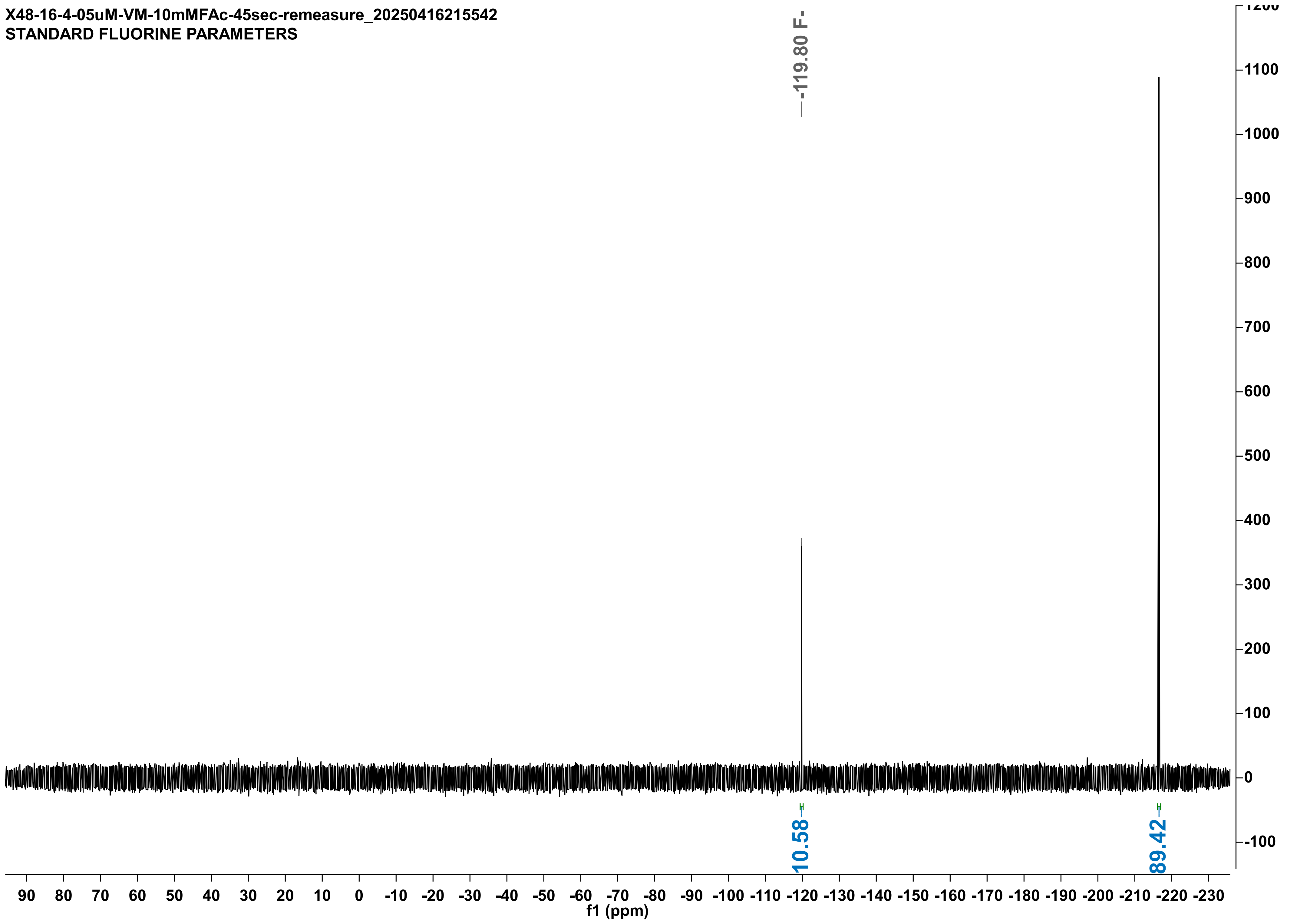

X48-16-4-05uM-VM-10mMFAc-45sec-remeasure_20250416215542
STANDARD FLUORINE PARAMETERS
-119.80 F-
10.58
89.42
f1 (ppm)

X48-16-4-05uM-VM-DUP-10mMFAc-45sec_20250416203831
STANDARD FLUORINE PARAMETERS

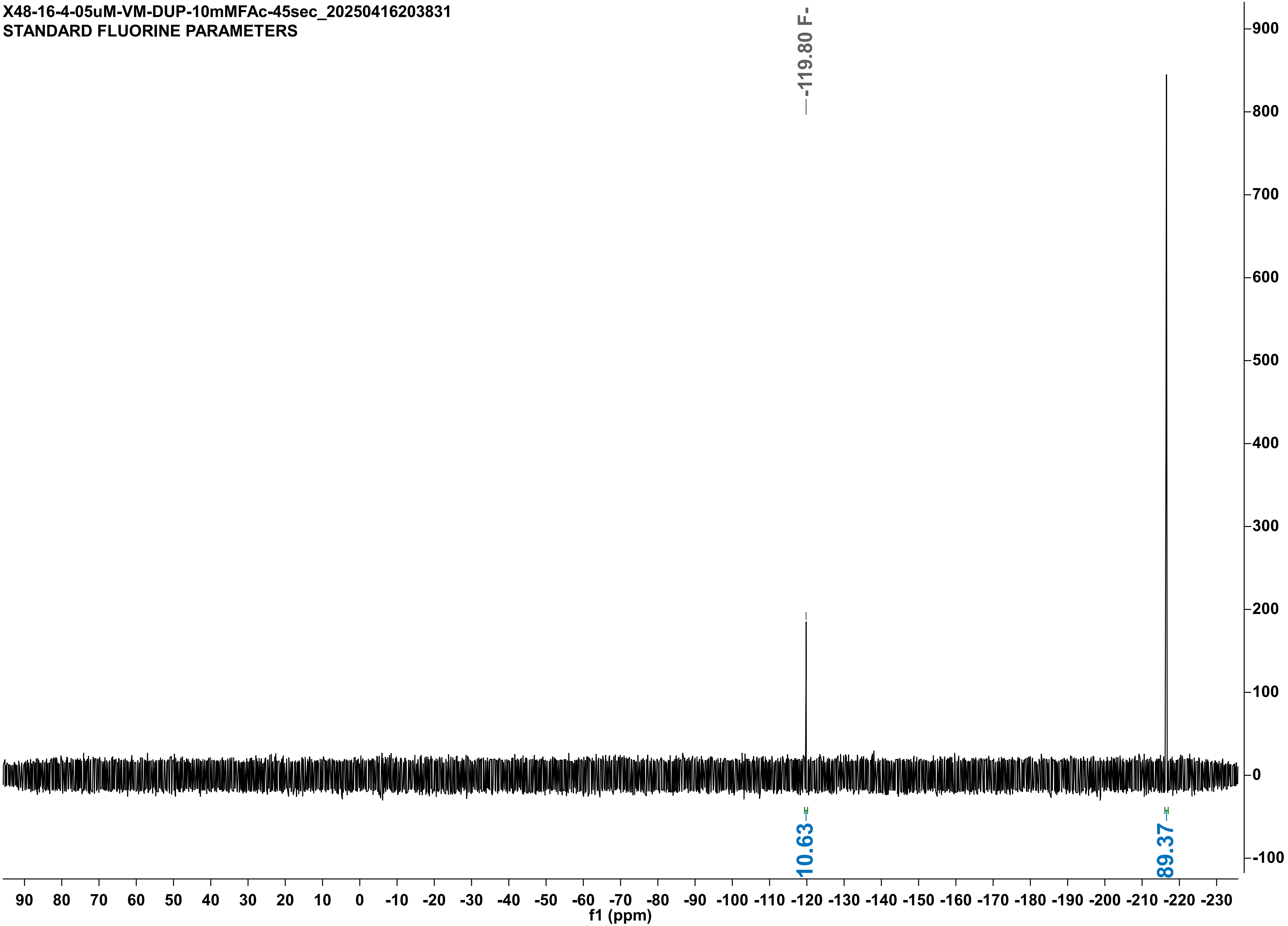

X46-05uMRLM-10mMFAc-1min_20250320160803
STANDARD FLUORINE PARAMETERS

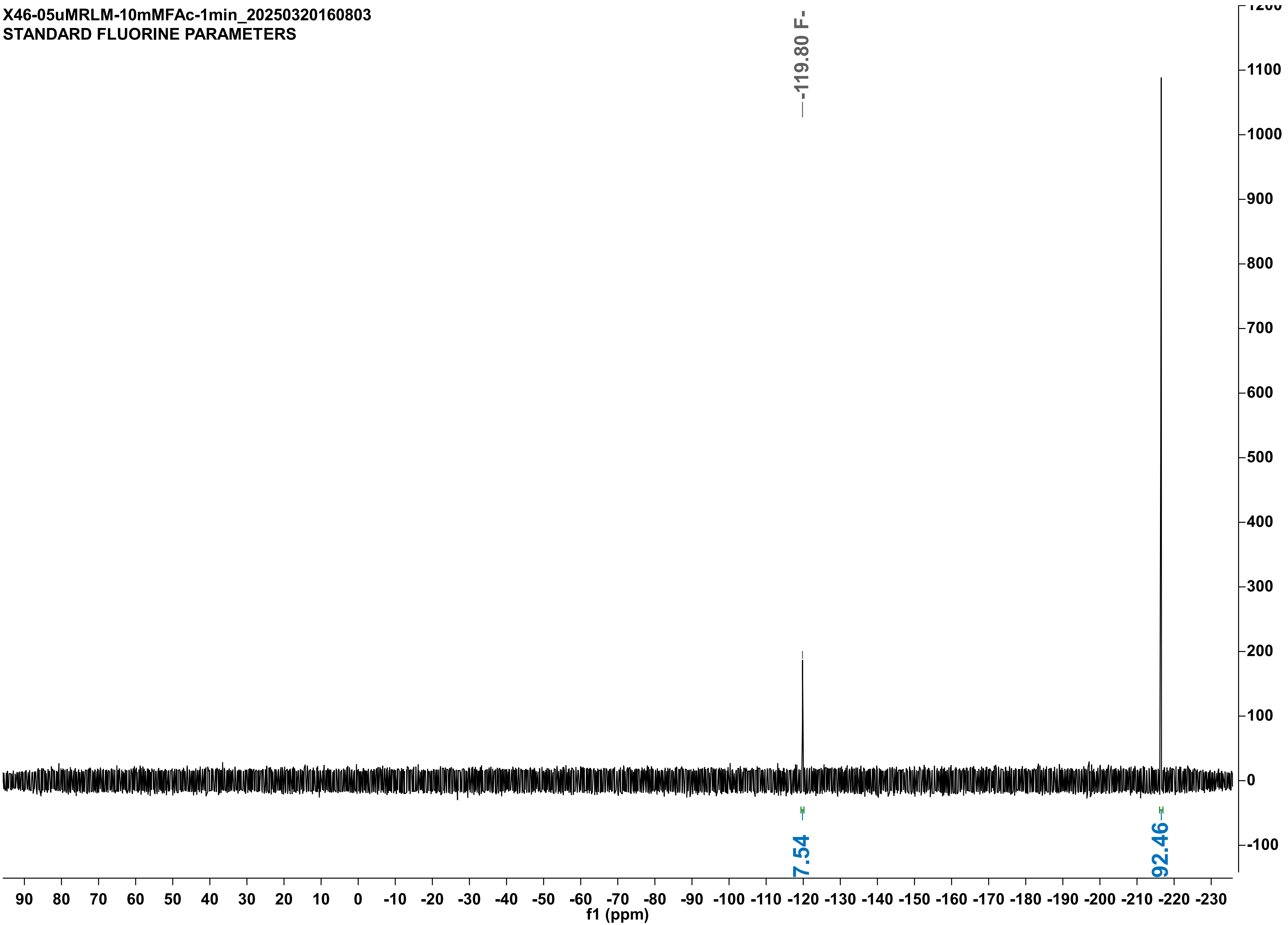

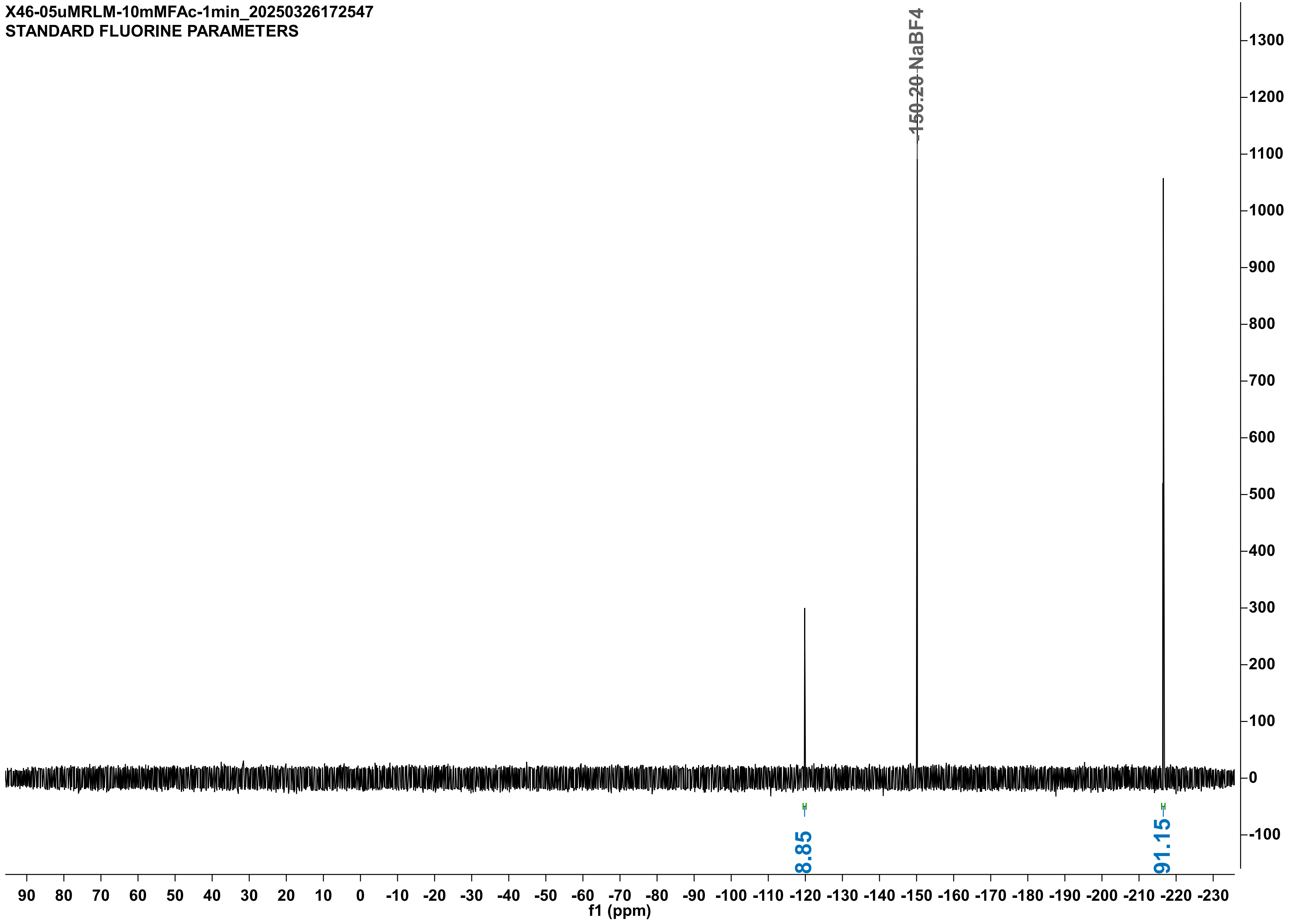
X46-05uMRLM-10mMFAc-1min_20250326172547
STANDARD FLUORINE PARAMETERS
-150.20 NaBF4
8.85
91.15
f1 (ppm)

X48-15-4-05uMQ245G-10mMFAc-1min_20250415191946
STANDARD FLUORINE PARAMETERS

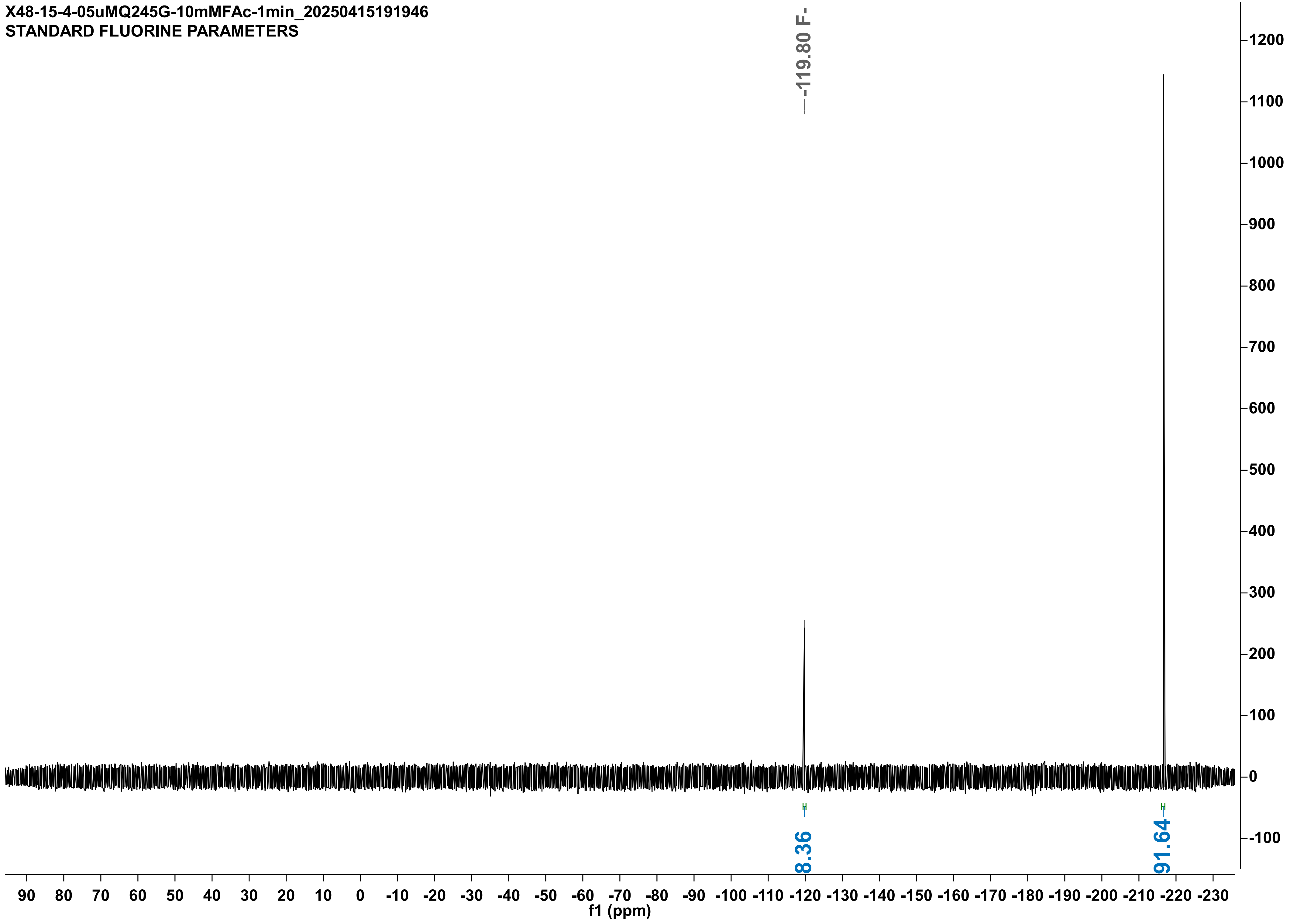

X48-15-4-05uMQ245G-DUP-10mMFAc-1min_20250415192016
STANDARD FLUORINE PARAMETERS

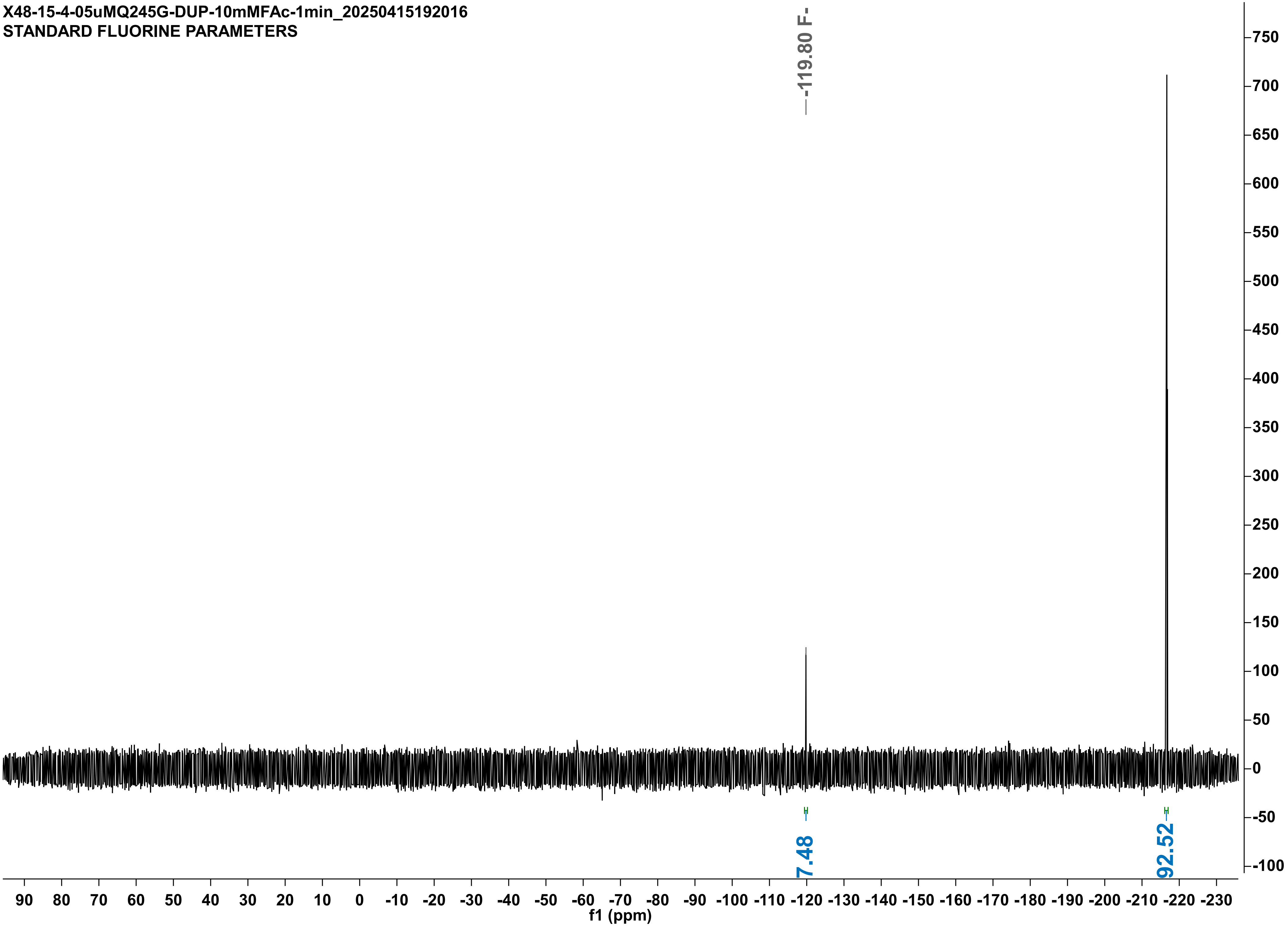

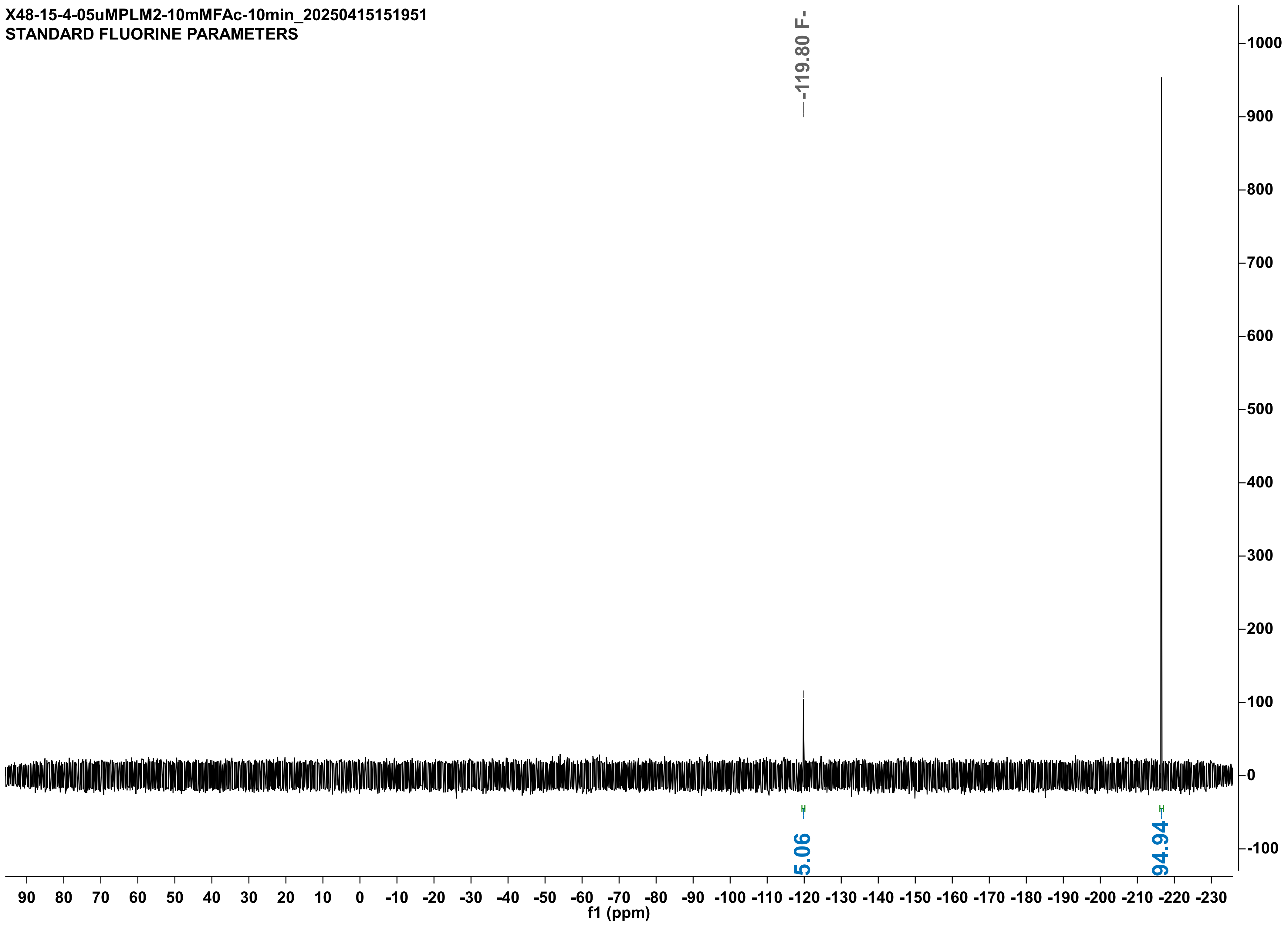

X48-15-4-05uMPLM2-10mMFAc-10min_20250415151951
STANDARD FLUORINE PARAMETERS
-119.80 F-
5.06
94.94
f1 (ppm)

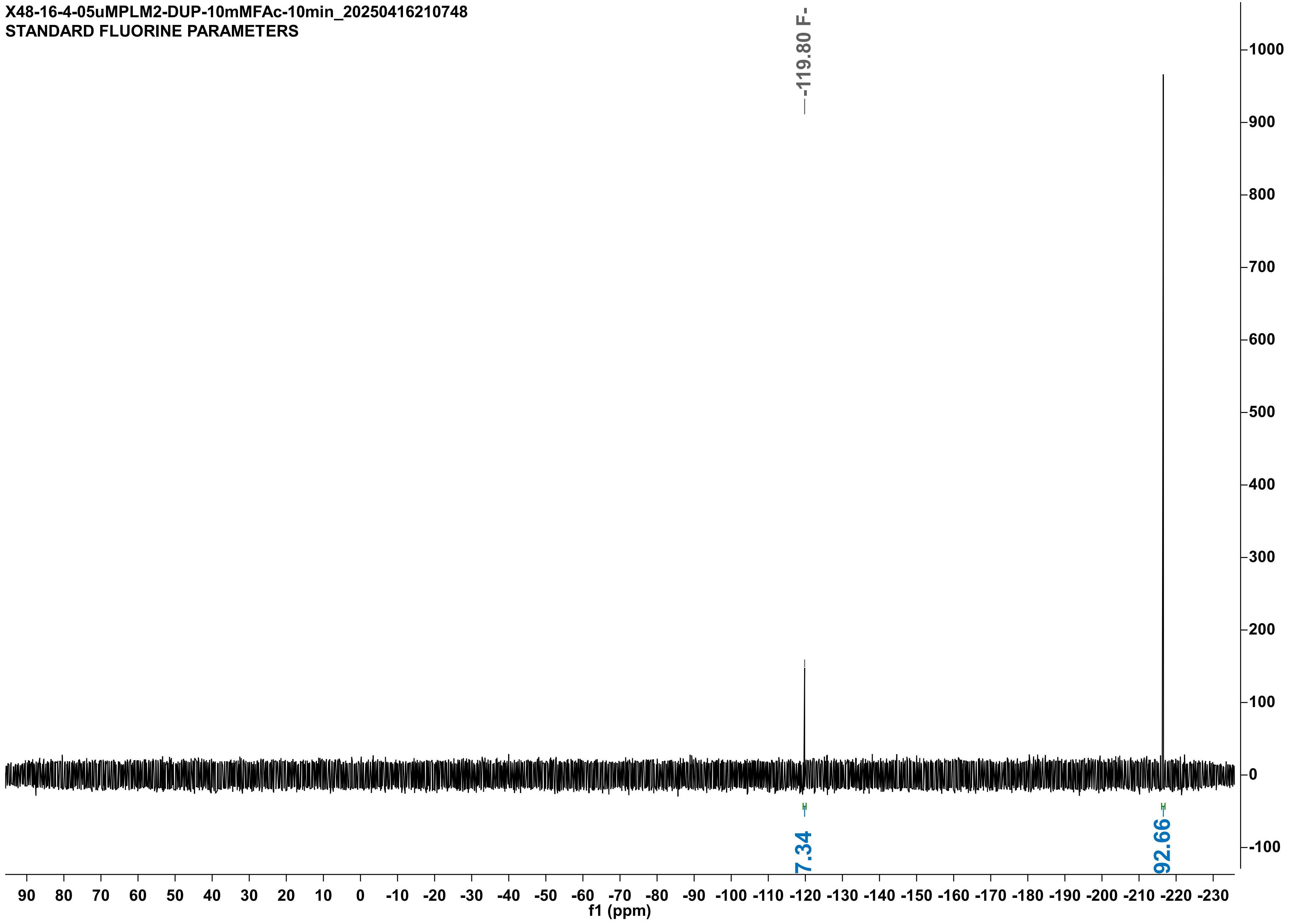

X48-16-4-05uMPLM2-DUP-10mMFAc-10min_20250416210748
STANDARD FLUORINE PARAMETERS
-119.80 F-
7.34
92.66
f1 (ppm)

X48-15-4-05uMLW--10mMFAc-2min_20250415162833
STANDARD FLUORINE PARAMETERS

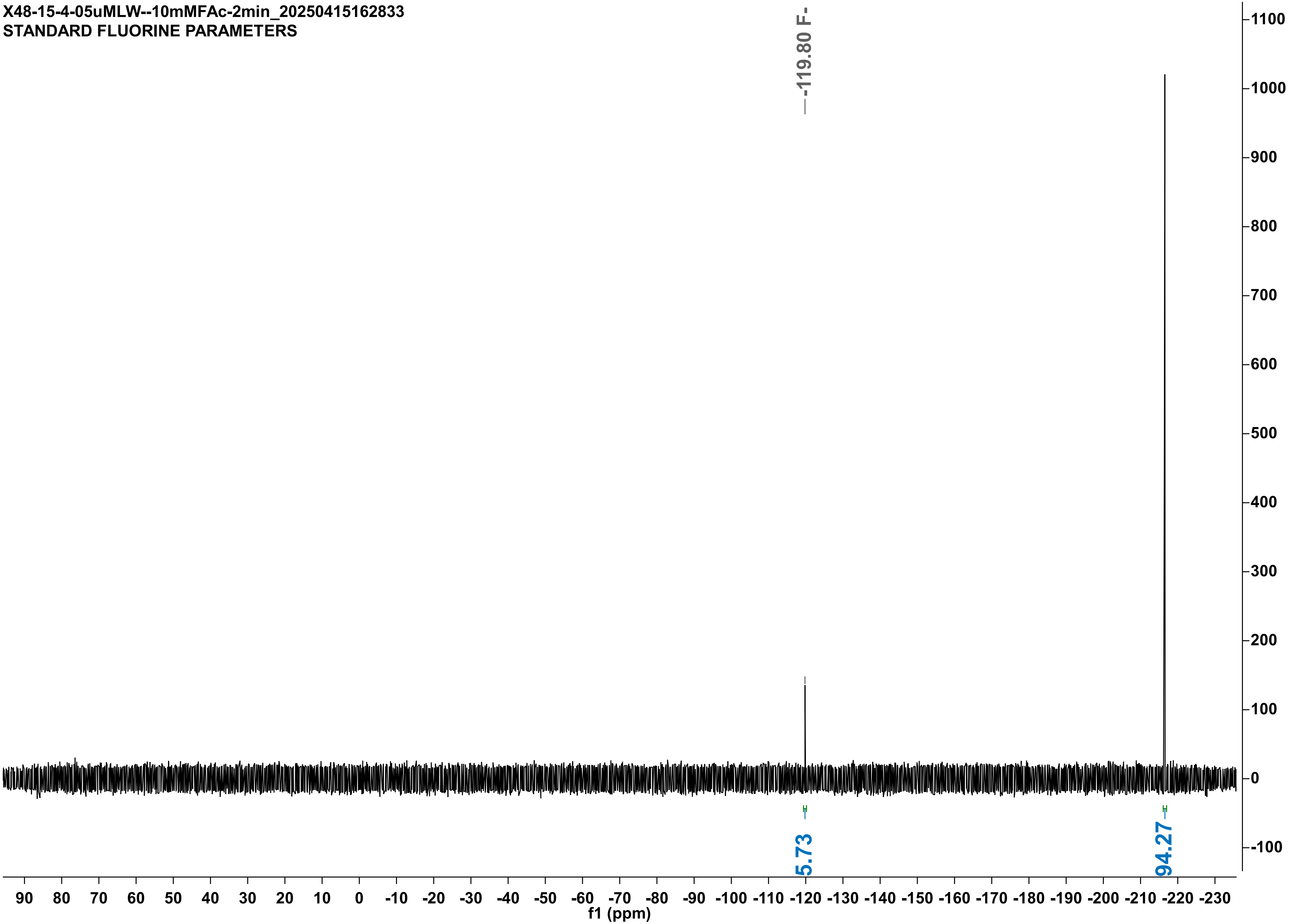

X48-15-4-05uMLW--DUPLO-10mMFAc-2min_20250415181716
STANDARD FLUORINE PARAMETERS

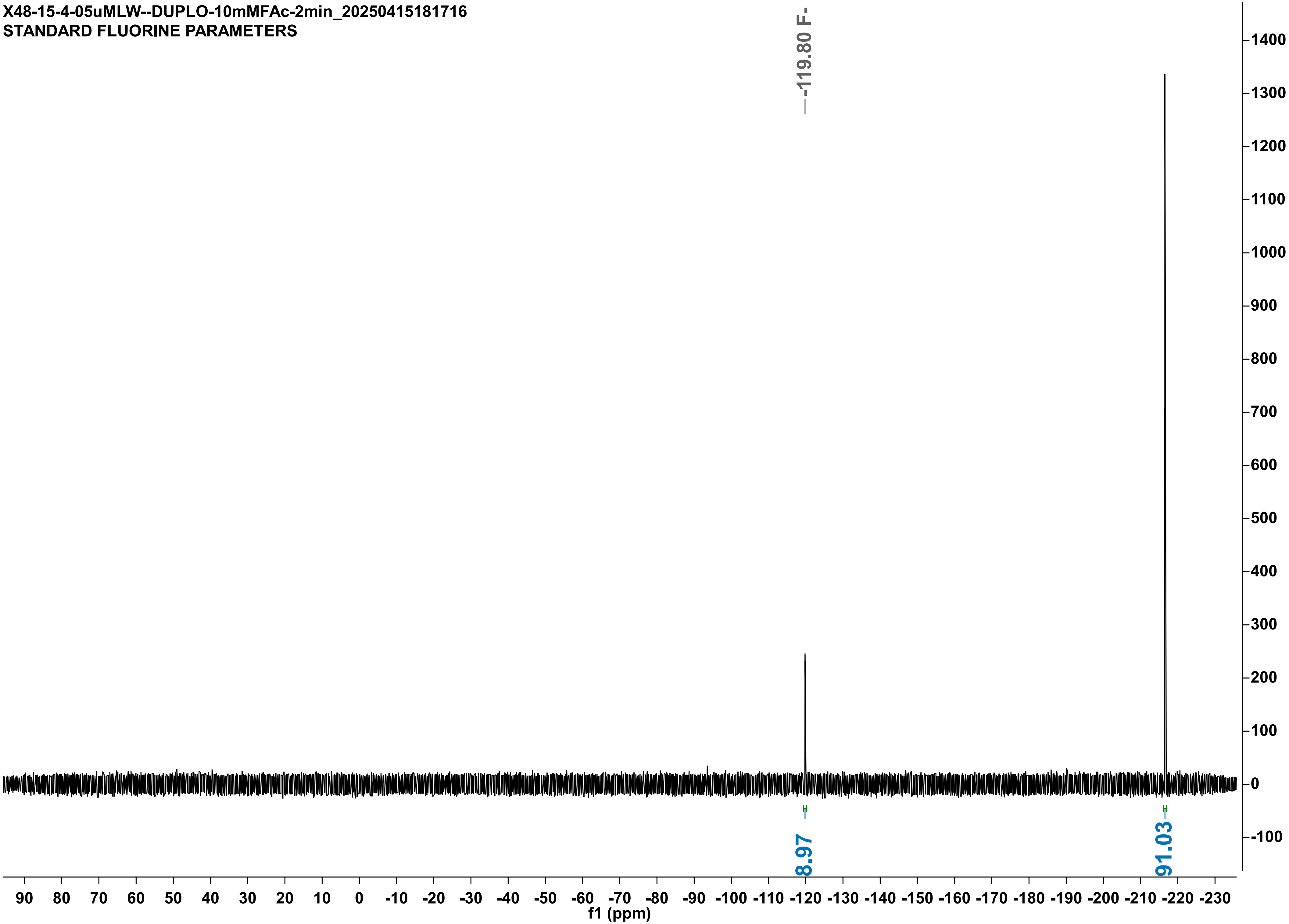

X48-16-4-05uMLVM-10mMFAc-45sec_20250416203054
STANDARD FLUORINE PARAMETERS

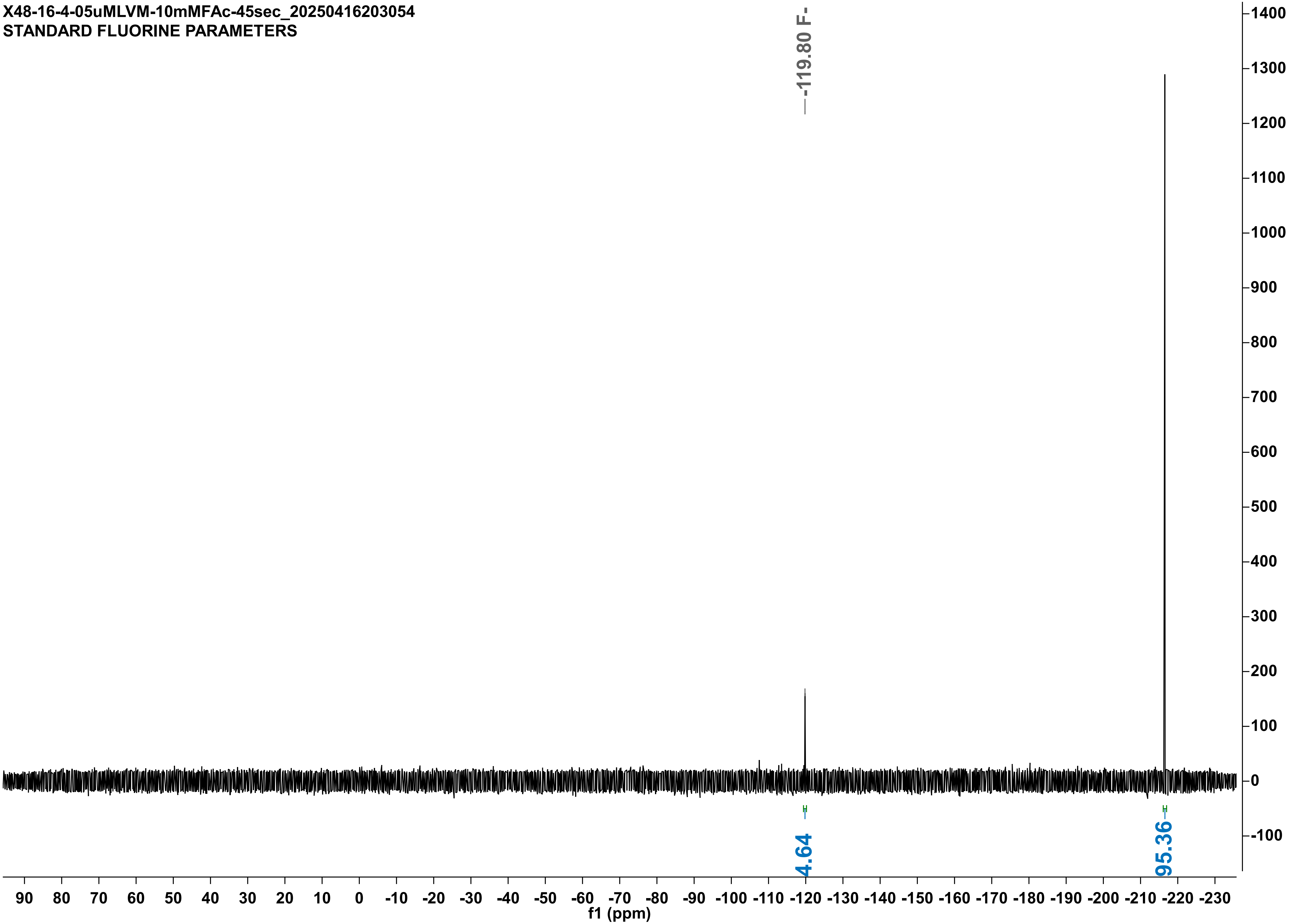

X48-16-4-05uMLVM-DUP-10mMFAc-45sec_20250416203114
STANDARD FLUORINE PARAMETERS

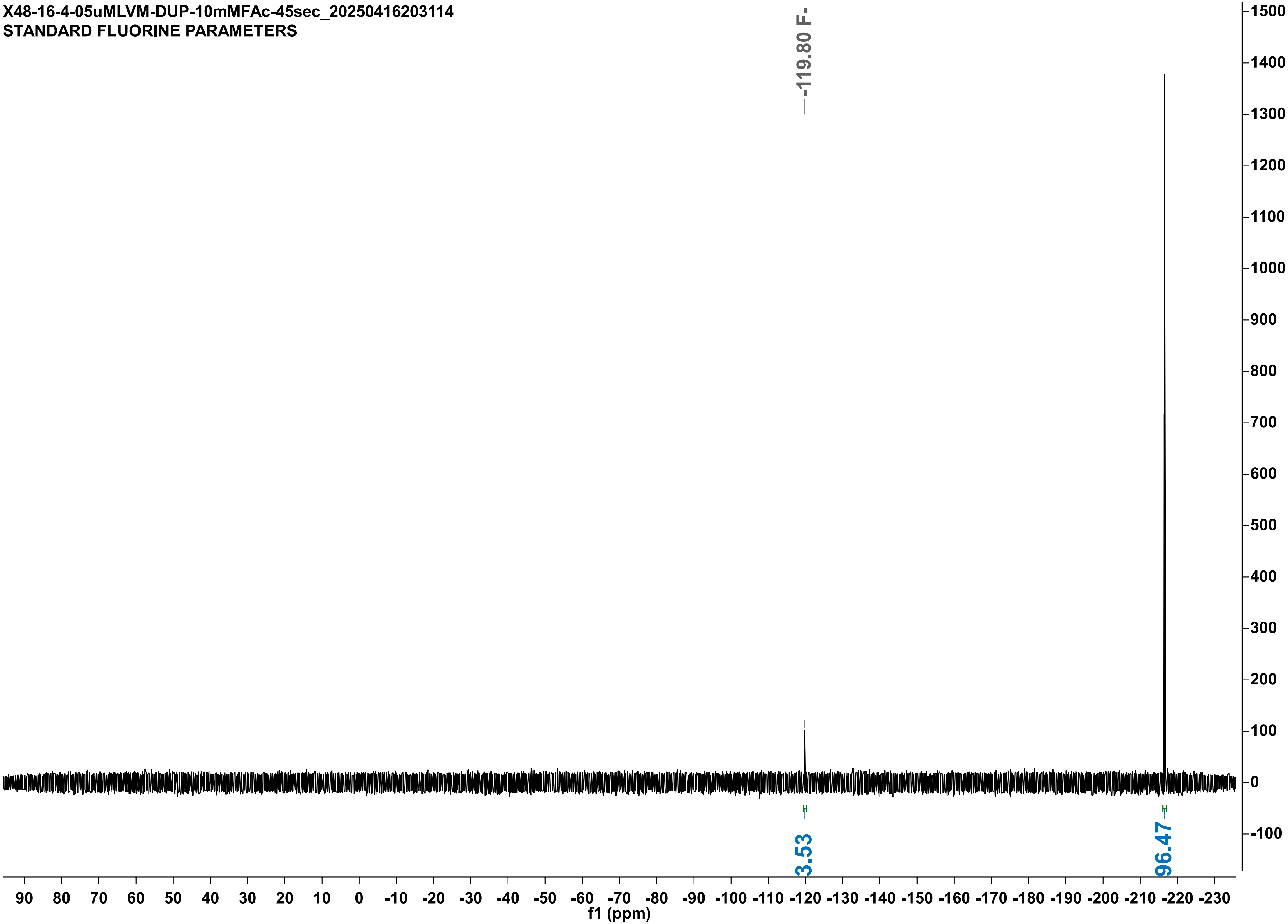

X48-16-4-05uM-FI-10mMFAc-45sec_20250416210706
STANDARD FLUORINE PARAMETERS

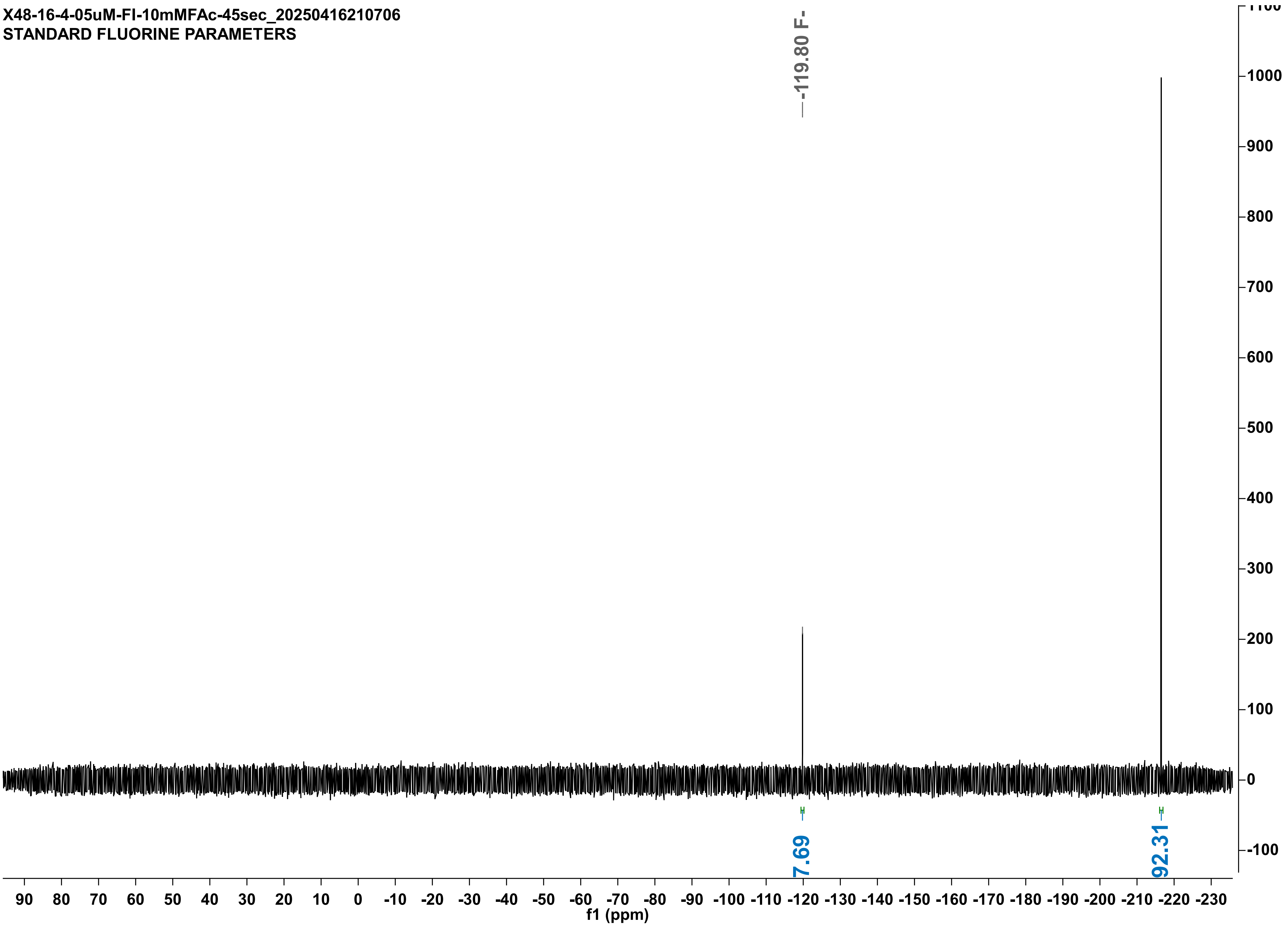

X48-16-4-05uM-FI-DUPLO-10mMFAc-45sec_20250416210716
STANDARD FLUORINE PARAMETERS

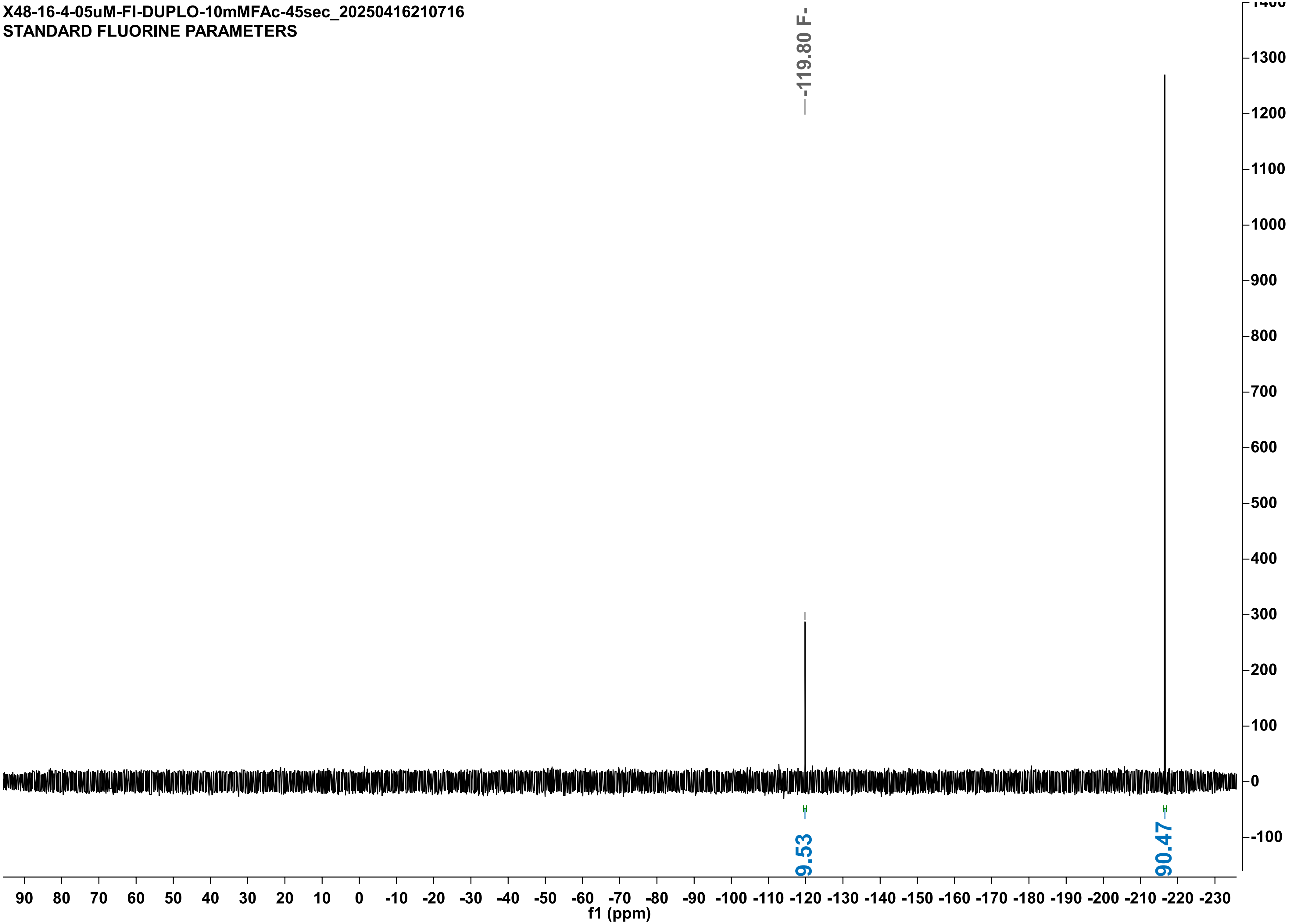

X46-9-4-NOENZ-10mMFPA-20min-noIS_20250409154323
STANDARD FLUORINE PARAMETERS

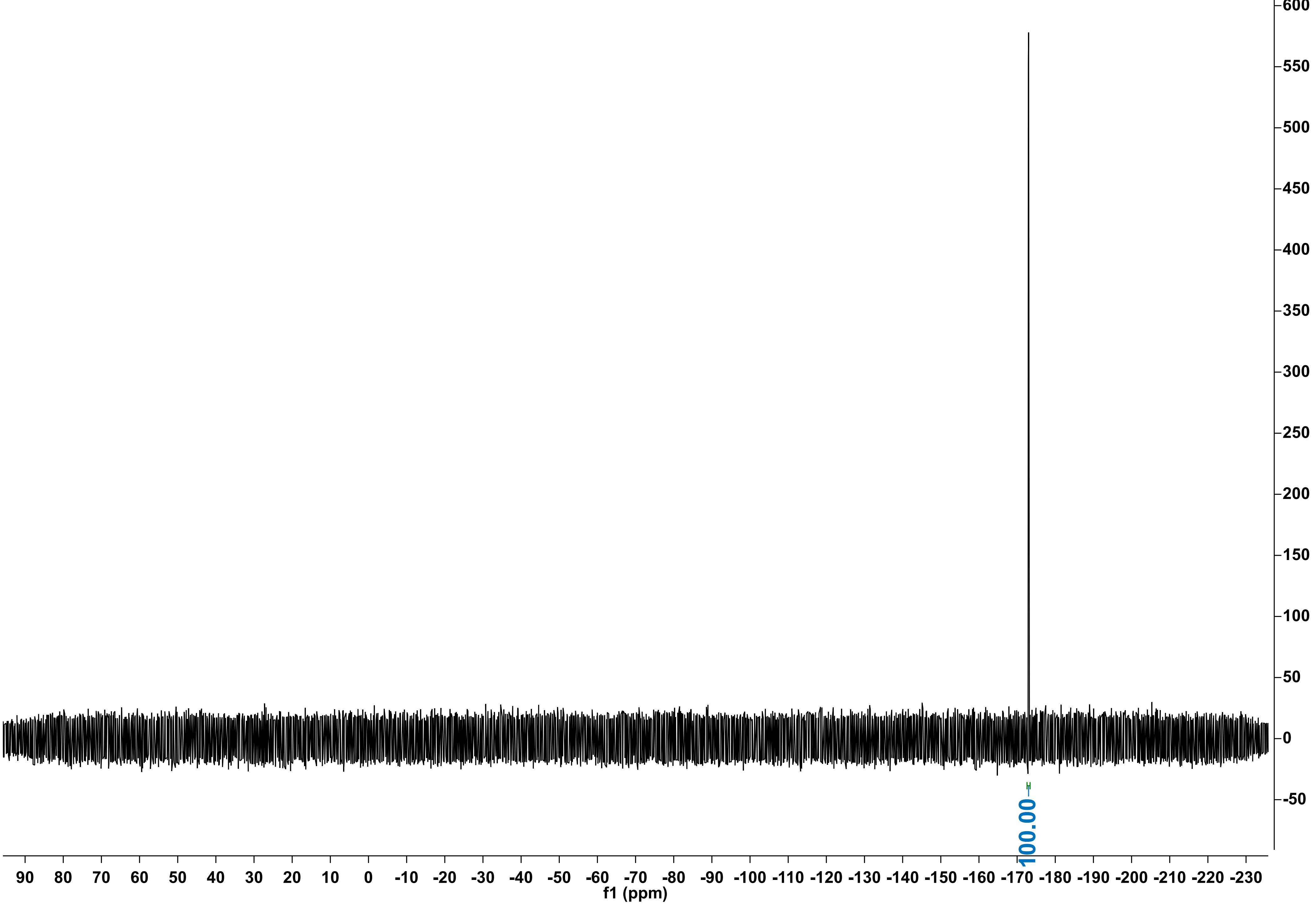

X46-9-4-05uMH272A-2-10mMFPA-20min-noIS_20250409154342
STANDARD FLUORINE PARAMETERS

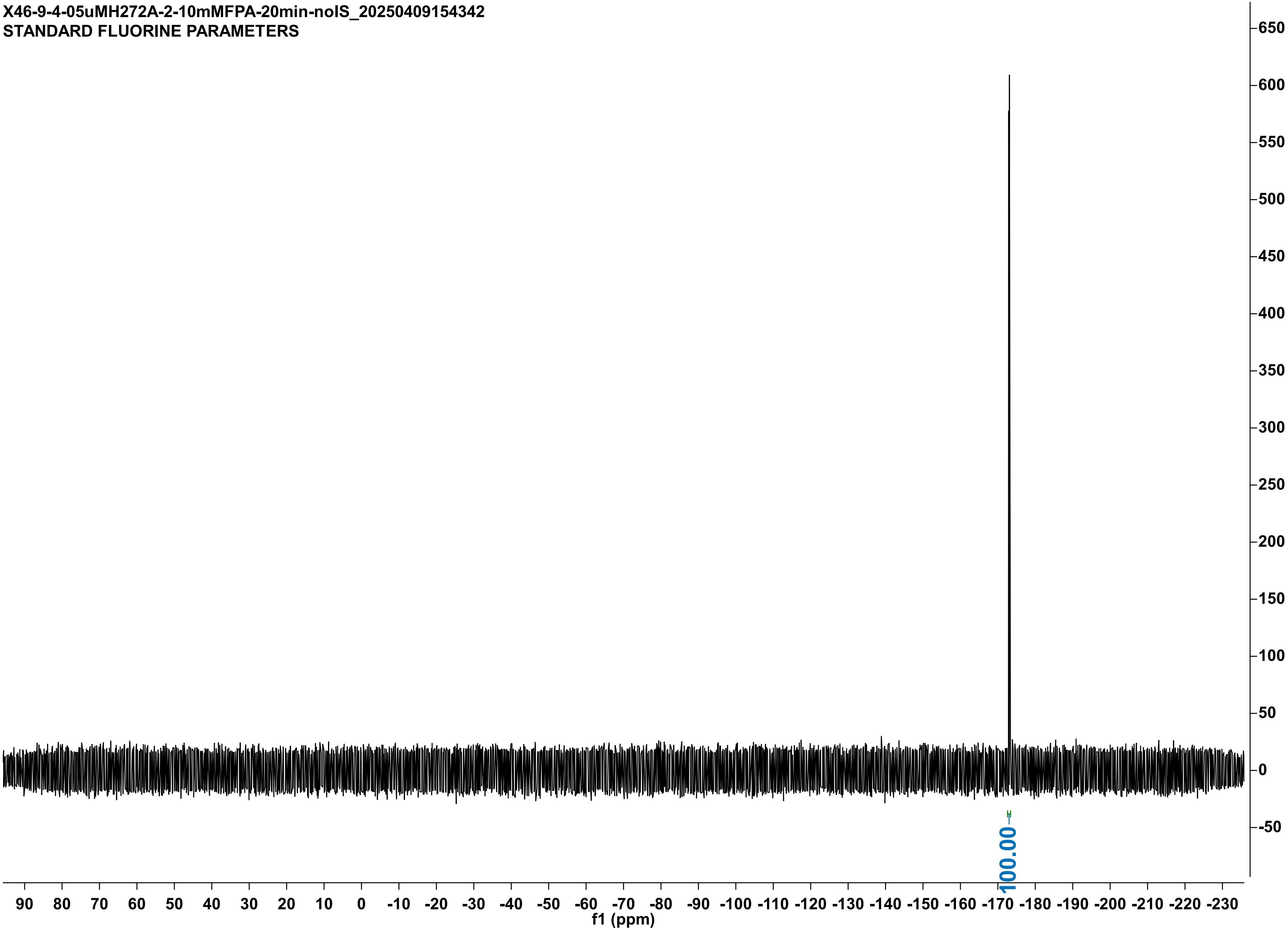

X48-11-4-05uMWT1-10mMFPA-10min_20250411170910
STANDARD FLUORINE PARAMETERS

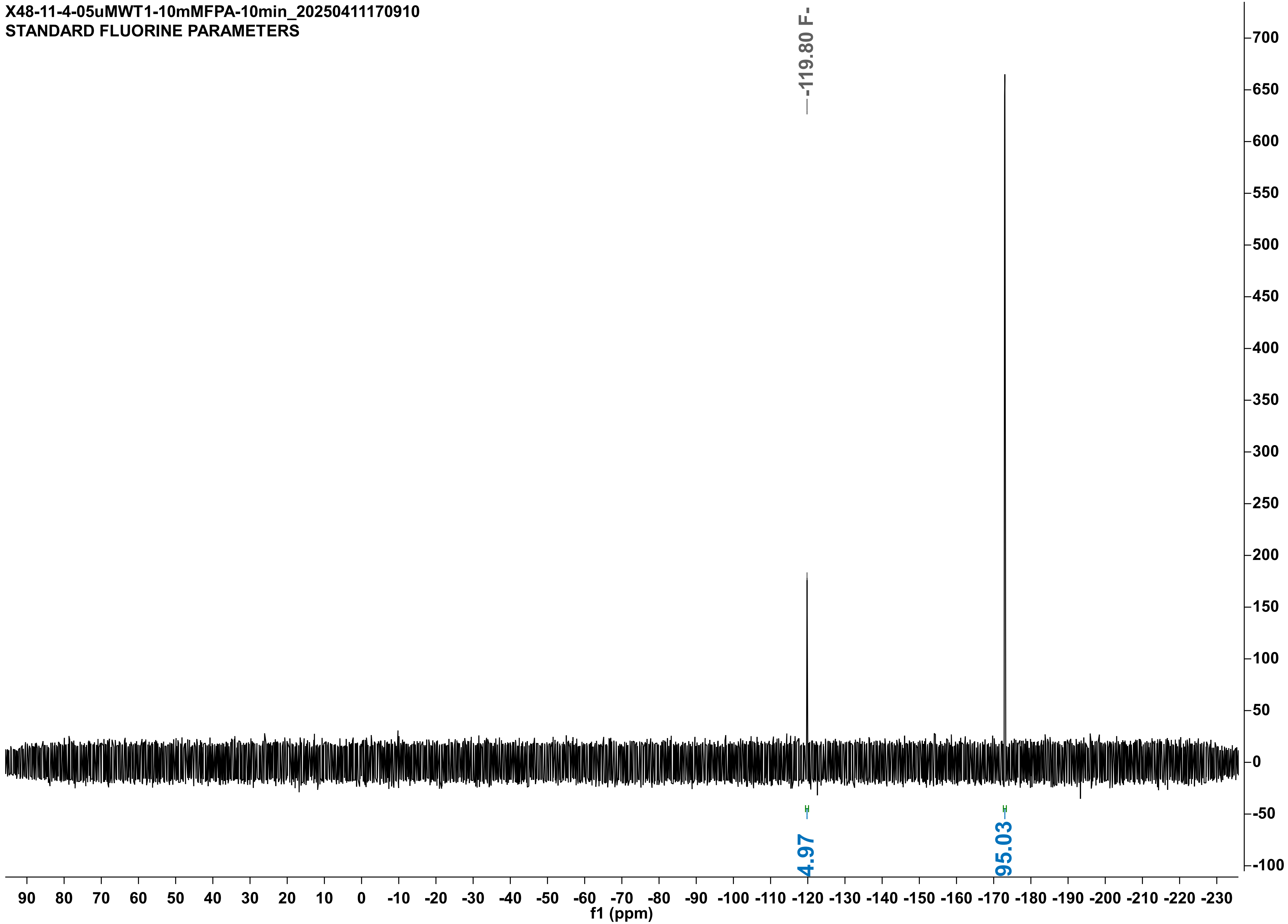

X48-11-4-05uMWT2-10mMFPA-10min_20250411170920
STANDARD FLUORINE PARAMETERS

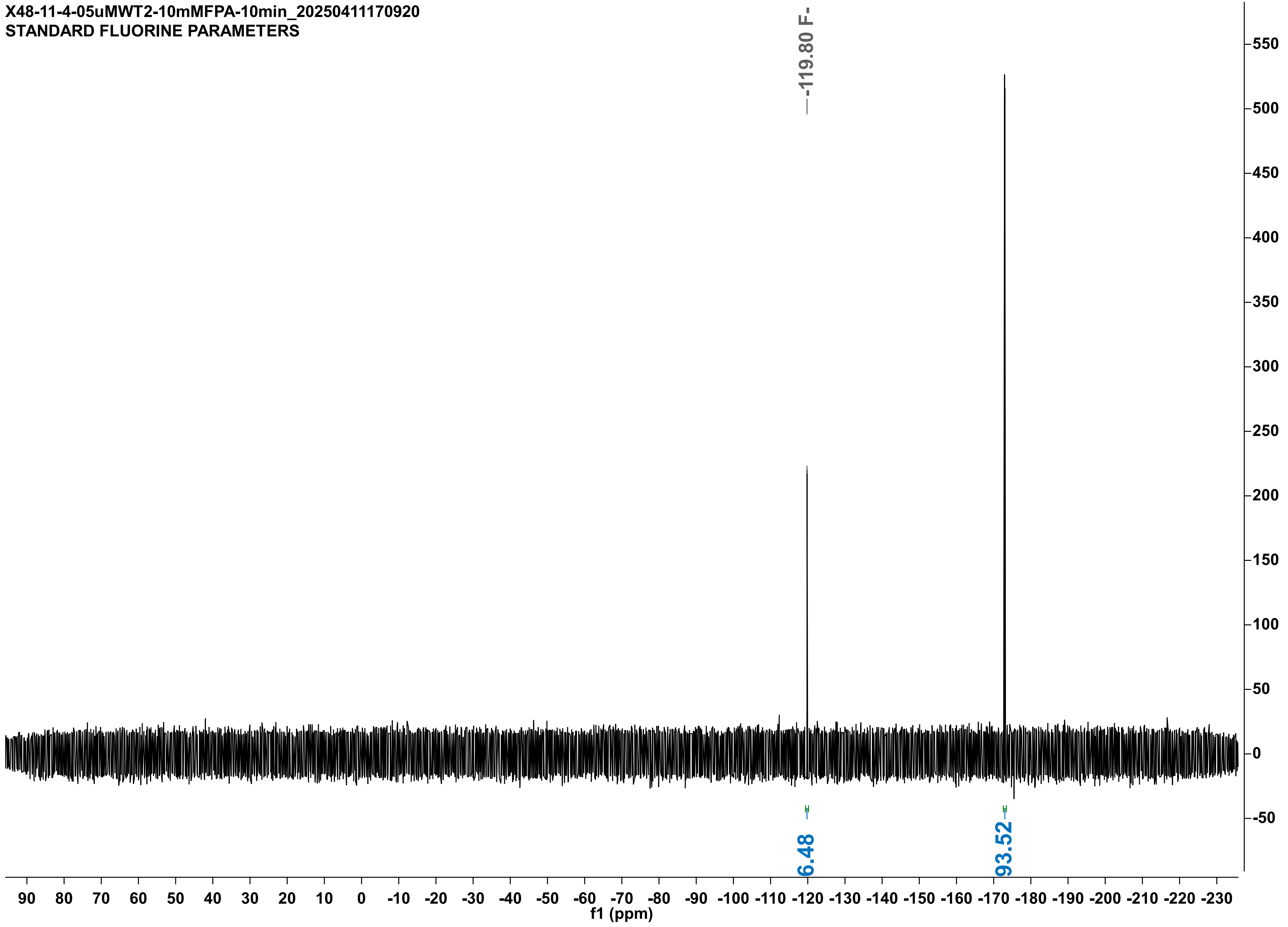

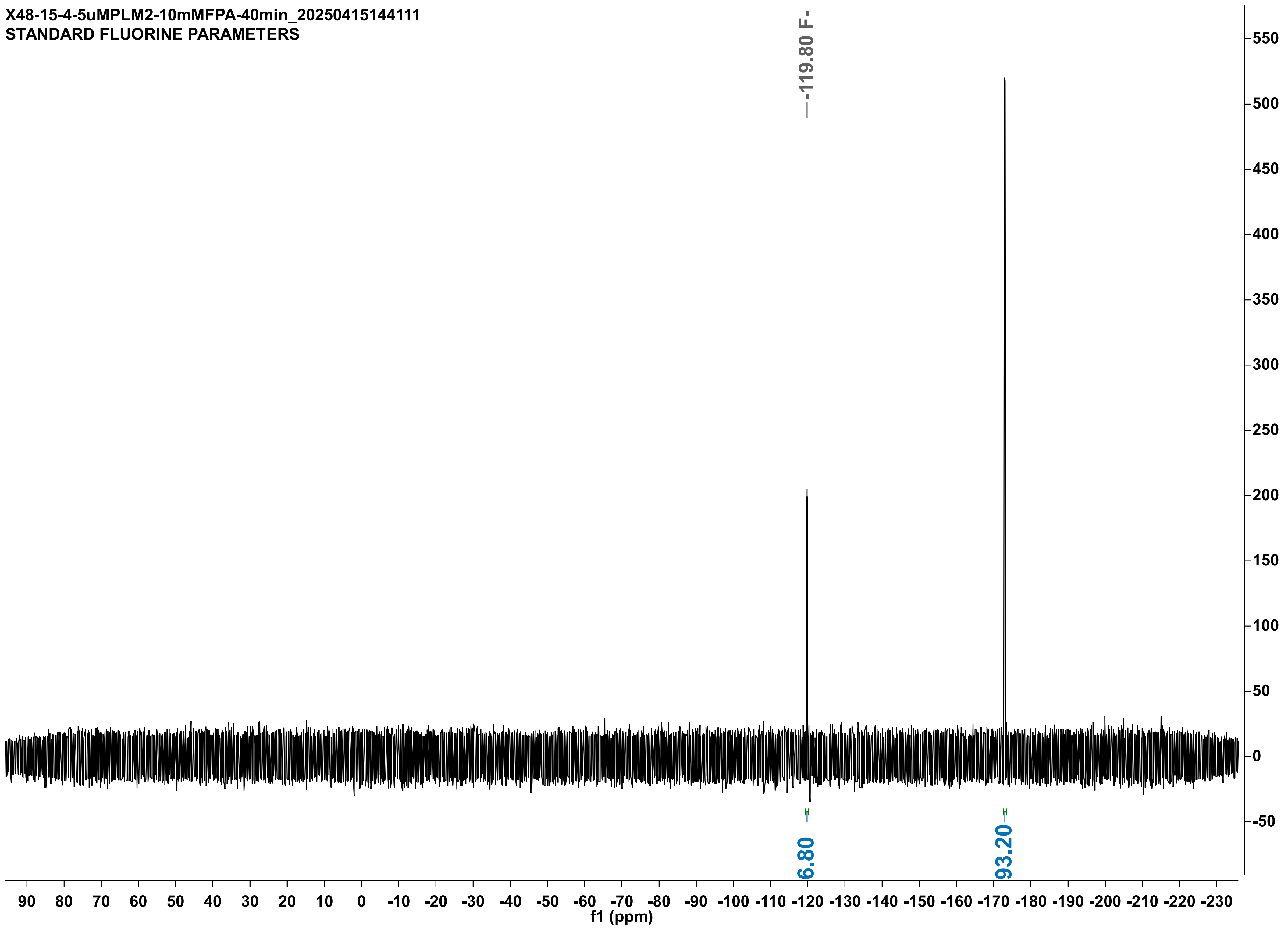

X48-15-4-5uMPLM2-10mMFPA-40min_20250415144111
STANDARD FLUORINE PARAMETERS
-119.80 F-
6.80
93.20
f1 (ppm)

X48-15-4-5uMPLM2-duplo-10mMFPA-40min_20250415154332
STANDARD FLUORINE PARAMETERS

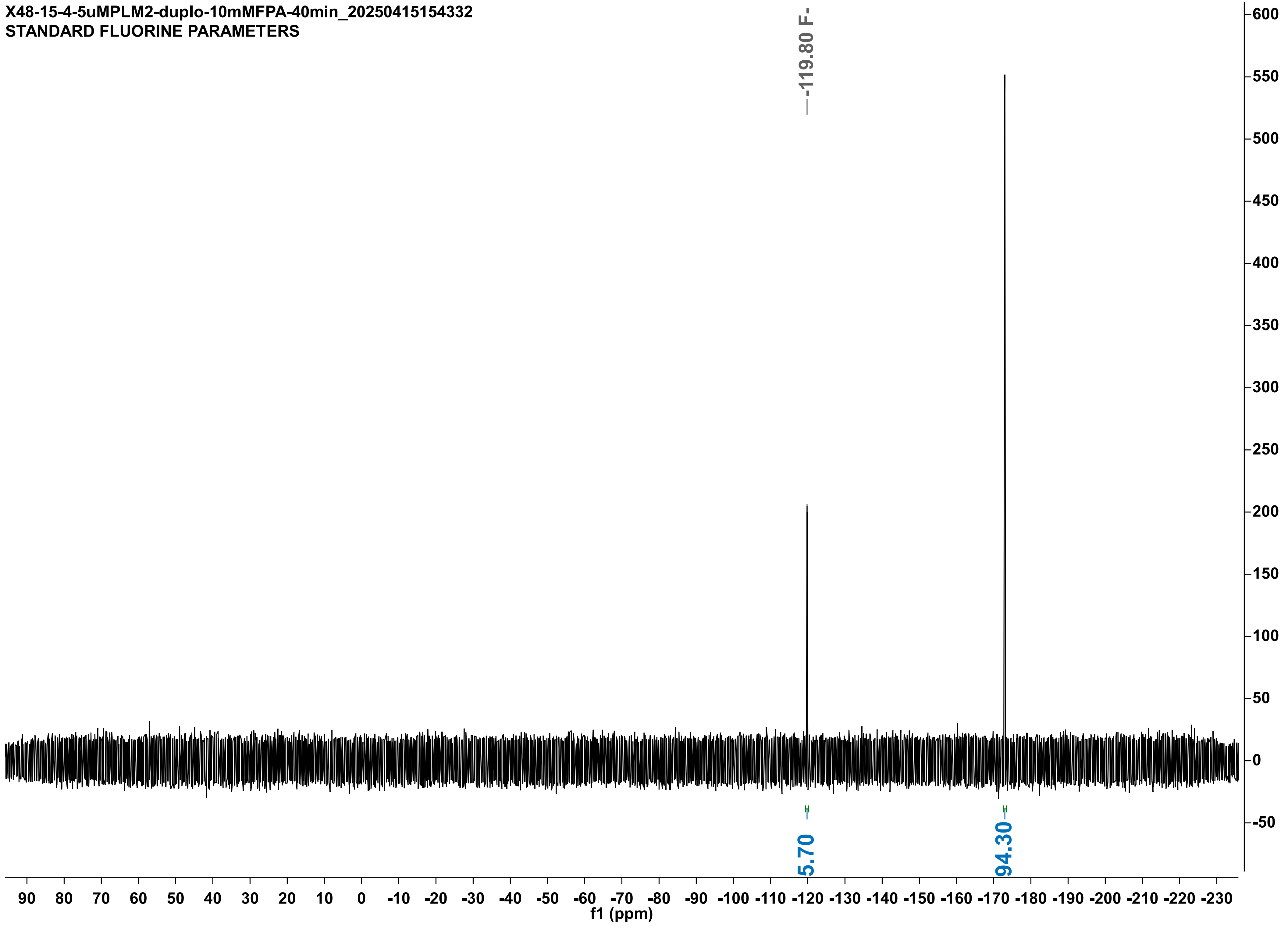

X48-15-4-5uMRPL-10mMFPA-2h30min_20250415151629
STANDARD FLUORINE PARAMETERS

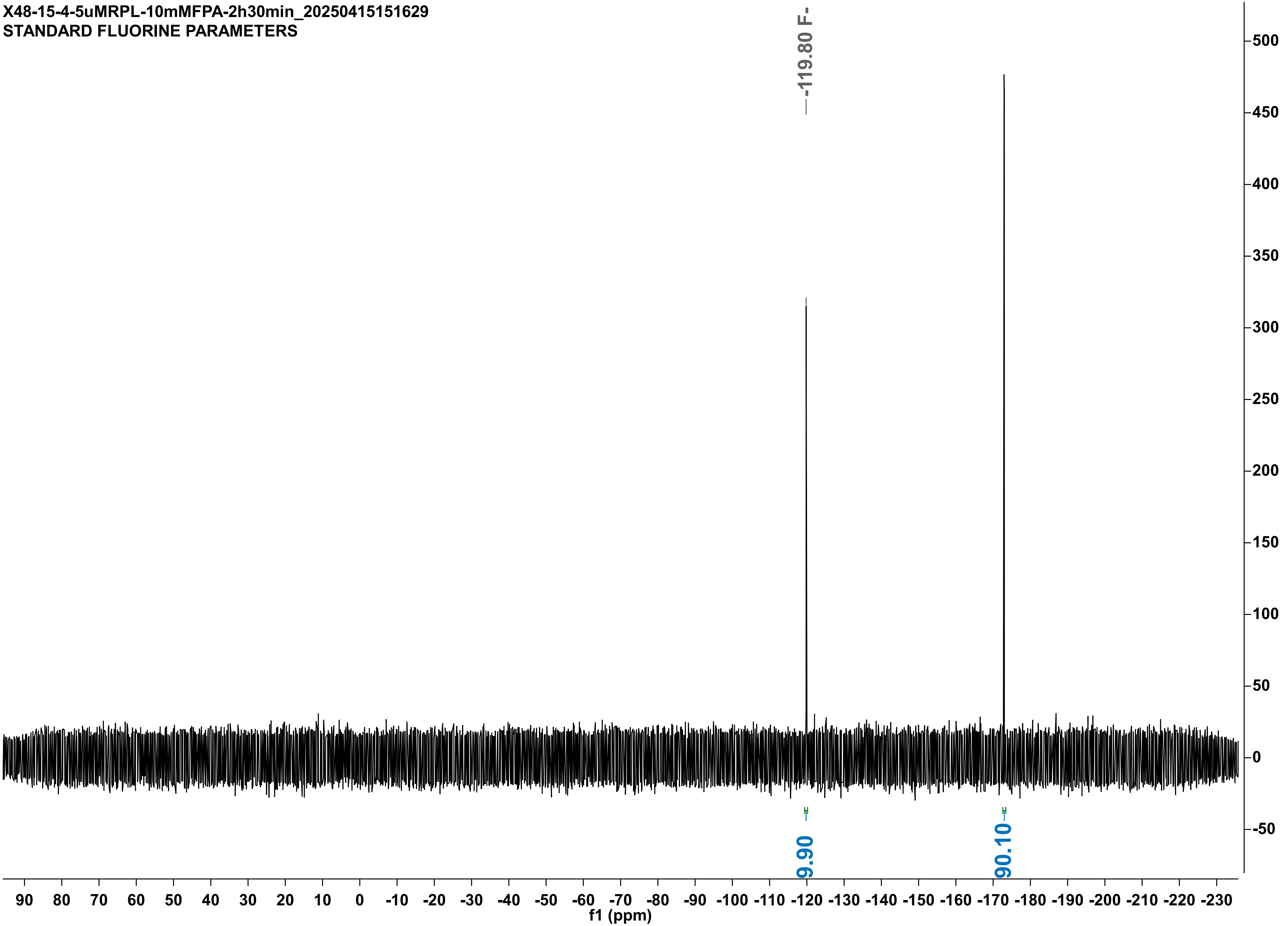

X48-15-4-5uMRPL-DUPLO-10mMFPA-2h30min_20250415174344
STANDARD FLUORINE PARAMETERS

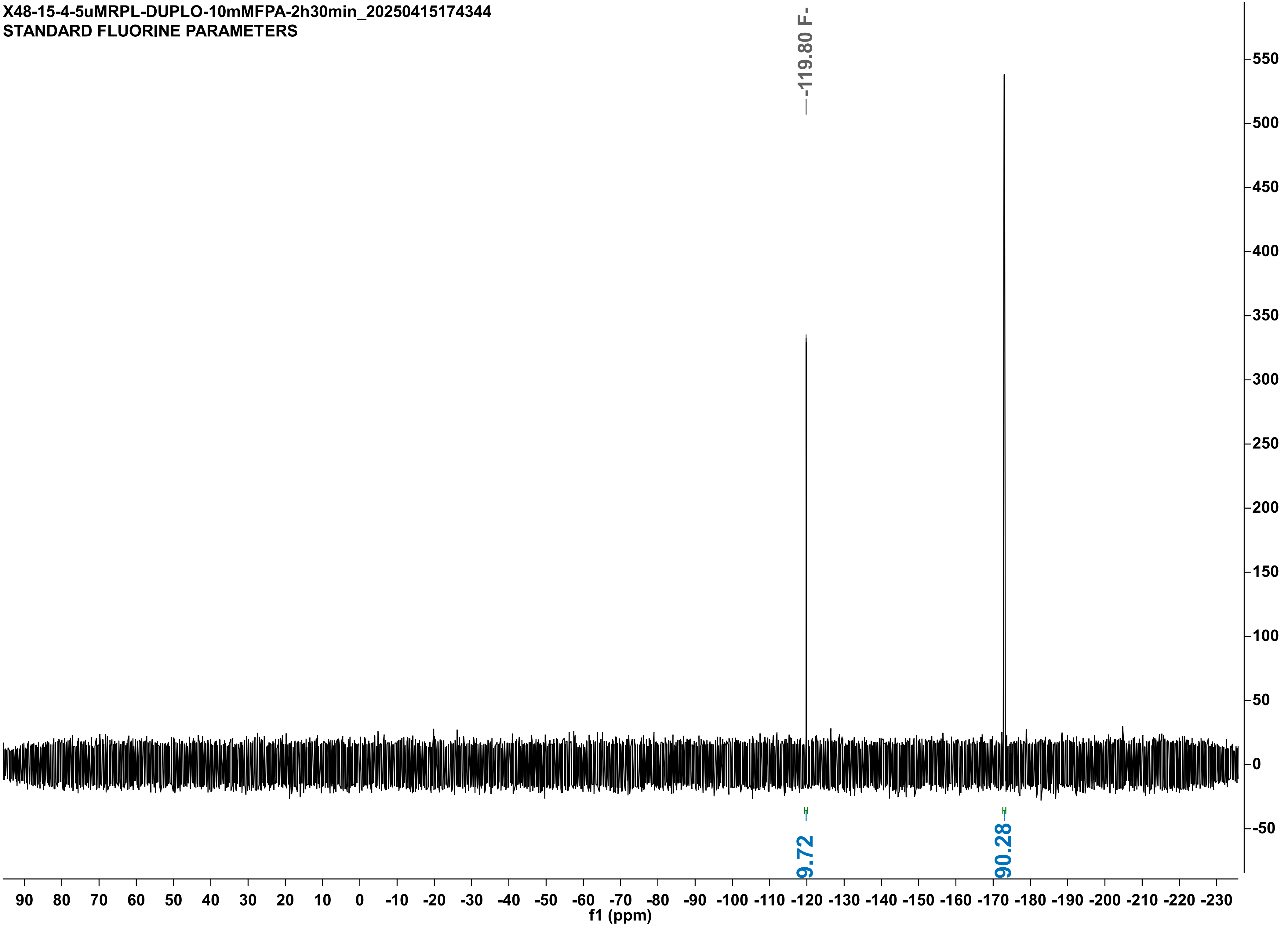

X48-11-4-05uMVW--10mMFPA-30min_20250411154817
STANDARD FLUORINE PARAMETERS

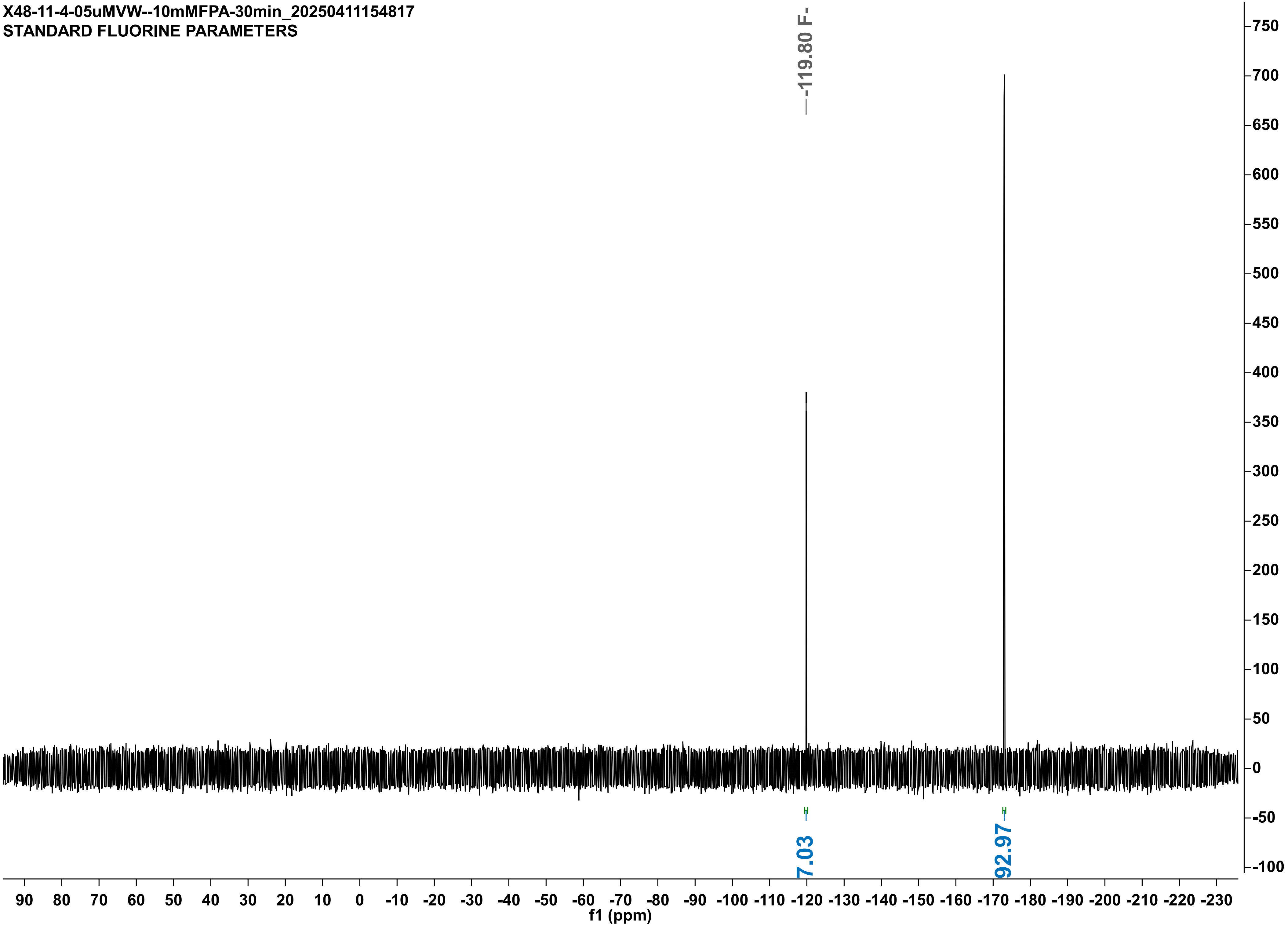

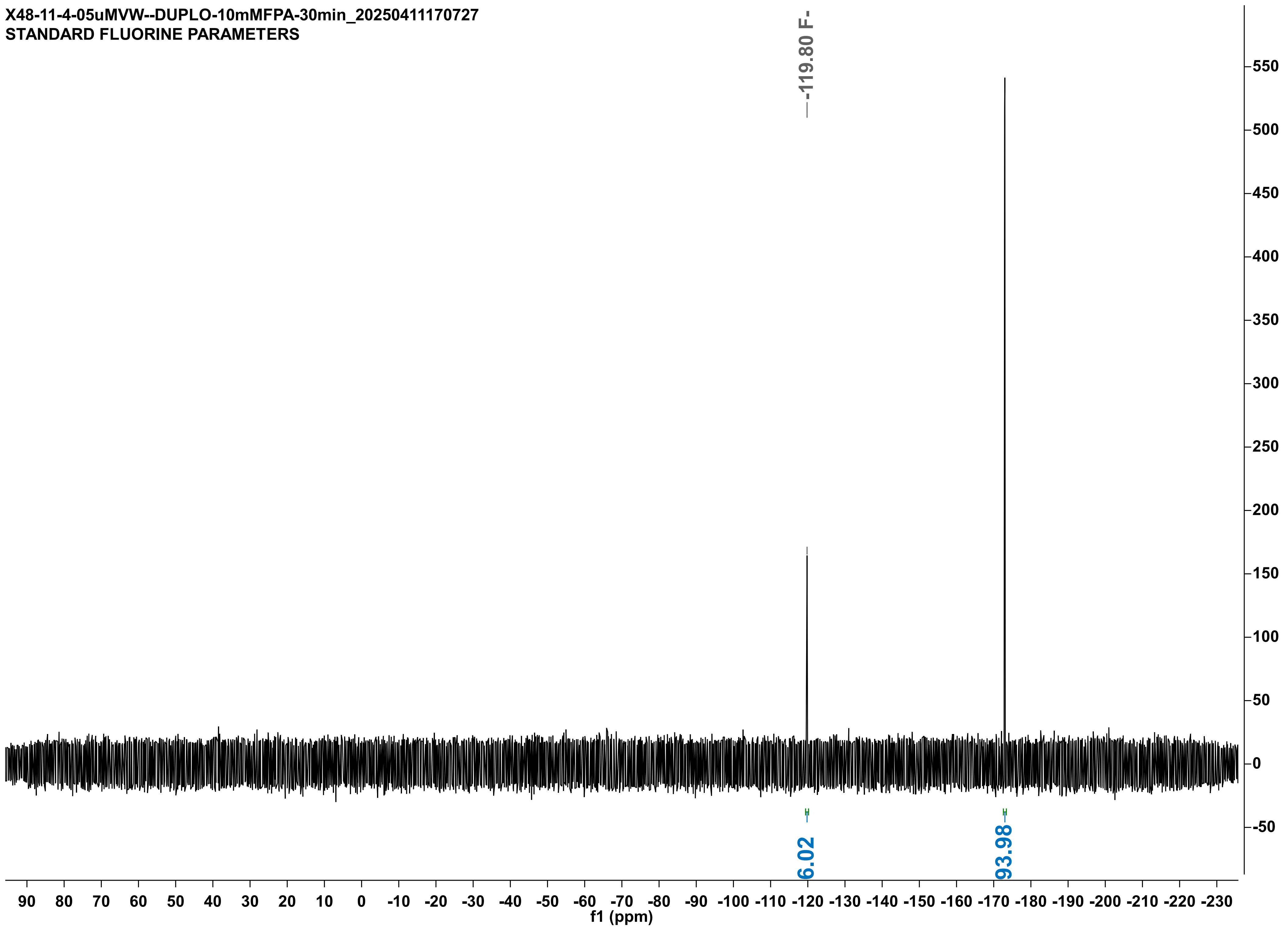
X48-11-4-05uMVW--DUPLO-10mMFPA-30min_20250411170727
STANDARD FLUORINE PARAMETERS
-119.80 F-
6.02
93.98
f1 (ppm)

X48-16-4-05uM-VM-DUP-10mMFPA-15min_20250416184333
STANDARD FLUORINE PARAMETERS

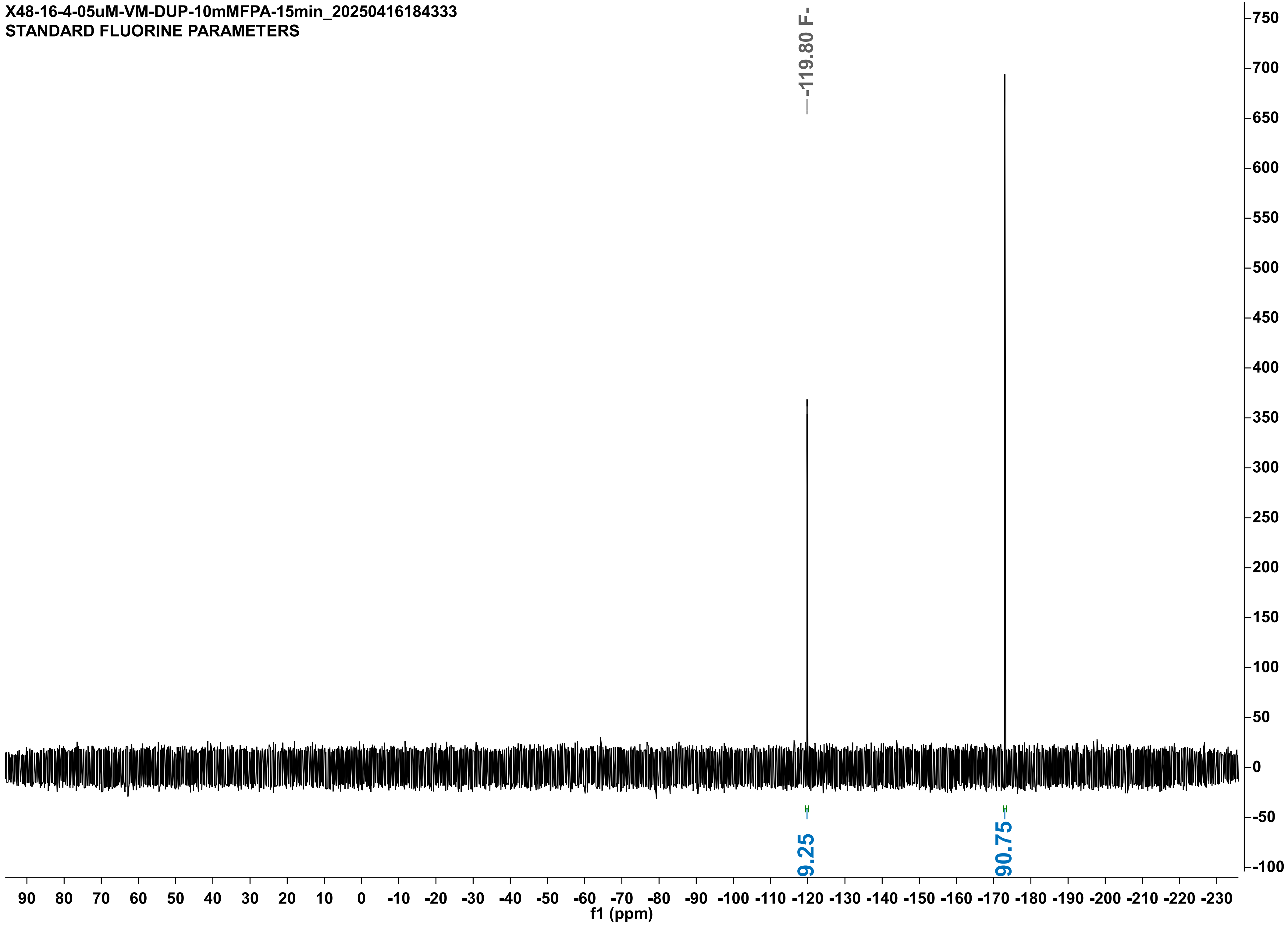

X48-16-4-05uM-VM-10mMFPA-15min_20250416184332
STANDARD FLUORINE PARAMETERS

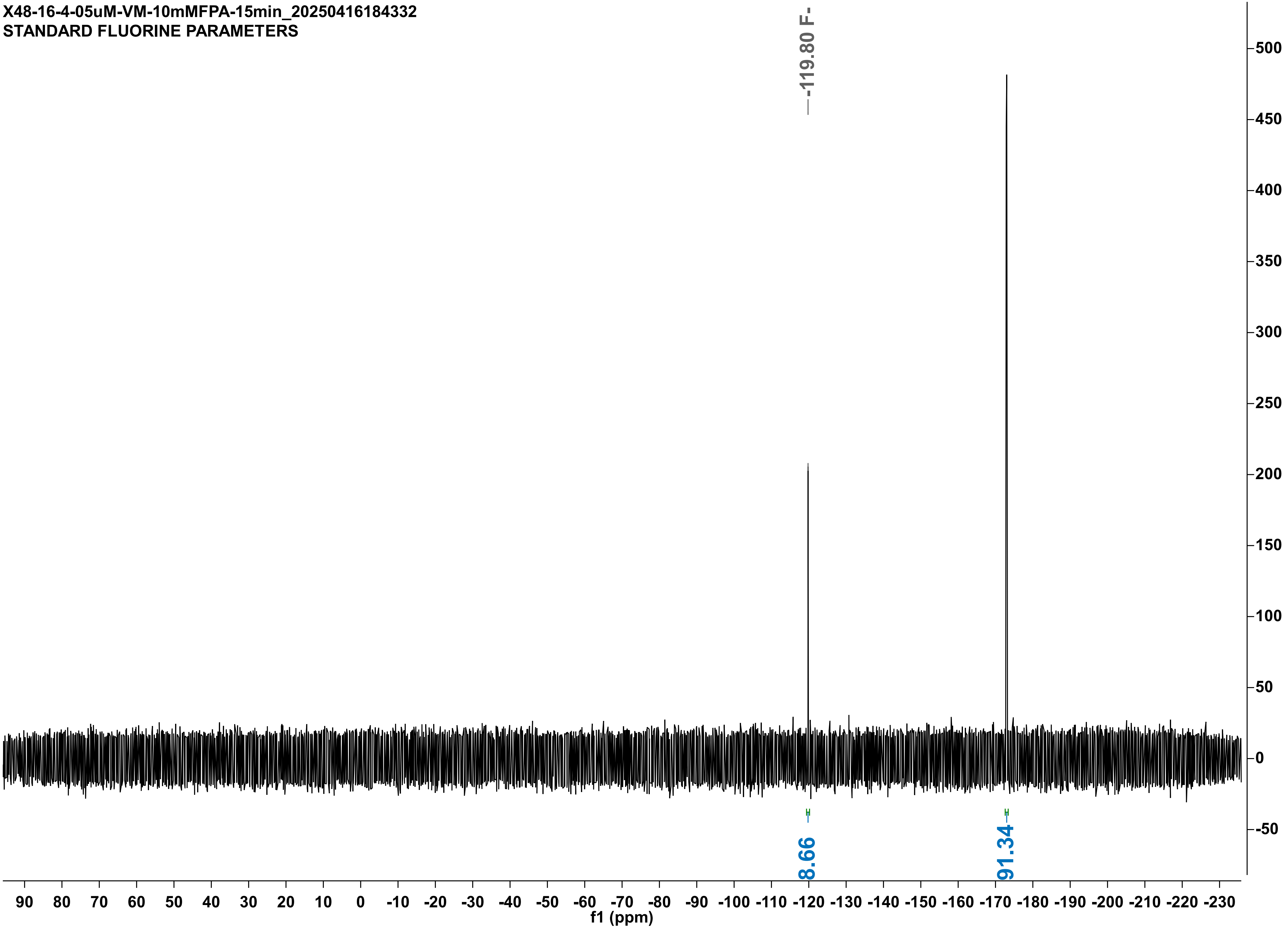

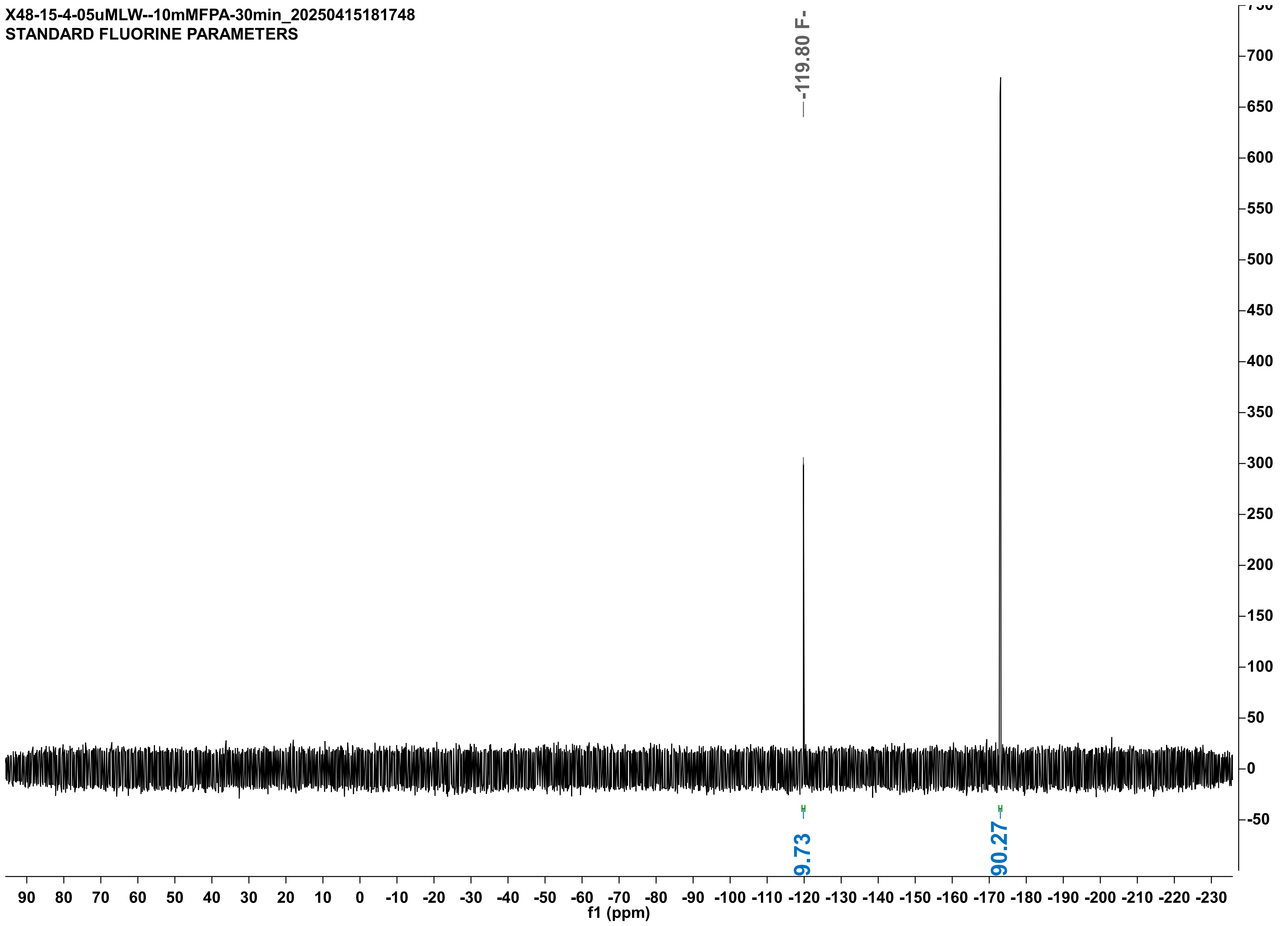
X48-15-4-05uMLW--10mMFPA-30min_20250415181748
STANDARD FLUORINE PARAMETERS
-119.80 F-
9.73
90.27
f1 (ppm)
90 80 70 60 50 40 30 20 10 0 -10 -20 -30 -40 -50 -60 -70 -80 -90 -100 -110 -120 -130 -140 -150 -160 -170 -180 -190 -200 -210 -220 -230
750 700 650 600 550 500 450 400 350 300 250 200 150 100 50 0 -50

X48-16-4-05uMLW--10mMFPA-30min_20250416202647
STANDARD FLUORINE PARAMETERS

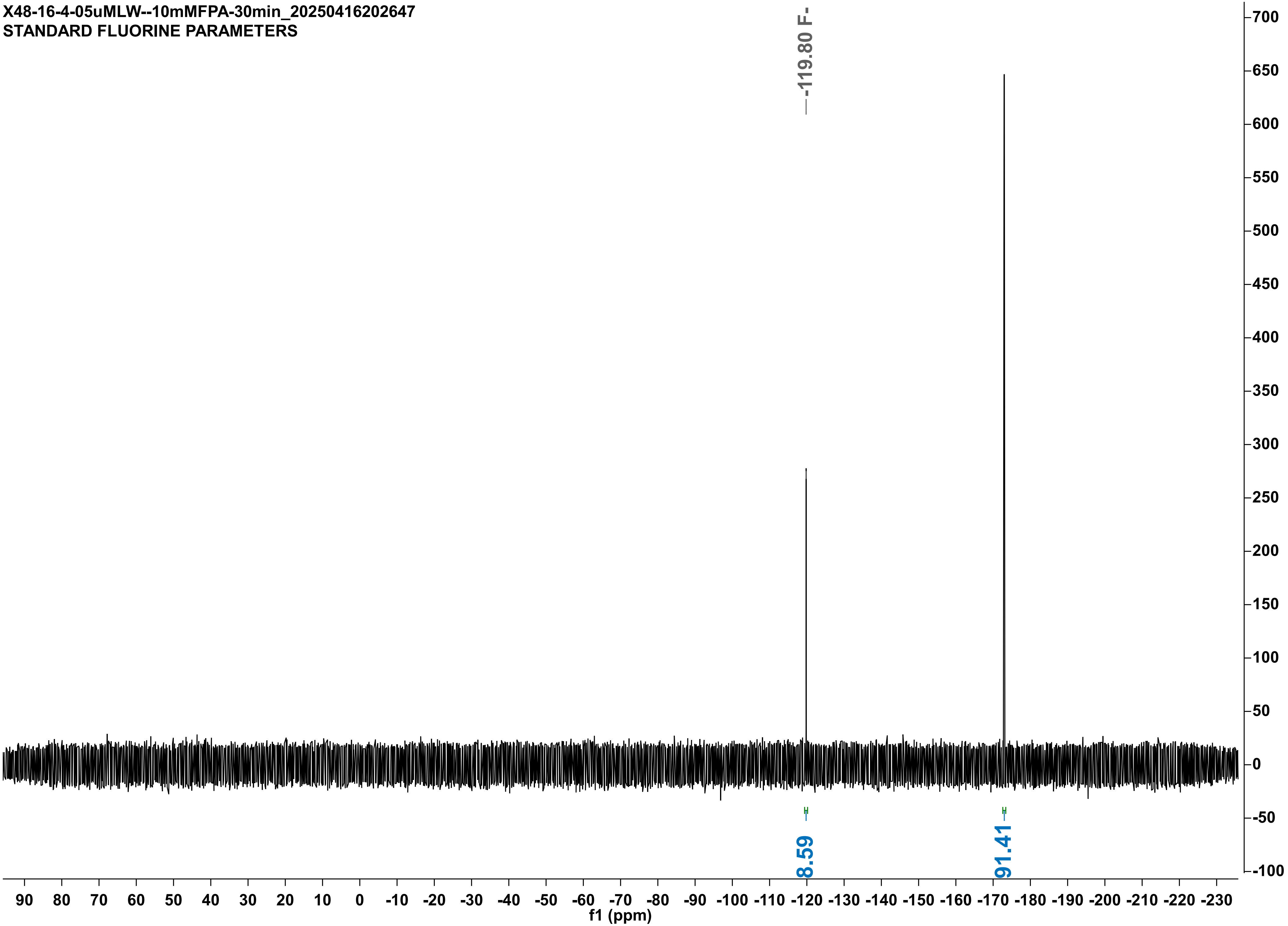

X48-16-4-05uMRLM-10mMFPA-25min_20250416203025
STANDARD FLUORINE PARAMETERS

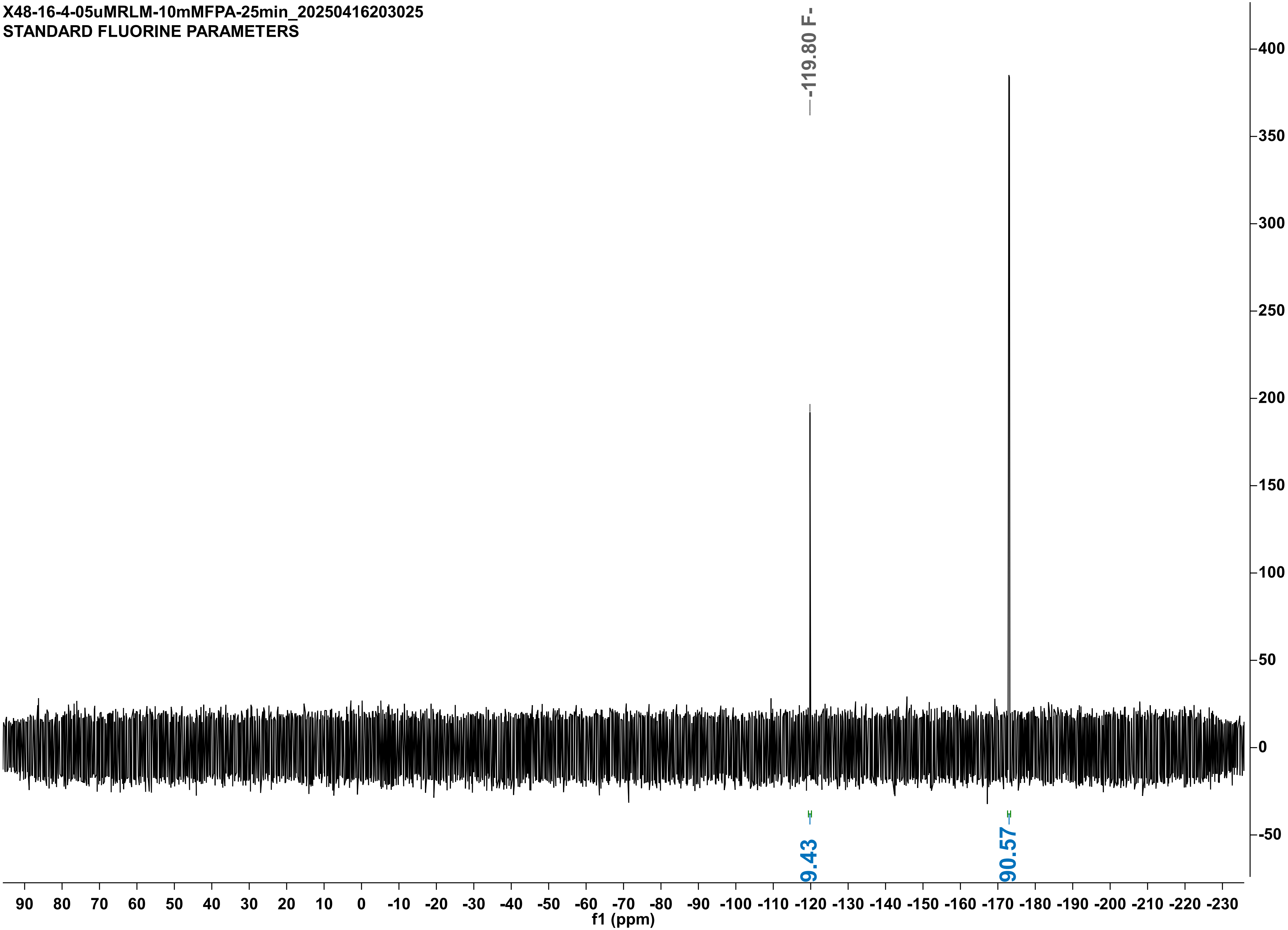

X48-16-4-05uMRLM-DUP-10mMFPA-25min_20250416203039
STANDARD FLUORINE PARAMETERS

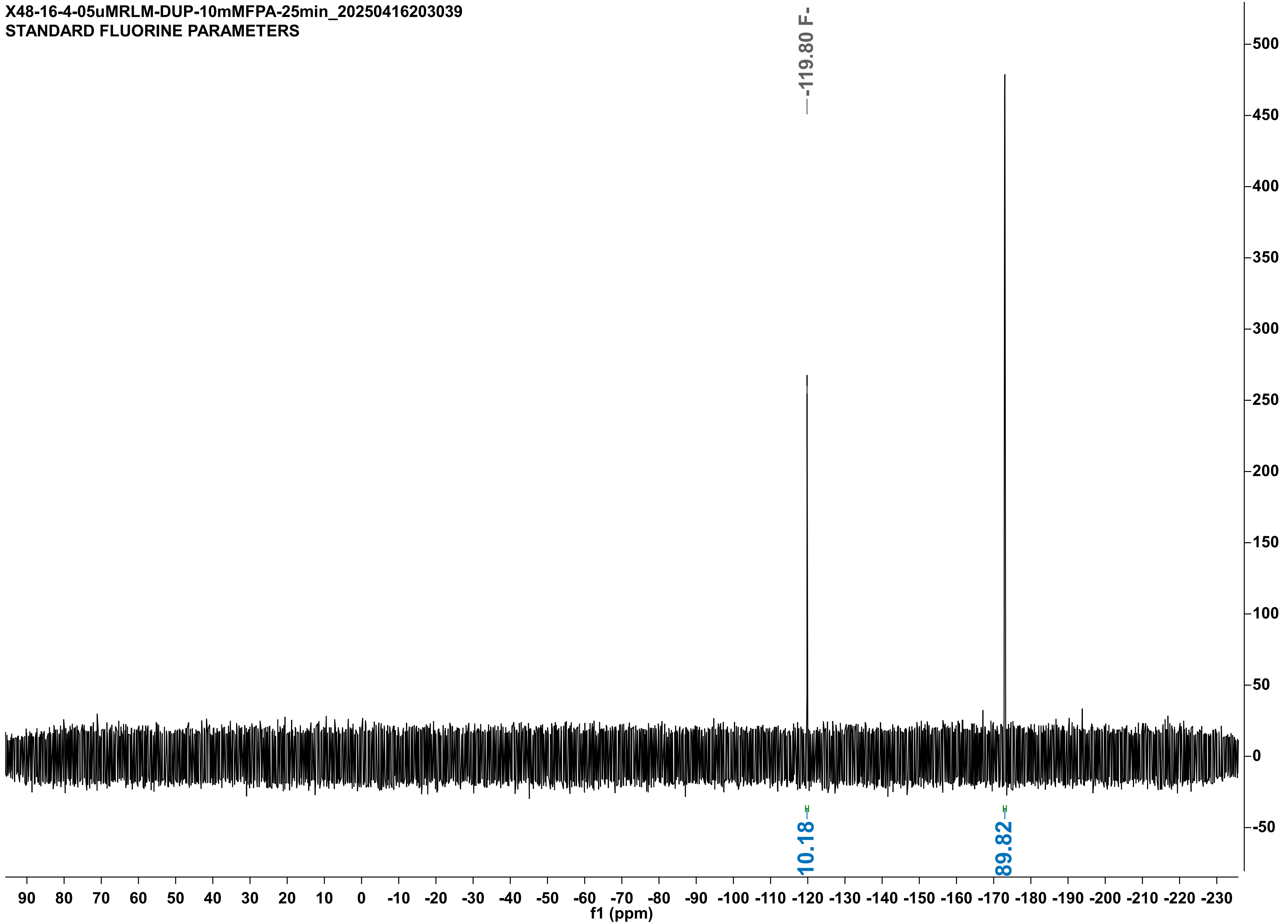

X48-15-4-5uMRHL-DUPLO-10mMFPA-90min_20250415174520
STANDARD FLUORINE PARAMETERS

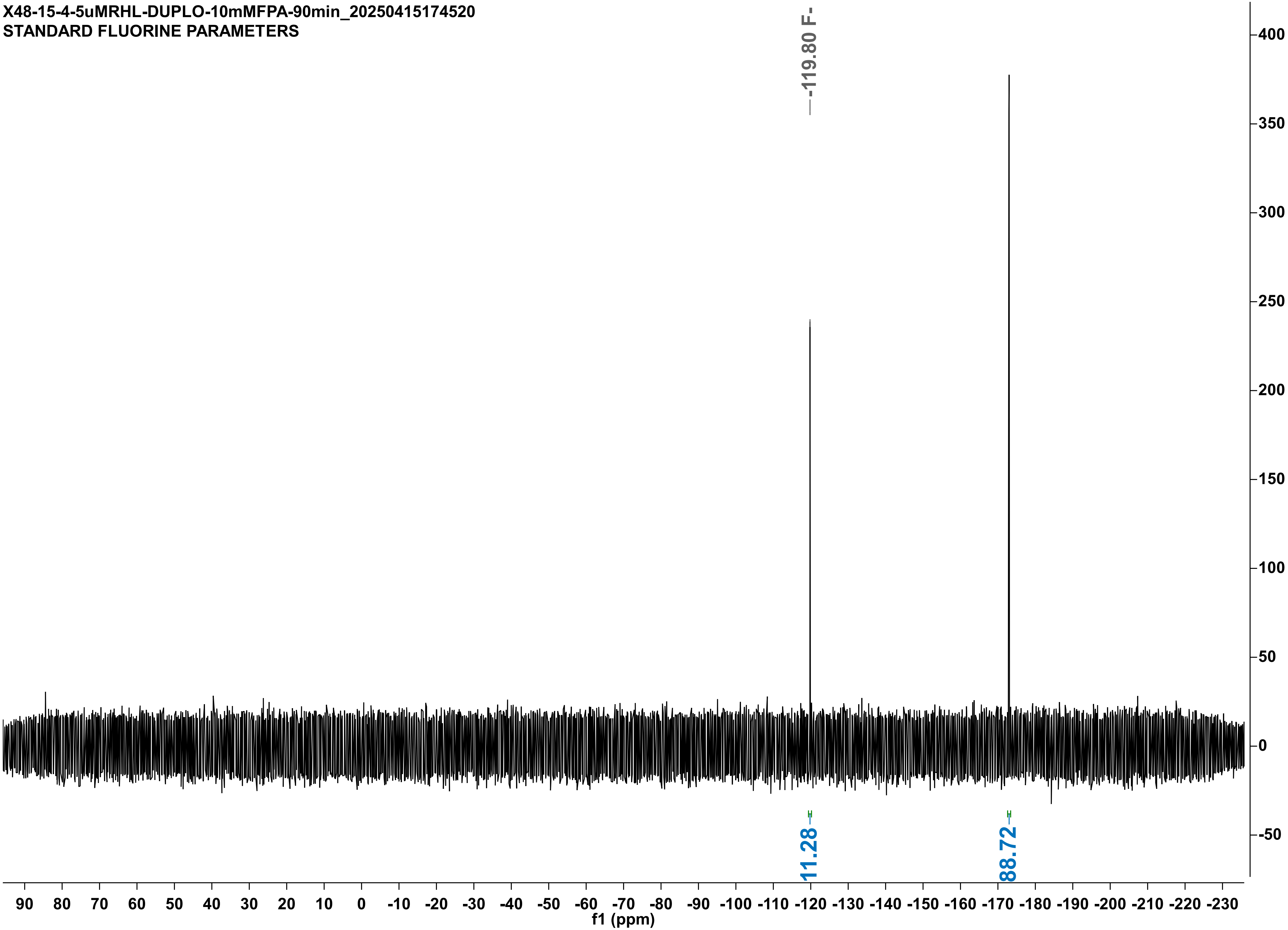

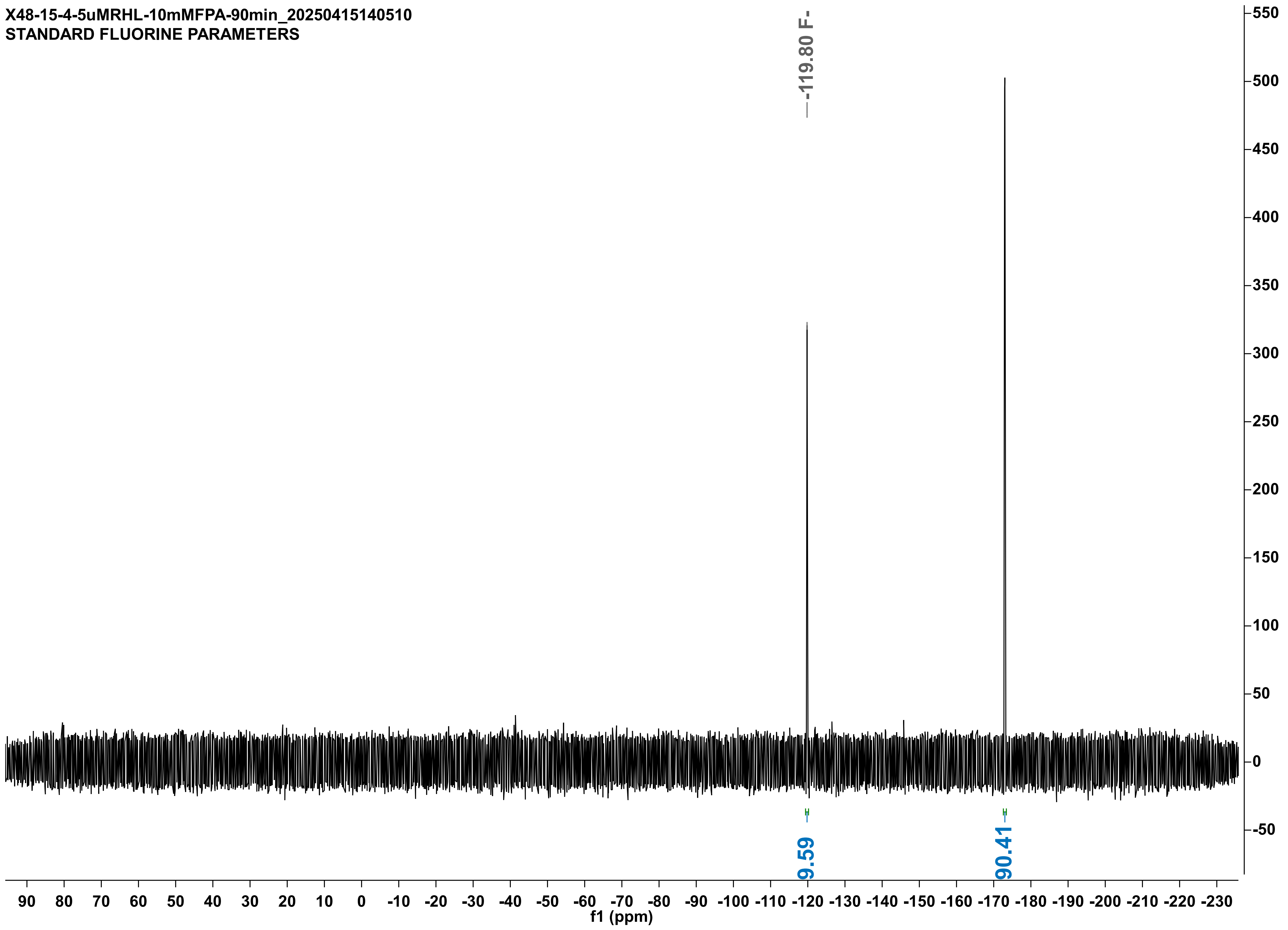
X48-15-4-5uMRHL-10mMFPA-90min_20250415140510
STANDARD FLUORINE PARAMETERS
-119.80 F-
9.59
90.41
f1 (ppm)

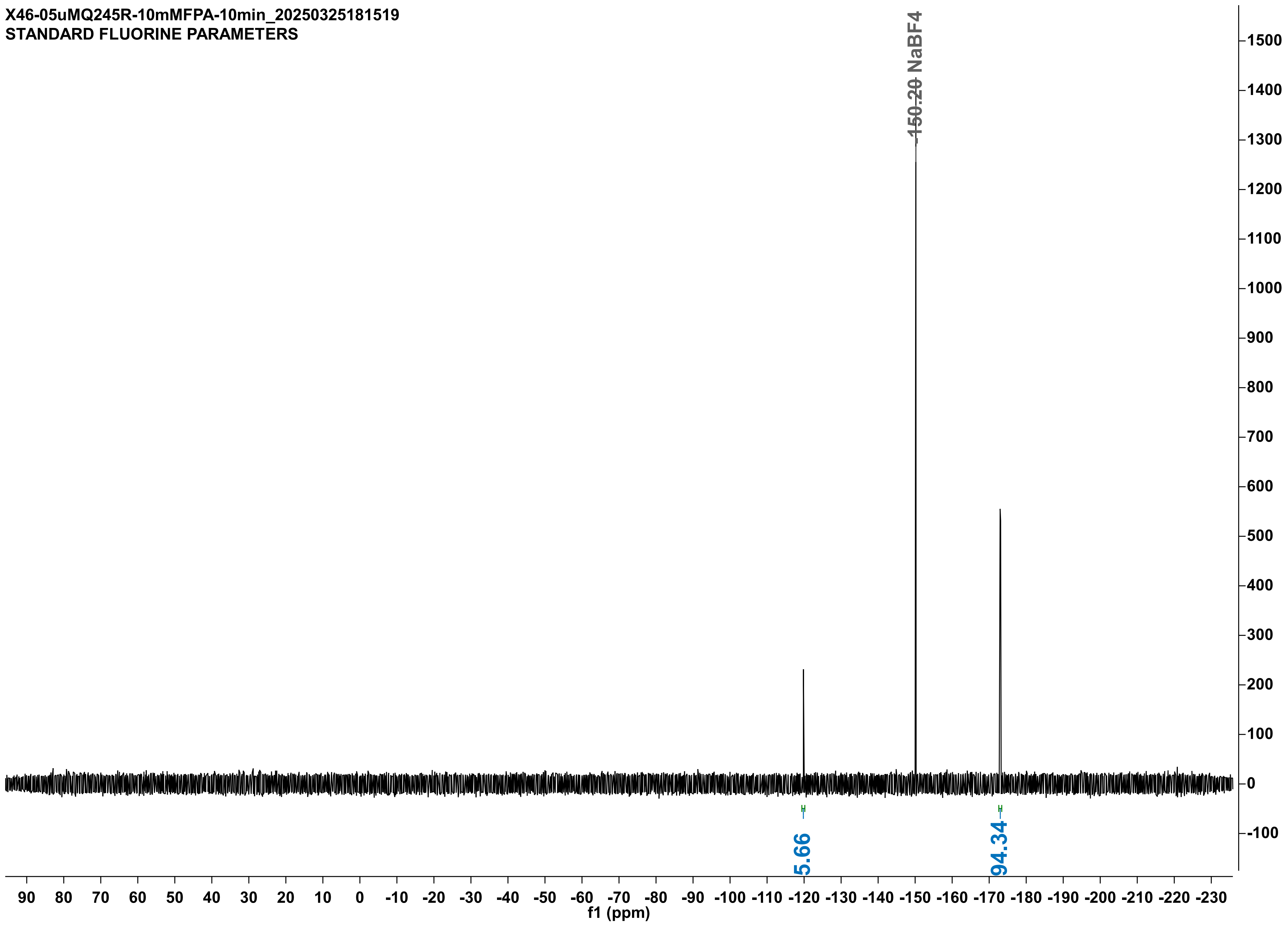

X46-05uMQ245R-10mMFPA-10min_20250325181519
STANDARD FLUORINE PARAMETERS
-150.20 NaBF4
5.66
94.34
f1 (ppm)

X46-9-4-05uMQ245R-DUPLO-10mMFPA-10min-noIS_20250409152234
STANDARD FLUORINE PARAMETERS

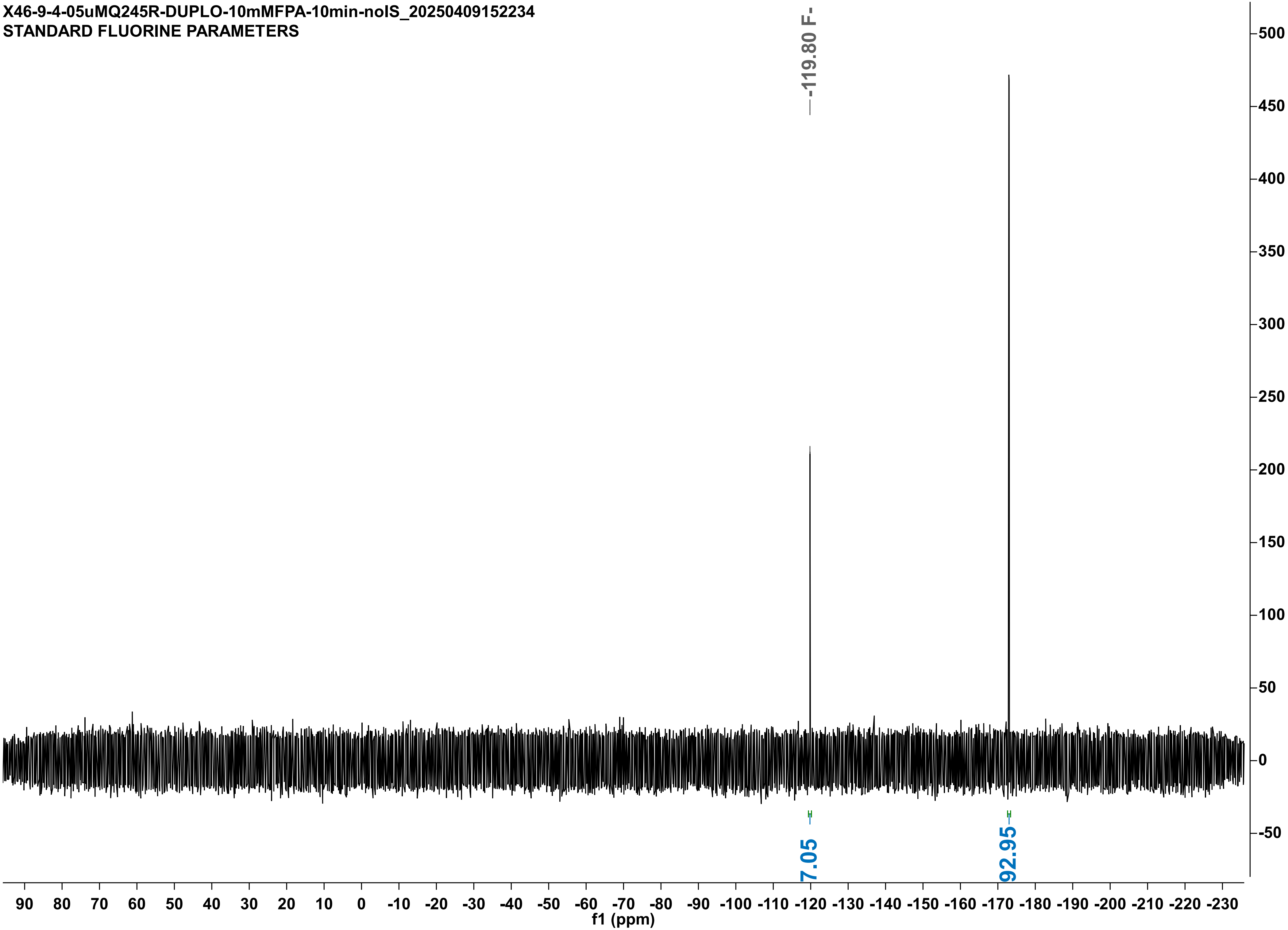

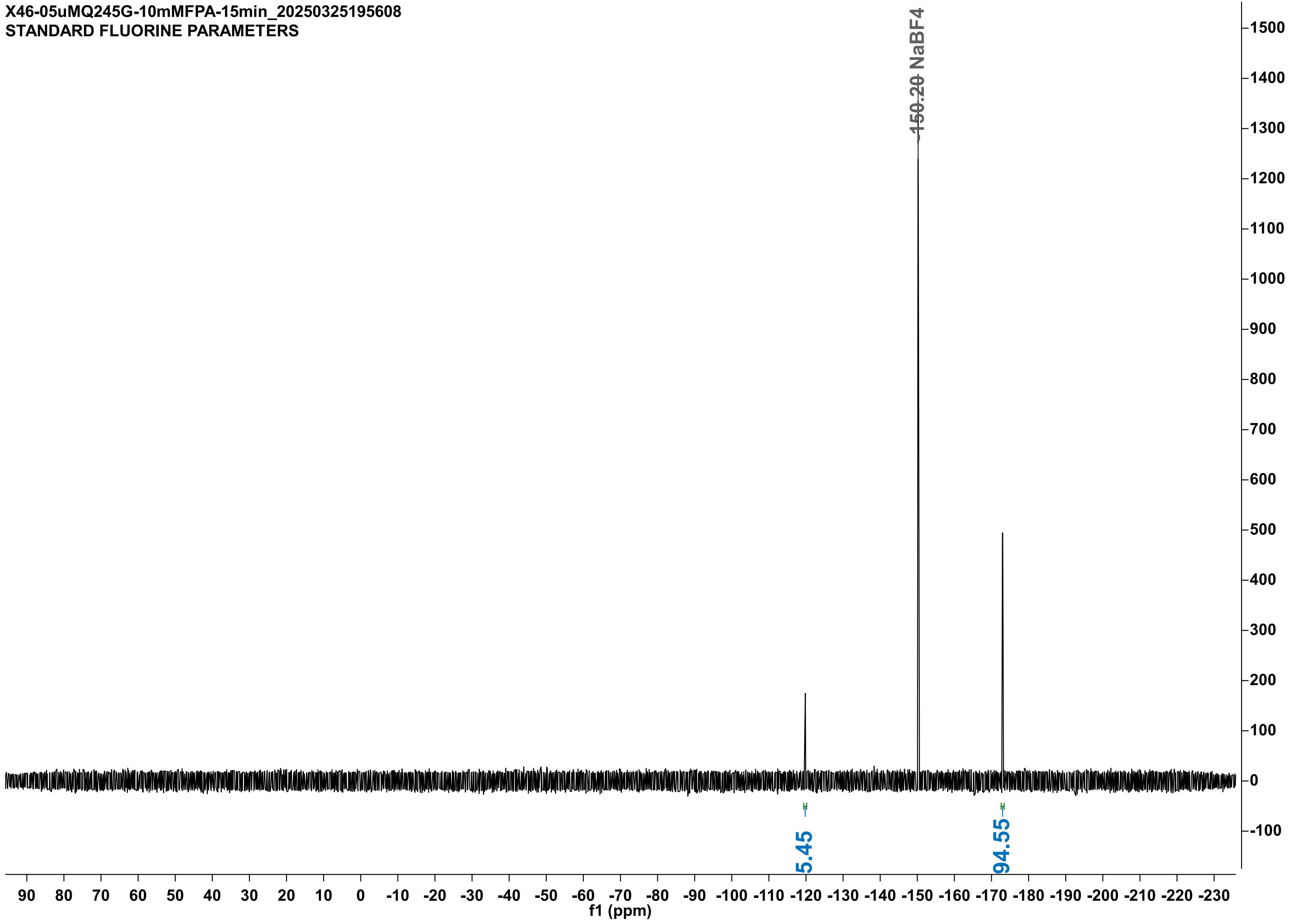

X46-05uMQ245G-10mMFPA-15min_20250325195608
STANDARD FLUORINE PARAMETERS
-150.20 NaBF4
5.45
94.55
f1 (ppm)

X46-9-4-05uMQ245G-DUPLO-10mMFPA-15min-noIS_20250409154236
STANDARD FLUORINE PARAMETERS

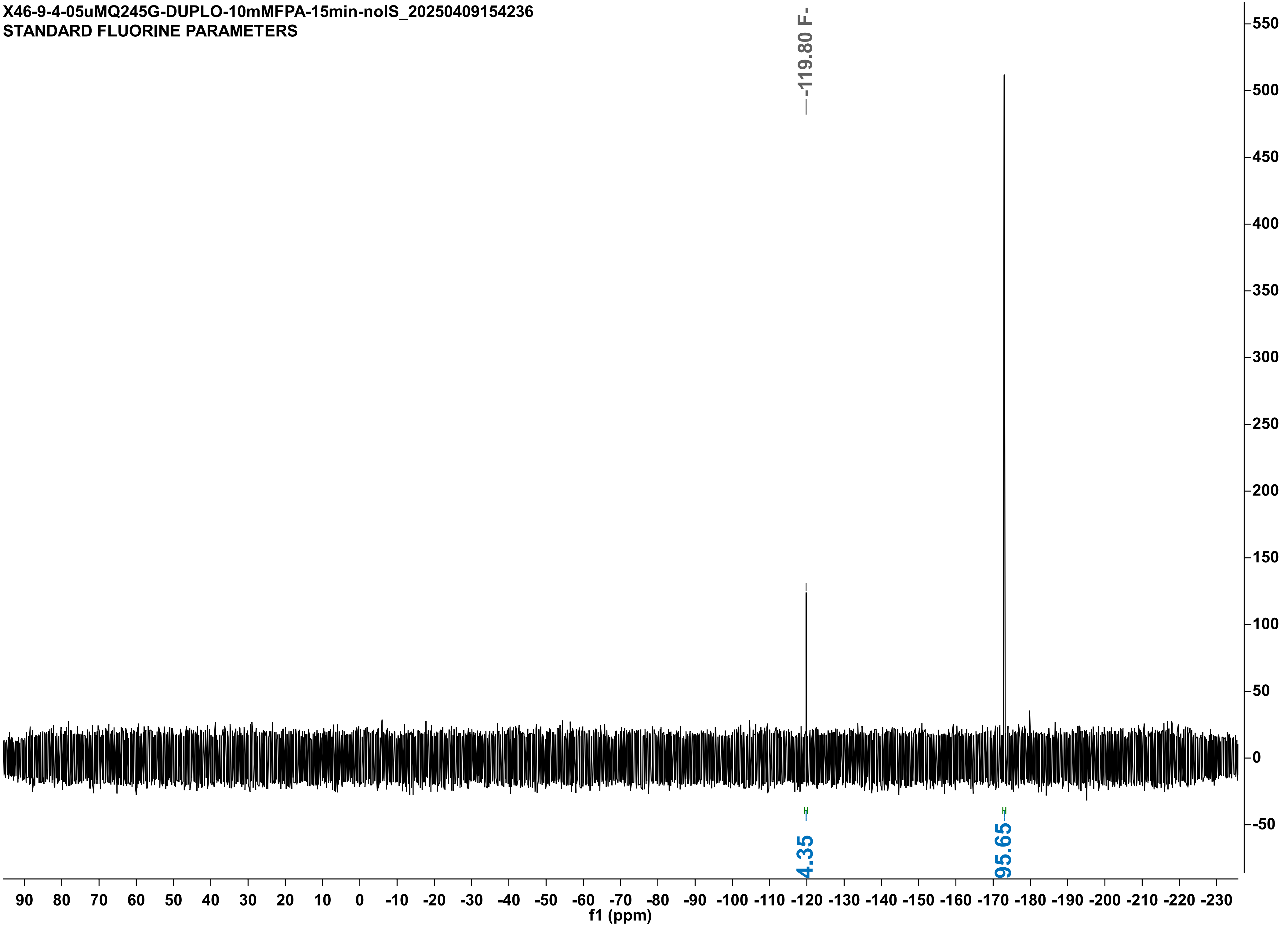

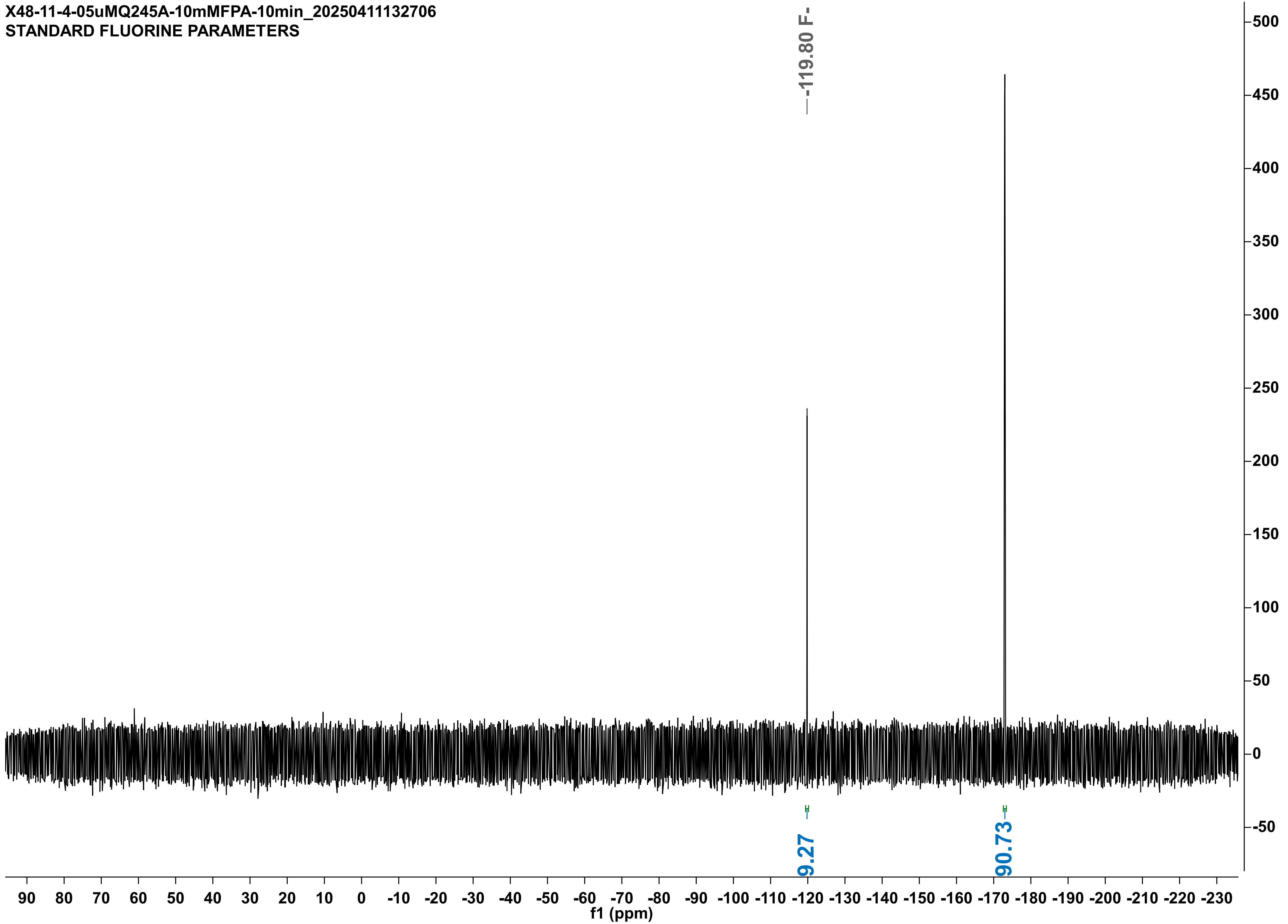
X48-11-4-05uMQ245A-10mMFPA-10min_20250411132706
STANDARD FLUORINE PARAMETERS
-119.80 F-
9.27
90.73
f1 (ppm)

X48-11-4-05uMQ245-DUPLO-10mMFPA-10min_20250411174154
STANDARD FLUORINE PARAMETERS

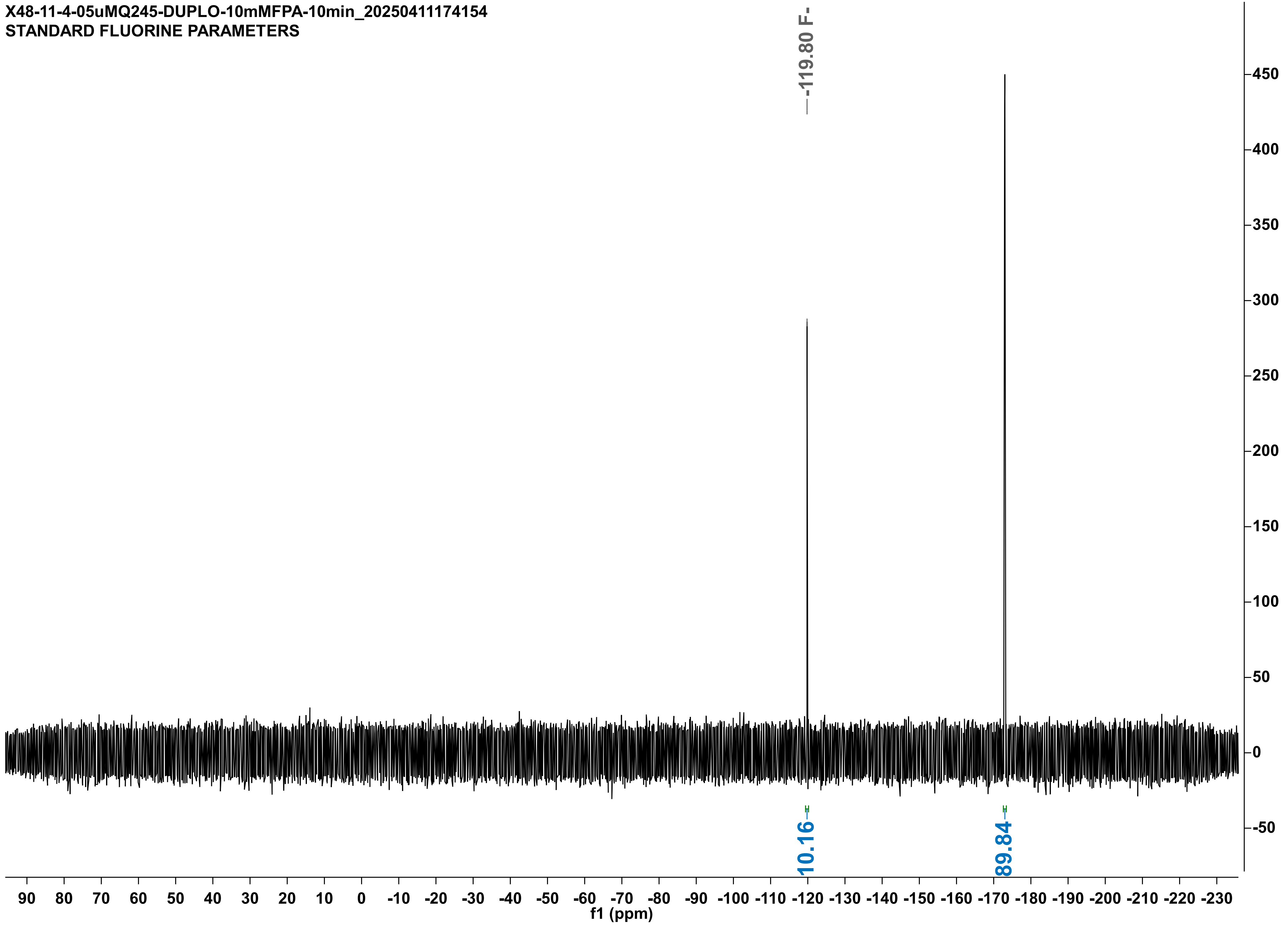

X48-15-4-5uM-ML-duplo-10mMFPA-40min_20250415155244
STANDARD FLUORINE PARAMETERS

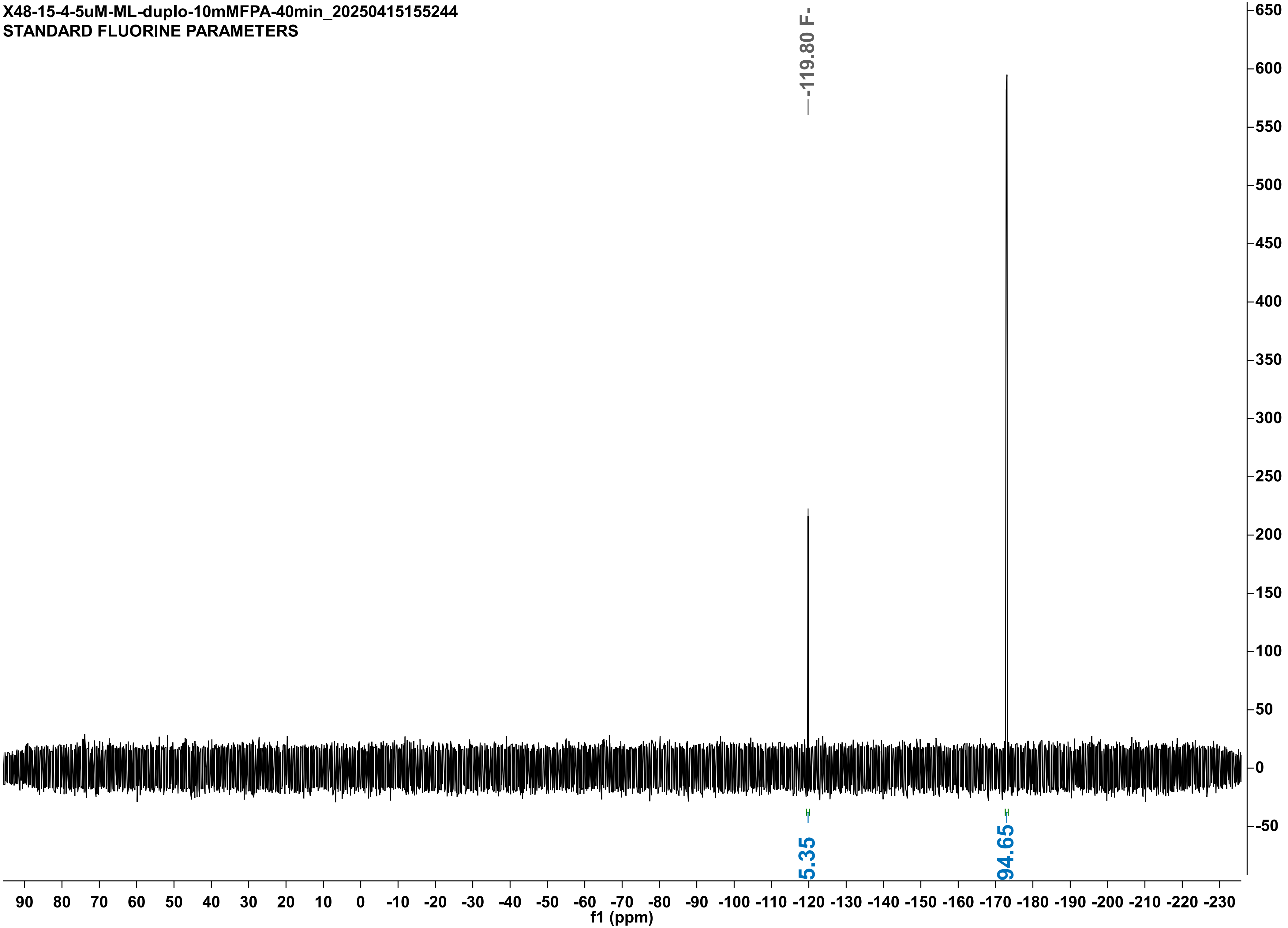

X48-15-4-5uM-ML-10mMFPA-40min_20250415144131
STANDARD FLUORINE PARAMETERS

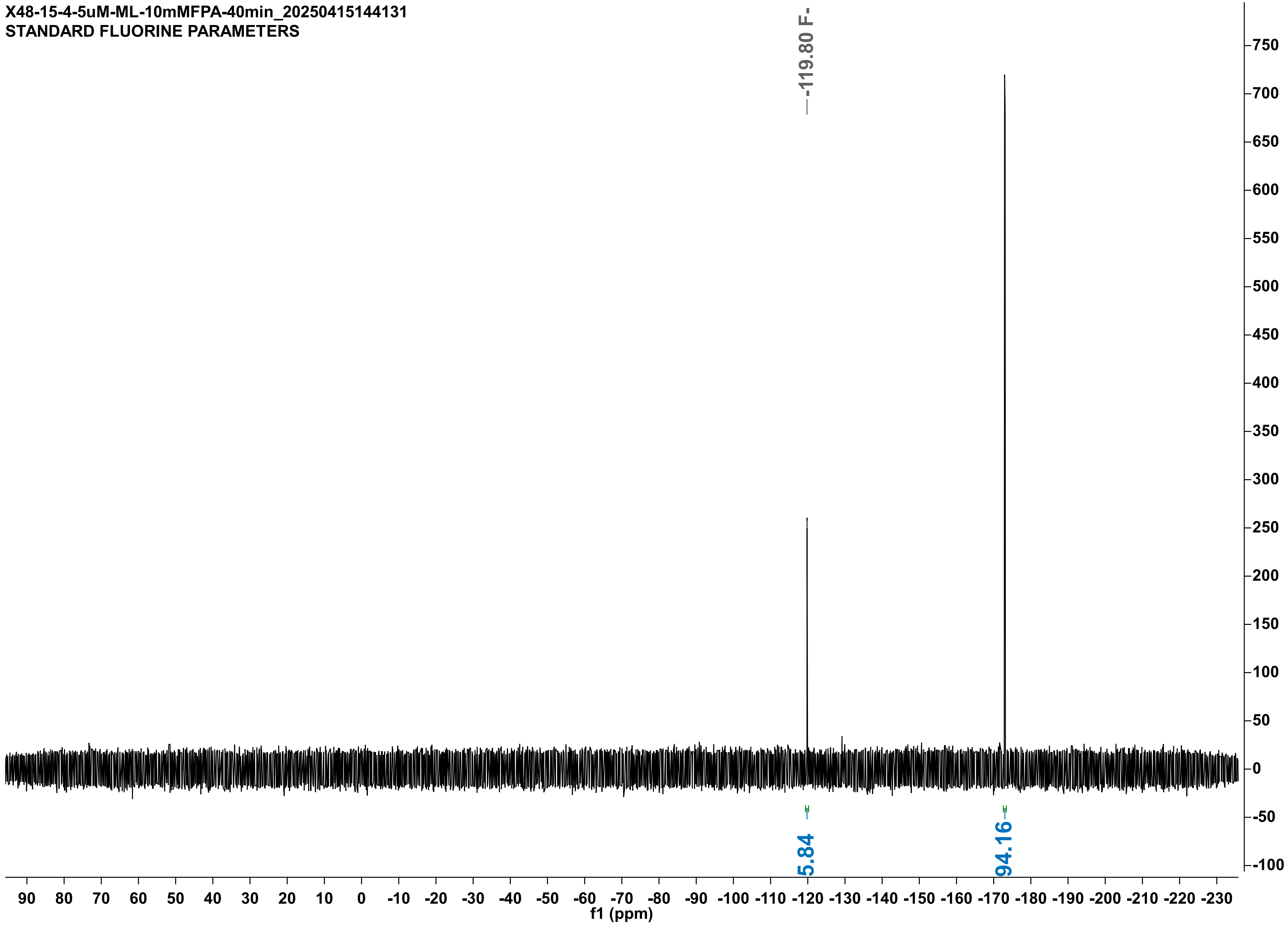

X48-16-4-05uMLVM-DUP-10mMFPA-15min_20250416184330
STANDARD FLUORINE PARAMETERS

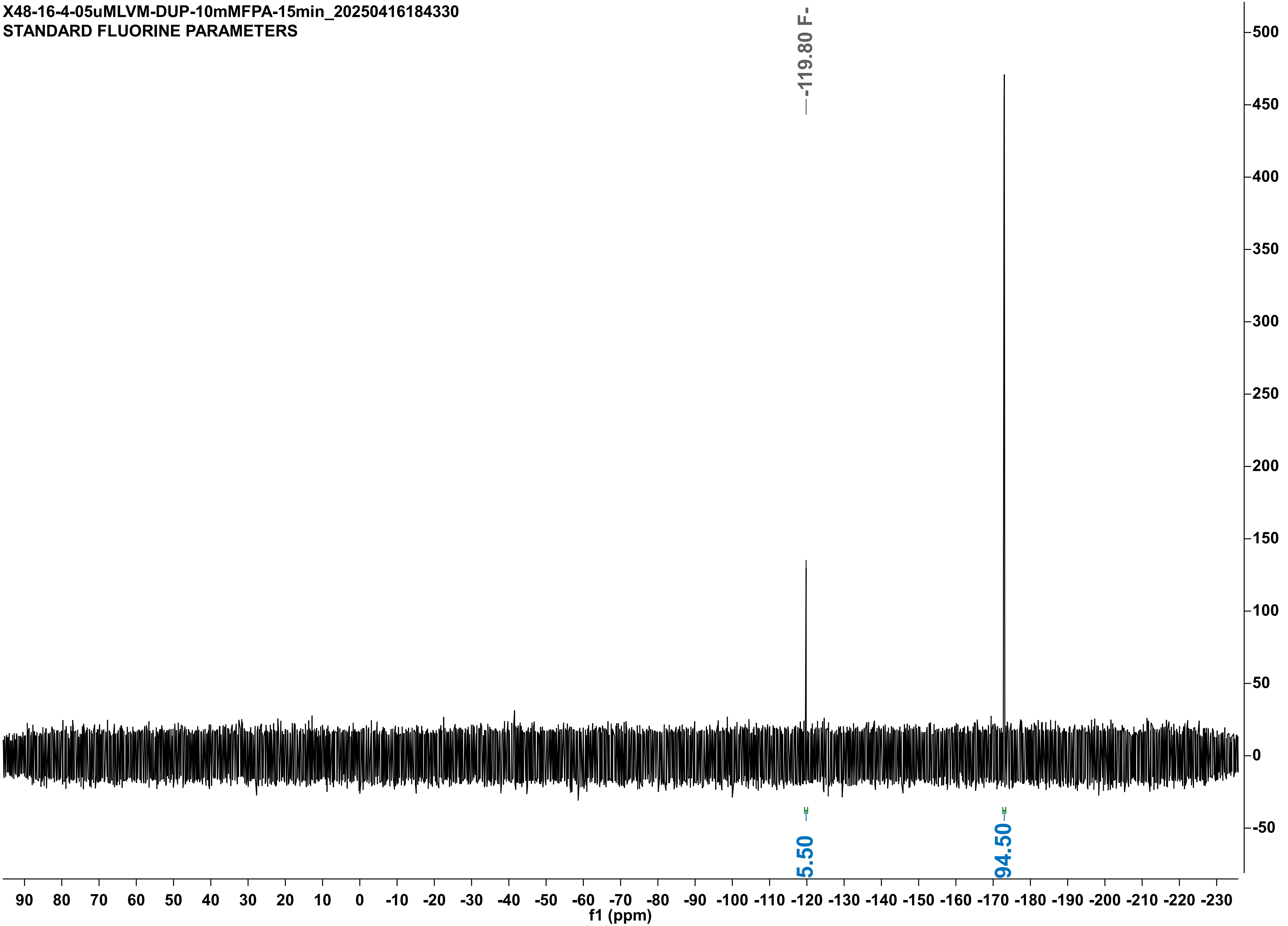

X48-16-4-05uMLVM-10mMFPA-15min_20250416184329
STANDARD FLUORINE PARAMETERS

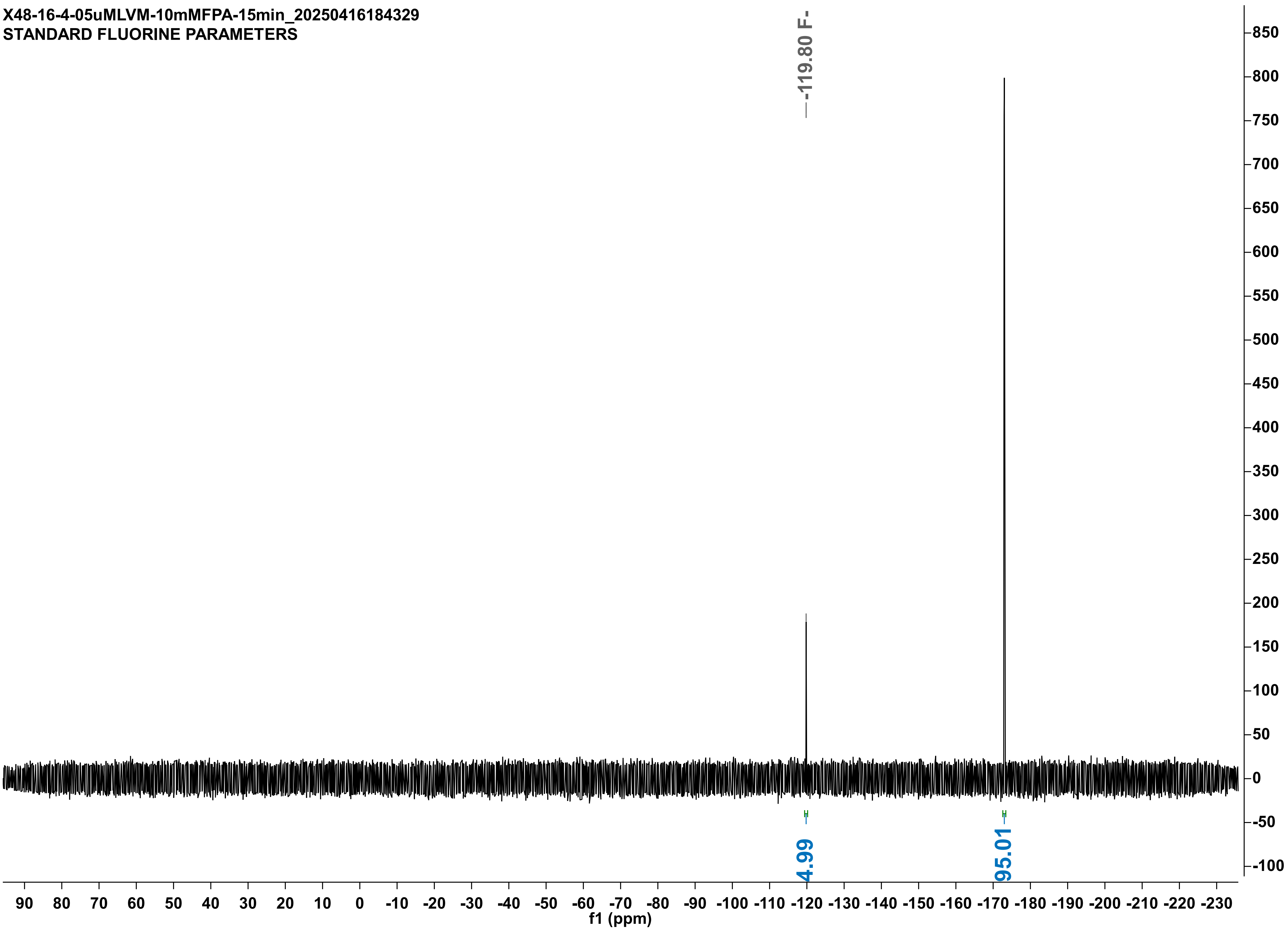

X46-9-4-05uM-FI-DUPLO-10mMFPA-15min-noIS_20250409154310
STANDARD FLUORINE PARAMETERS

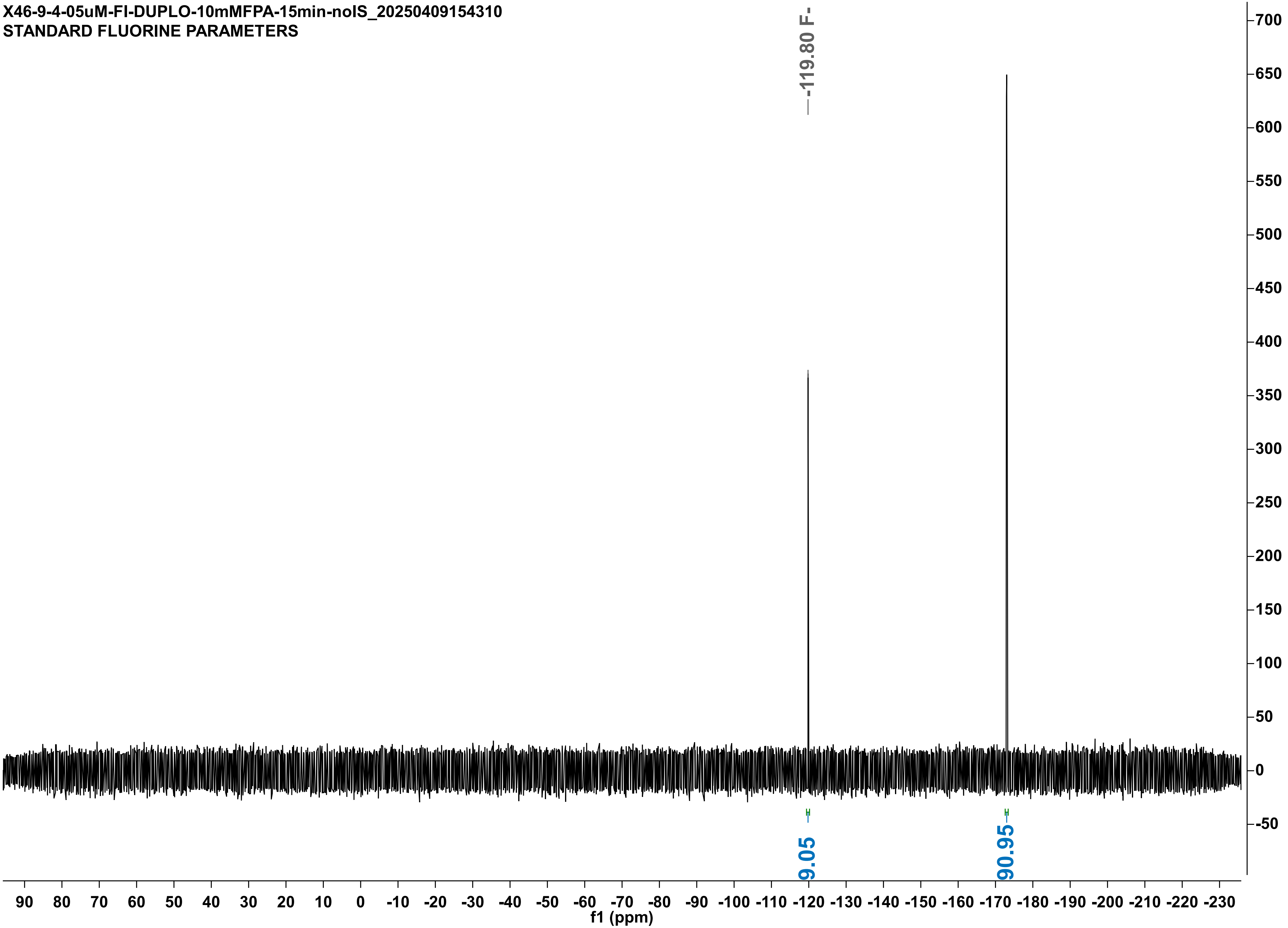

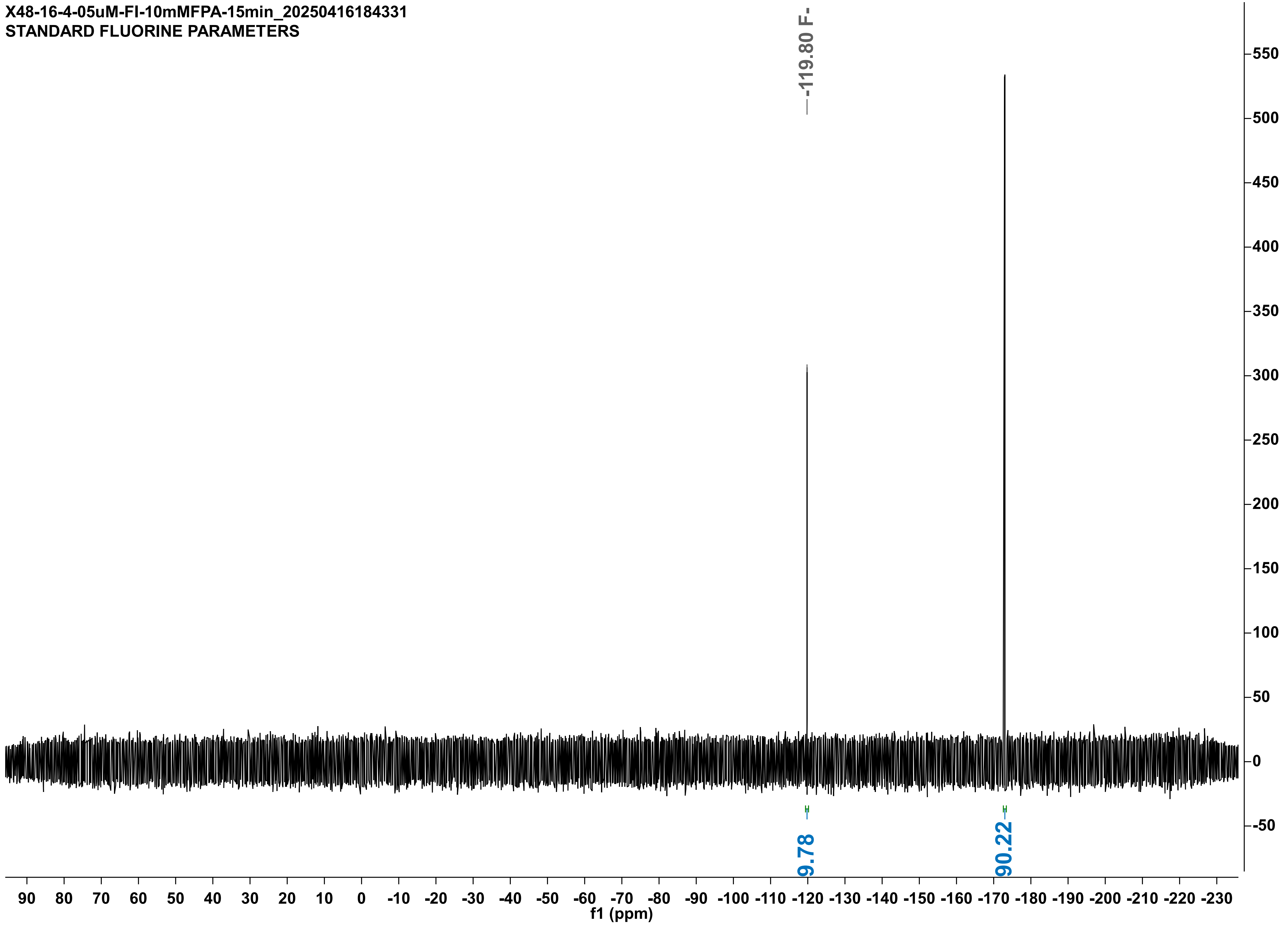

X48-16-4-05uM-FI-10mMFPA-15min_20250416184331
STANDARD FLUORINE PARAMETERS
-119.80 F-
9.78
90.22
f1 (ppm)

X46-1uMWT2-10mM2FAc-24h_20250319172047
STANDARD FLUORINE PARAMETERS

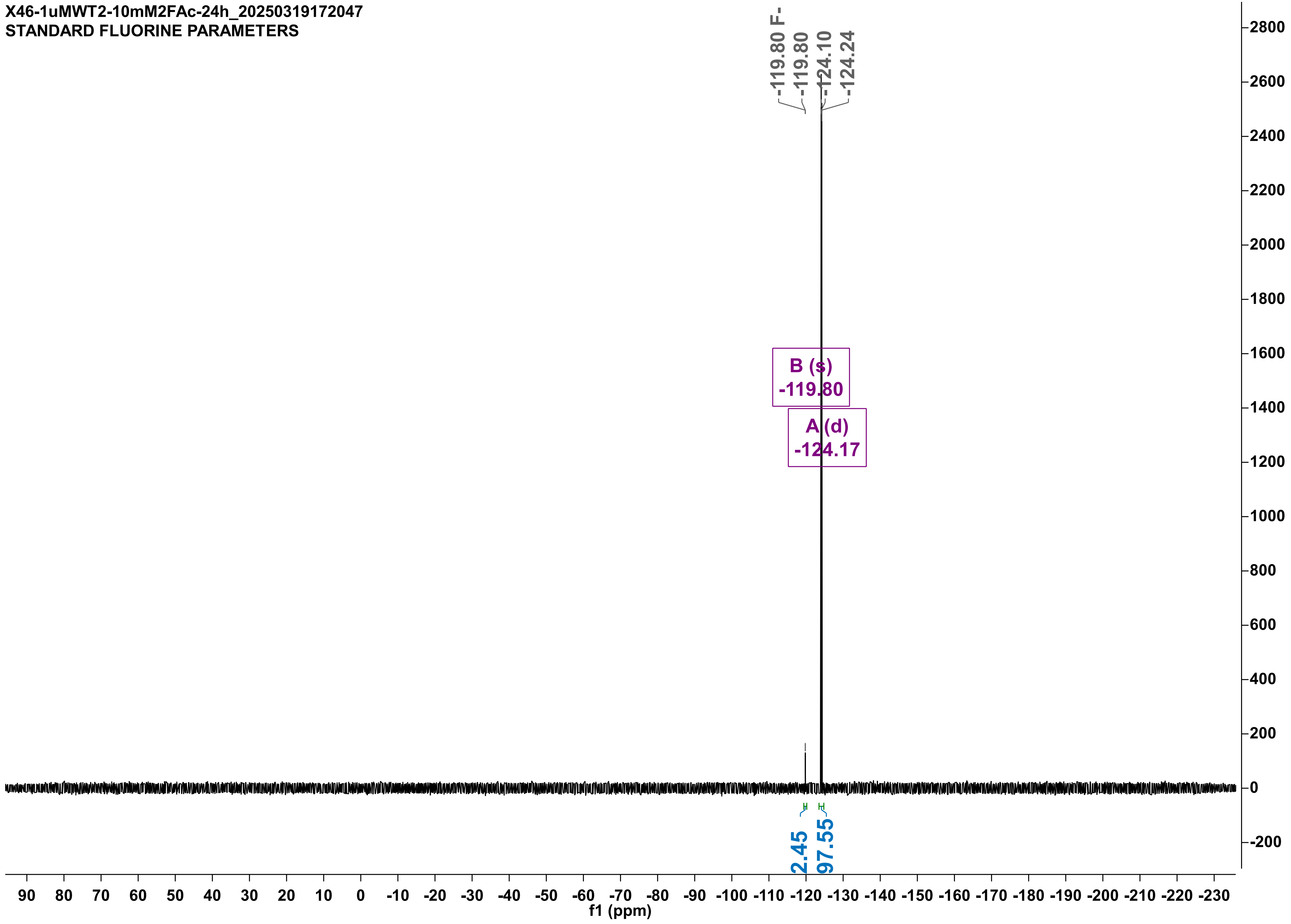

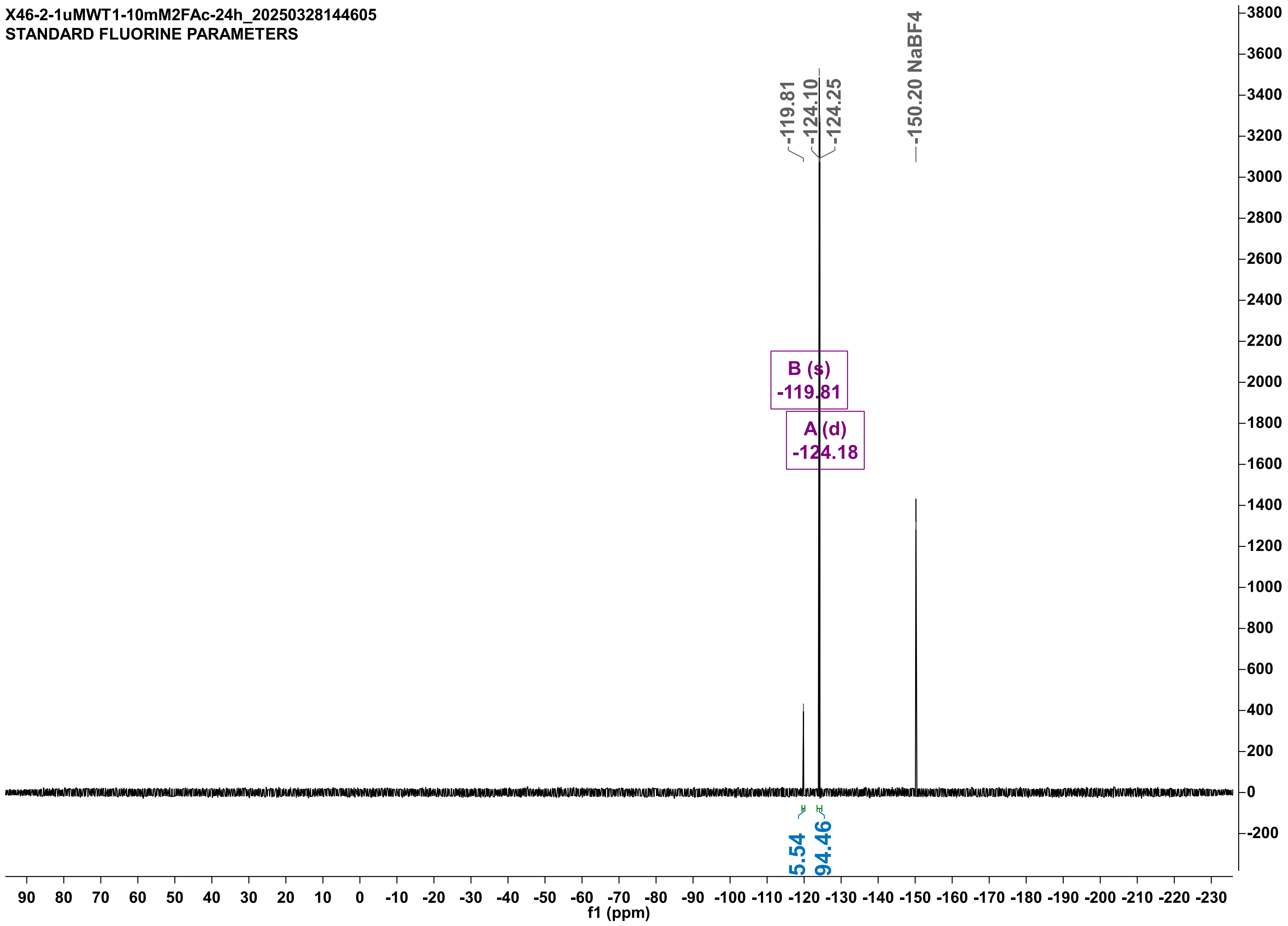

X46-2-1uMWT1-10mM2FAc-24h_20250328144605
STANDARD FLUORINE PARAMETERS
-119.81
-124.10
-124.25
-150.20 NaBF4
B (s)
-119.81
A (d)
-124.18
5.54
94.46
f1 (ppm)

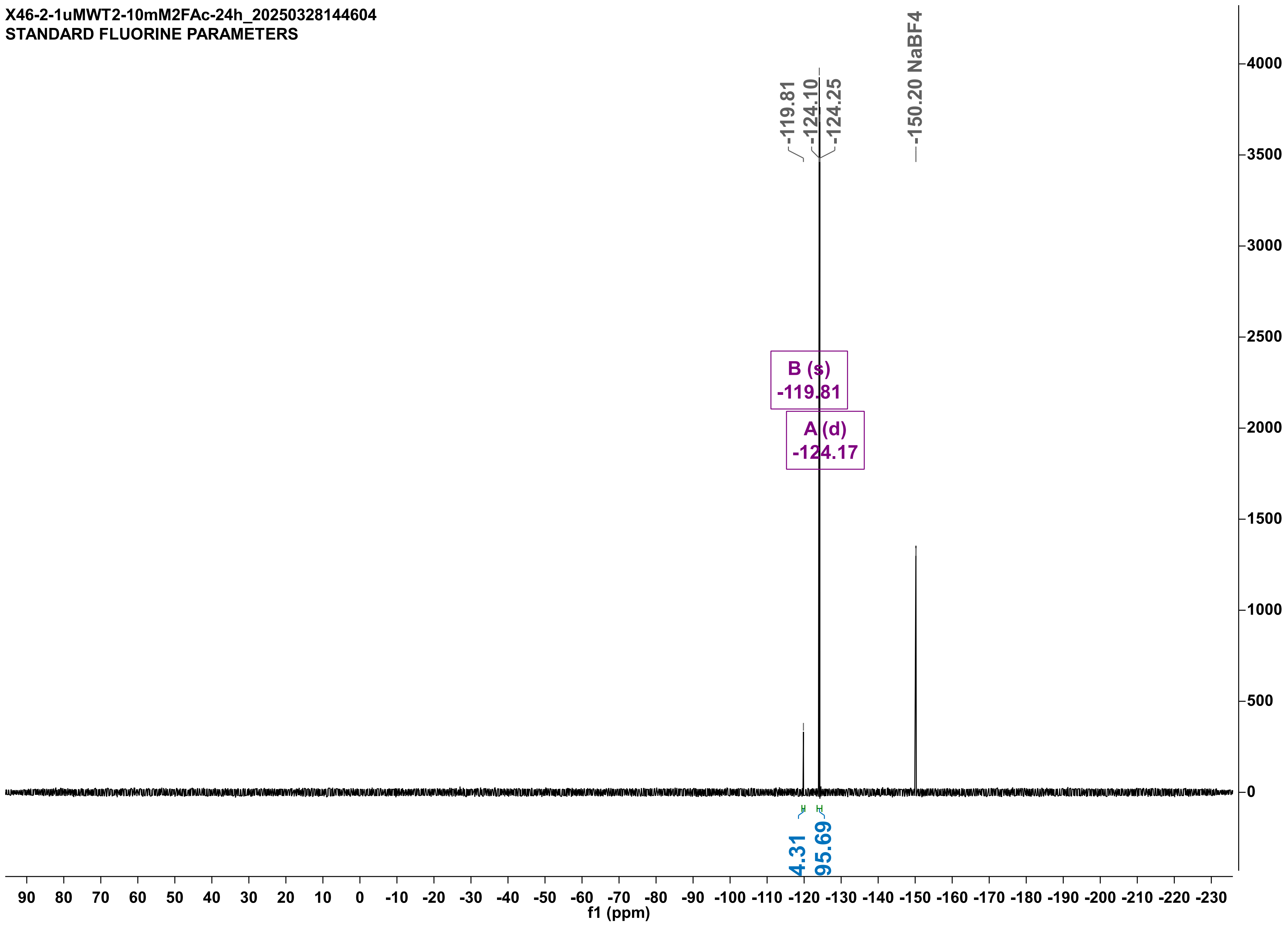

X46-2-1uMWT2-10mM2FAc-24h_20250328144604
STANDARD FLUORINE PARAMETERS
-119.81
-124.10
-124.25
-150.20 NaBF4
B (s)
-119.81
A (d)
-124.17
4.31
95.69
f1 (ppm)

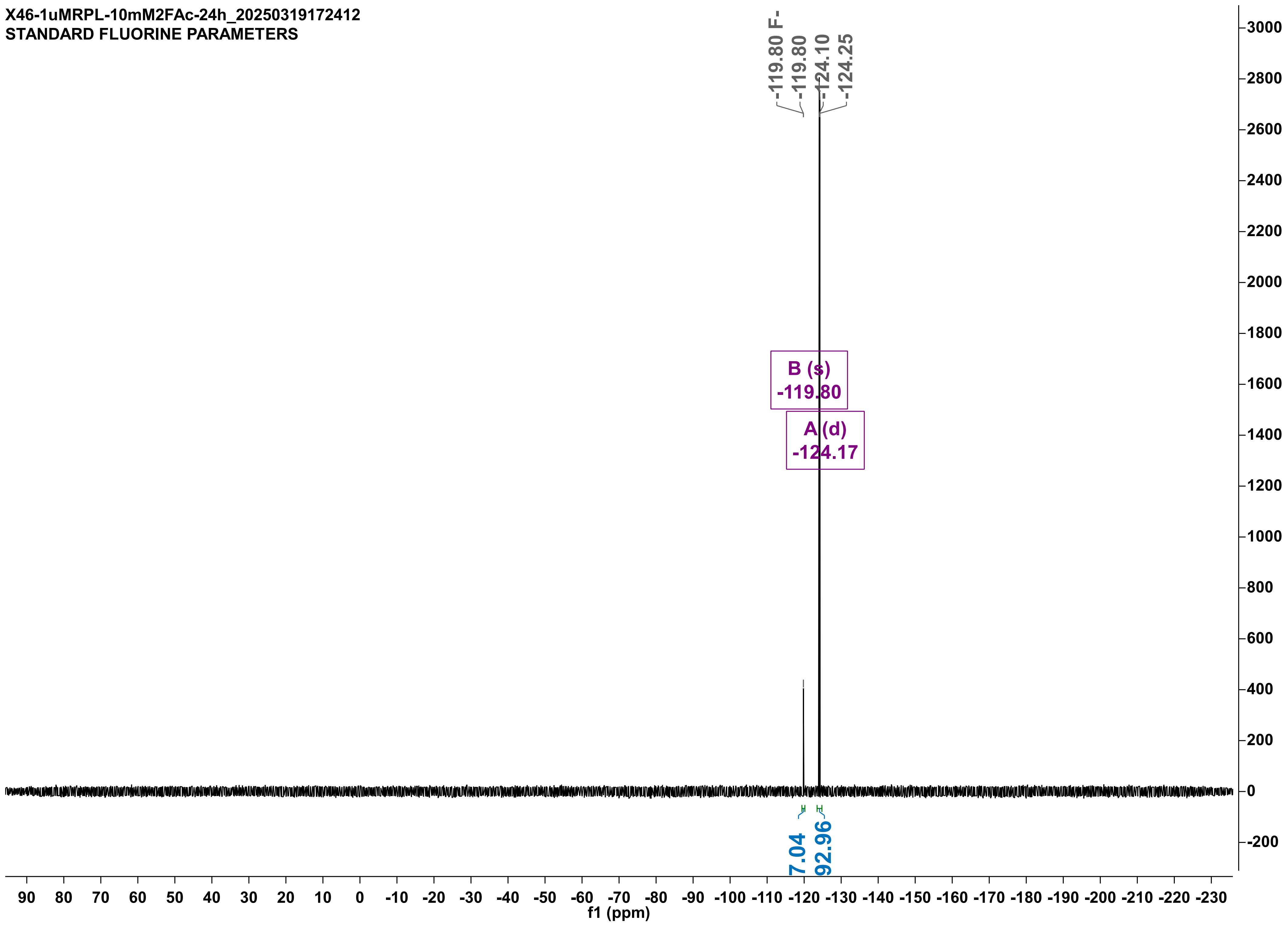

X46-1uMRPL-10mM2FAc-24h_20250319172412
STANDARD FLUORINE PARAMETERS
-119.80 F-
-119.80
-124.10
-124.25
B (s)
-119.80
A (d)
-124.17
7.04
92.96
f1 (ppm)

X46-2-1uMRPL-duplo-10mM2FAc-24H-noIS_20250328184254
STANDARD FLUORINE PARAMETERS

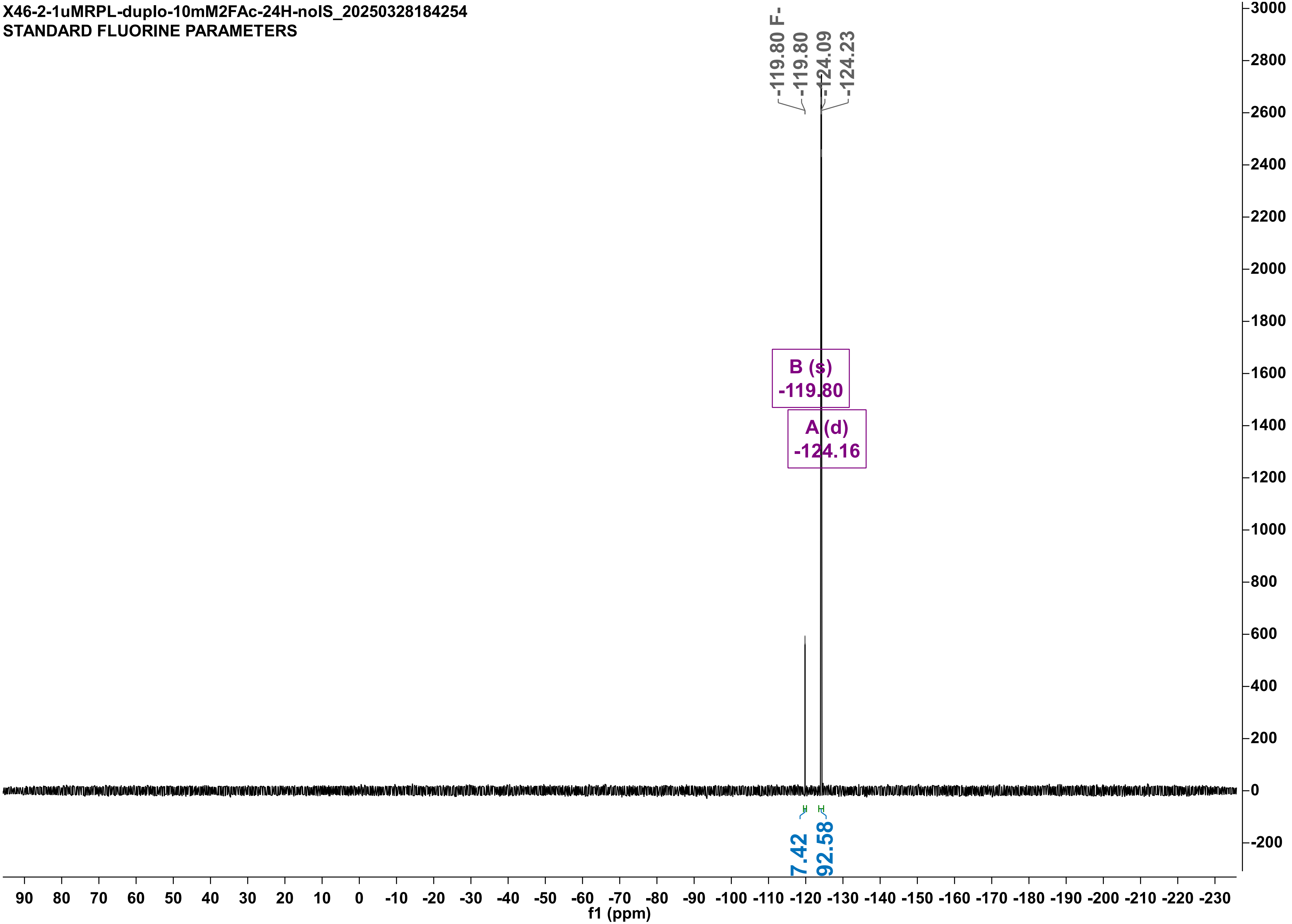

X46-1uMVW--10mM2FAc-24h_20250319173217
STANDARD FLUORINE PARAMETERS

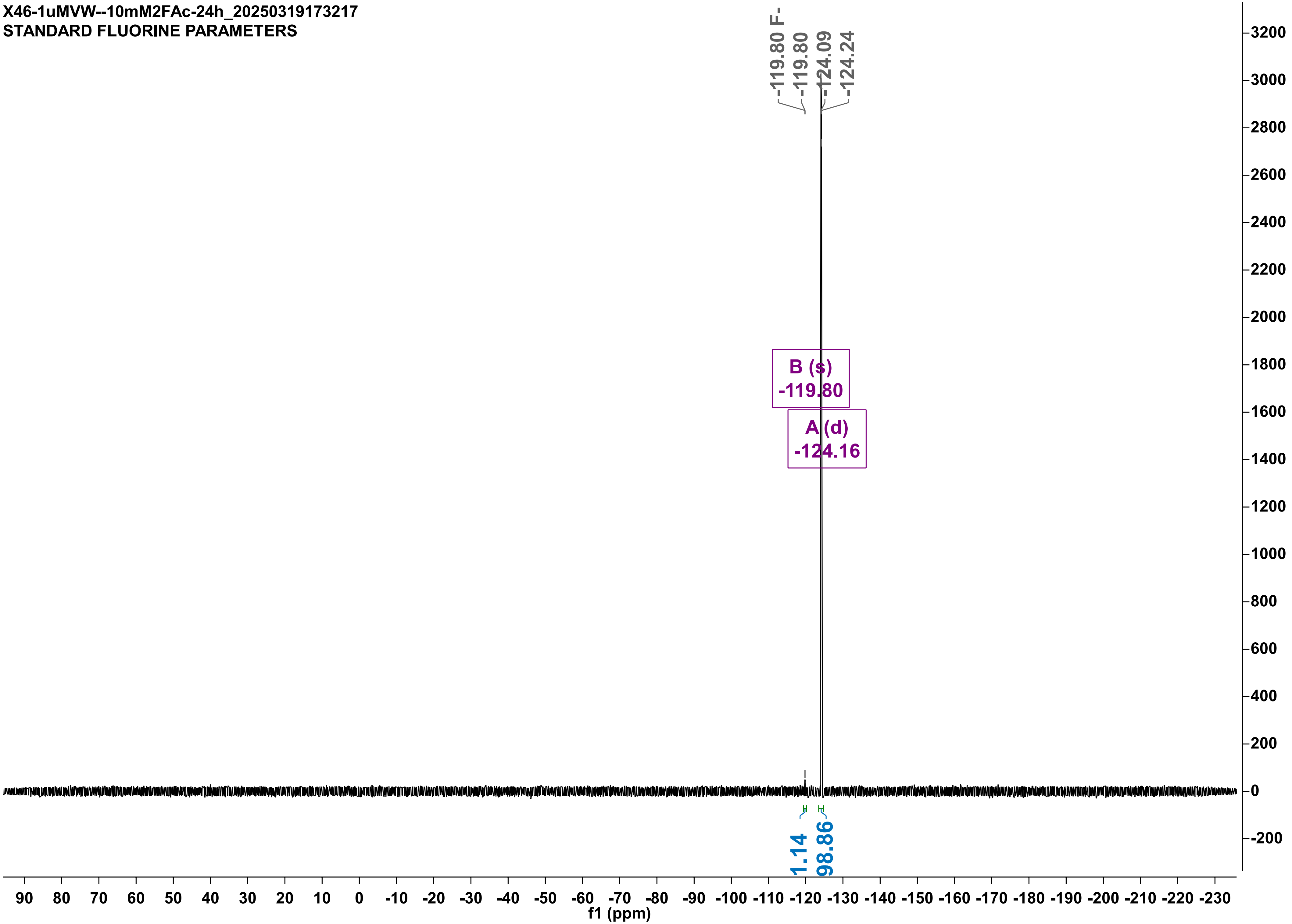

X46-9-4-1uMVW--remeasure-10mM2FAc-24h-noIS_20250410134628
STANDARD FLUORINE PARAMETERS

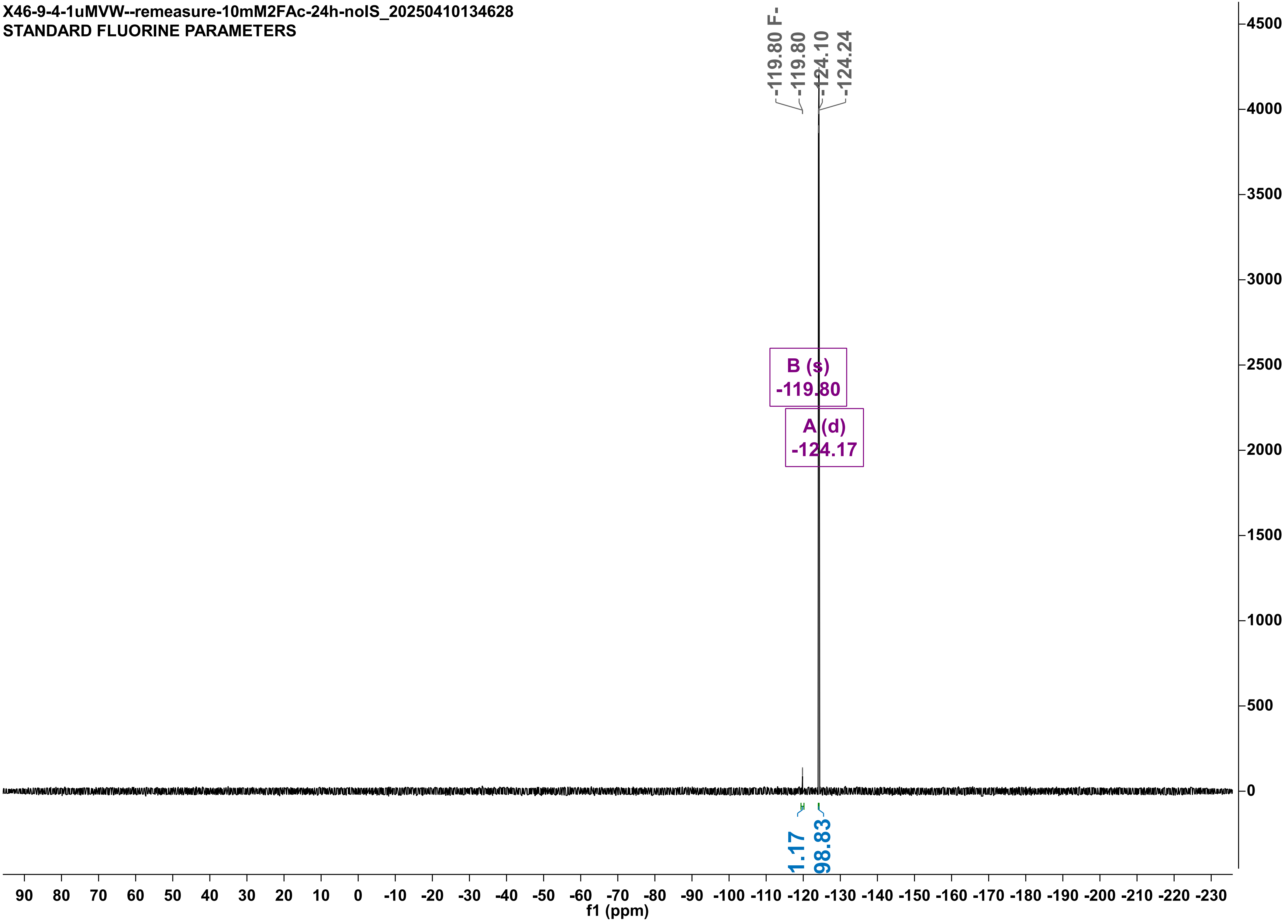

X46-1uMLW--10mM2FAc-24h_20250319173817
STANDARD FLUORINE PARAMETERS

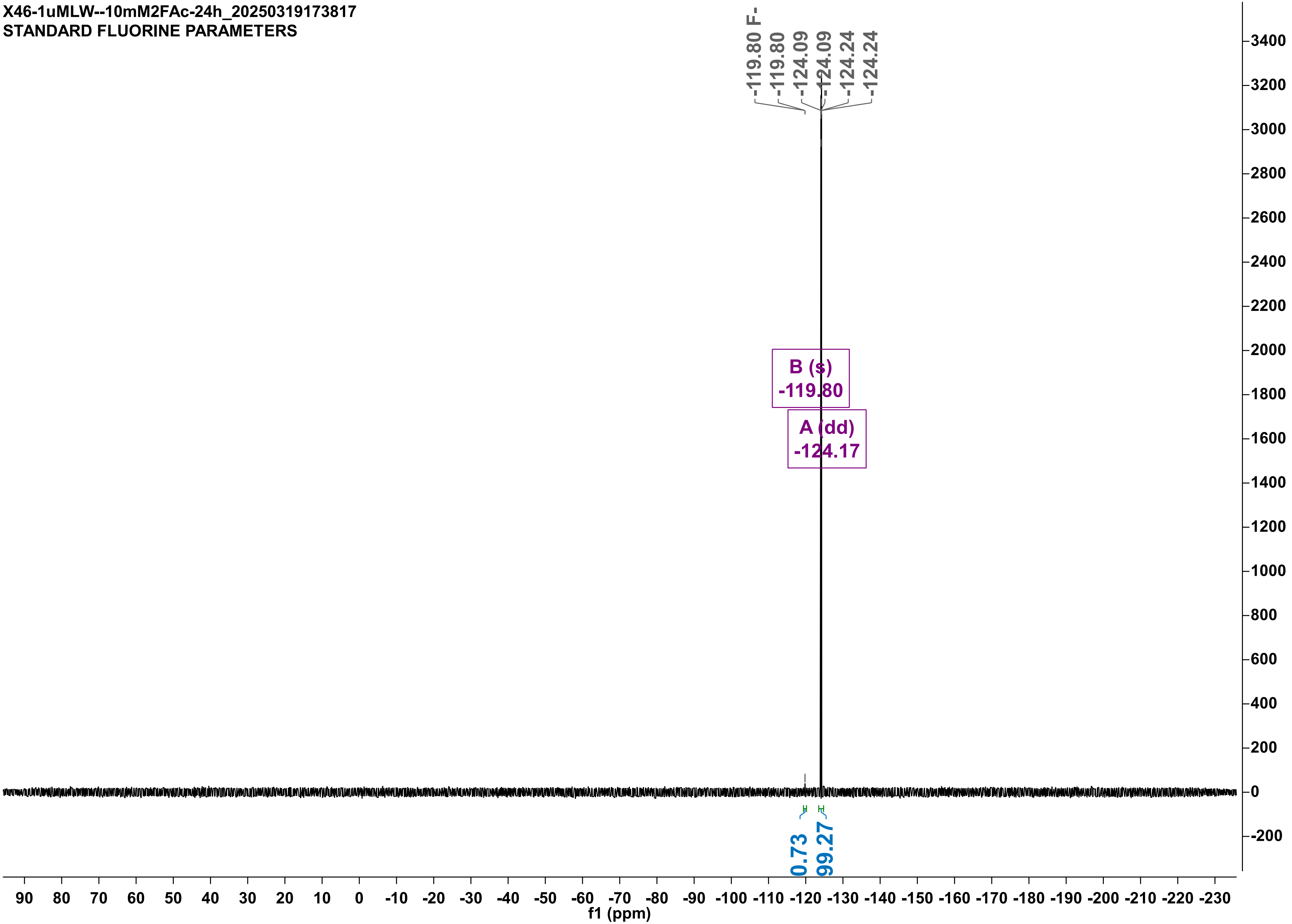

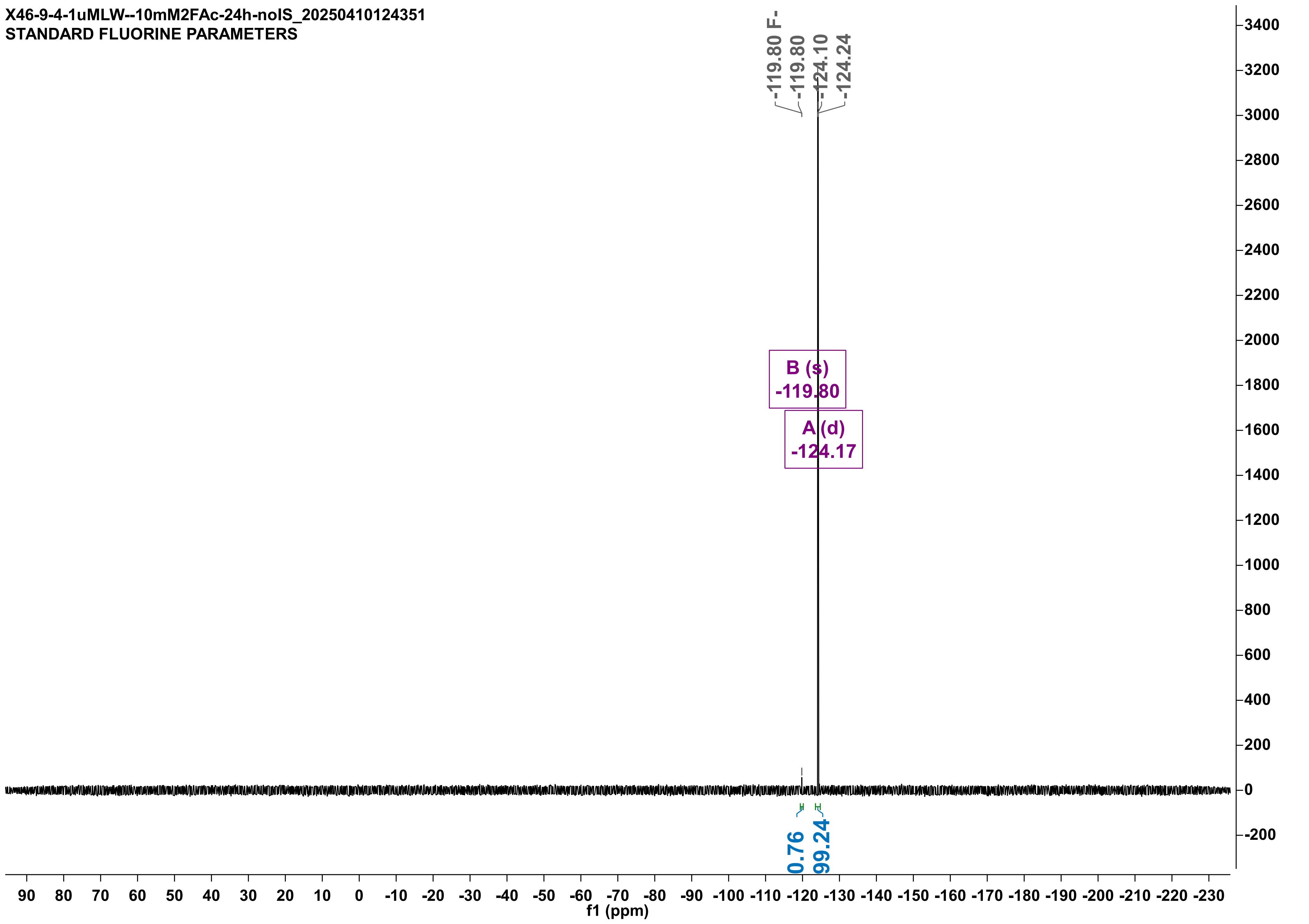
X46-9-4-1uMLW--10mM2FAc-24h-noIS_20250410124351
STANDARD FLUORINE PARAMETERS
-119.80 F-
-119.80
-124.10
-124.24
B (s)
-119.80
A (d)
-124.17
0.76
99.24
f1 (ppm)

X46-1uMRLM-10mM2FAc-24h_20250321155455
STANDARD FLUORINE PARAMETERS

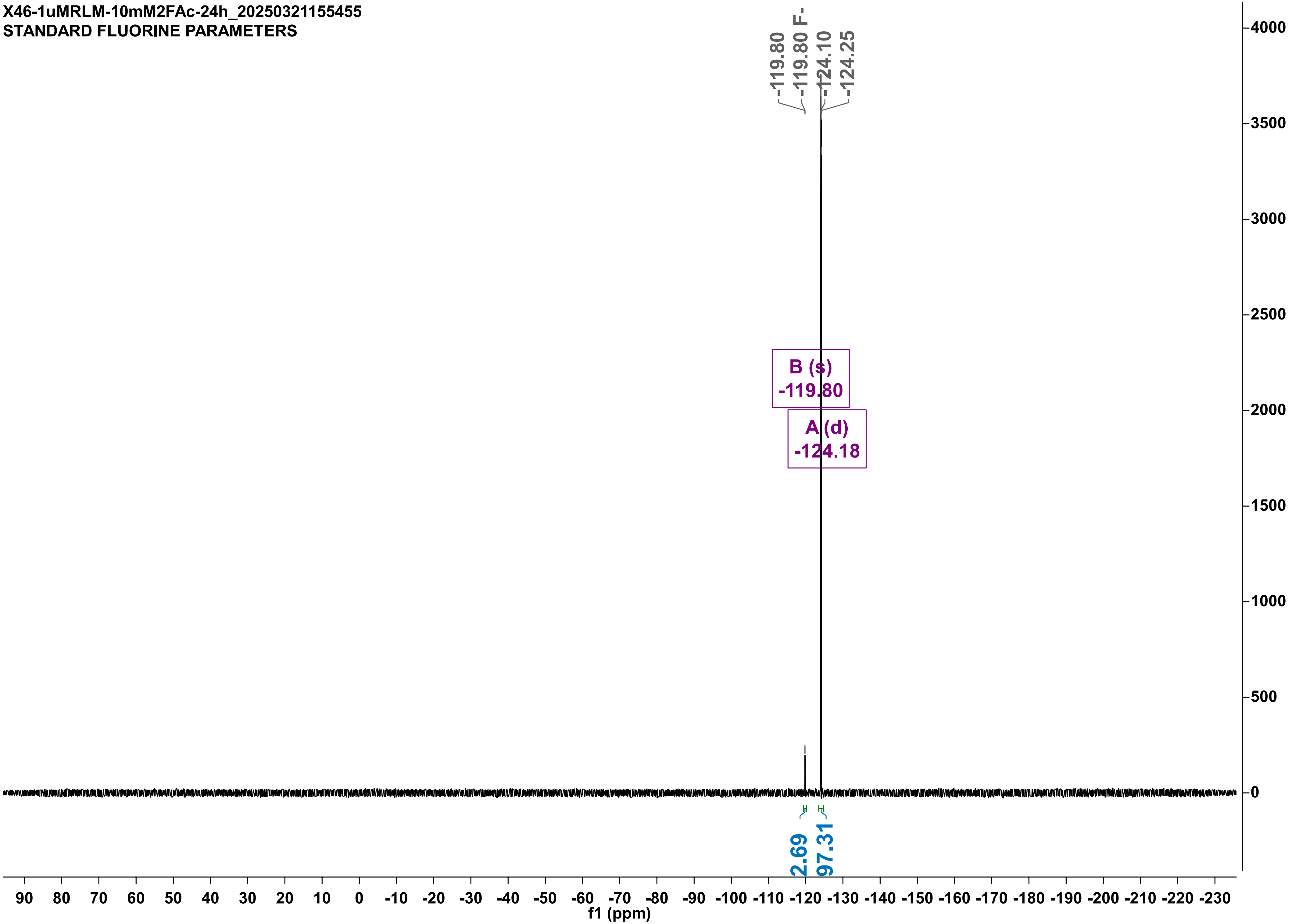

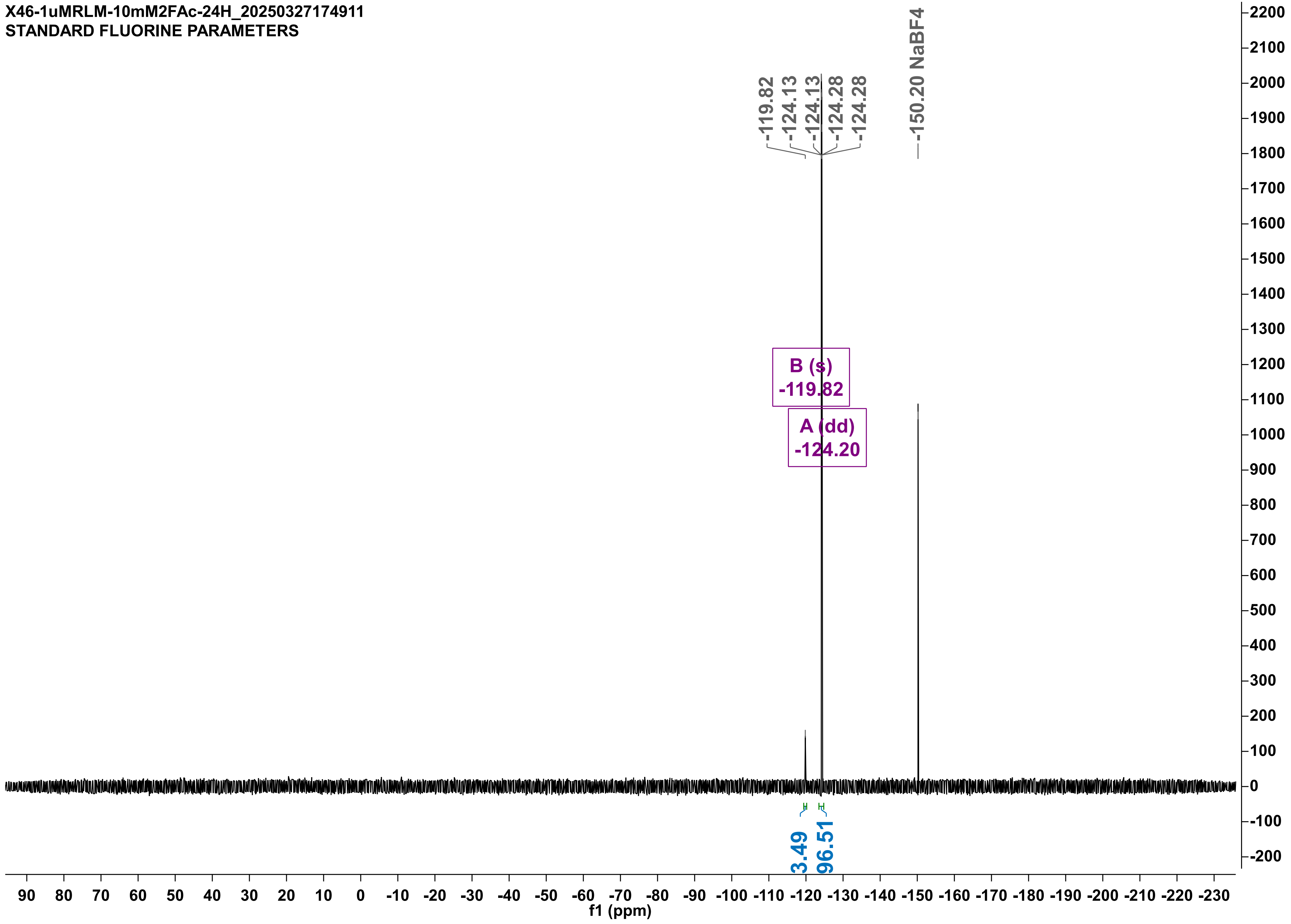

X46-1uMRLM-10mM2FAc-24H_20250327174911
STANDARD FLUORINE PARAMETERS
-119.82
-124.13
-124.13
-124.28
-124.28
-150.20 NaBF4
B (s)
-119.82
A (dd)
-124.20
3.49
96.51
f1 (ppm)

X46-1uMRHL-10mM2FAc-24h_20250321155408
STANDARD FLUORINE PARAMETERS

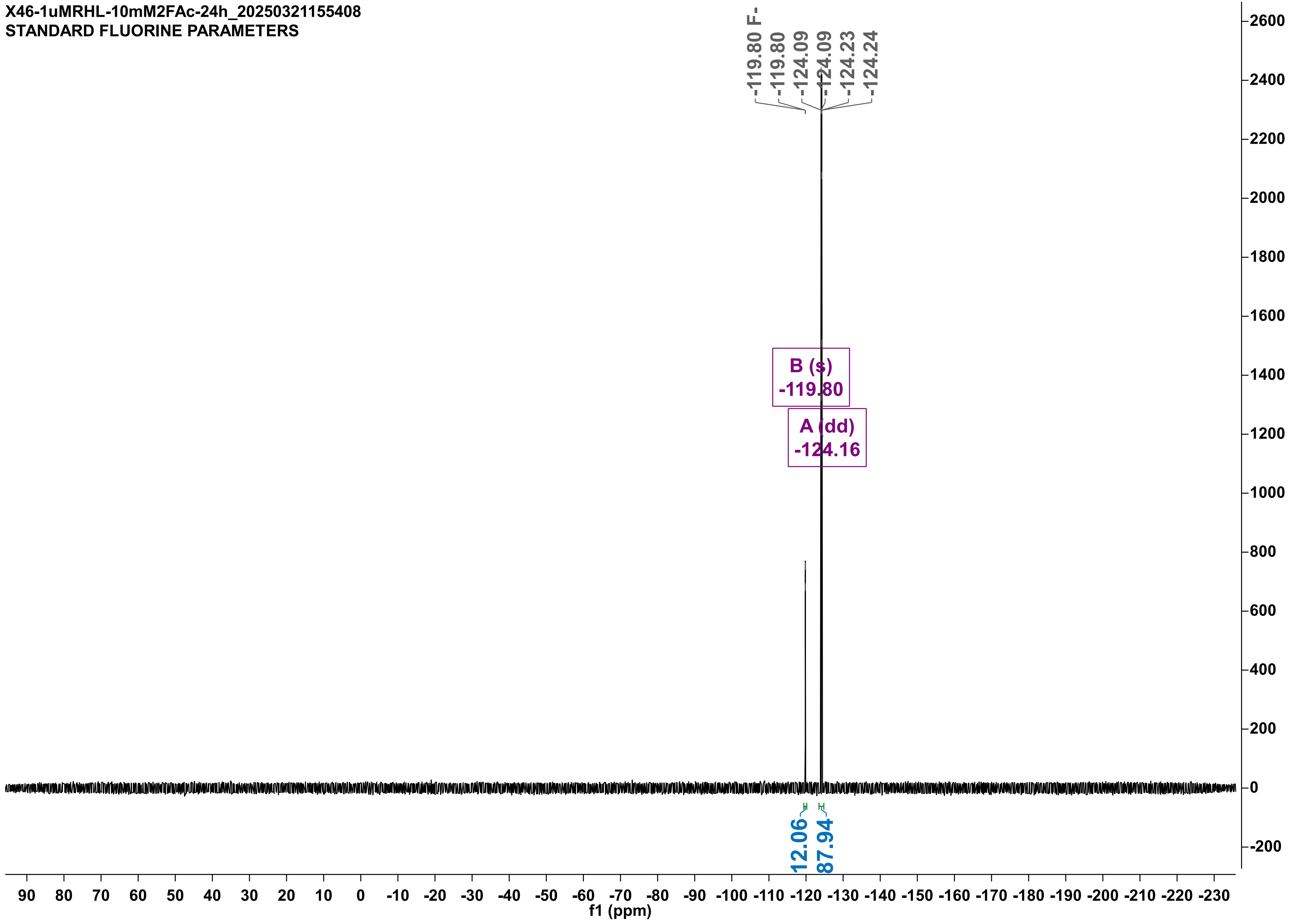

X46-9-4-1uMRHL-DUPLO-10mM2FAc-24h-noIS_20250410133658
STANDARD FLUORINE PARAMETERS

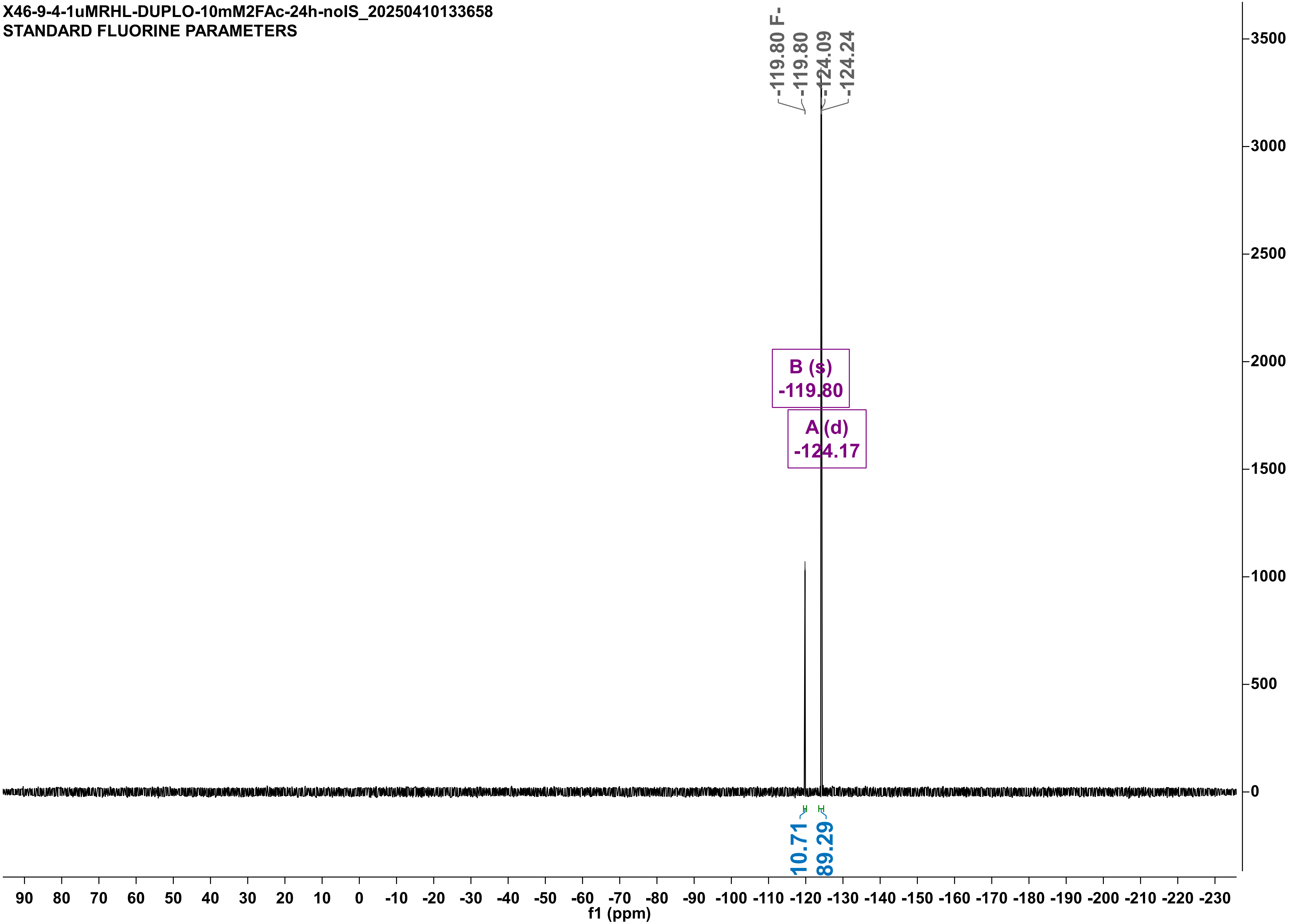

X46-9-4-1uM-VM-DUPLO-10mM2FAc-24h-noIS_20250410133608
STANDARD FLUORINE PARAMETERS

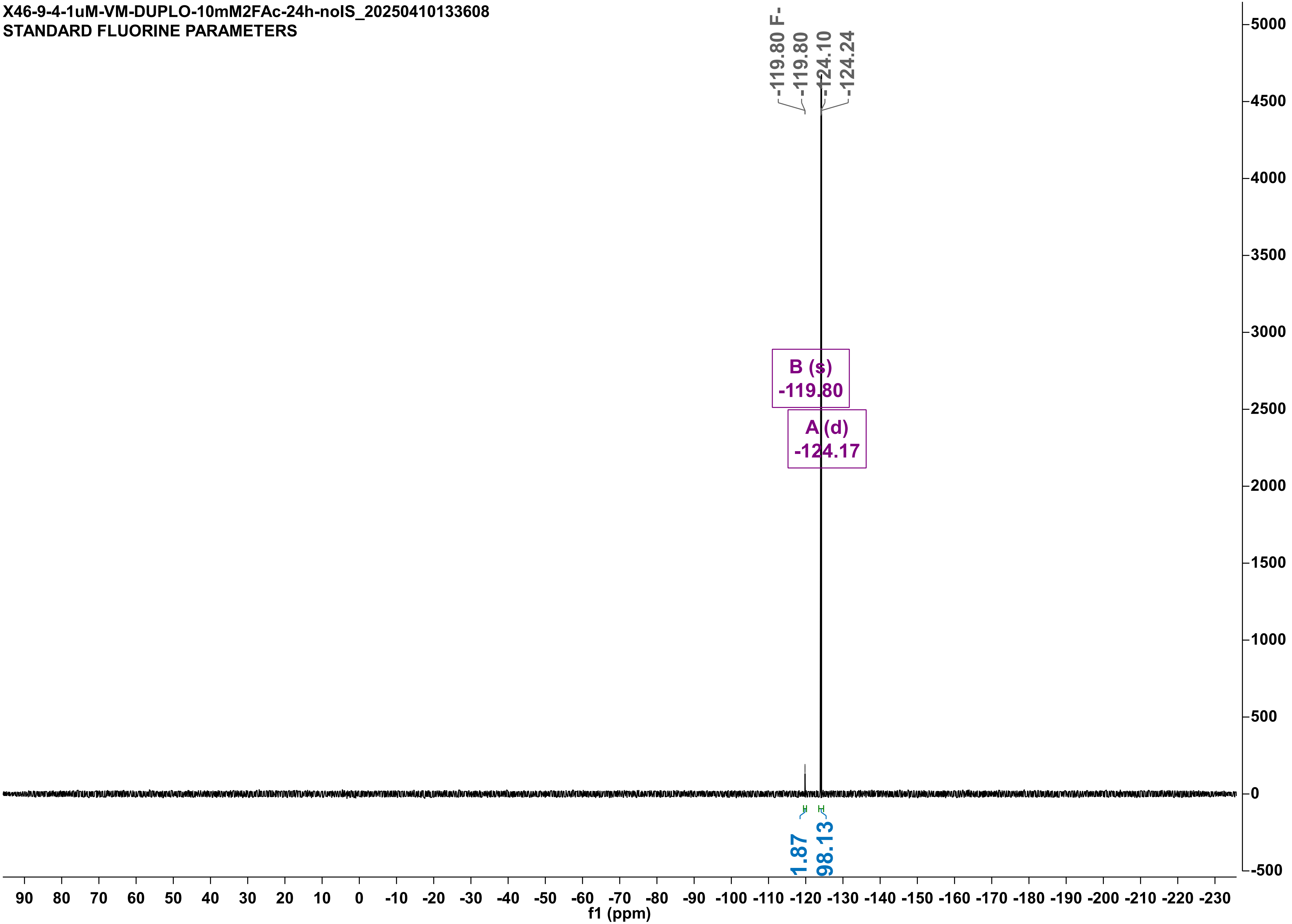

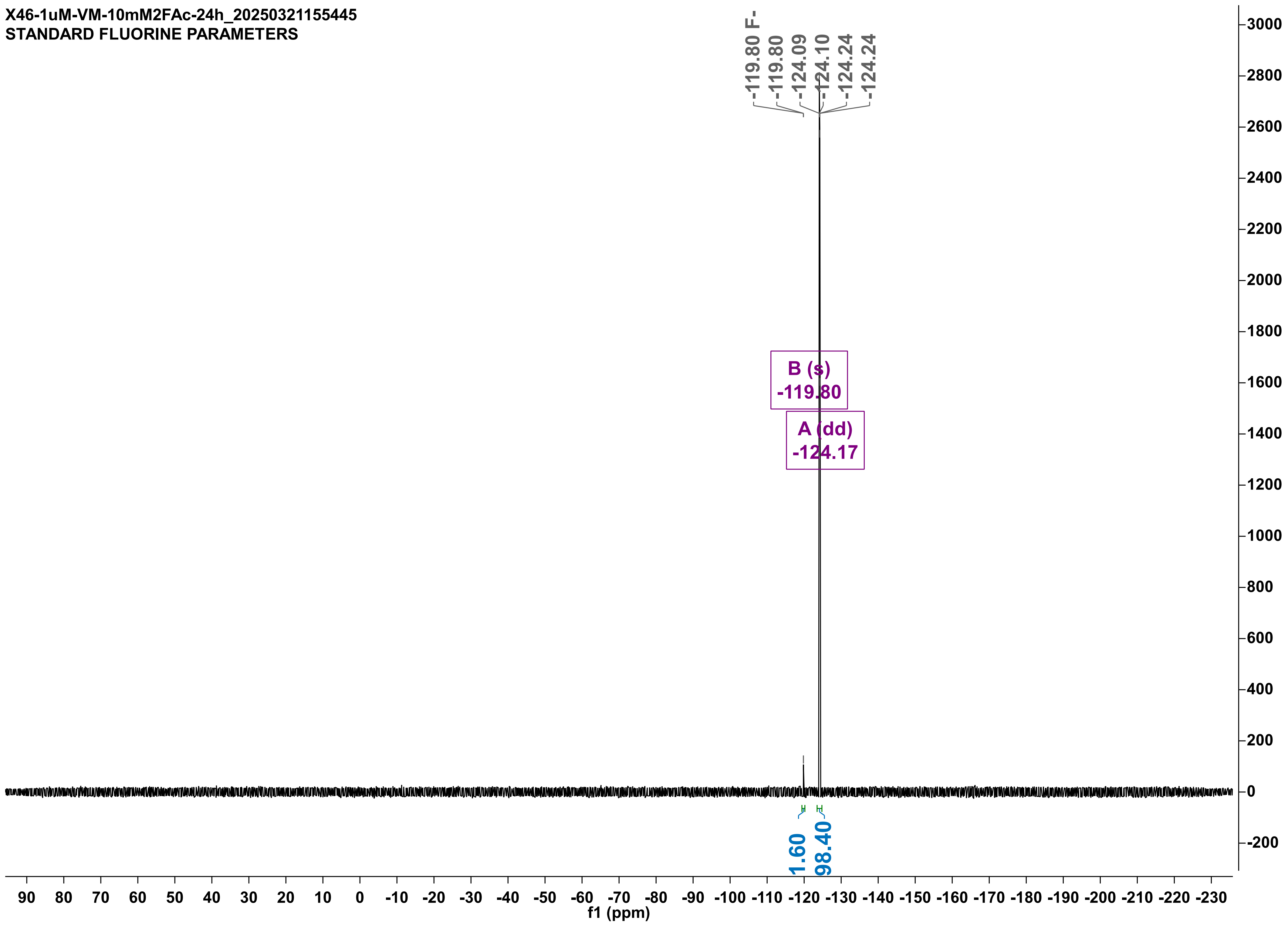

X46-1uM-VM-10mM2FAc-24h_20250321155445
STANDARD FLUORINE PARAMETERS
-119.80 F-
-119.80
-124.09
-124.10
-124.24
-124.24
B (s)
-119.80
A (dd)
-124.17
1.60
98.40
f1 (ppm)

X46-1uMPLM2-10mM2FAc-24h_20250327182014
STANDARD FLUORINE PARAMETERS

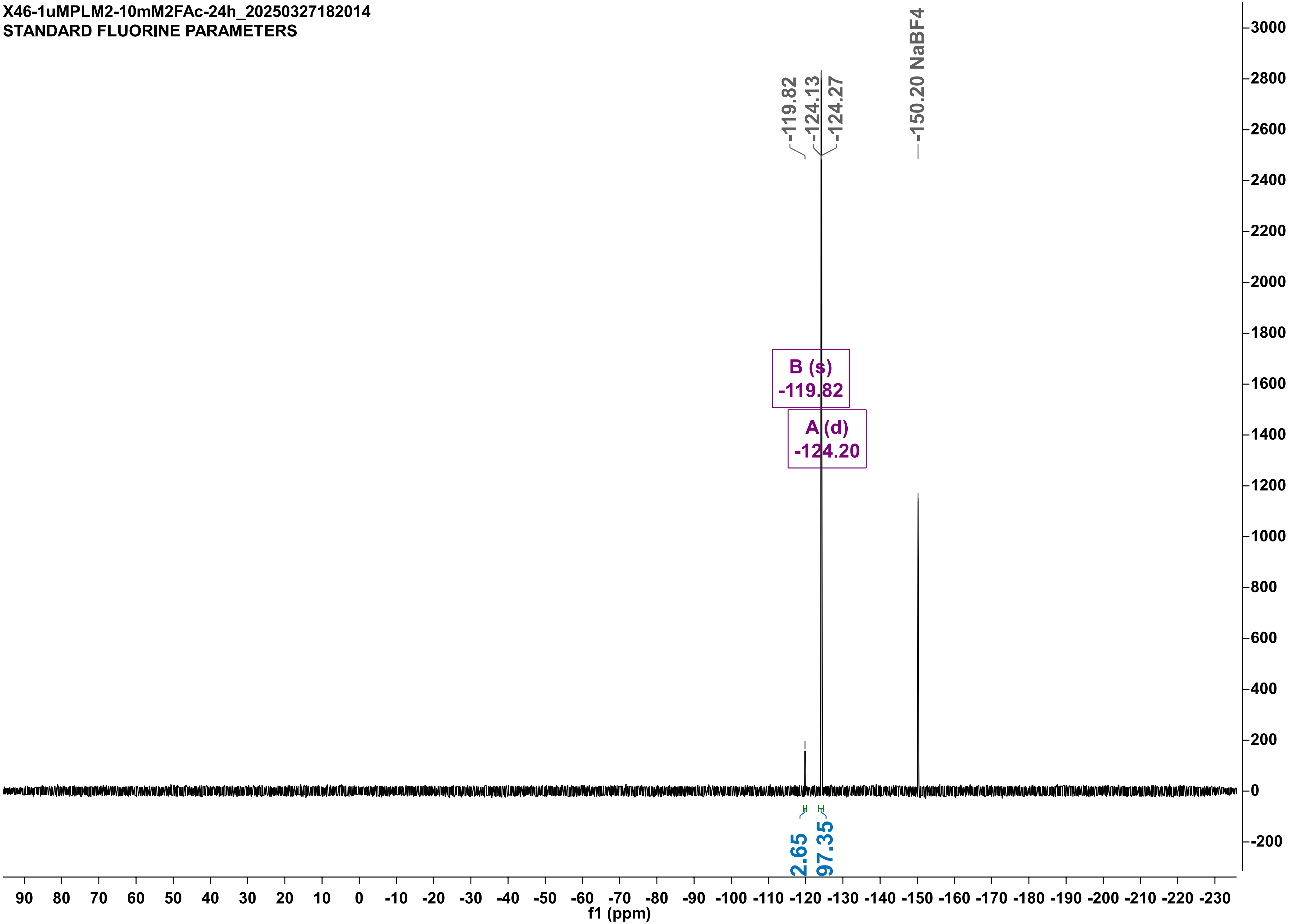

X46-2-1uMPLM2-duplo-10mM2FAc-24H-noIS_20250328184251
STANDARD FLUORINE PARAMETERS

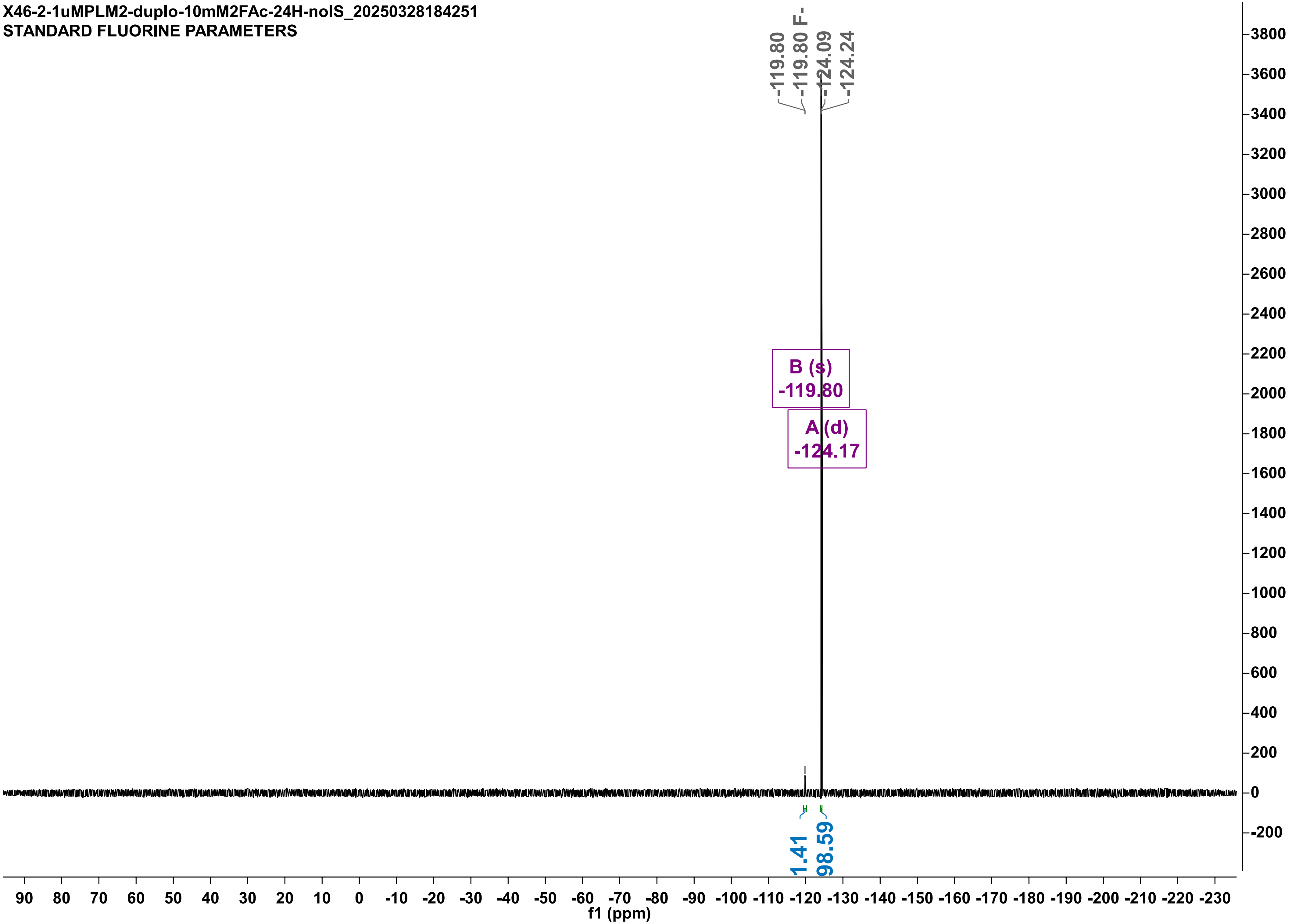

X48-15-4-1uMPLM2-rep-10mM2FAc-24h_20250415175946
STANDARD FLUORINE PARAMETERS

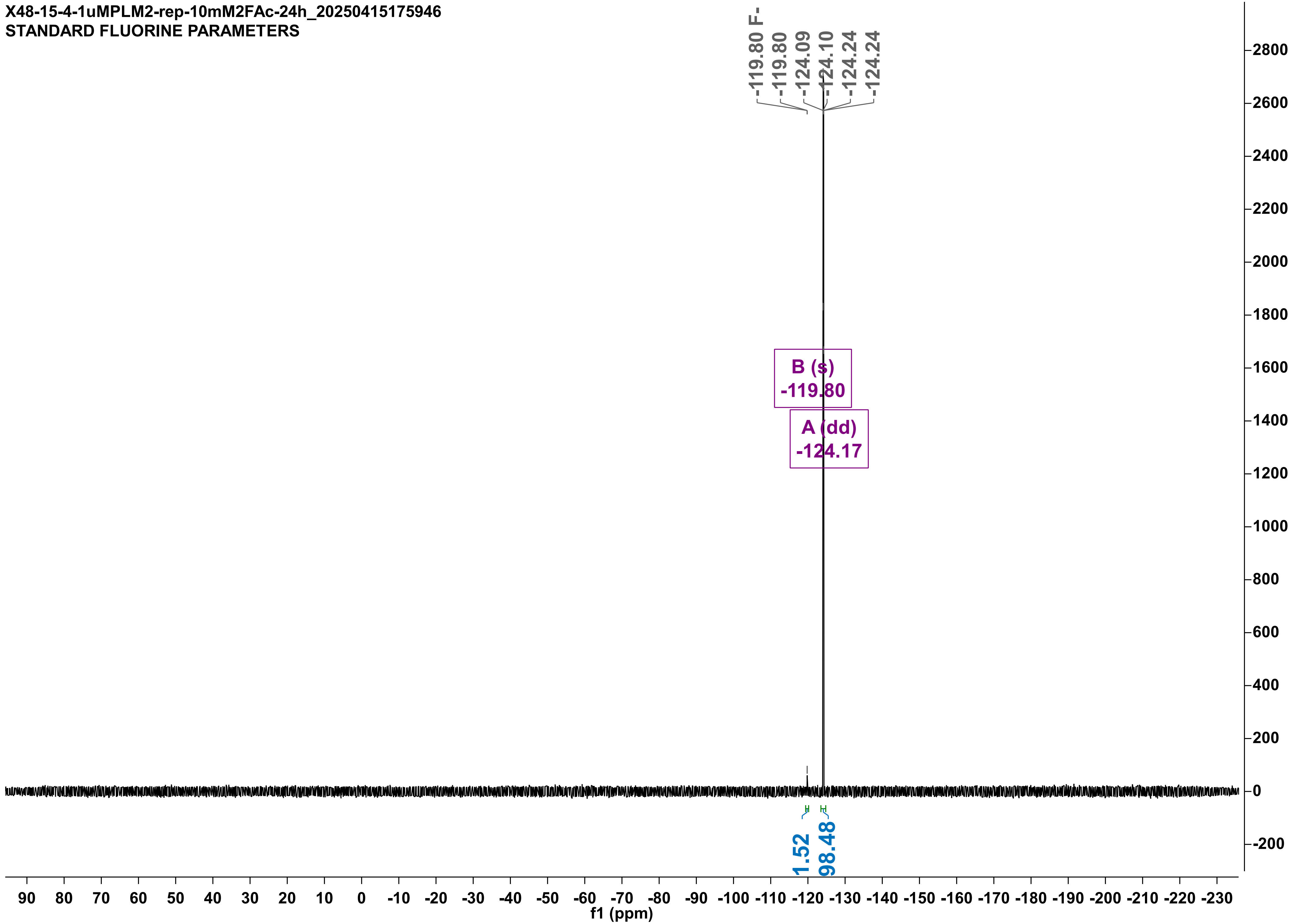

X46-1uM-ML-10mM2FAc-24H_20250327180540
STANDARD FLUORINE PARAMETERS

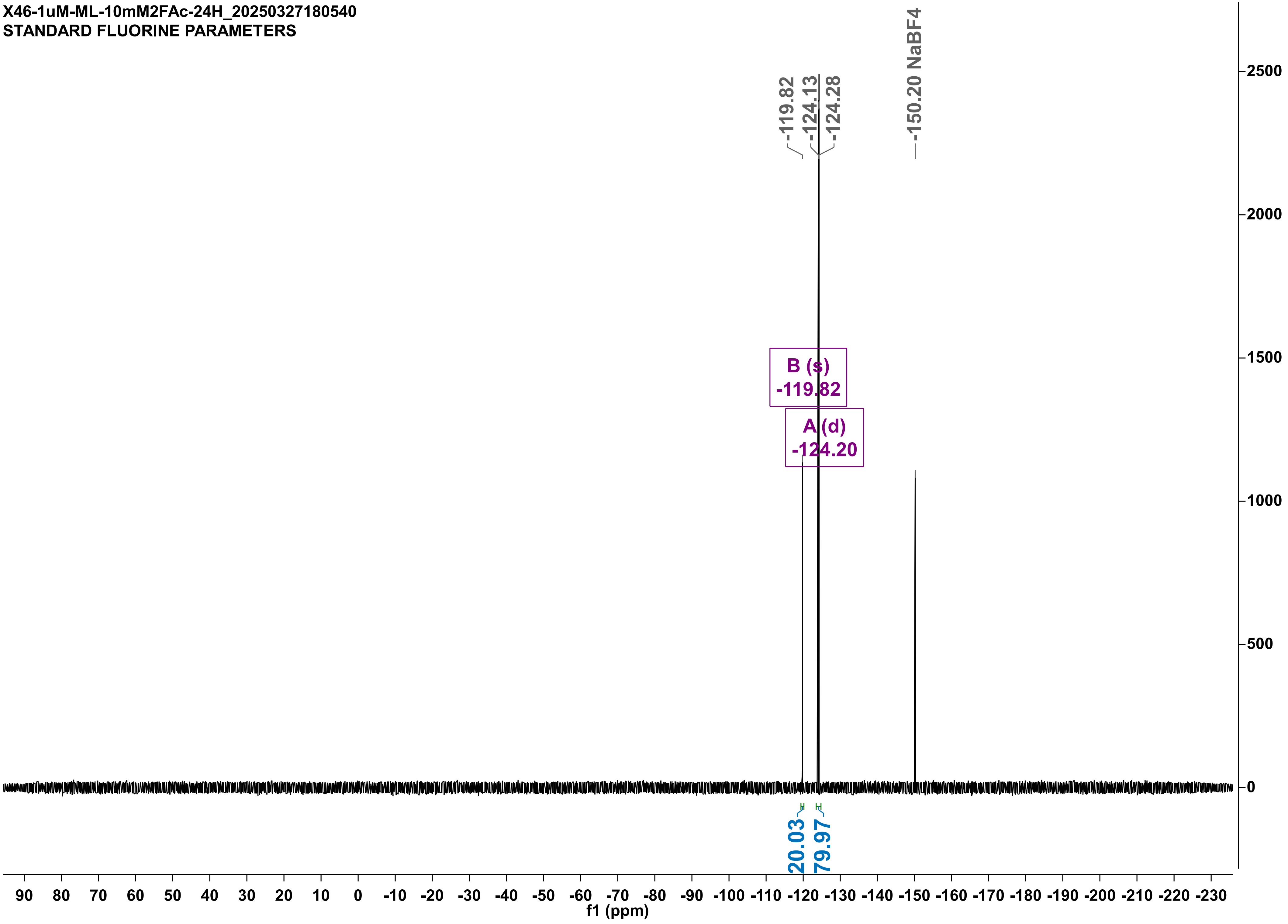

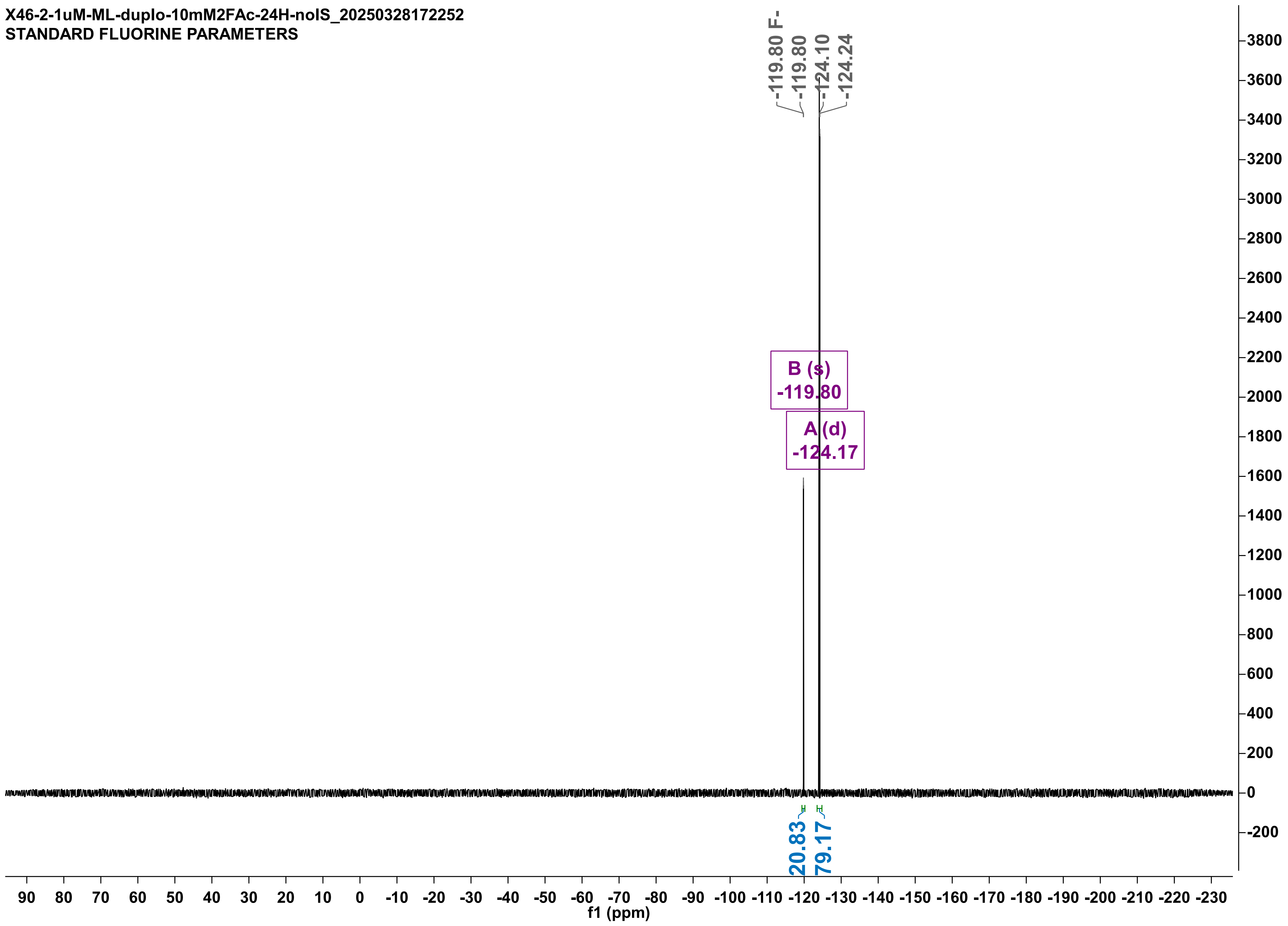
X46-2-1uM-ML-duplo-10mM2FAc-24H-noIS_20250328172252
STANDARD FLUORINE PARAMETERS
-119.80 F-
-119.80
-124.10
-124.24
B (s)
-119.80
A (d)
-124.17
20.83
79.17
f1 (ppm)

X48-15-4-1uM-ML-rep-10mM2FAc-24h_20250415180014
STANDARD FLUORINE PARAMETERS

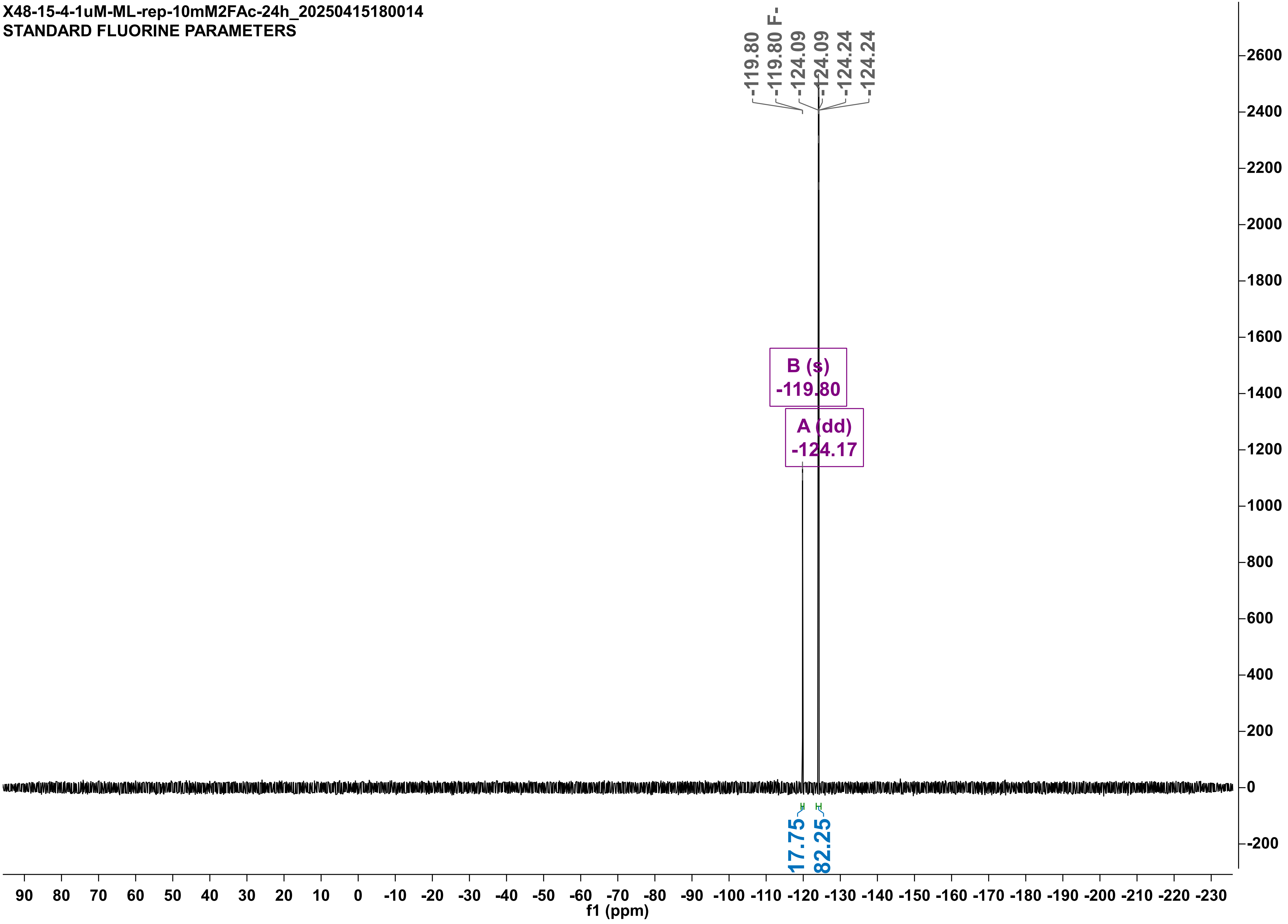

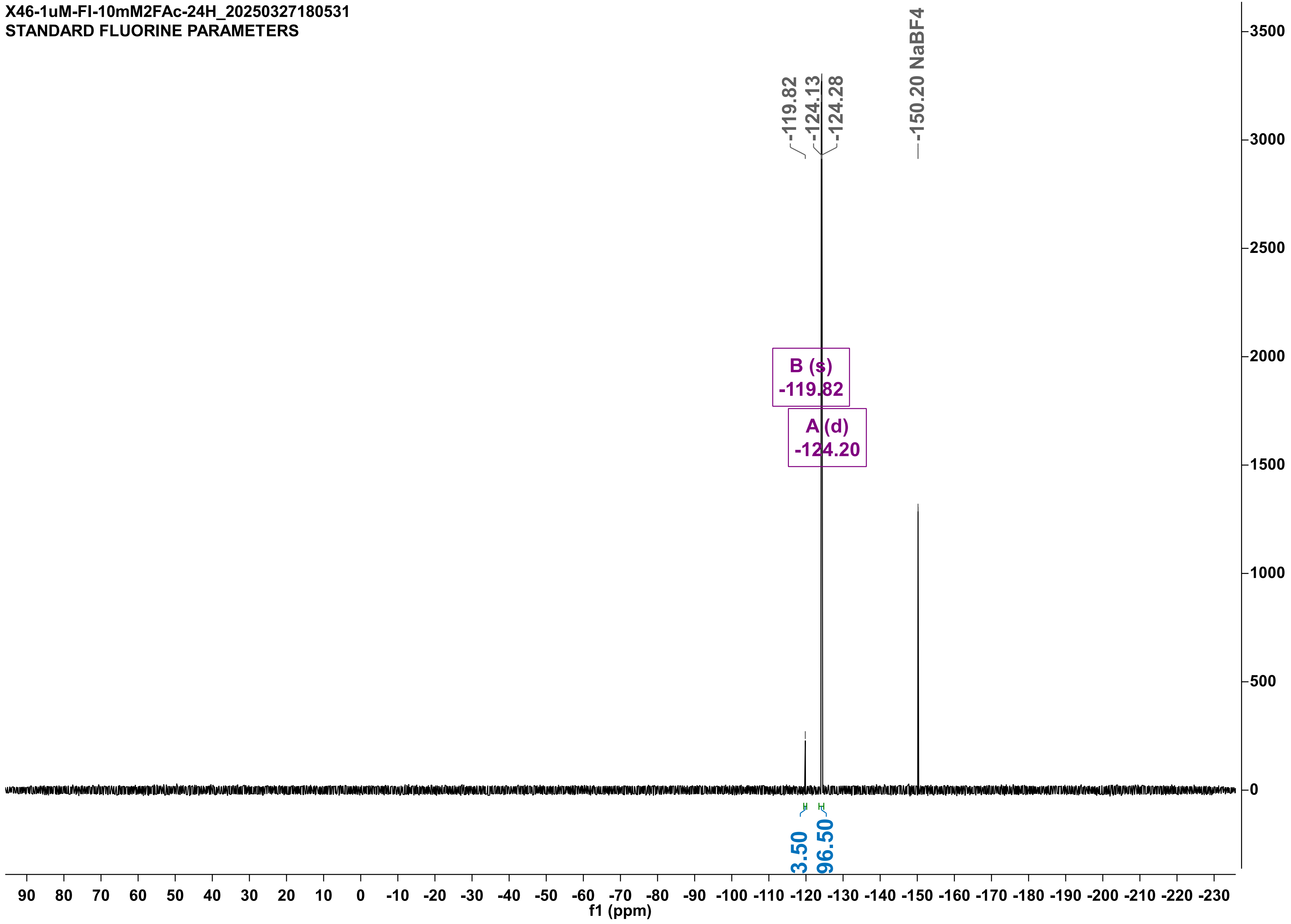

X46-1uM-FI-10mM2FAc-24H_20250327180531
STANDARD FLUORINE PARAMETERS
-119.82
-124.13
-124.28
-150.20 NaBF4
B (s)
-119.82
A (d)
-124.20
3.50
96.50
f1 (ppm)

X46-9-4-1uM-FI-DUPLO-10mM2FAc-24h-noIS_20250410133632
STANDARD FLUORINE PARAMETERS

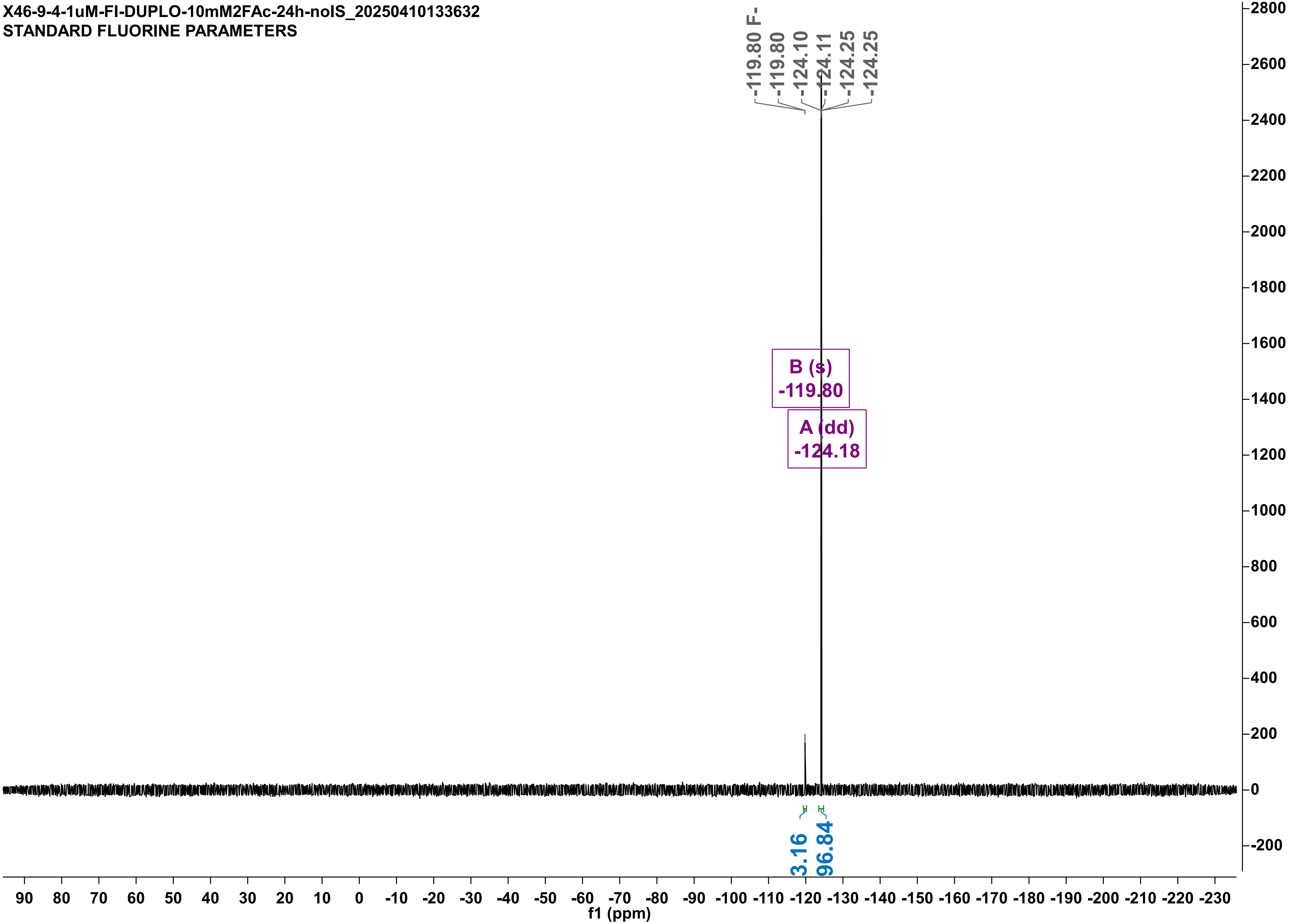

X46-1uMLVM-10mM2FAc-24H_20250327181224
STANDARD FLUORINE PARAMETERS

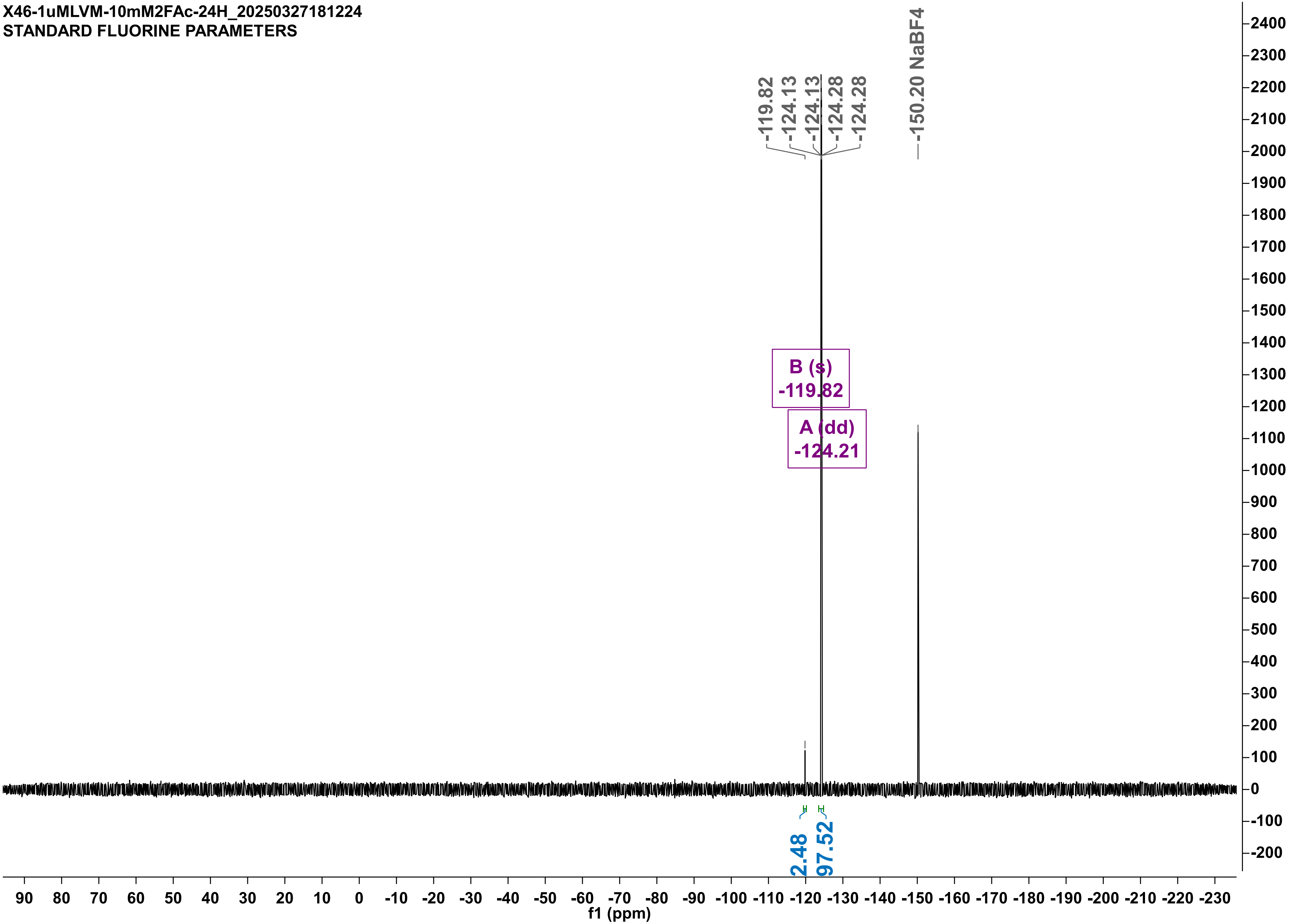

X46-2-1uMLVM-duplo-10mM2FAc-24H-noIS_20250328184256
STANDARD FLUORINE PARAMETERS

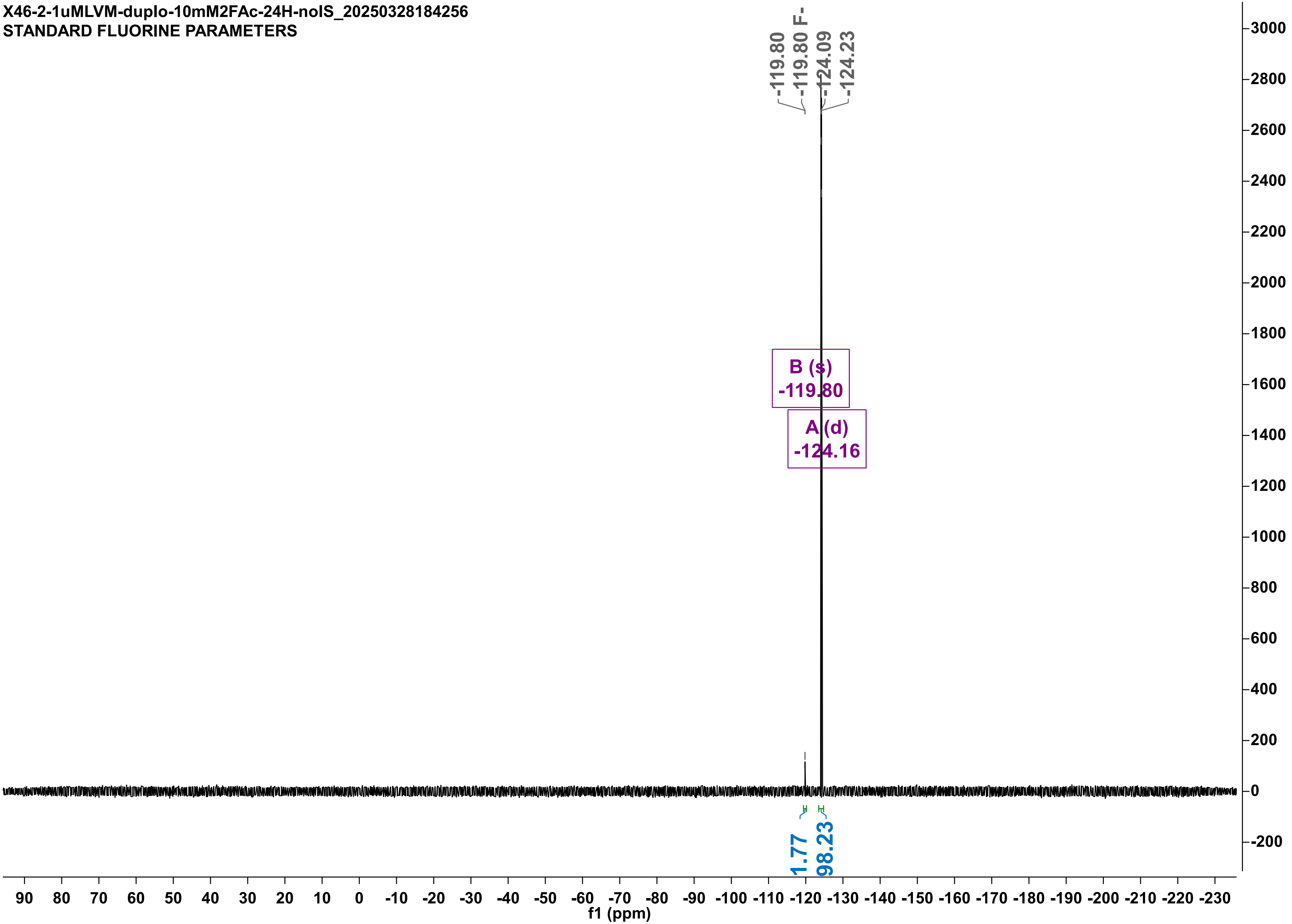

X46-1uMQ245A-10mMF2Ac-24H_20250326185253
STANDARD FLUORINE PARAMETERS

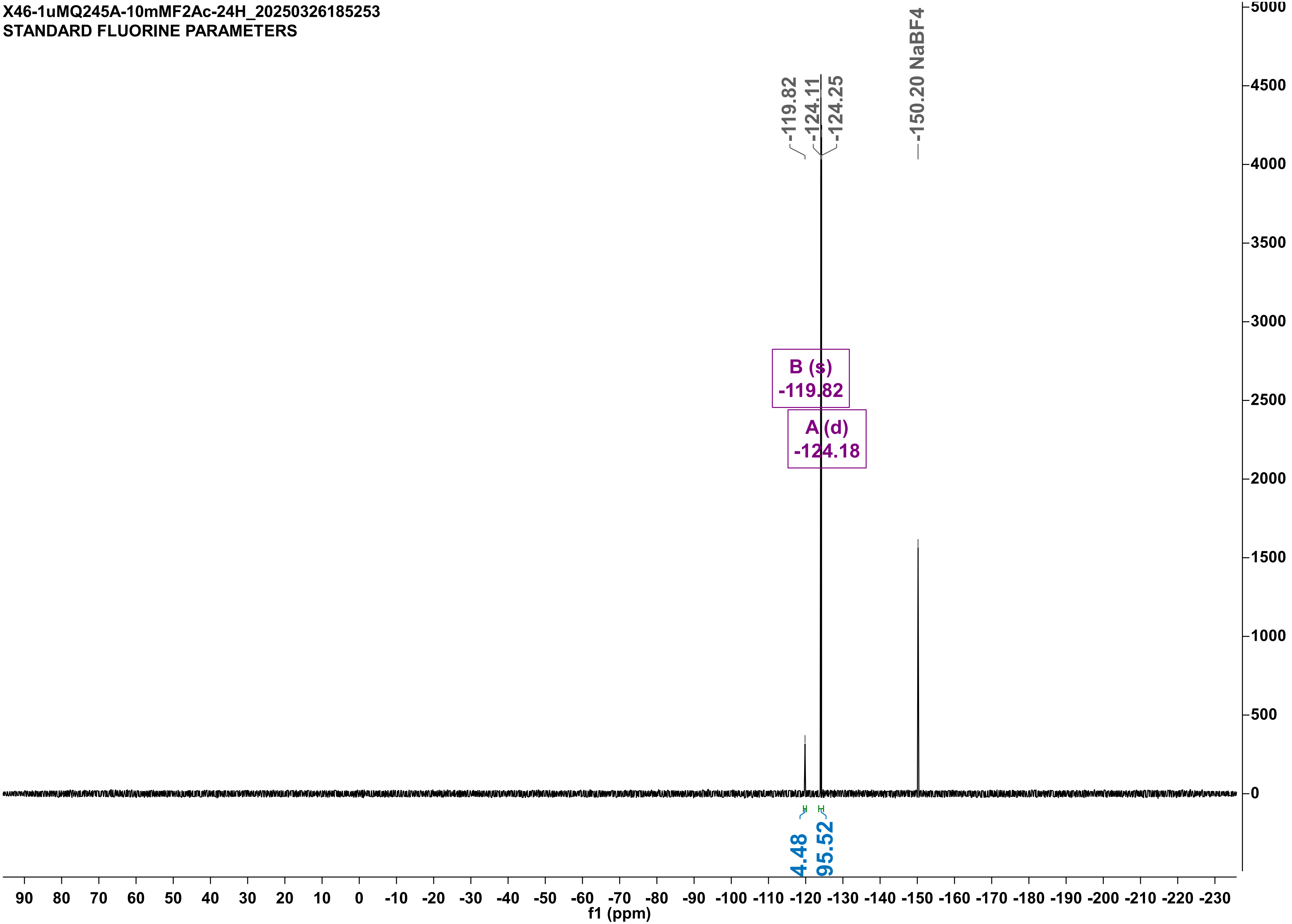

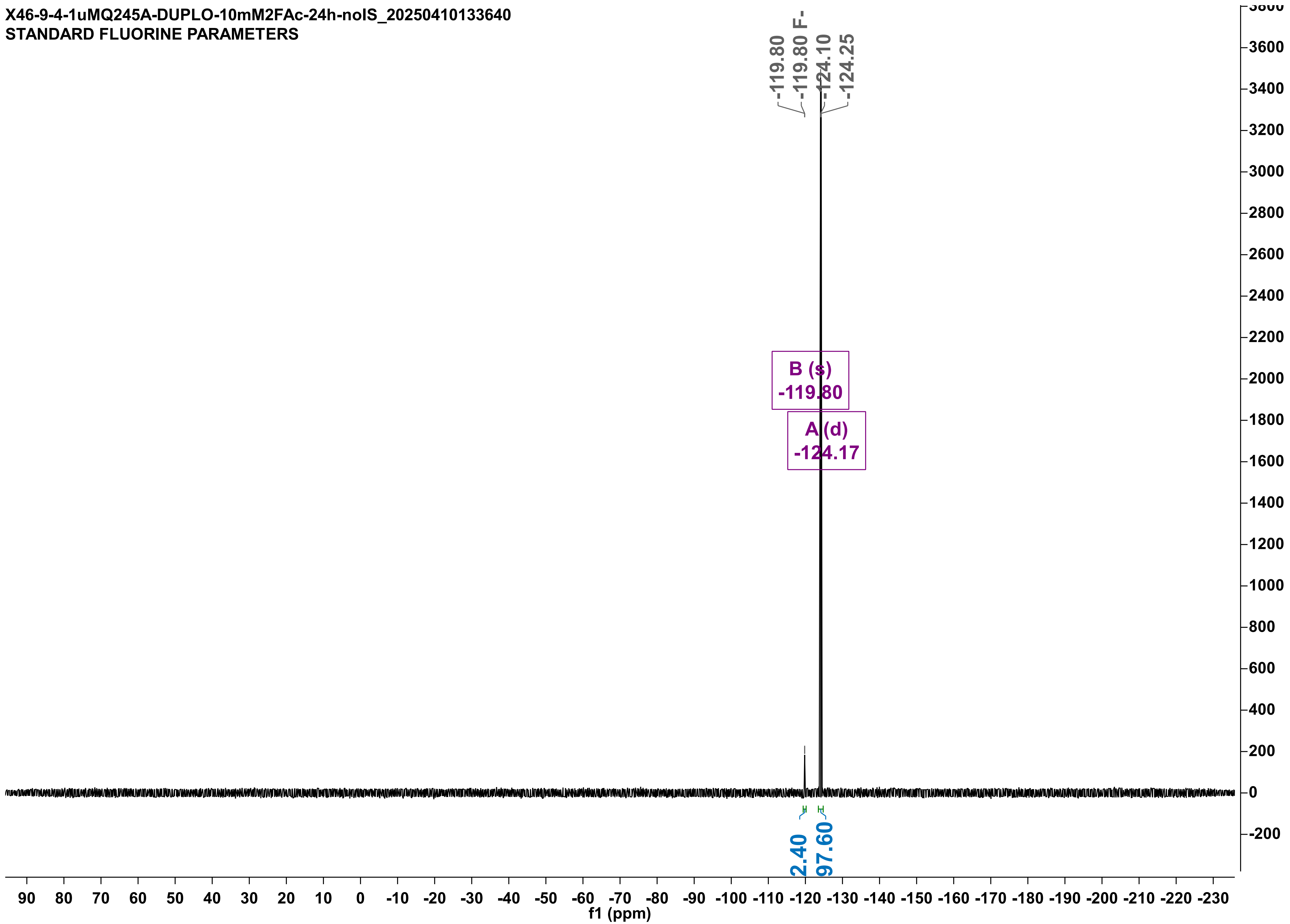

X46-9-4-1uMQ245A-DUPLO-10mM2FAc-24h-noIS_20250410133640
STANDARD FLUORINE PARAMETERS
-119.80
-119.80 F-
-124.10
-124.25
B (s)
-119.80
A (d)
-124.17
2.40
97.60
90 80 70 60 50 40 30 20 10 0 -10 -20 -30 -40 -50 -60 -70 -80 -90 -100 -110 -120 -130 -140 -150 -160 -170 -180 -190 -200 -210 -220 -230
f1 (ppm)
3600 3400 3200 3000 2800 2600 2400 2200 2000 1800 1600 1400 1200 1000 800 600 400 200 0 -200

X46-1uMQ245G-10mMF2Ac-24H_20250326191725
STANDARD FLUORINE PARAMETERS

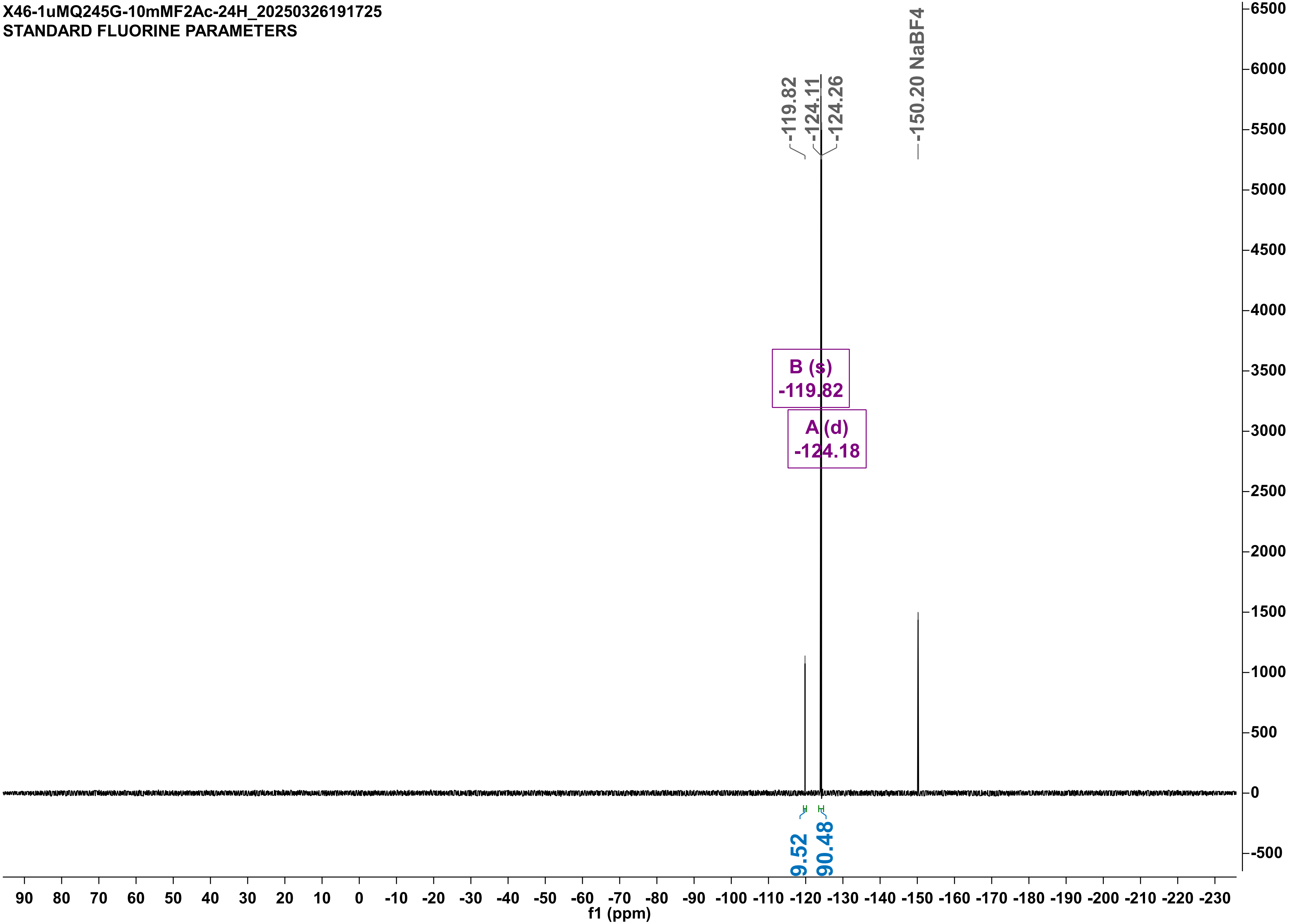

X46-9-4-1uMQ245G-DUPLO-10mM2FAc-24h-noIS_20250410133648
STANDARD FLUORINE PARAMETERS

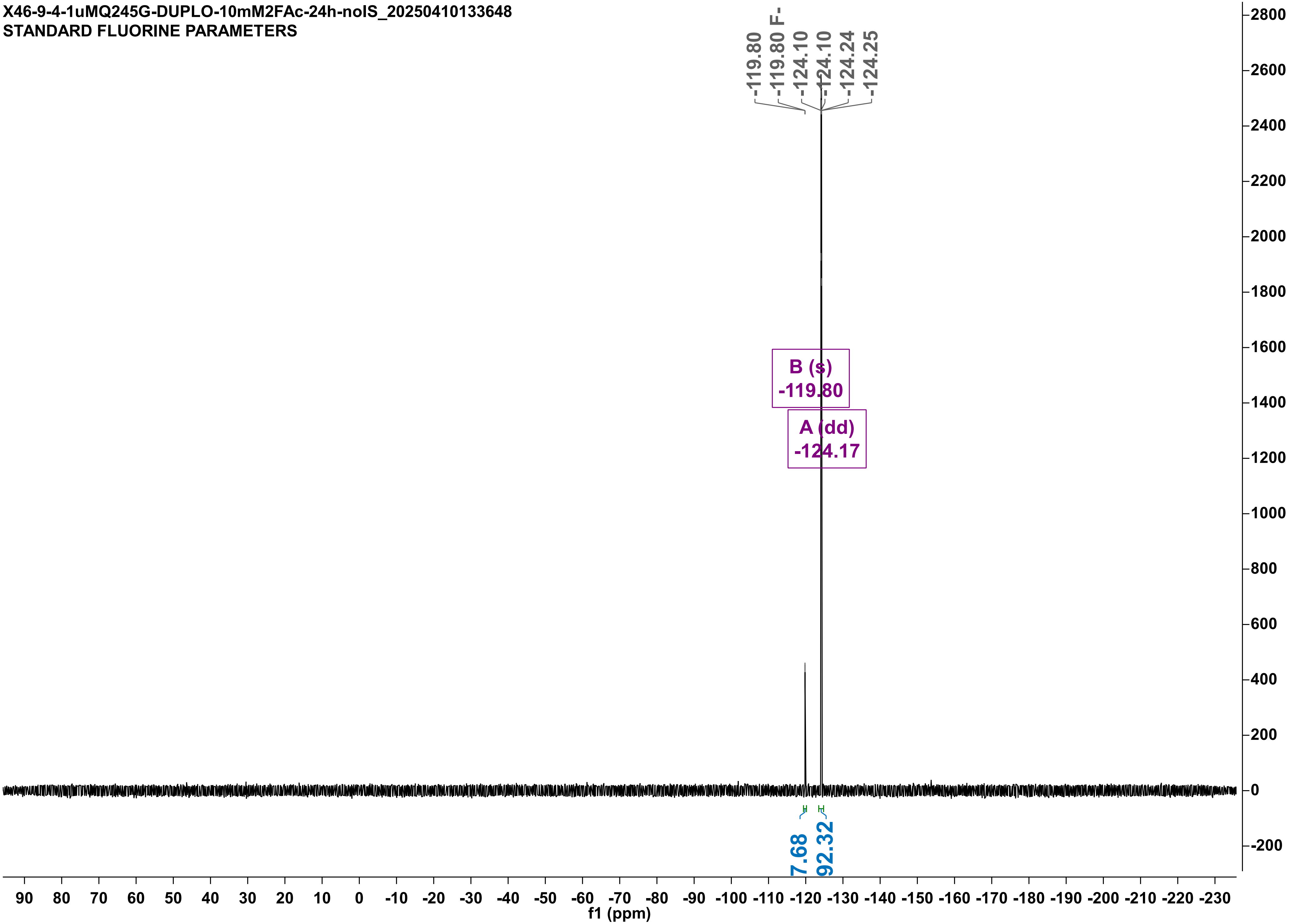

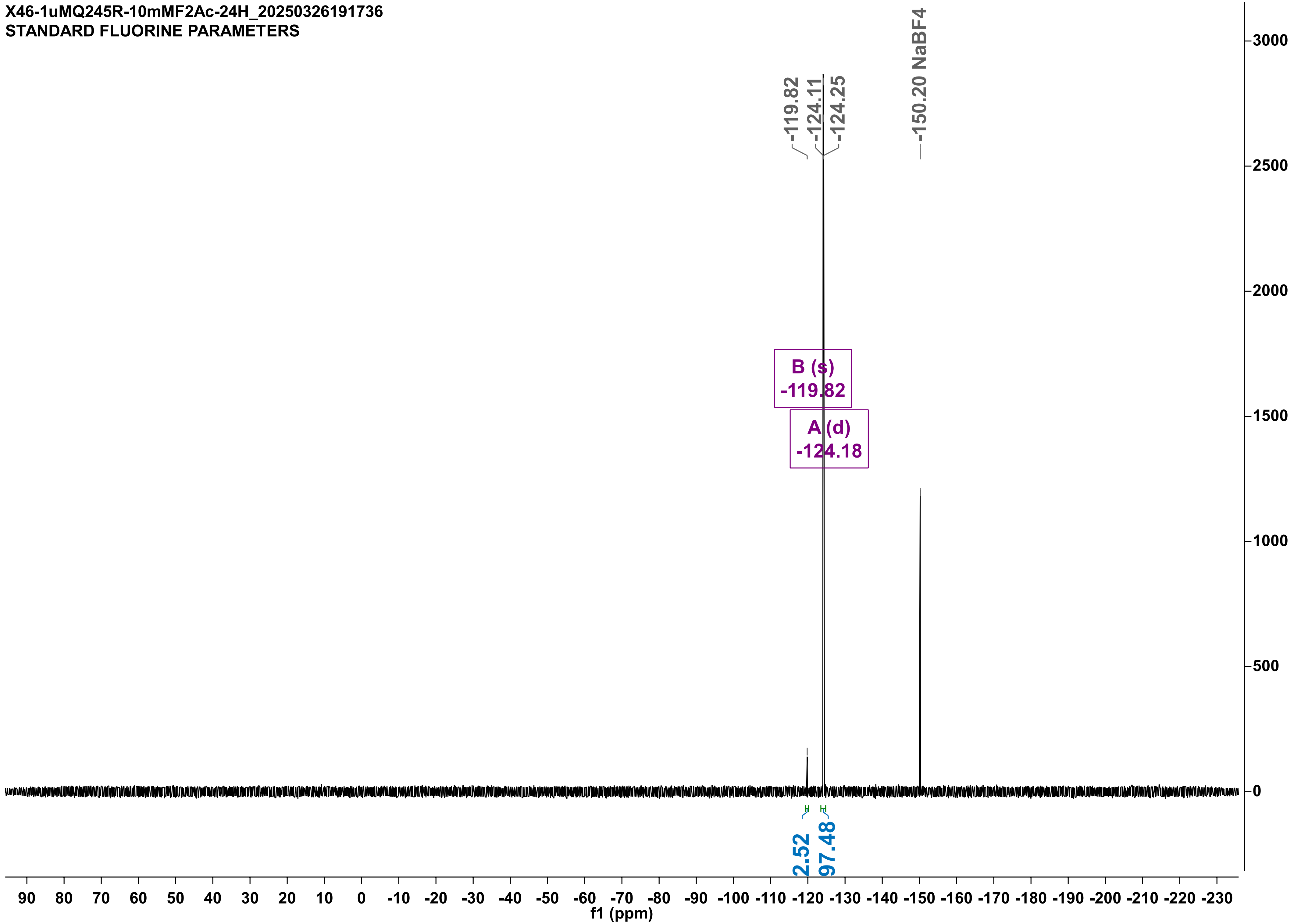

X46-1uMQ245R-10mMF2Ac-24H_20250326191736
STANDARD FLUORINE PARAMETERS
-119.82
-124.11
-124.25
-150.20 NaBF4
B (s)
-119.82
A (d)
-124.18
2.52
97.48
3000
2500
2000
1500
1000
500
0
90 80 70 60 50 40 30 20 10 0 -10 -20 -30 -40 -50 -60 -70 -80 -90 -100 -110 -120 -130 -140 -150 -160 -170 -180 -190 -200 -210 -220 -230
f1 (ppm)

X46-9-4-1uMQ245R-DUPLO-10mM2FAc-24h-noIS_20250410133653
STANDARD FLUORINE PARAMETERS

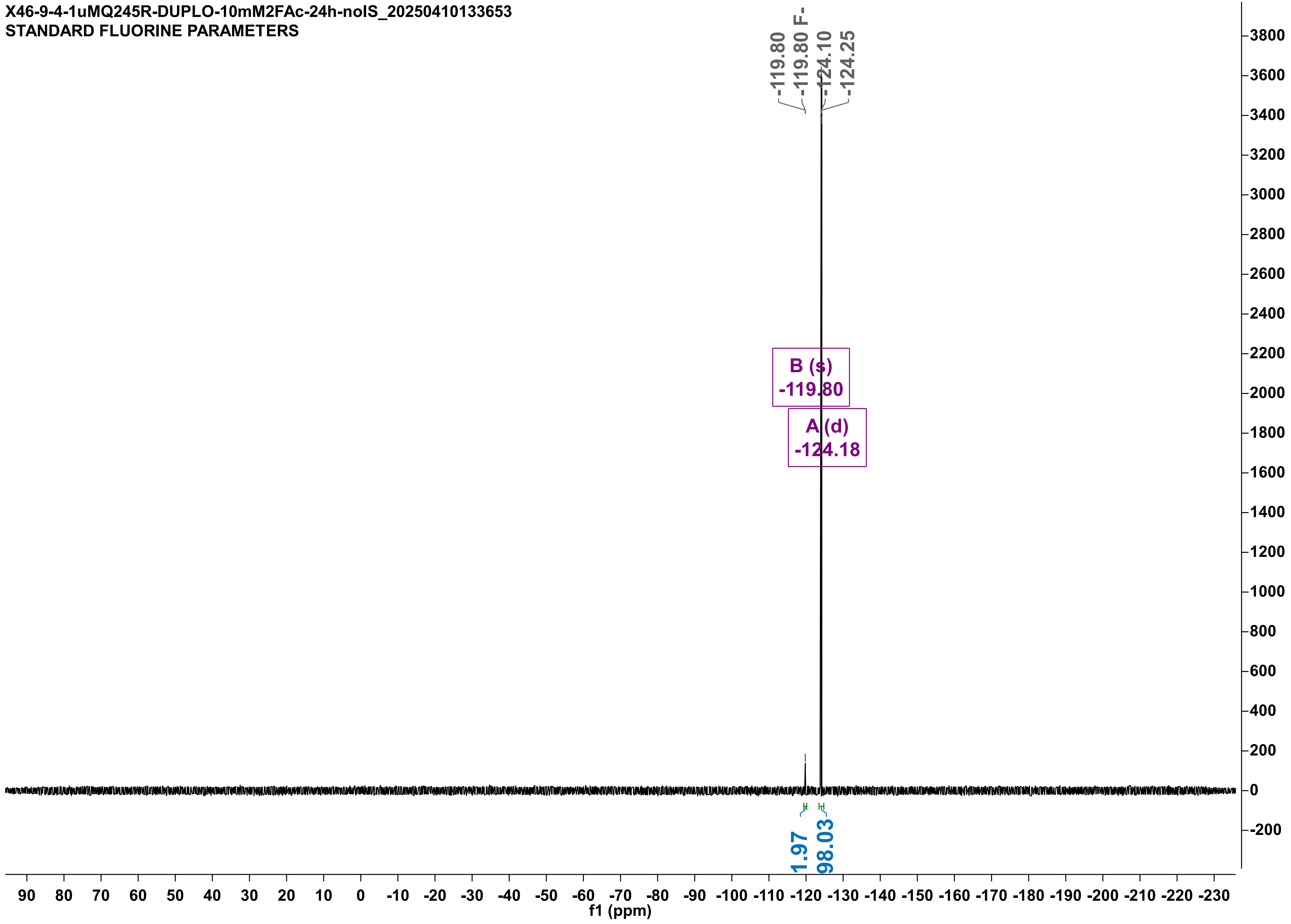

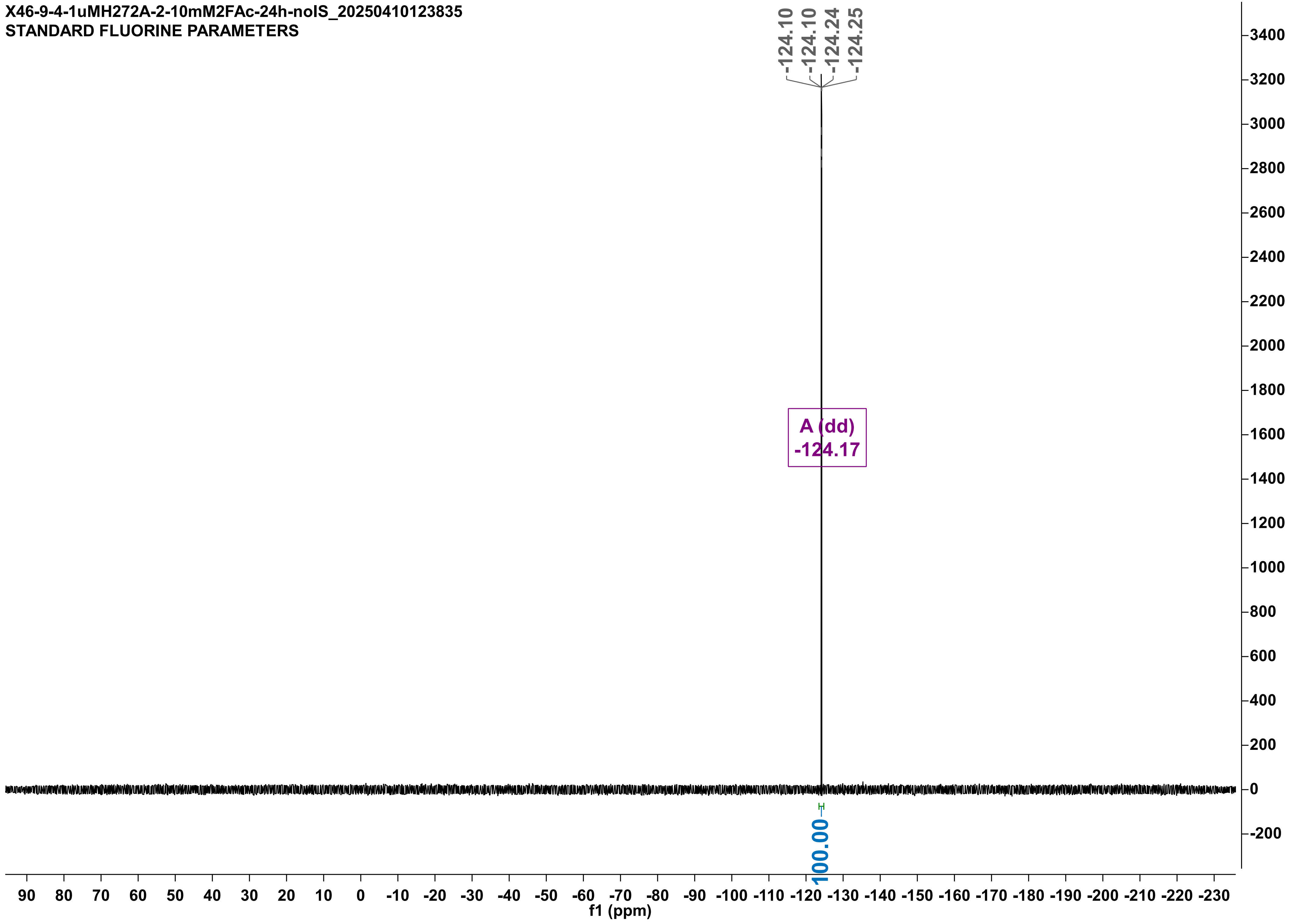

X46-9-4-1uMH272A-2-10mM2FAc-24h-noIS_20250410123835
STANDARD FLUORINE PARAMETERS
-124.10
-124.10
-124.24
-124.25
A (dd)
-124.17
100.00
f1 (ppm)

X46-9-4-noenz-10mM2FAc-24h-noIS_20250410123746
STANDARD FLUORINE PARAMETERS

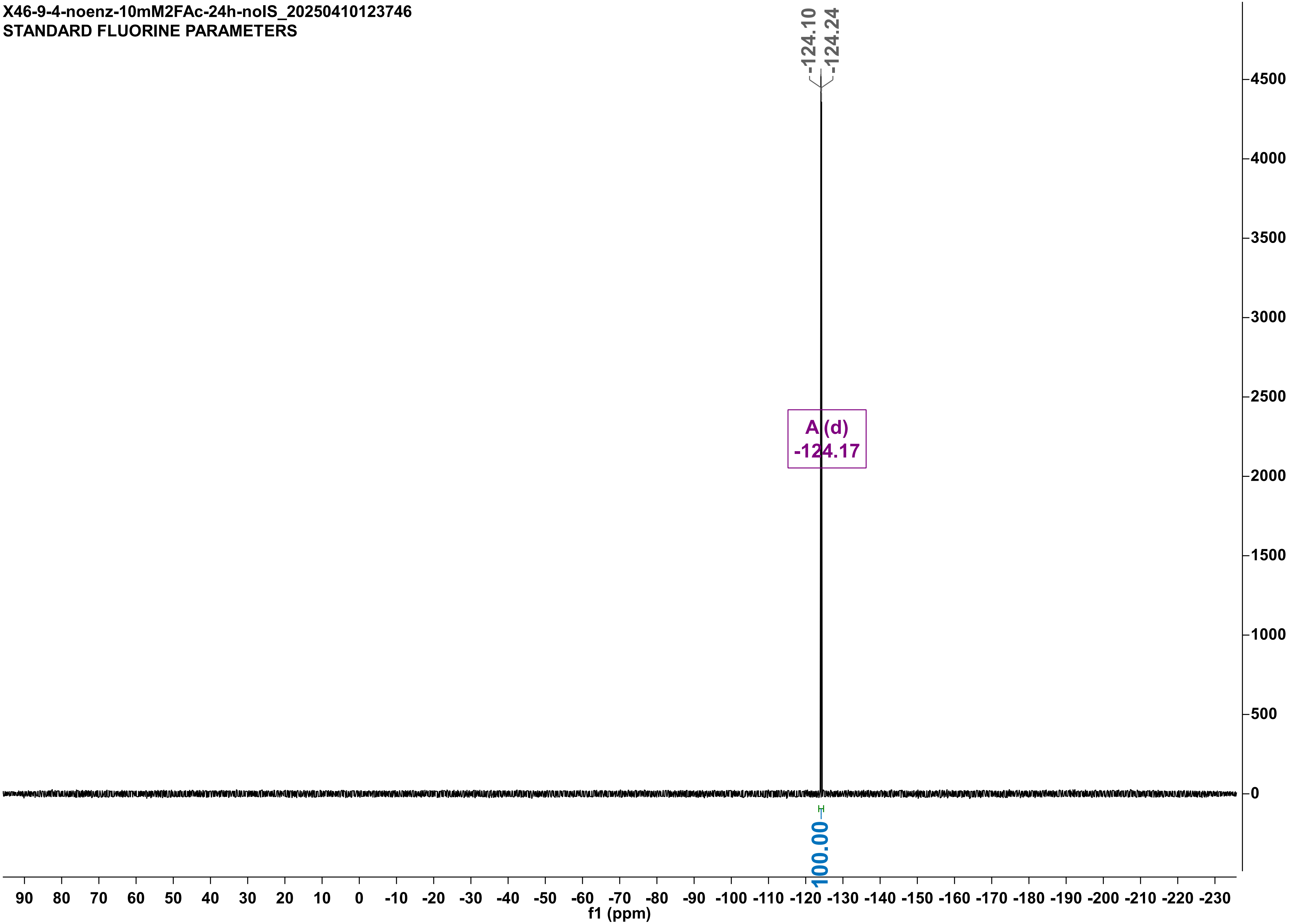

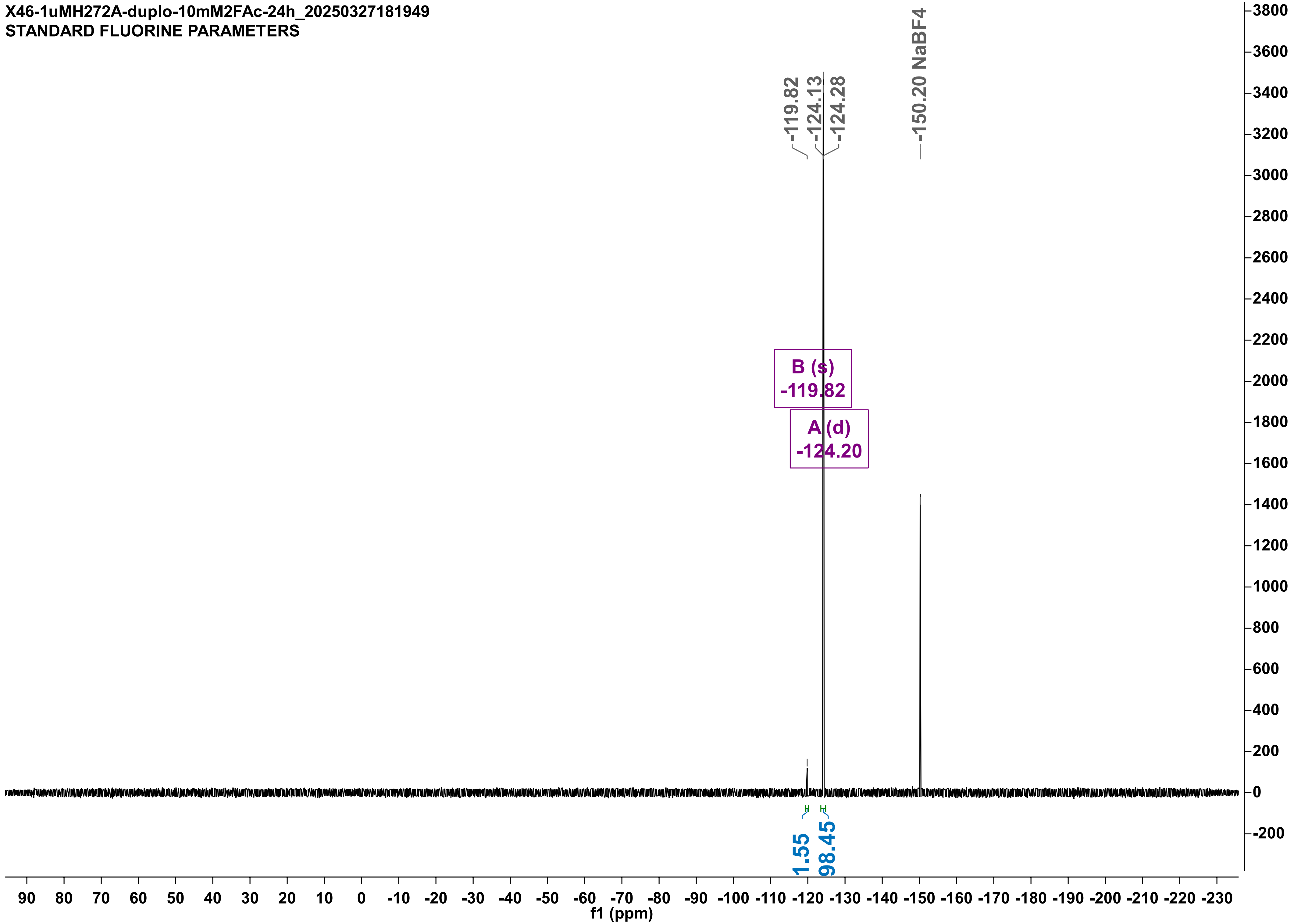

X46-1uMH272A-duplo-10mM2FAc-24h_20250327181949
STANDARD FLUORINE PARAMETERS
-119.82
-124.13
-124.28
-150.20 NaBF4
B (s)
-119.82
A (d)
-124.20
1.55
98.45
f1 (ppm)

X49-17-4-NOENZ-10mM2FPA-time24h_20250417143022
STANDARD FLUORINE PARAMETERS

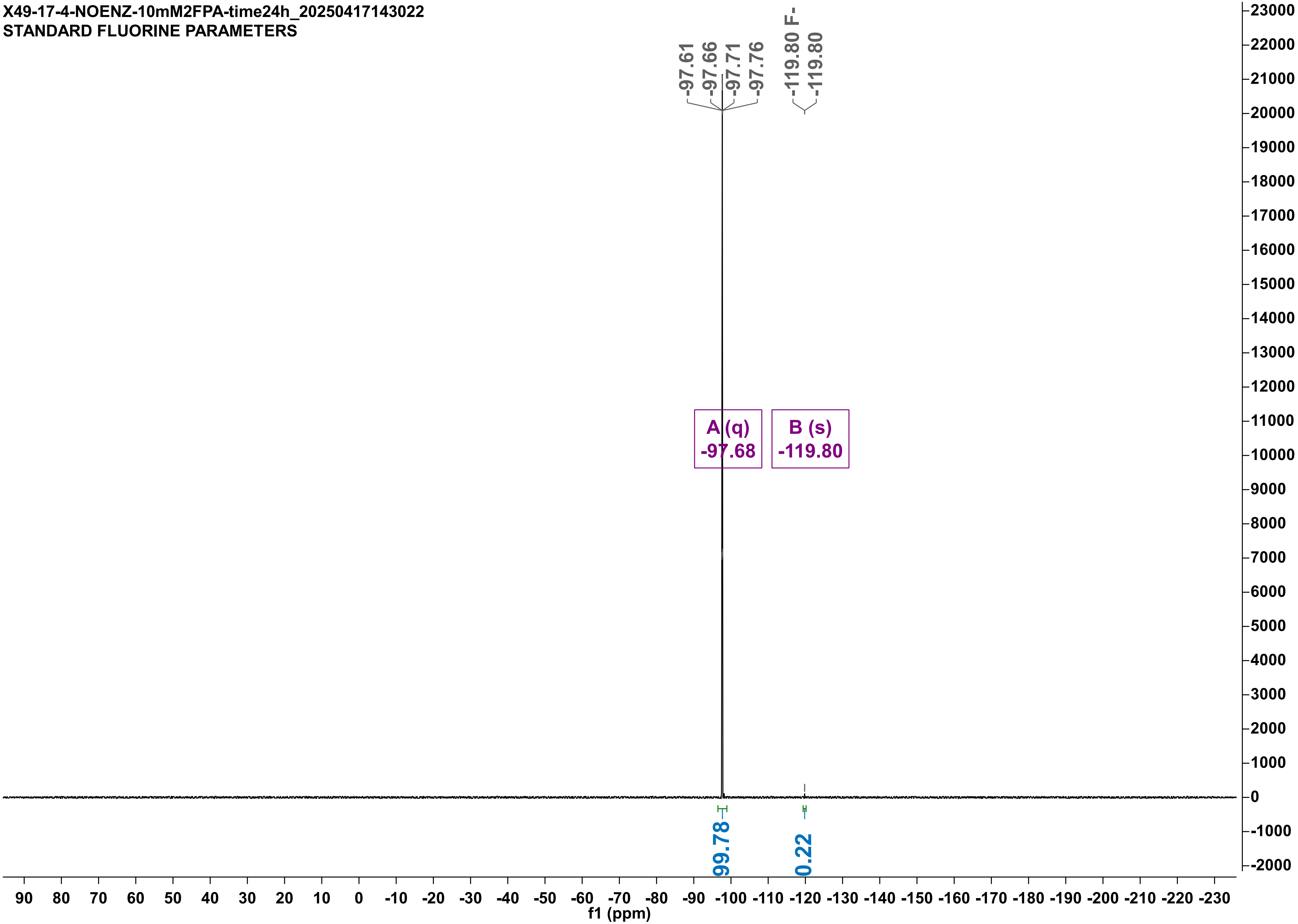

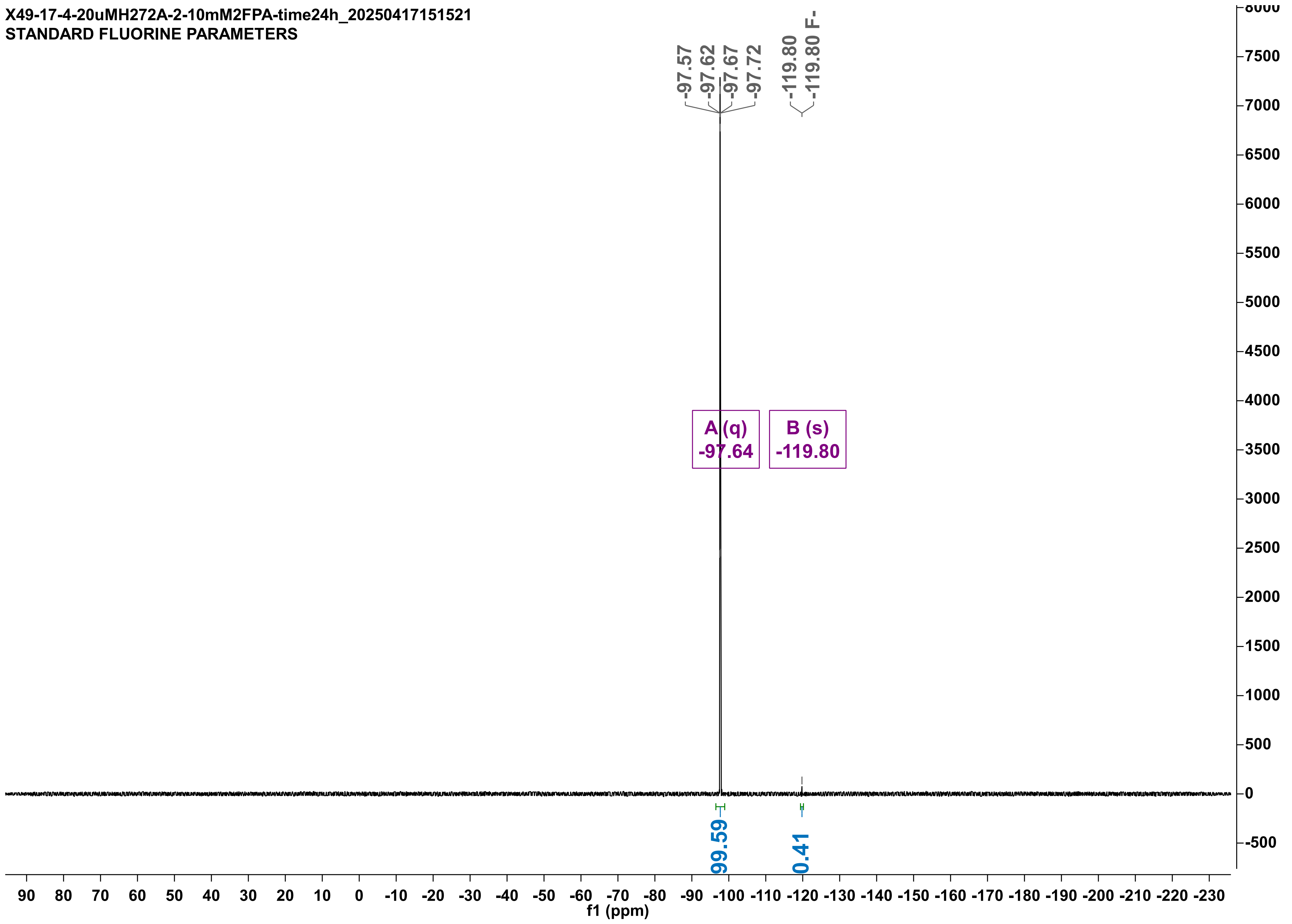

X49-17-4-20uMH272A-2-10mM2FPA-time24h_20250417151521
STANDARD FLUORINE PARAMETERS
-97.57
-97.62
-97.67
-97.72
-119.80
-119.80 F-
A (q)
-97.64
B (s)
-119.80
99.59
0.41
f1 (ppm)

X49-17-4-20uMWT1-10mM2FPA-time24h_20250417151701
STANDARD FLUORINE PARAMETERS

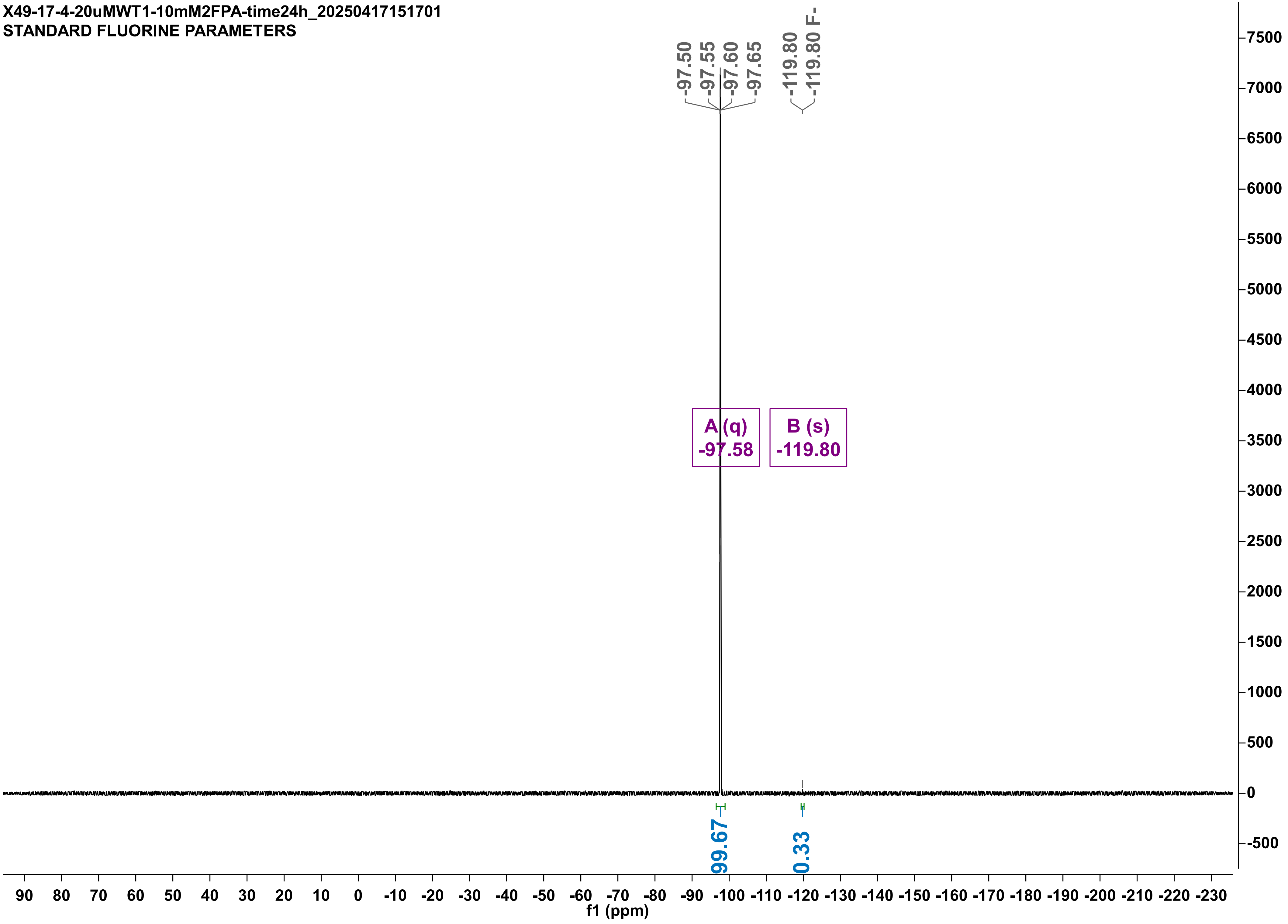

X49-17-4-20uMWT2-10mM2FPA-time24h_20250417161746
STANDARD FLUORINE PARAMETERS

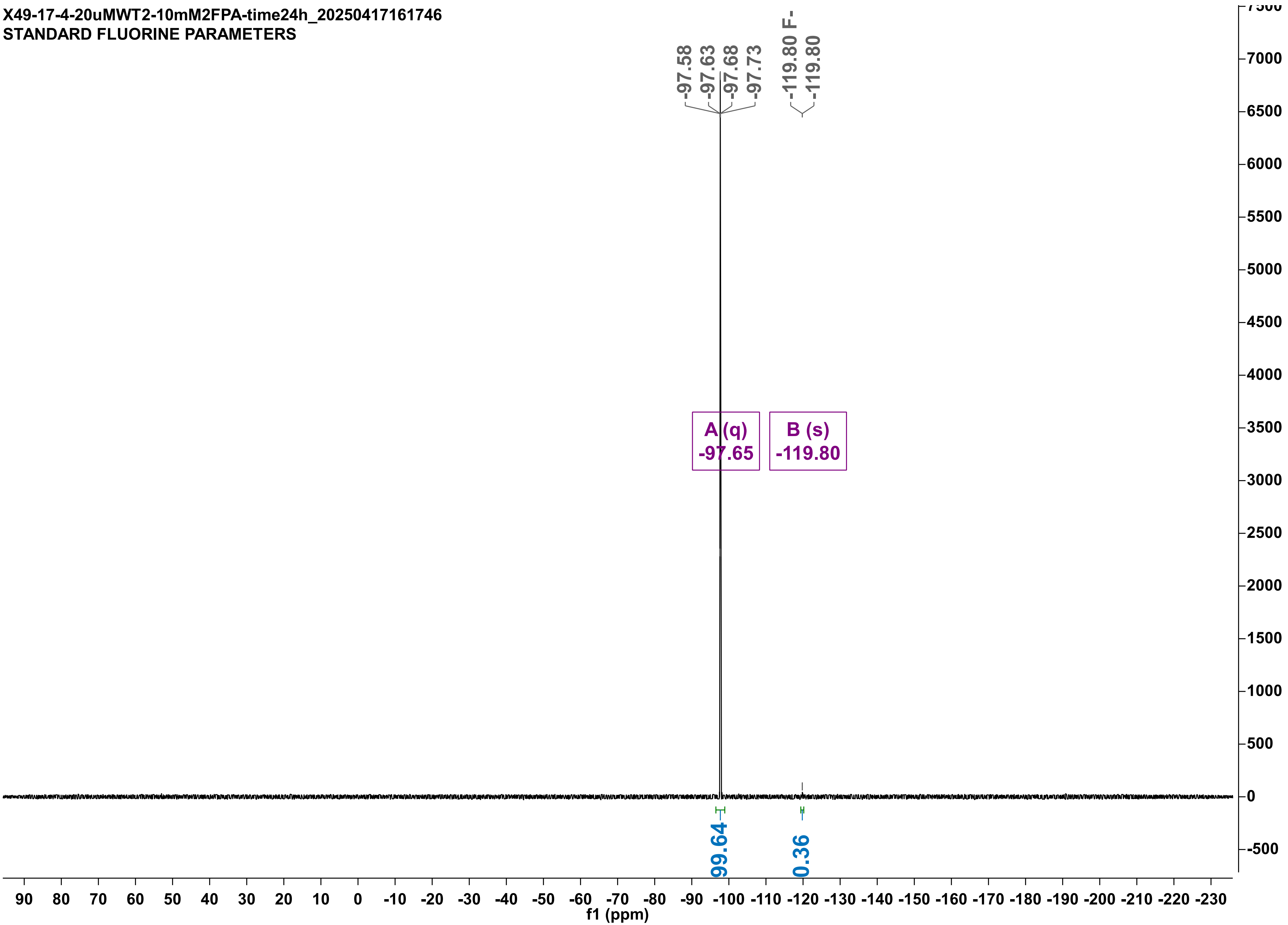

X46-20uMVW--10mMF2PA-24h_20250319165040
STANDARD FLUORINE PARAMETERS

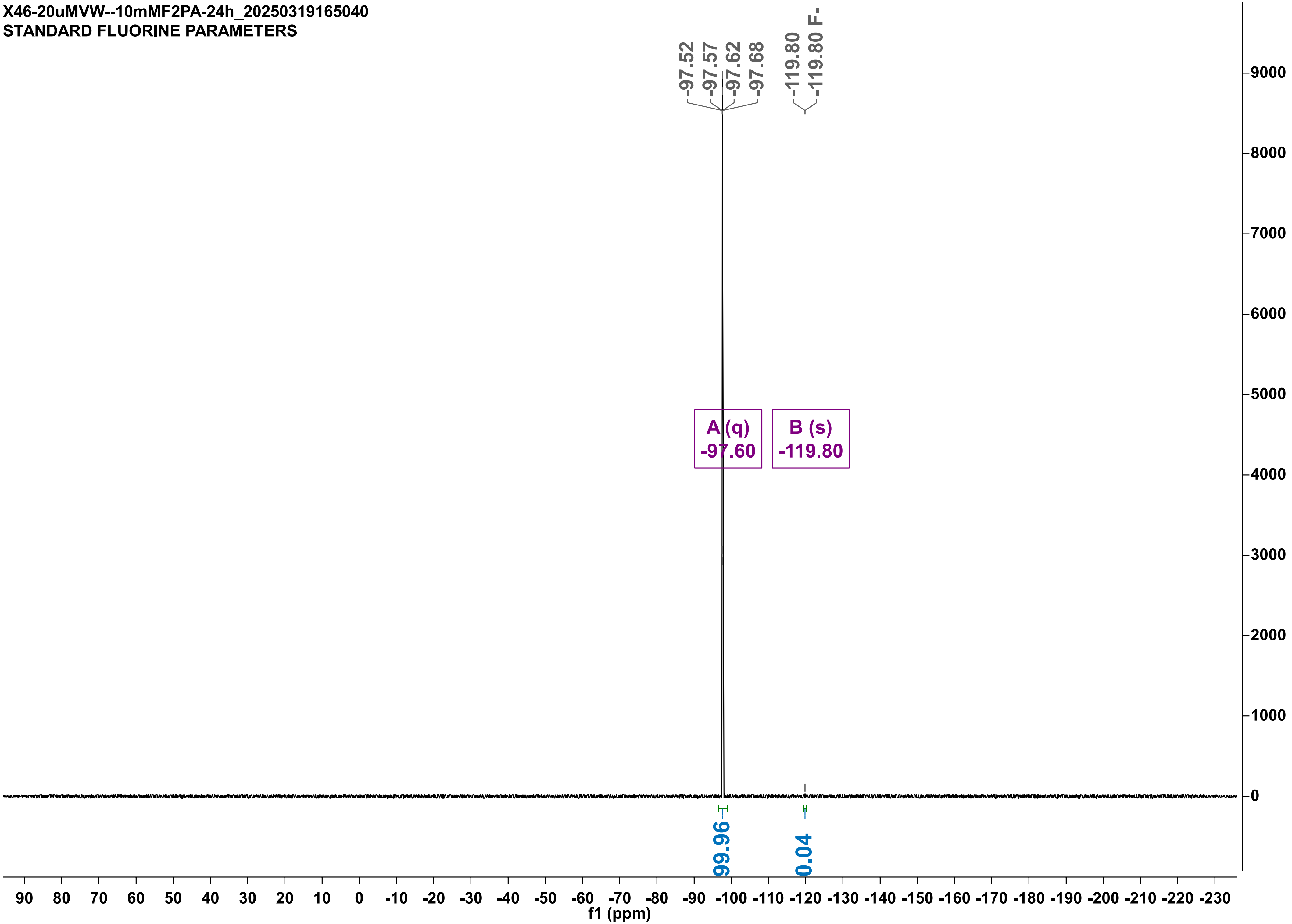

X49-17-4-20uMLW--10mM2FPA-time24h_20250417151555
STANDARD FLUORINE PARAMETERS

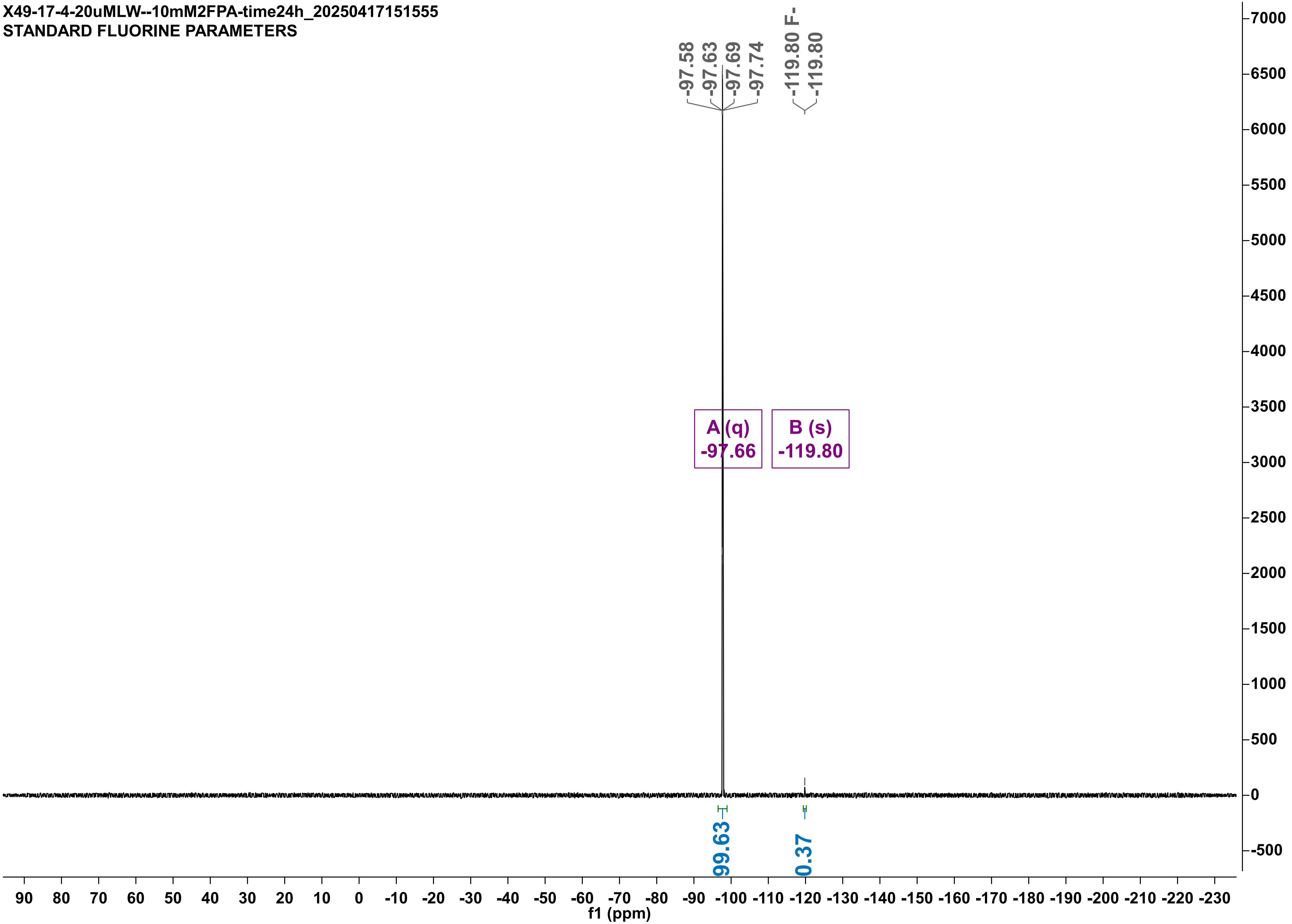

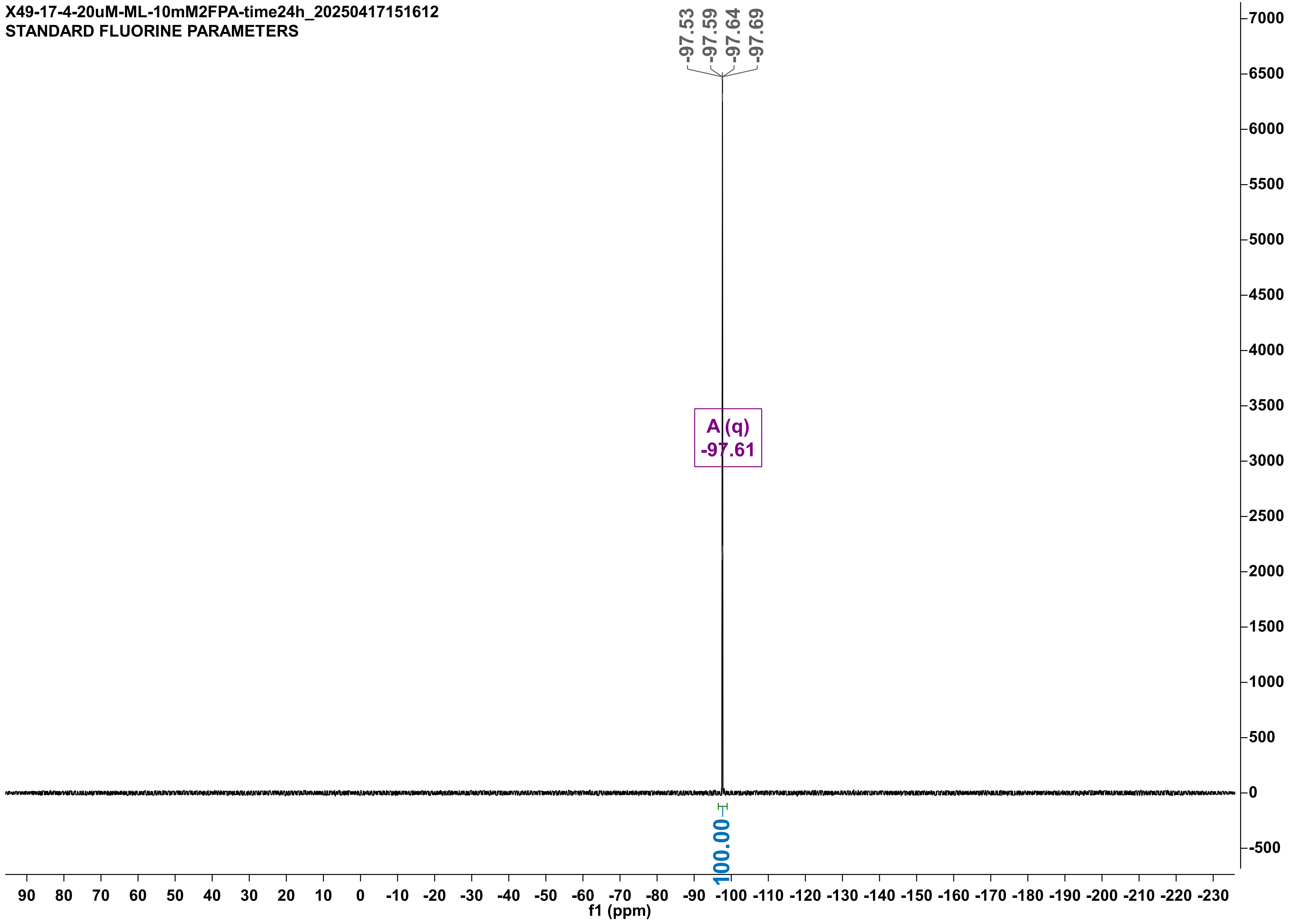

X49-17-4-20uM-ML-10mM2FPA-time24h_20250417151612
STANDARD FLUORINE PARAMETERS
-97.53
-97.59
-97.64
-97.69
A (q)
-97.61
100.00
f1 (ppm)

X49-17-4-20uMPLM-2-10mM2FPA-time24h_20250417151629
STANDARD FLUORINE PARAMETERS

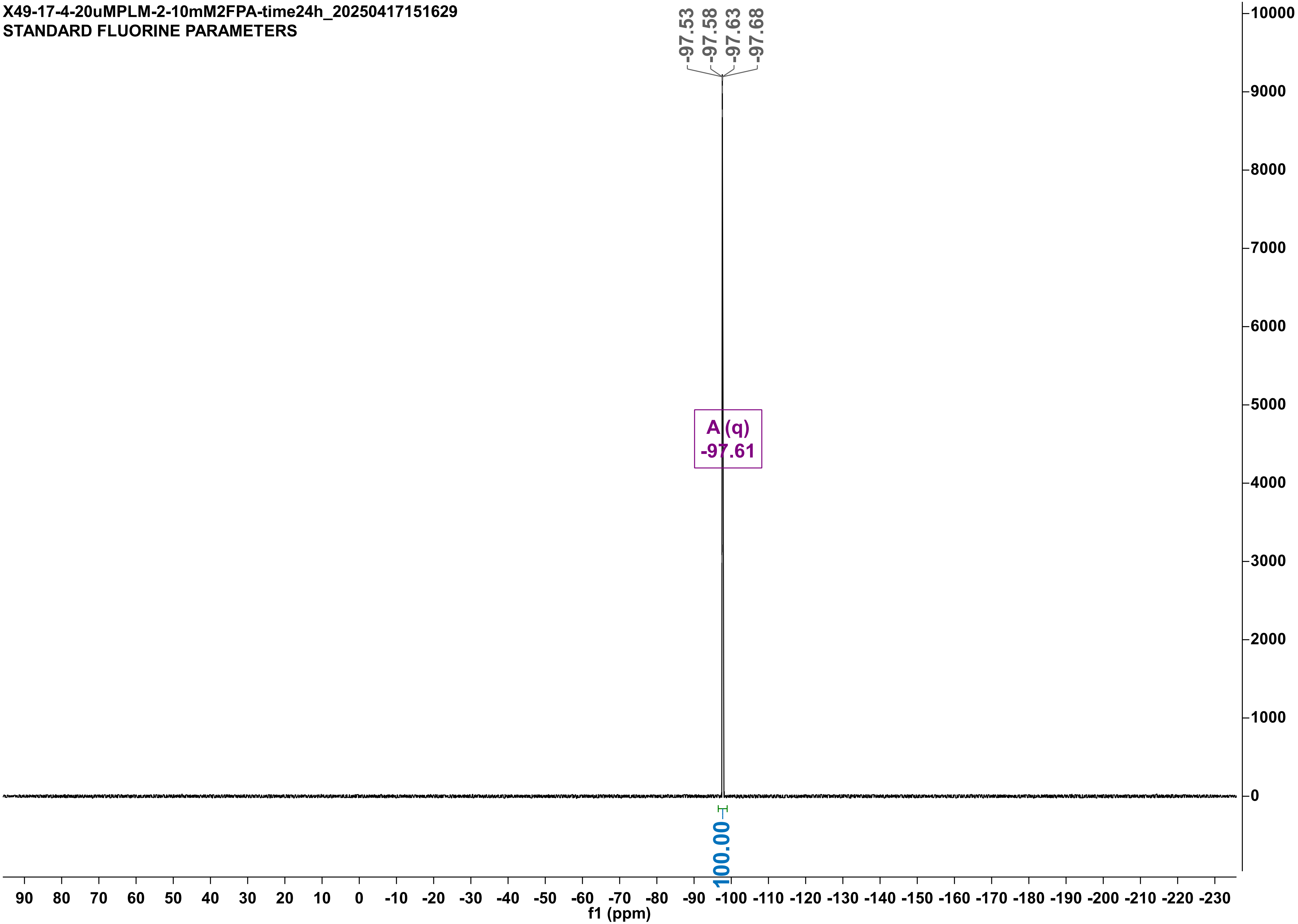

X49-17-4-20uMRHL-10mM2FPA-time24h_20250417161927
STANDARD FLUORINE PARAMETERS

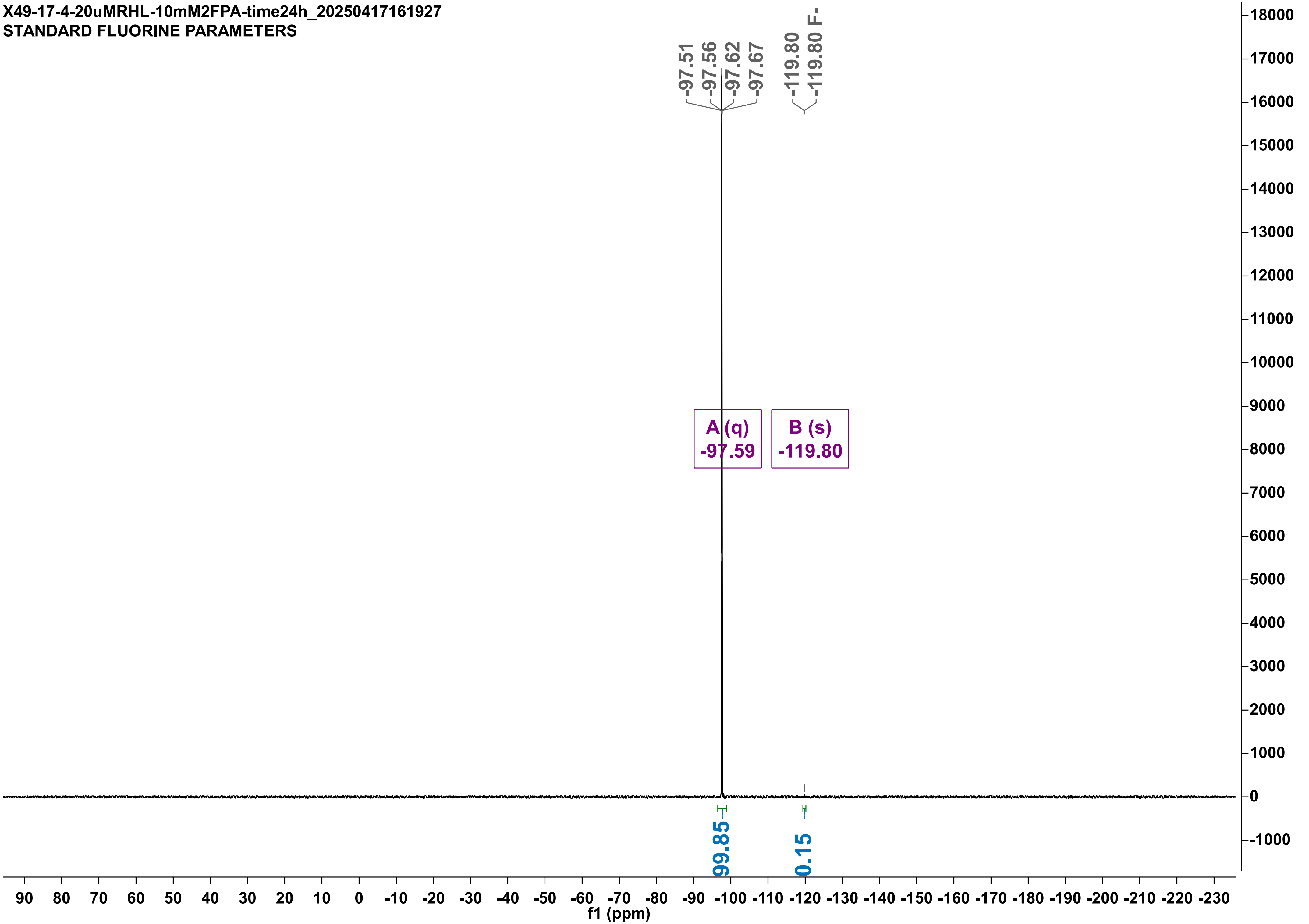

X49-17-4-20uMRLM-10mM2FPA-time24h_20250417161938
STANDARD FLUORINE PARAMETERS

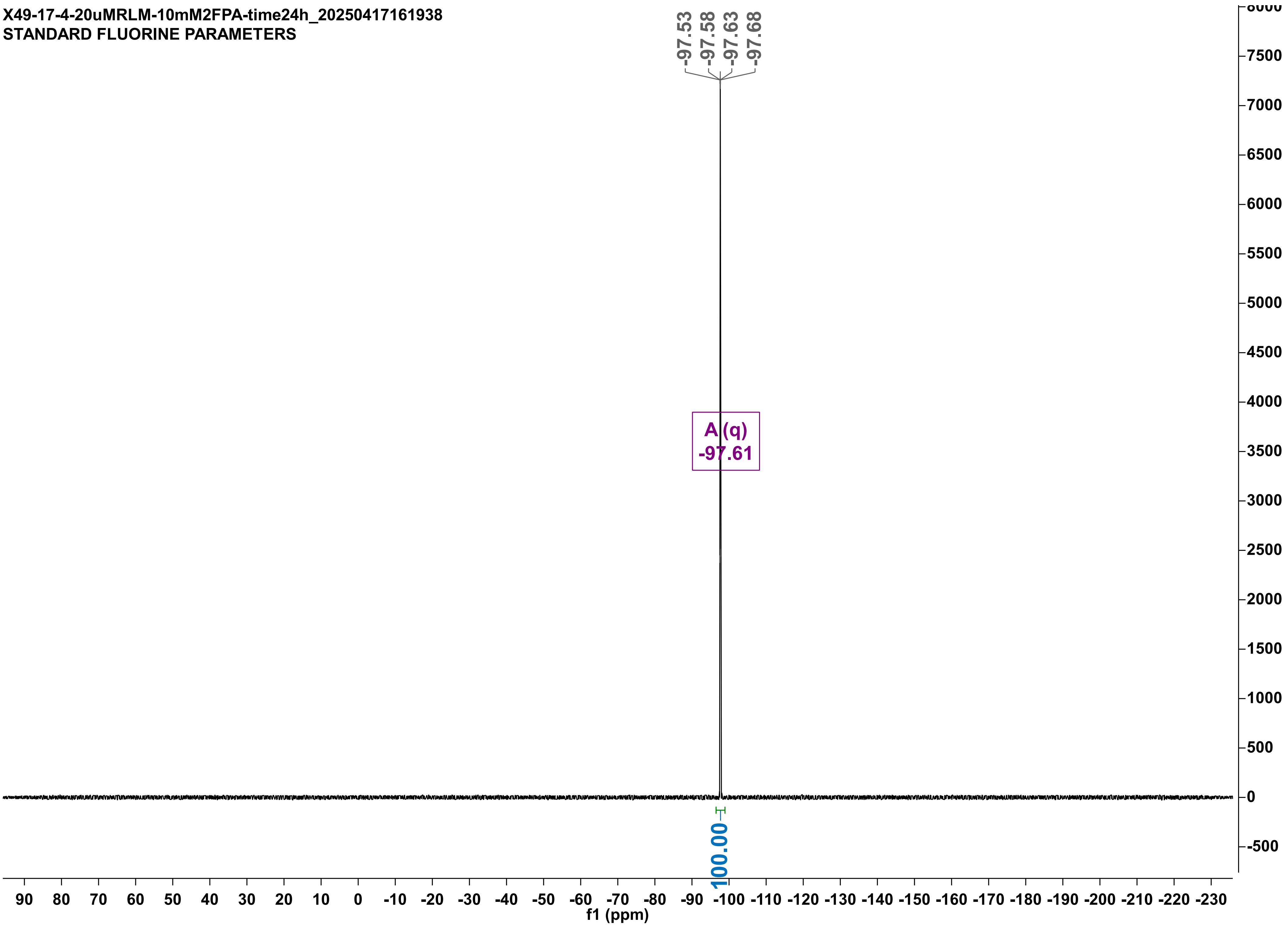

X49-17-4-20uMRPL-10mM2FPA-time24h_20250417161907
STANDARD FLUORINE PARAMETERS

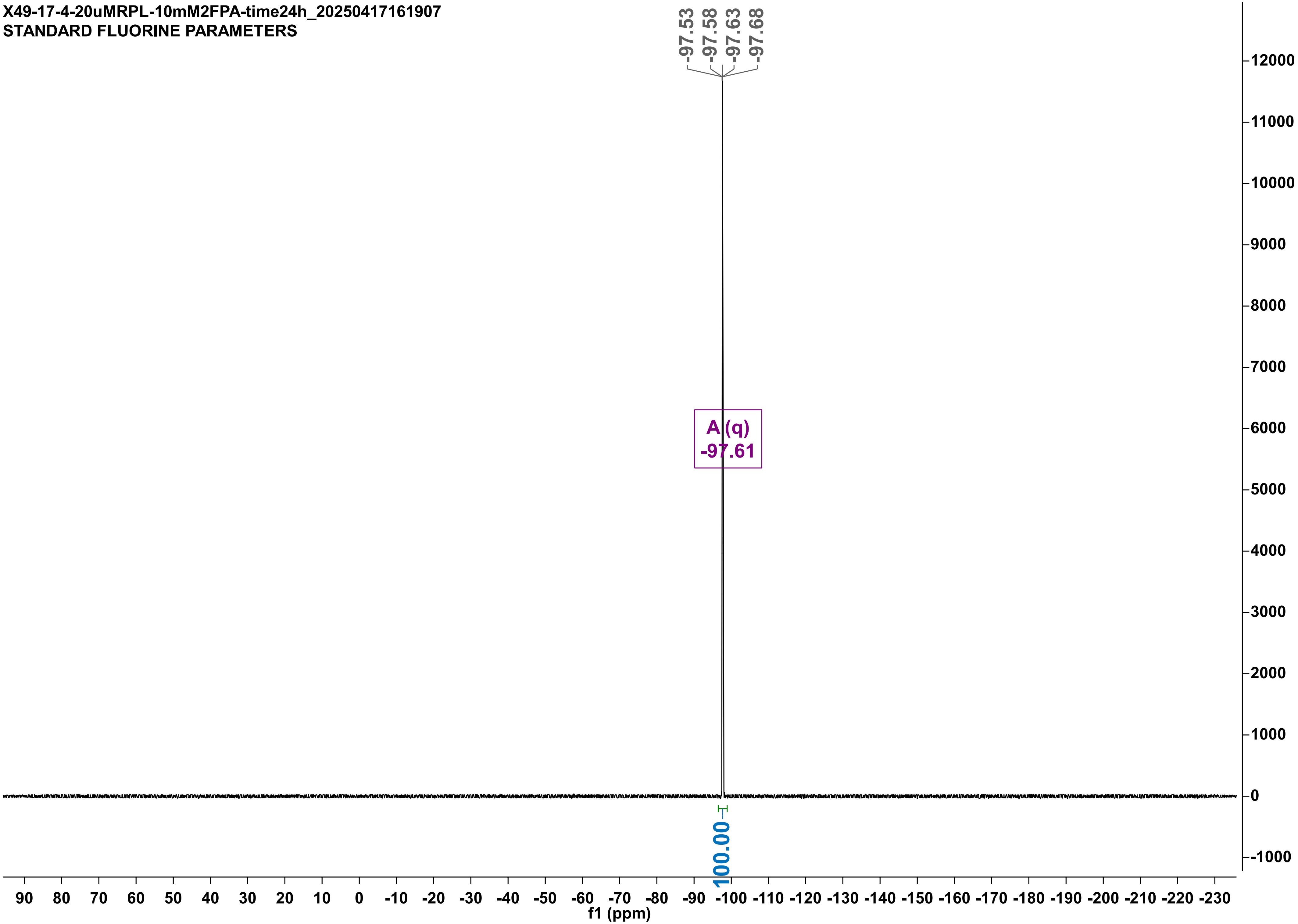

X49-30-5_20uM_-FI_10mM_2FPA_t24h_20250530133233
STANDARD FLUORINE PARAMETERS

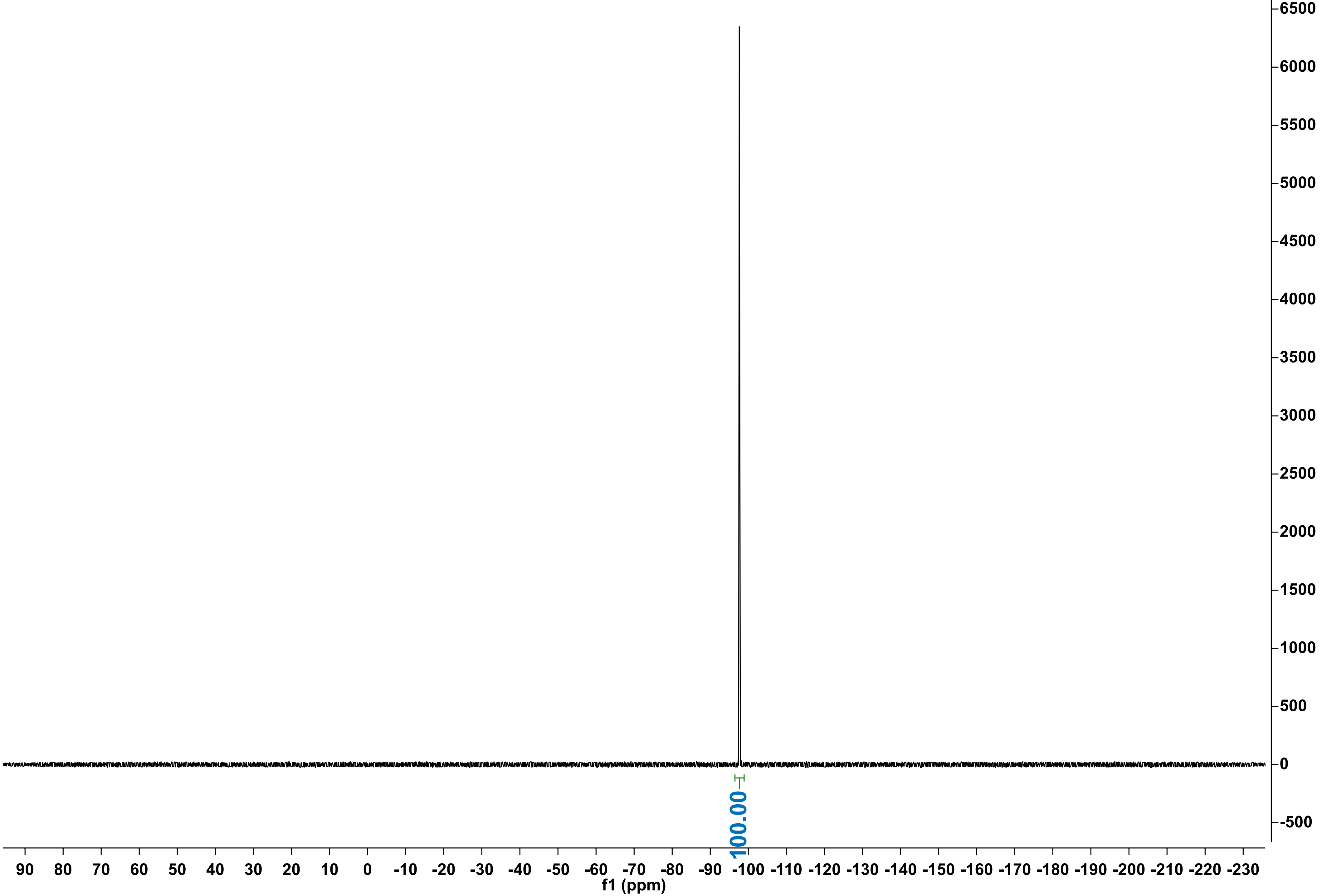

X49-30-5_20uM_LVM_10mM_2FPA_t24h_20250530124414
STANDARD FLUORINE PARAMETERS

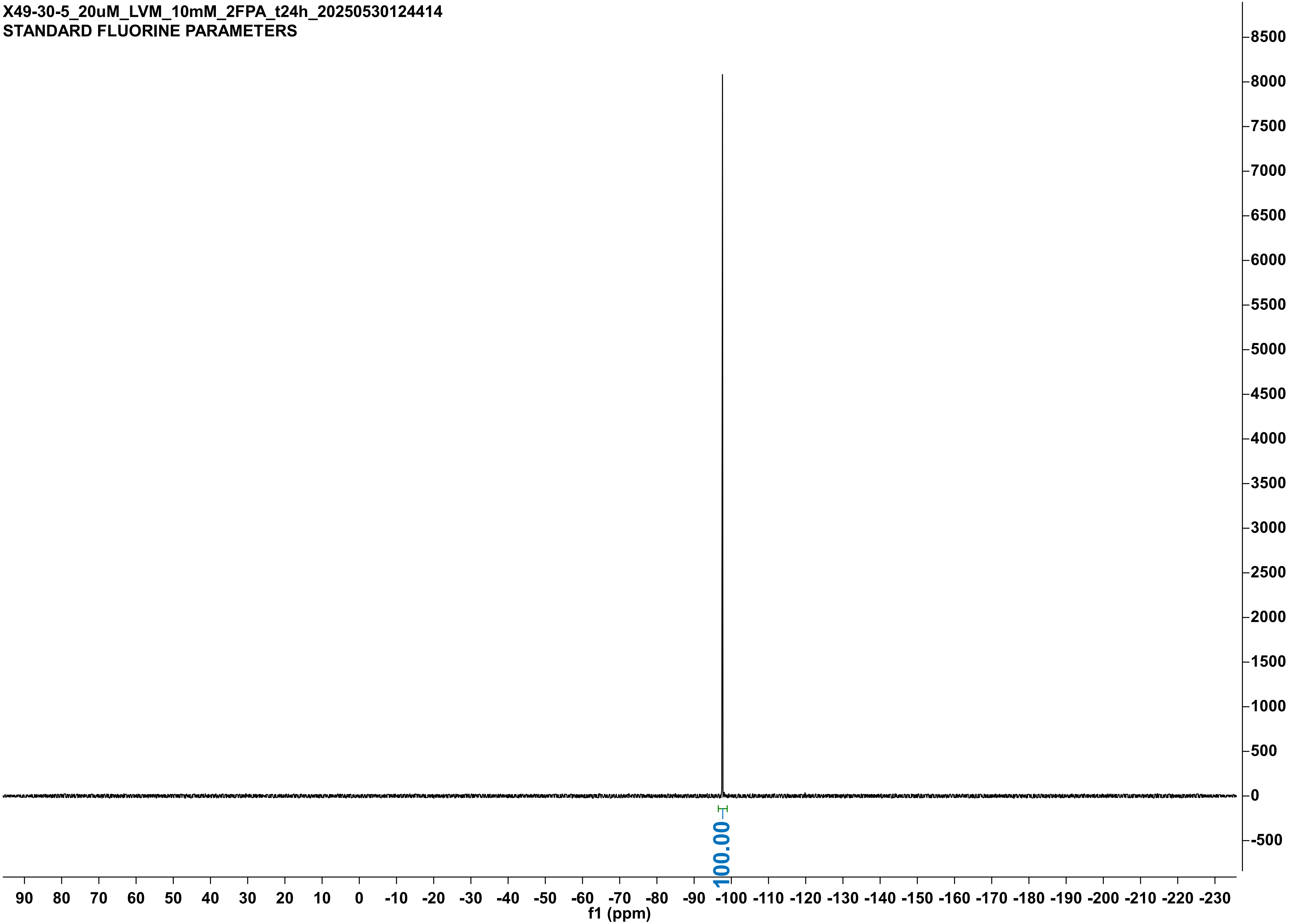

X49-30-5_20uM_Q245A_10mM_2FPA_t24h_20250530133729
STANDARD FLUORINE PARAMETERS

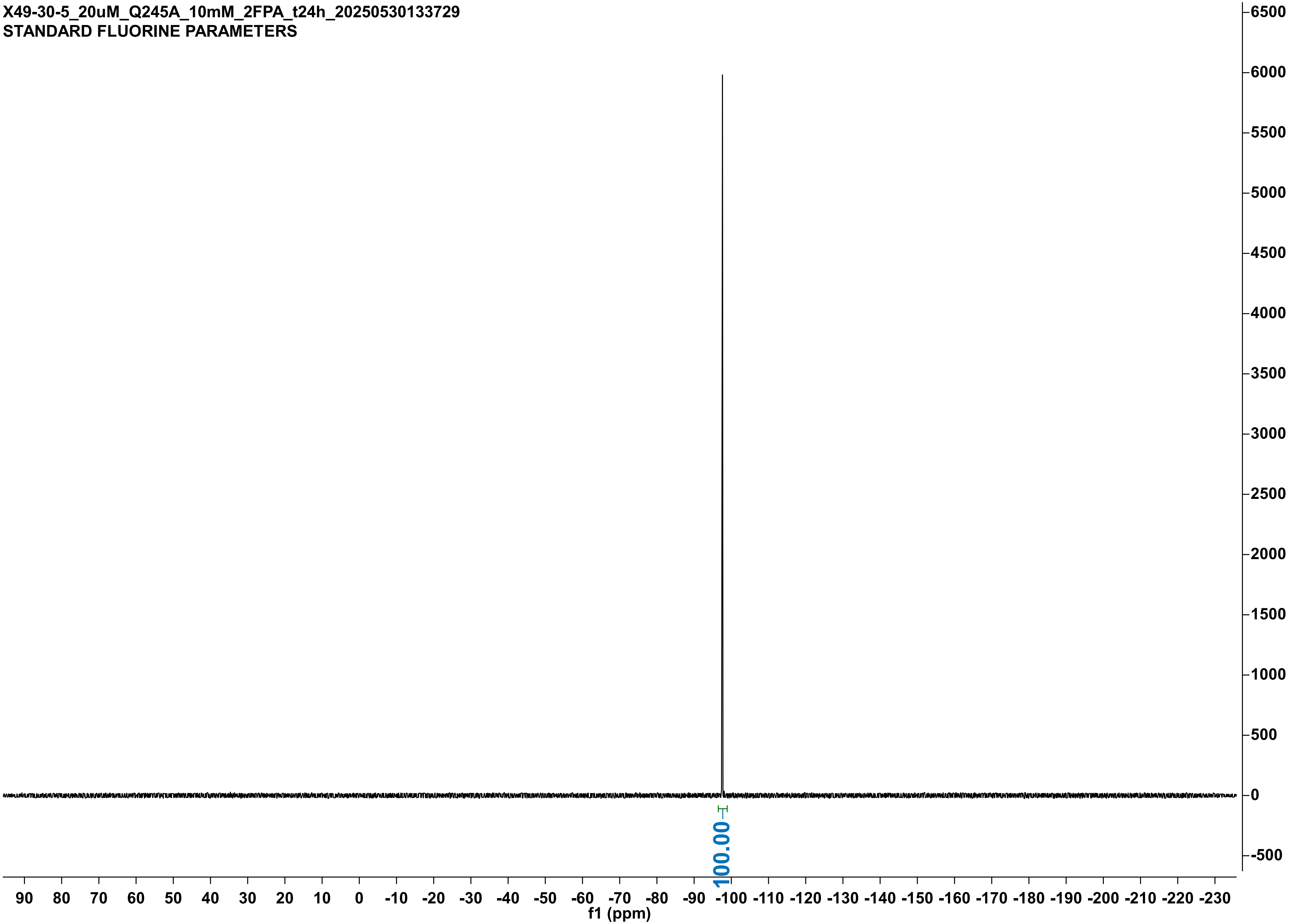

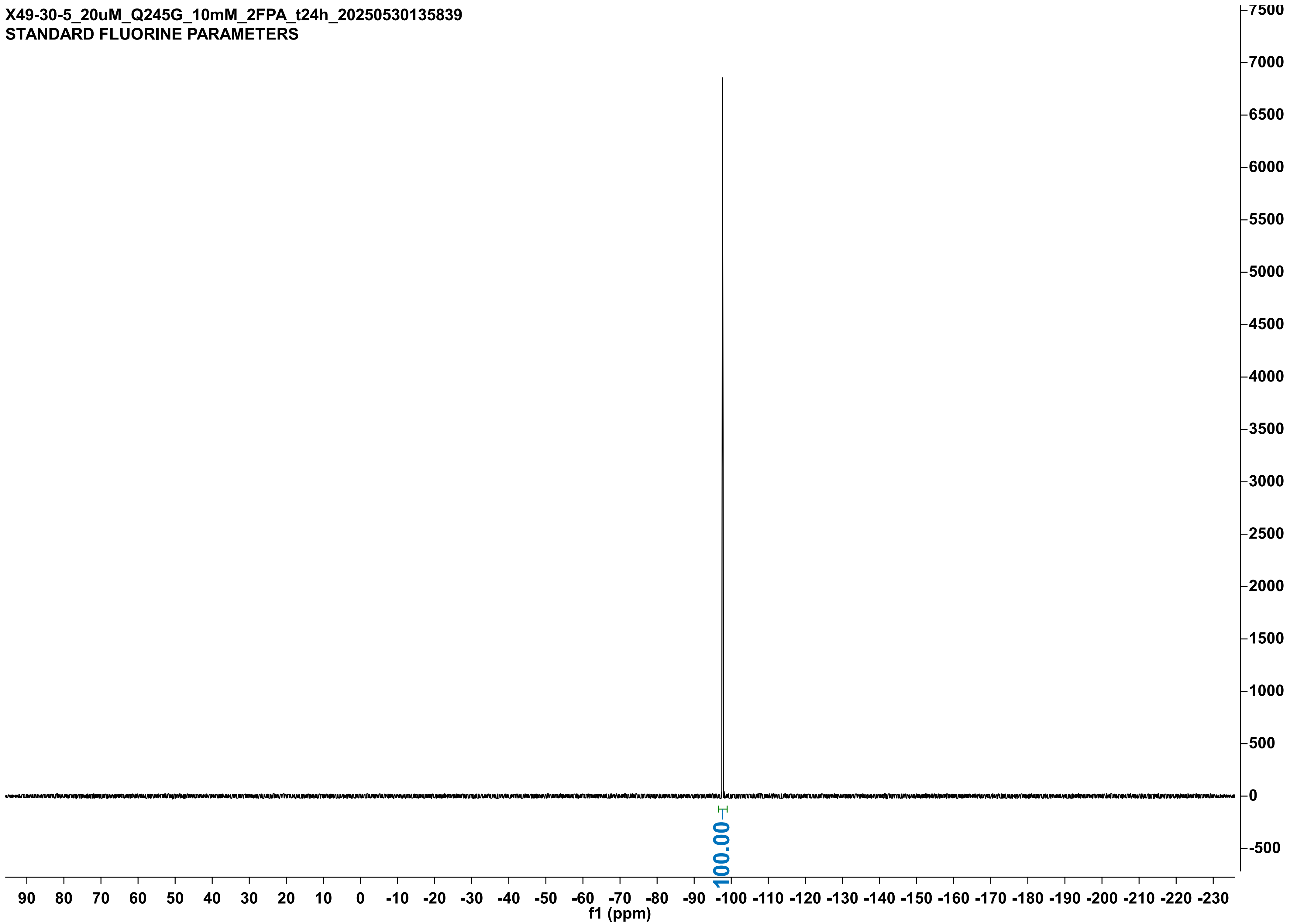

X49-30-5_20uM_Q245G_10mM_2FPA_t24h_20250530135839
STANDARD FLUORINE PARAMETERS
100.00
f1 (ppm)

X49-30-5_20uM_Q245R_10mM_2FPA_t24h_20250530140345
STANDARD FLUORINE PARAMETERS

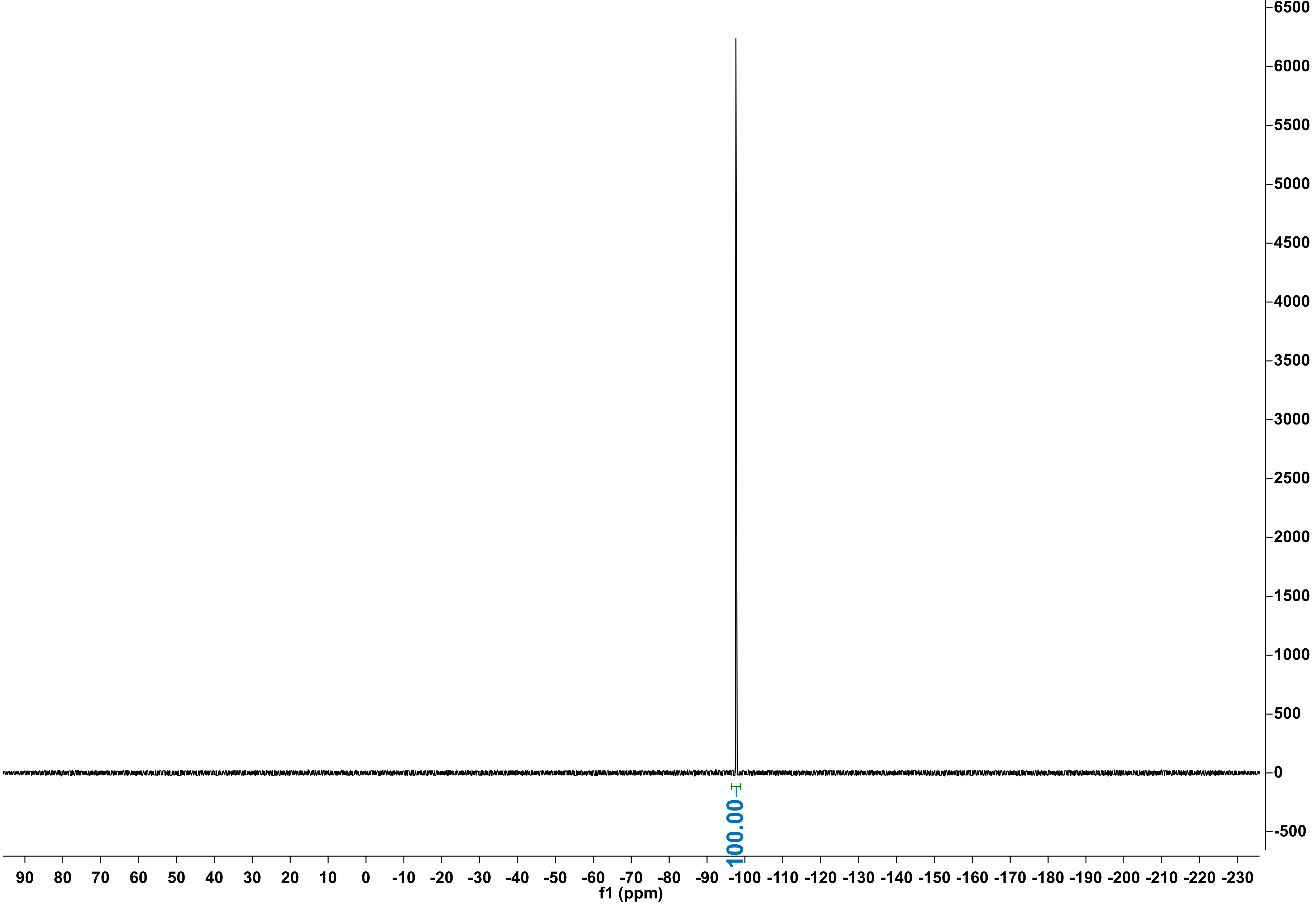

X49-30-5_20uM_-VM_10mM_2FPA_t24h_20250530132959
STANDARD FLUORINE PARAMETERS

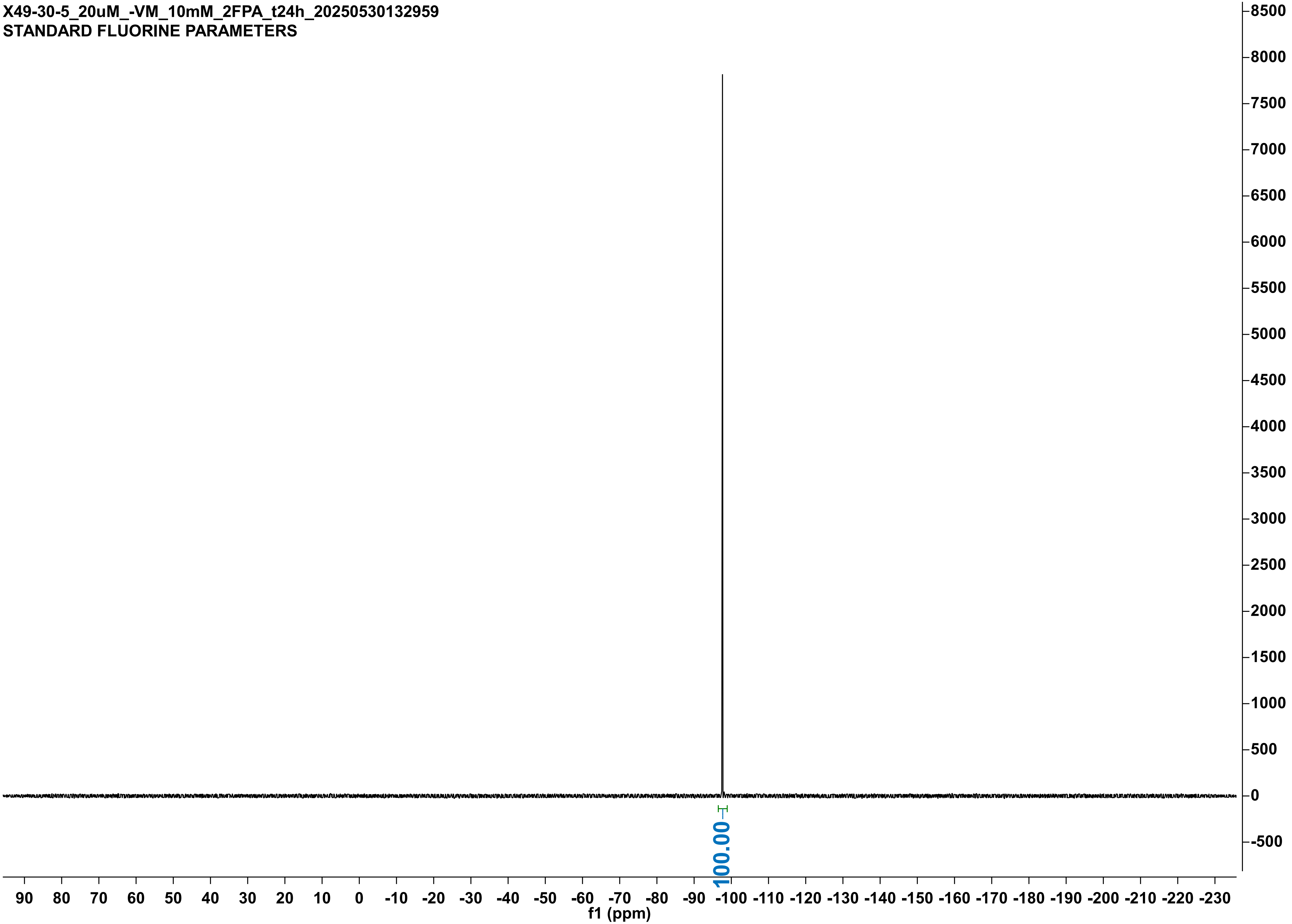

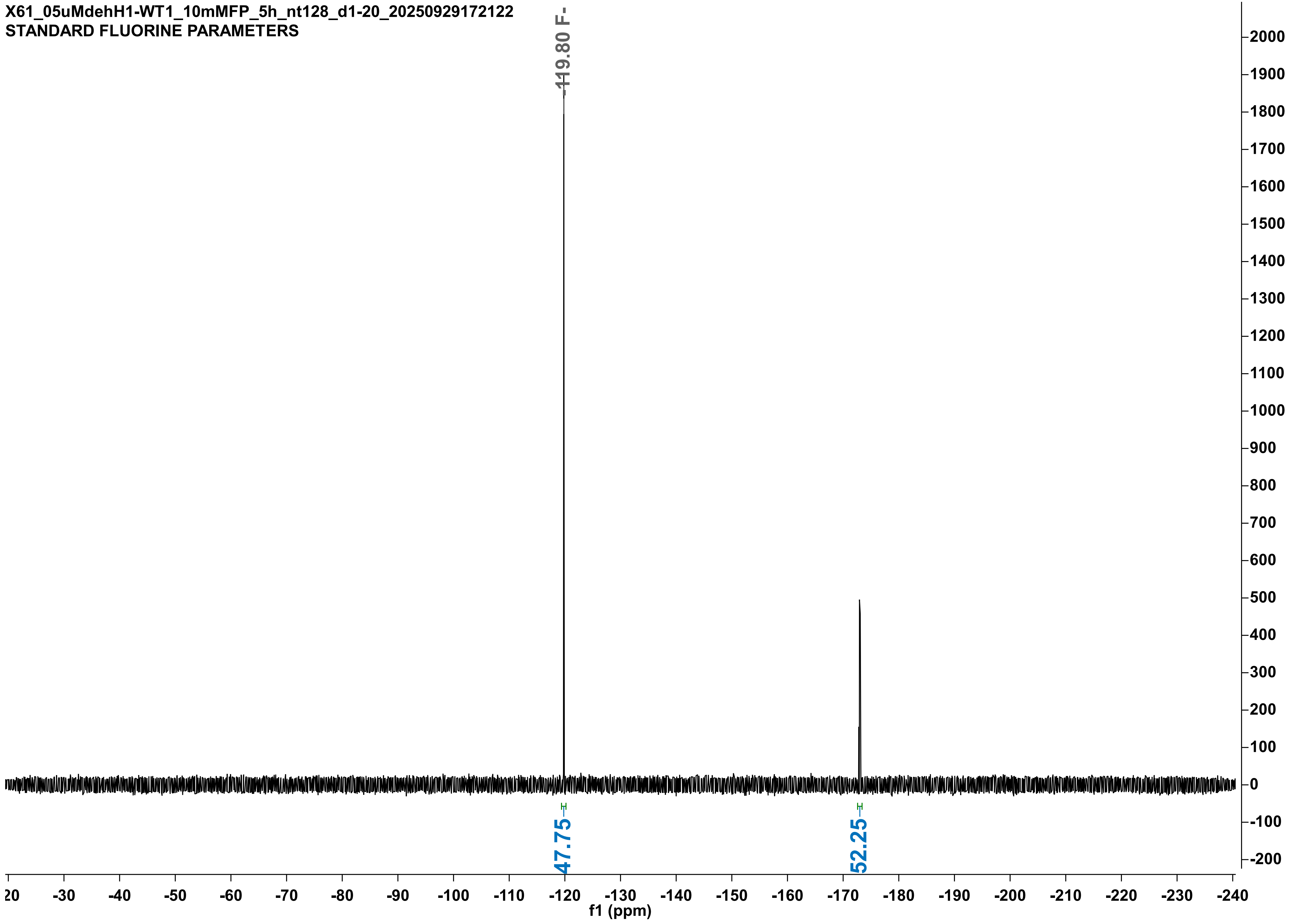

X61_05uMdehH1-WT1_10mMFP_5h_nt128_d1-20_20250929172122
STANDARD FLUORINE PARAMETERS
-119.80 F-
47.75
52.25
f1 (ppm)

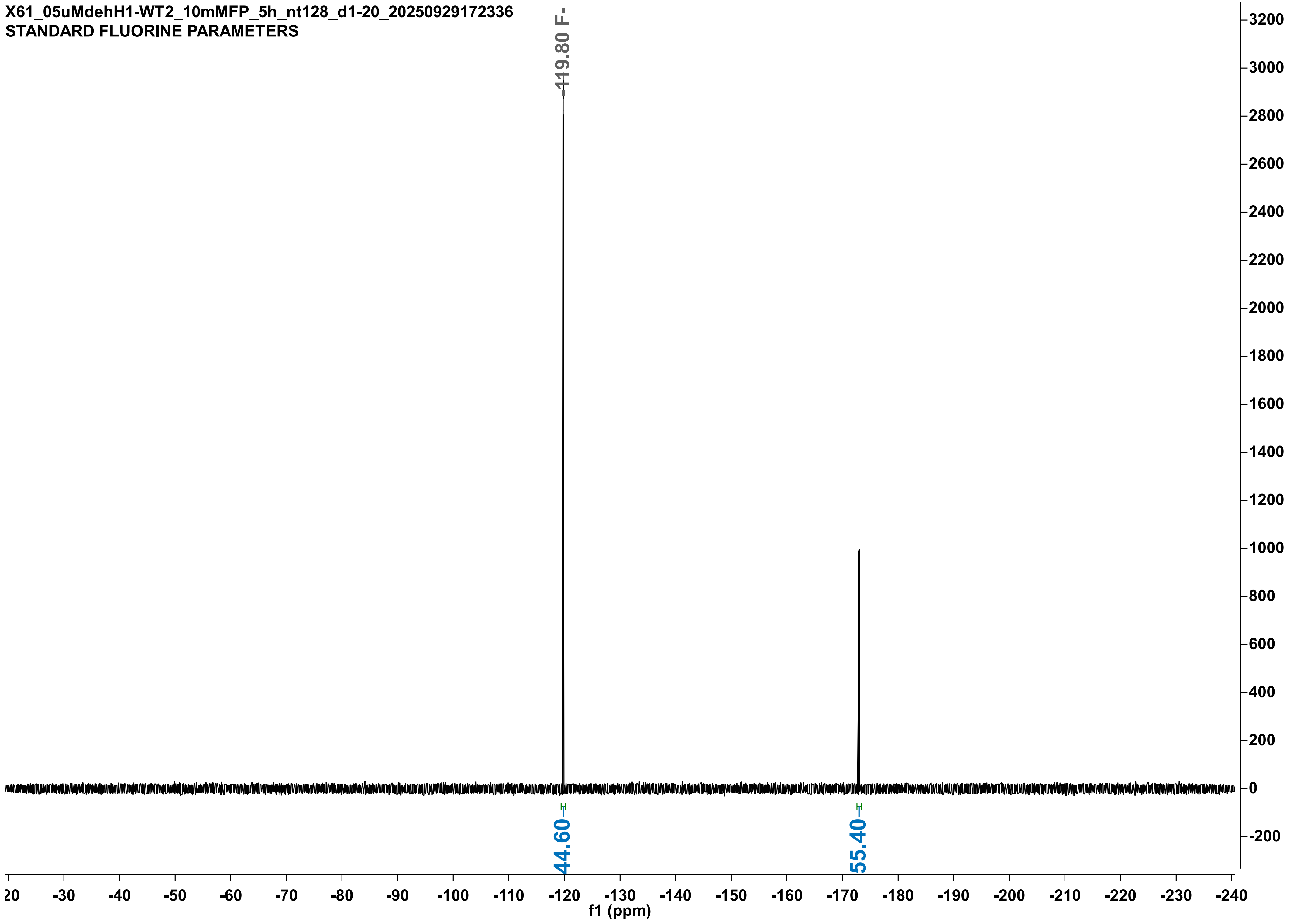

X61_05uMdehH1-WT2_10mMFP_5h_nt128_d1-20_20250929172336
STANDARD FLUORINE PARAMETERS
-119.80 F-
44.60
55.40
f1 (ppm)

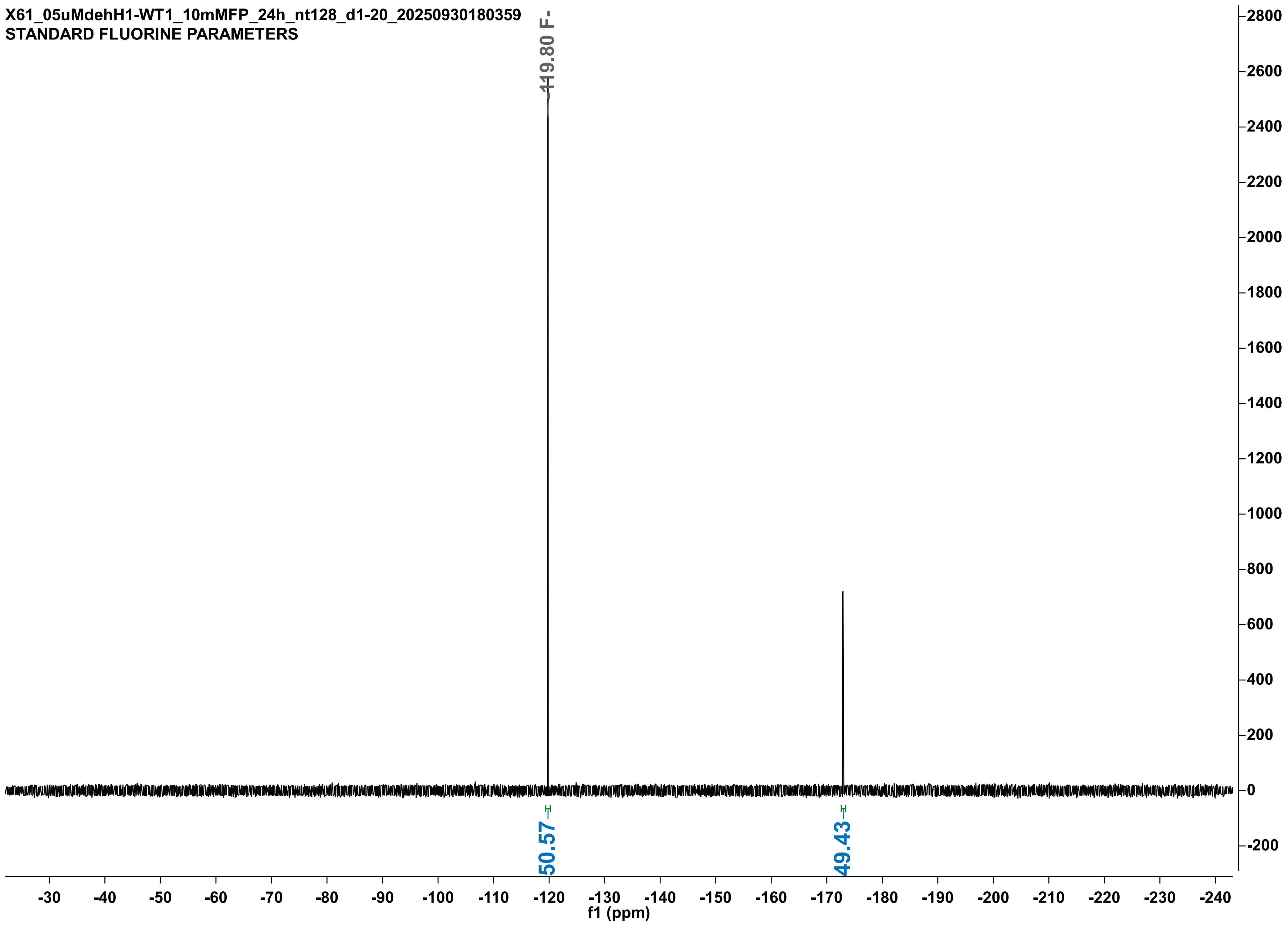

X61_05uMdehH1-WT1_10mMFP_24h_nt128_d1-20_20250930180359
STANDARD FLUORINE PARAMETERS
-119.80 F-
50.57
49.43
f1 (ppm)

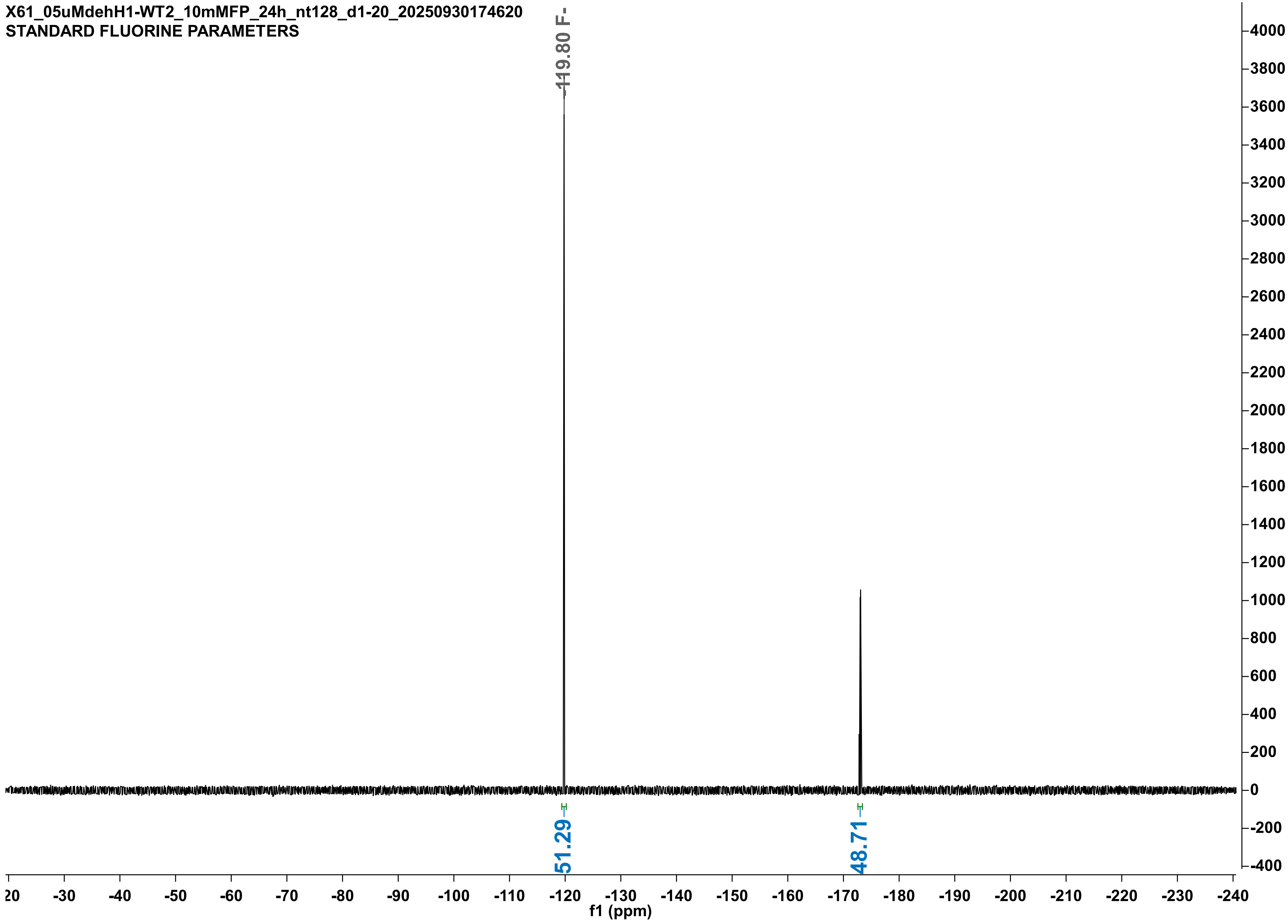
X61_05uMdehH1-WT2_10mMFP_24h_nt128_d1-20_20250930174620
STANDARD FLUORINE PARAMETERS
-119.80 F-
51.29
48.71
f1 (ppm)

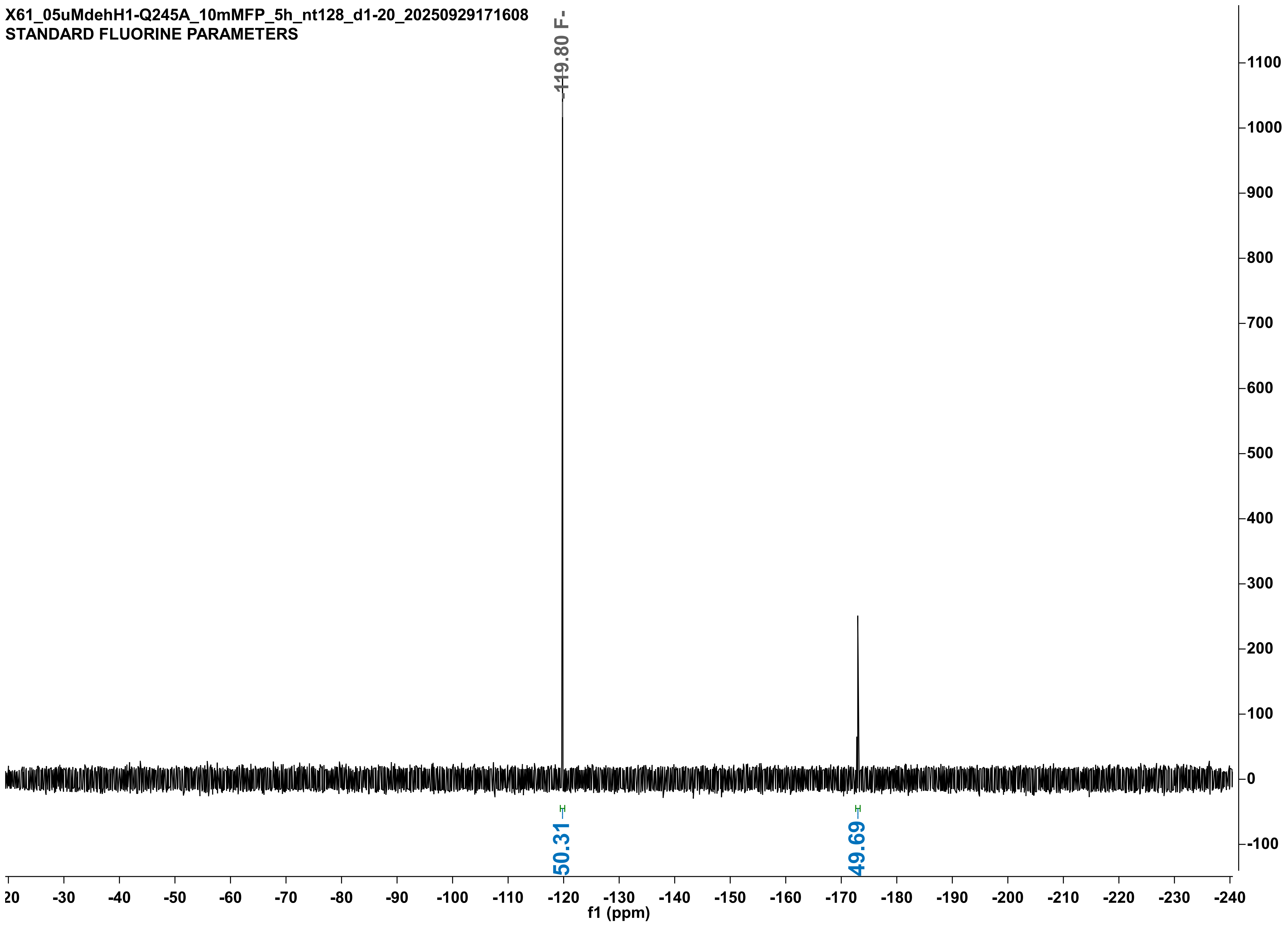

X61_05uMdehH1-Q245A_10mMFP_5h_nt128_d1-20_20250929171608
STANDARD FLUORINE PARAMETERS
-119.80 F-
50.31
49.69
20
-30
-40
-50
-60
-70
-80
-90
-100
-110
-120
-130
-140
-150
-160
-170
-180
-190
-200
-210
-220
-230
-240
f1 (ppm)
1100
1000
900
800
700
600
500
400
300
200
100
0
-100

X61_05uMdehH1-Q245A_10mMFP_24h_nt128_d1-20_20250930174653
STANDARD FLUORINE PARAMETERS

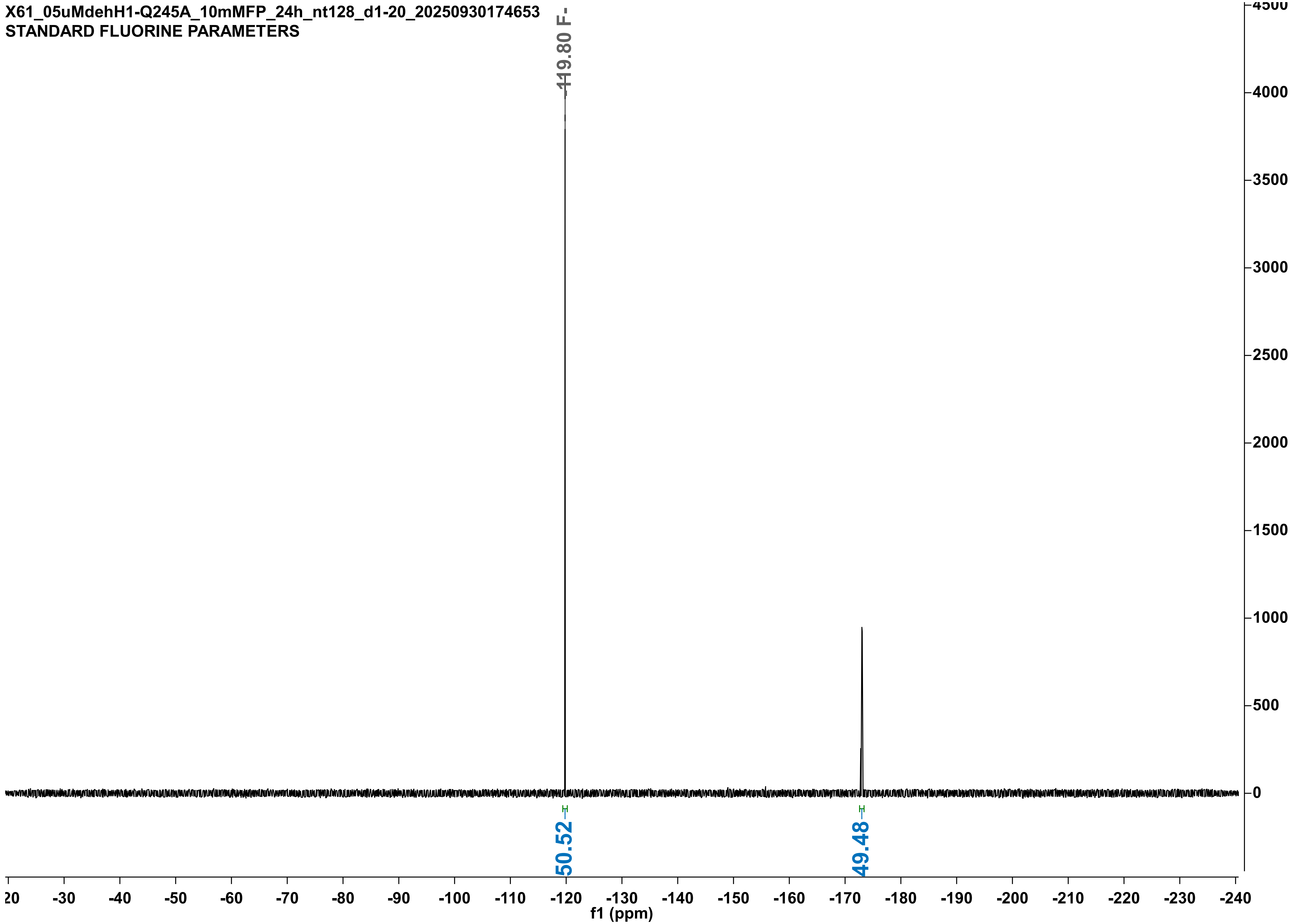

X61_05uMdehH1-Q245A-DUP_10mMFP_5h_nt128_d1-20_20250929171424
STANDARD FLUORINE PARAMETERS

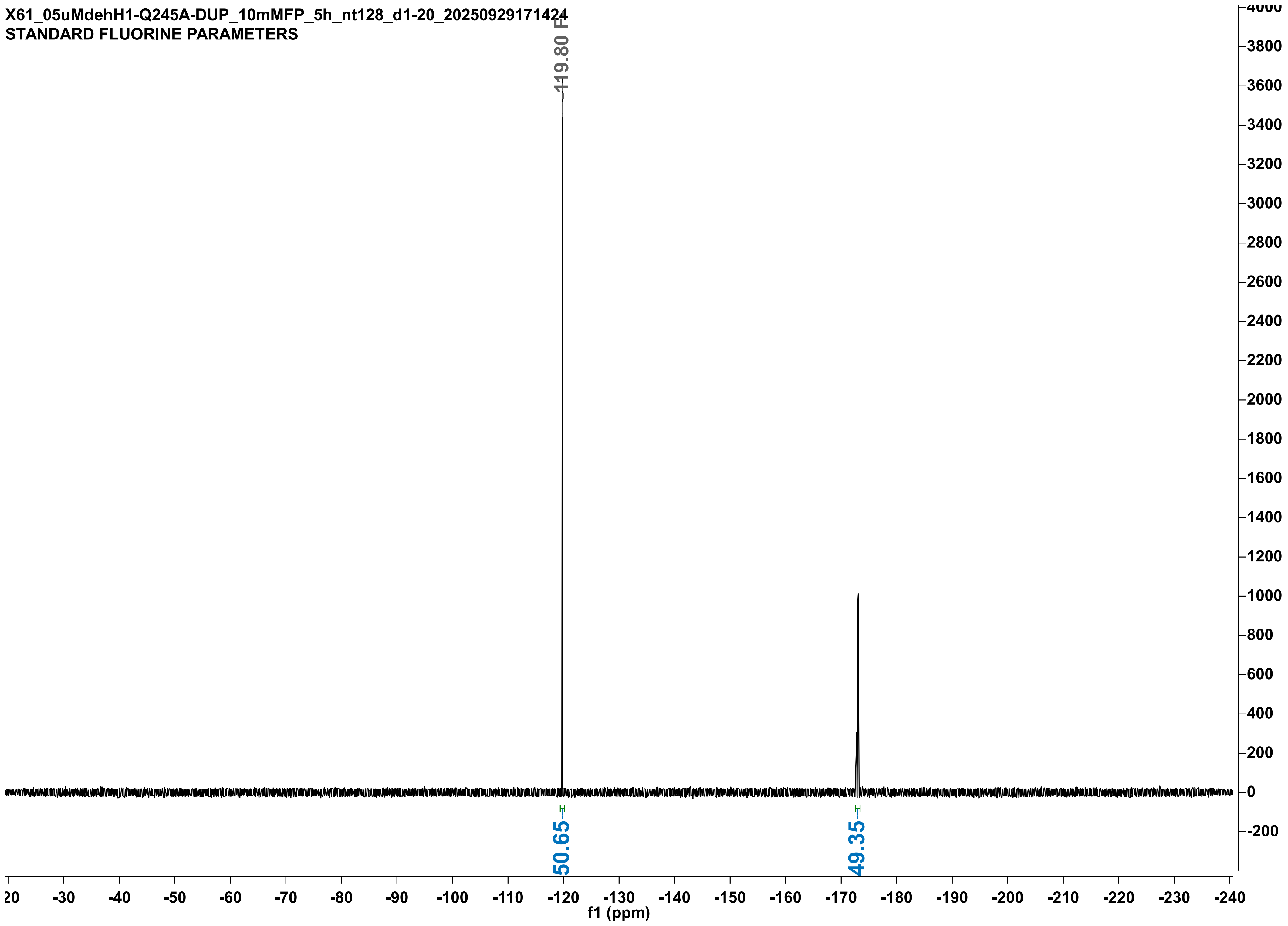

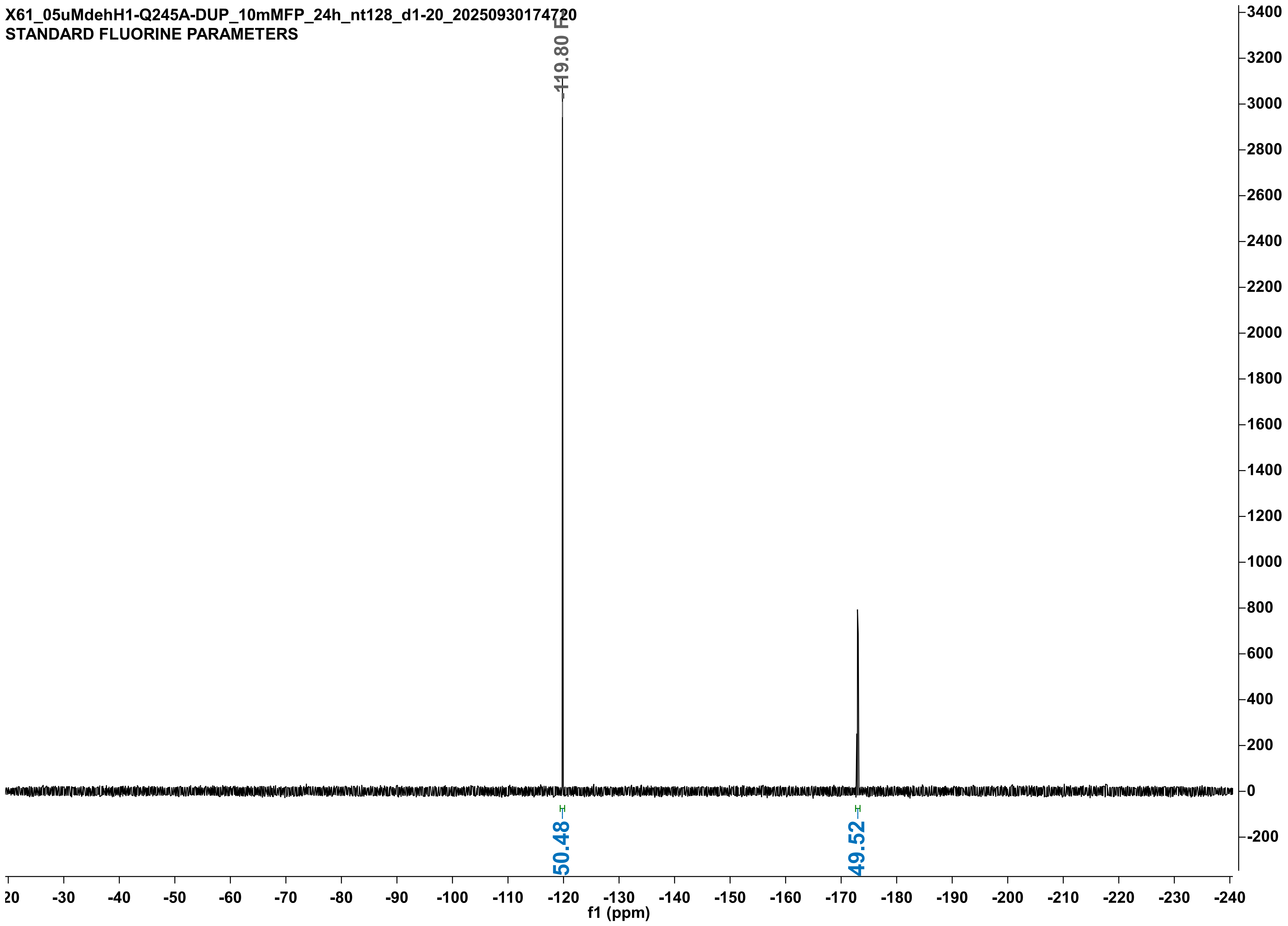

X61_05uMdehH1-Q245A-DUP_10mMFP_24h_nt128_d1-20_20250930174720
STANDARD FLUORINE PARAMETERS
-119.80 F
50.48
49.52
f1 (ppm)

X61_05uMdehH1-WT1_10mMFP_5h_20250929172104
STANDARD FLUORINE PARAMETERS

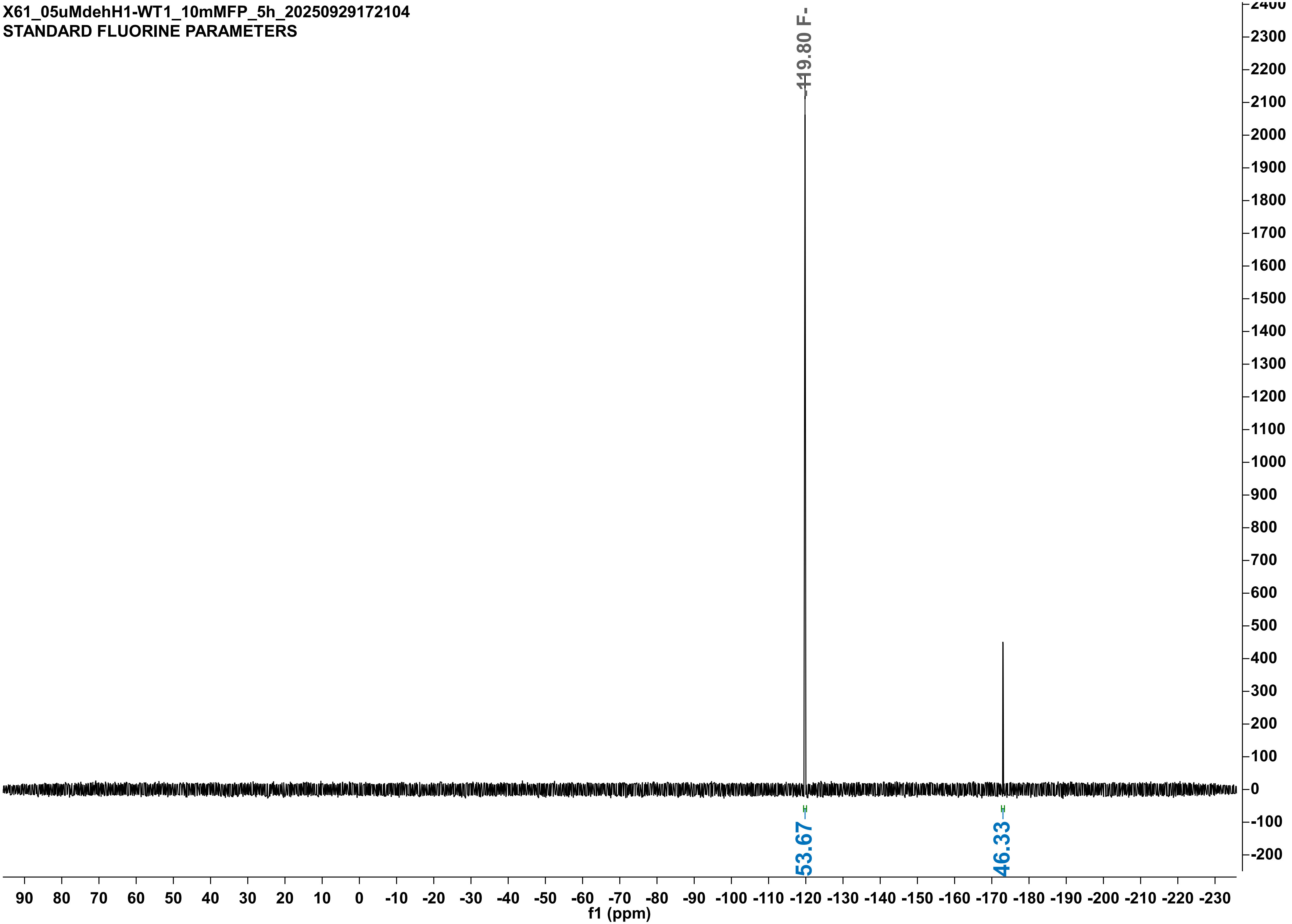

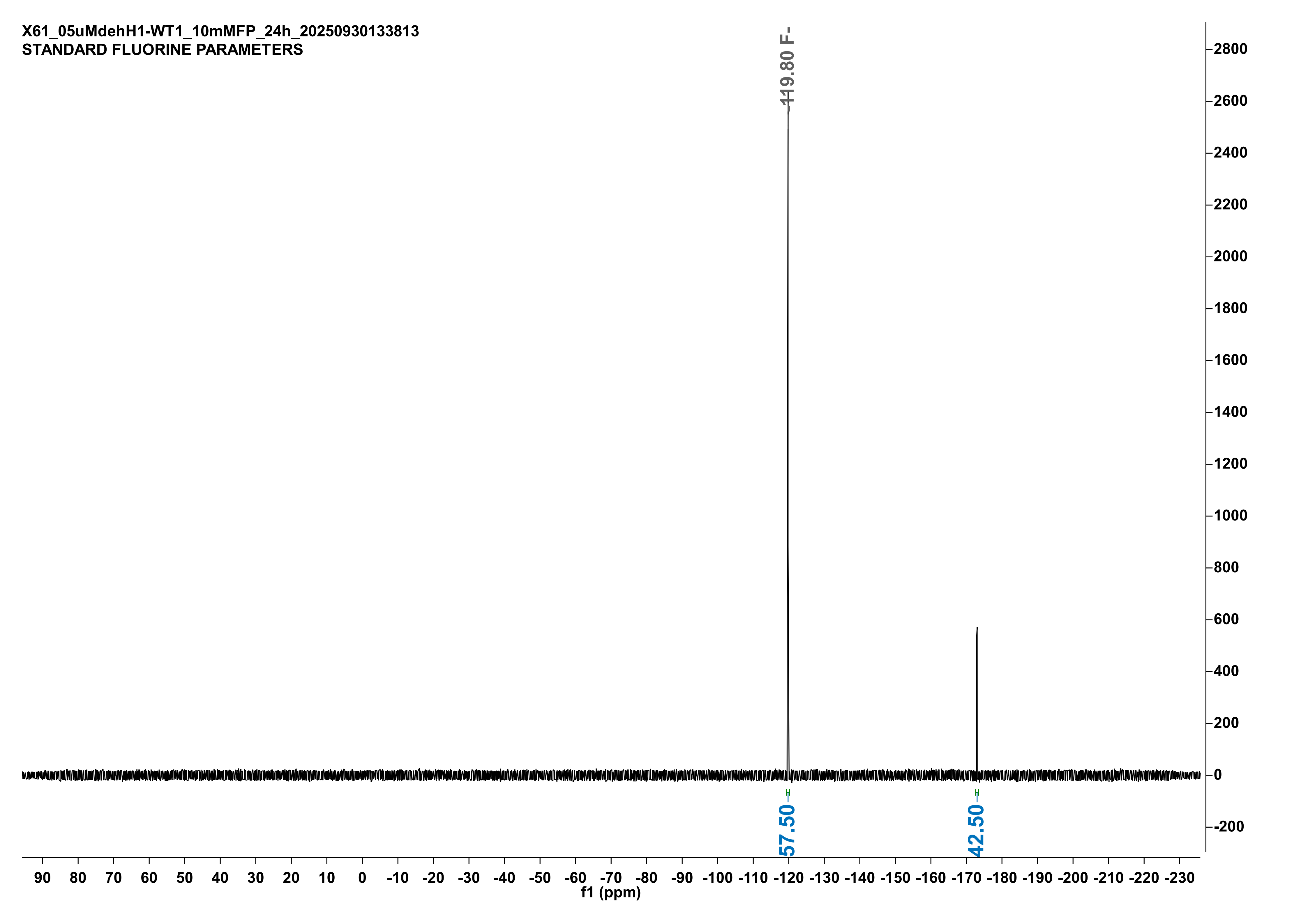
X61_05uMdehH1-WT1_10mMFP_24h_20250930133813
STANDARD FLUORINE PARAMETERS
-119.80 F-
57.50
42.50
f1 (ppm)

X61_05uMdehH1-Q245A_10mMFP_5h_20250929171504
STANDARD FLUORINE PARAMETERS

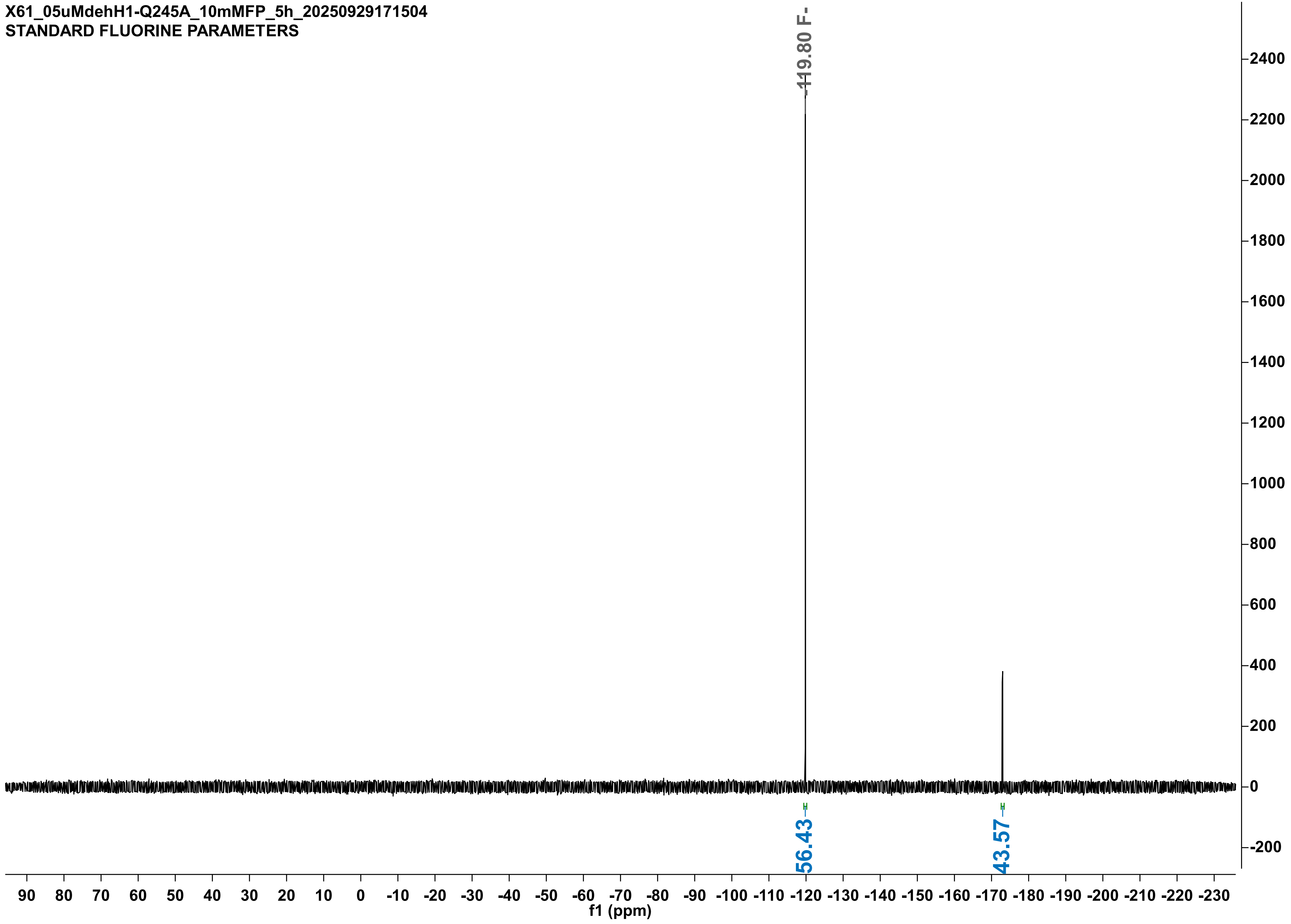

X61_05uMdehH1-Q245A_10mMFP_24h_20250930174641
STANDARD FLUORINE PARAMETERS

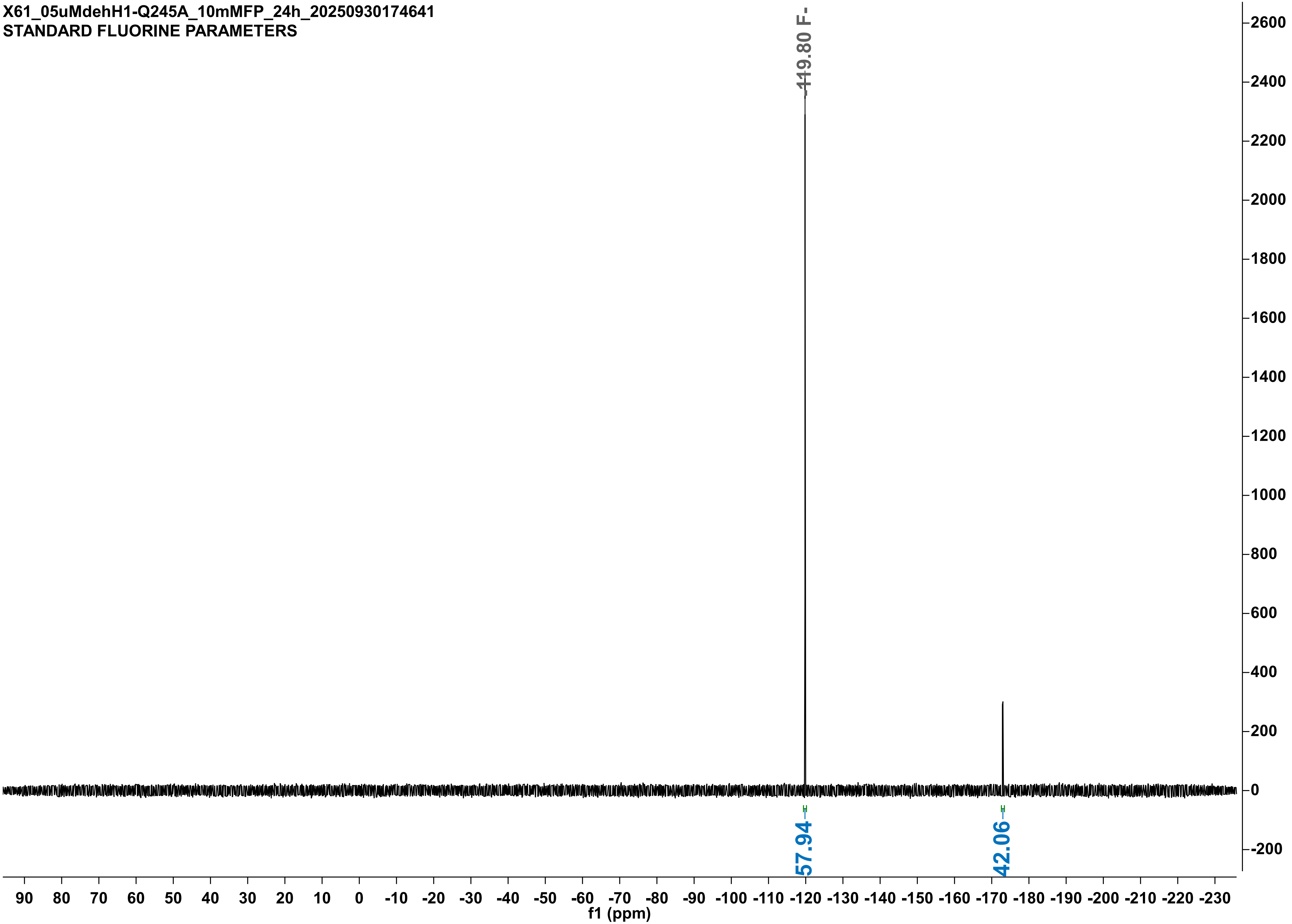

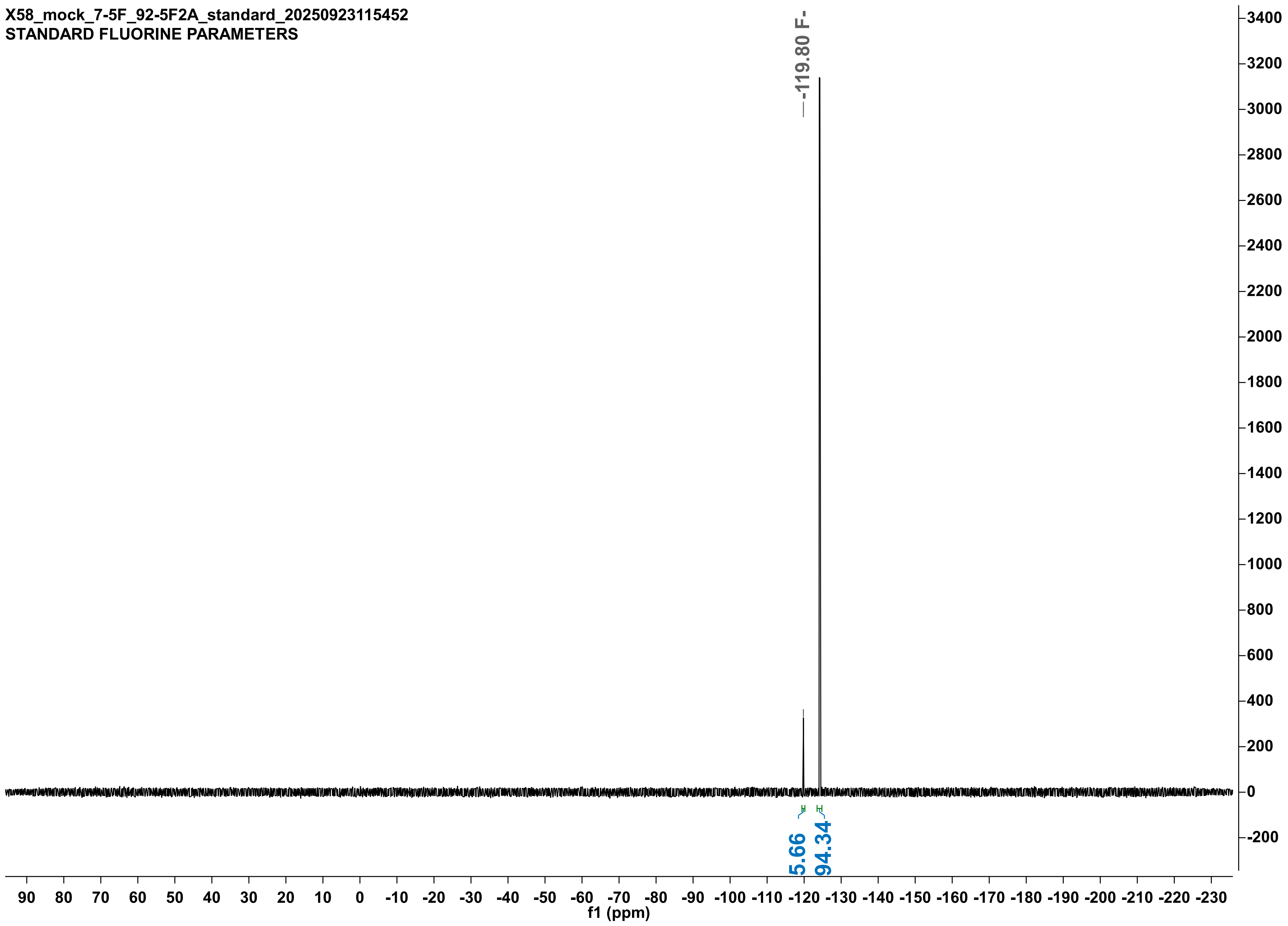
X58_mock_7-5F_92-5F2A_standard_20250923115452
STANDARD FLUORINE PARAMETERS
-119.80 F-
5.66
94.34
f1 (ppm)

X58_mock_7-5F_92-5F2A_pw90_20250923120646
STANDARD FLUORINE PARAMETERS

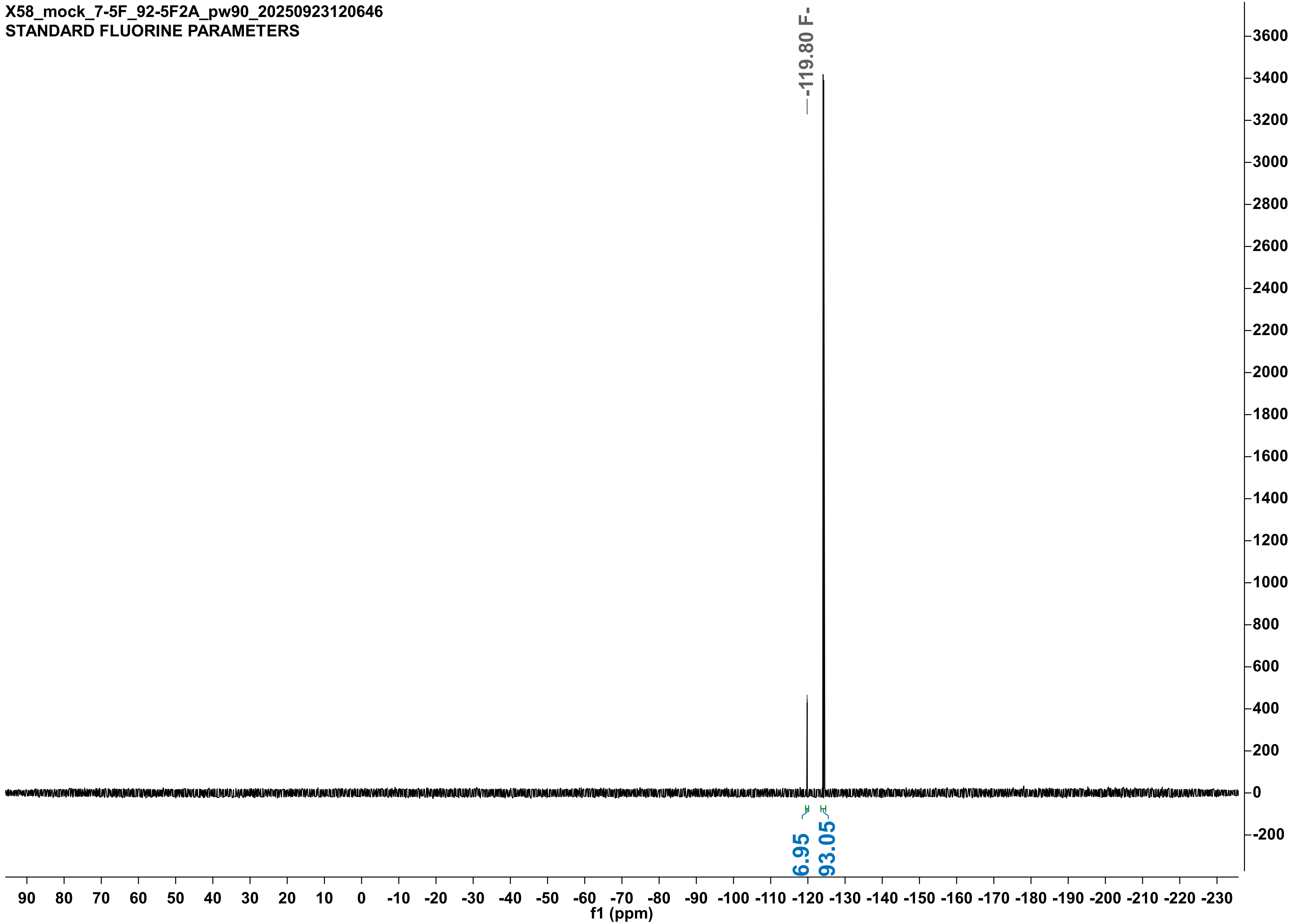

X58_mock_7-5F_92-5F2A_d1-5_20250923120716
STANDARD FLUORINE PARAMETERS

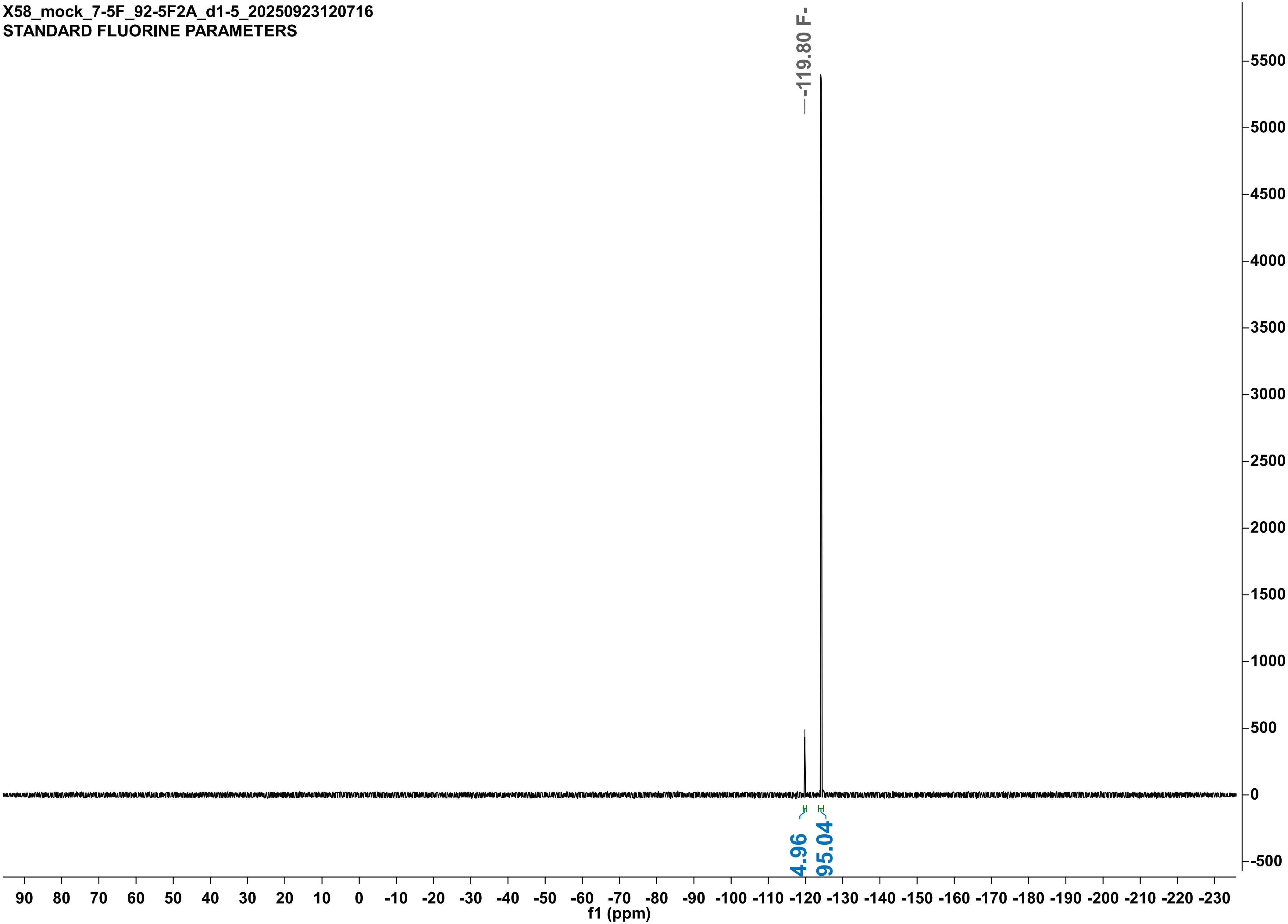

X58_mock_7-5F_92-5F2A_d1-10_20250923120800
STANDARD FLUORINE PARAMETERS

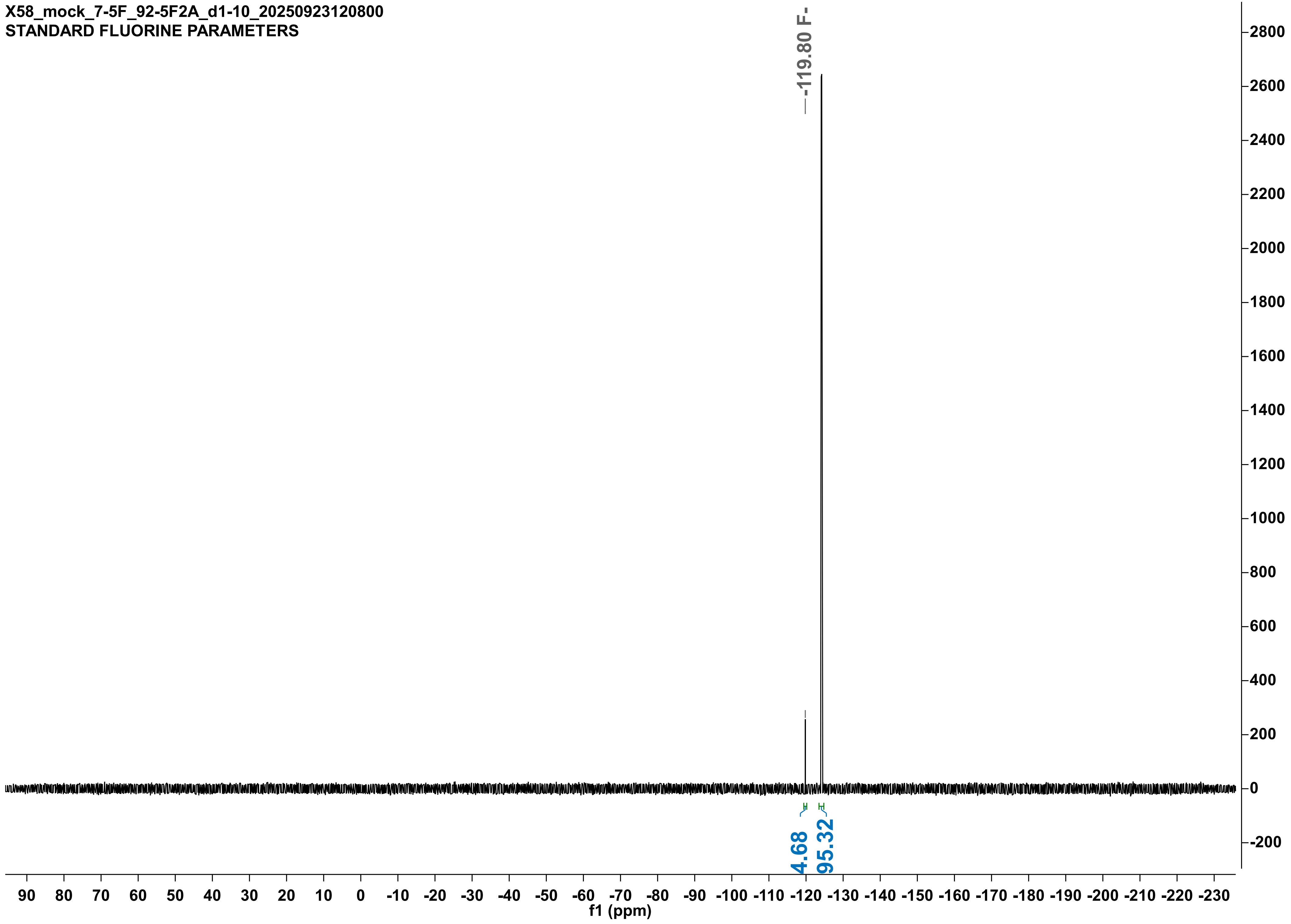

X58_mock_7-5F_92-5F2A_d1-15_20250923120817
STANDARD FLUORINE PARAMETERS

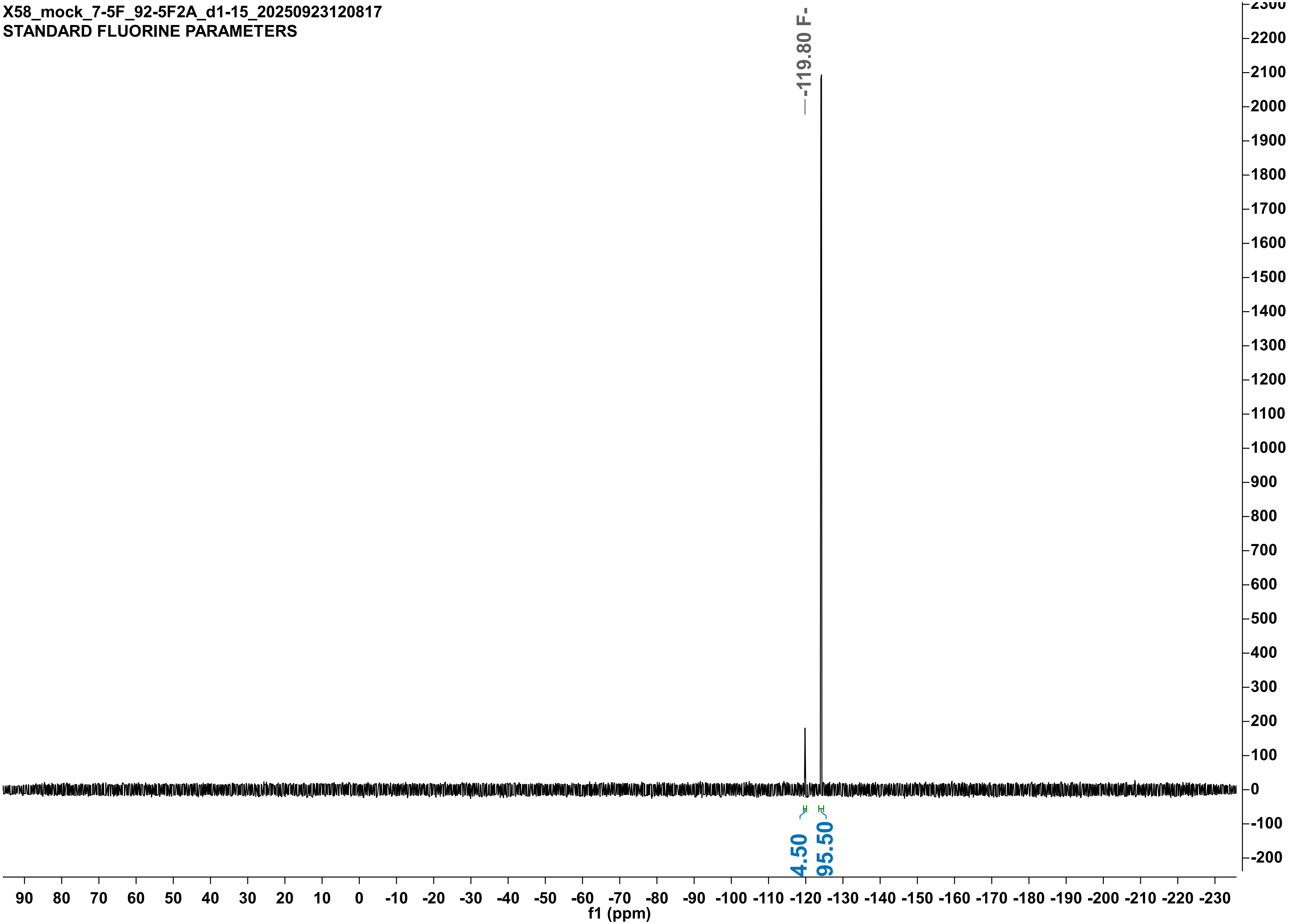

X58_mock_7-5F_92-5F2A_d1-20_20250923120831
STANDARD FLUORINE PARAMETERS

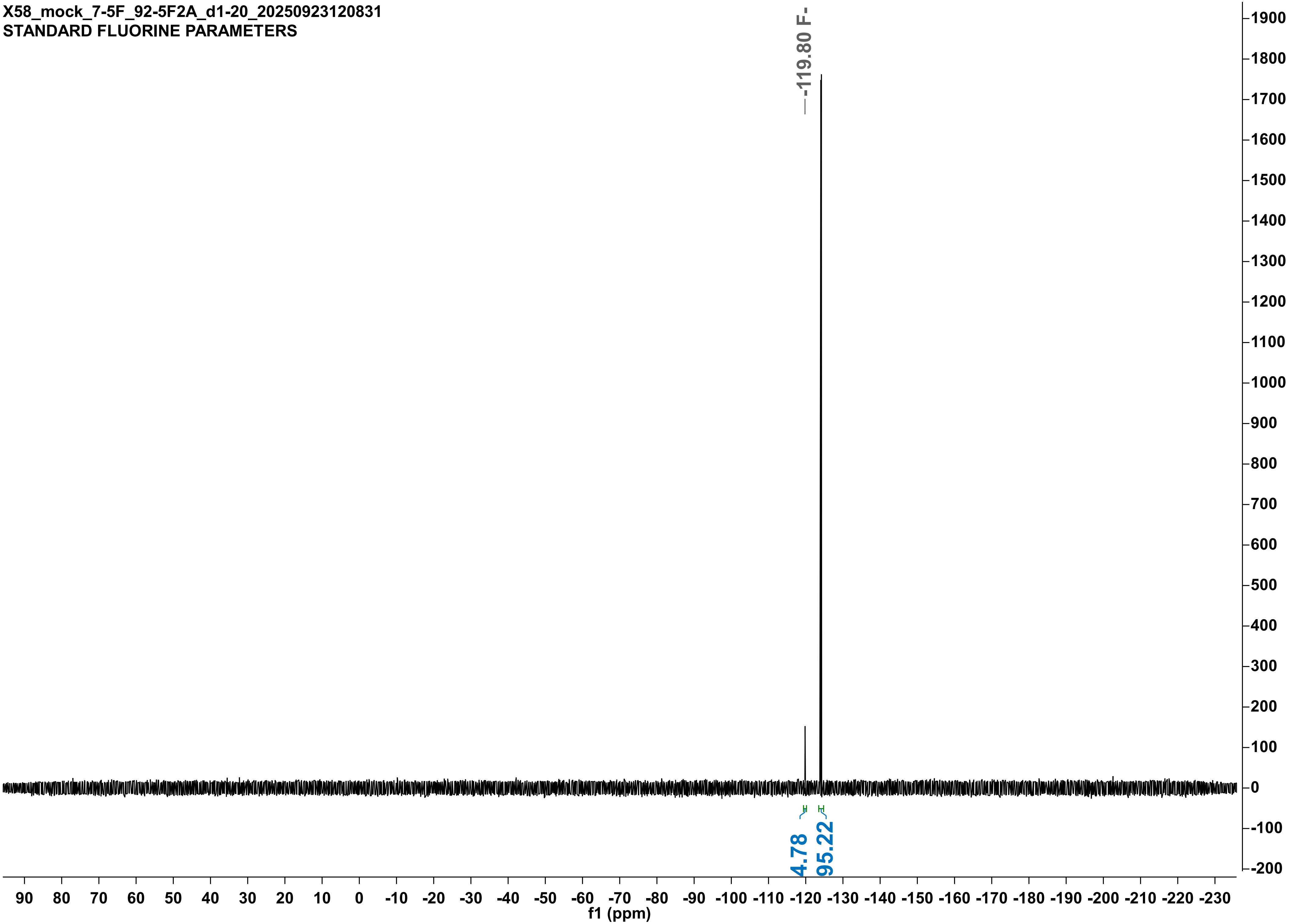

X58_mock_7-5F_92-5F2A_d1-20_nt64_20250923120849
STANDARD FLUORINE PARAMETERS

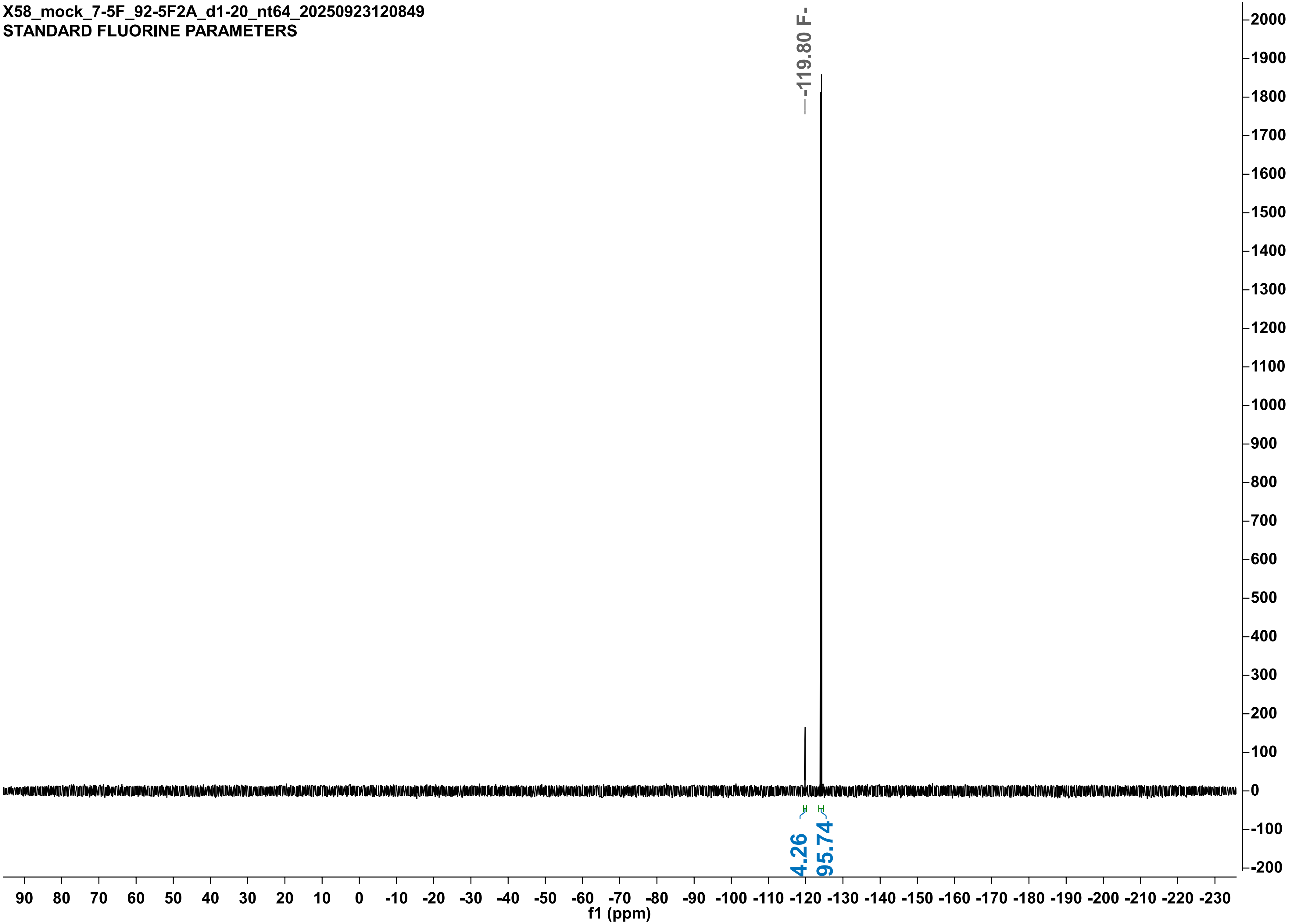

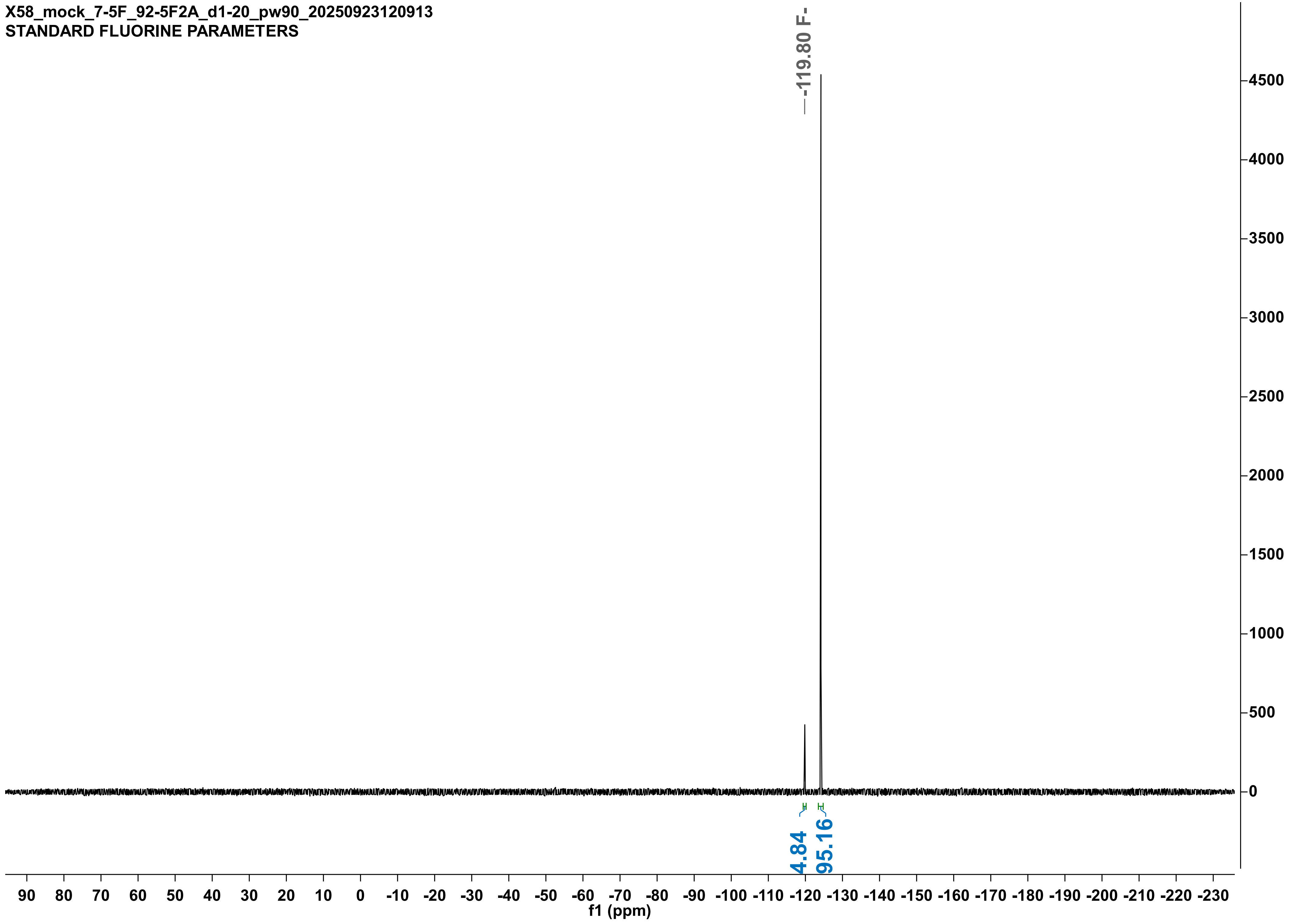

X58_mock_7-5F_92-5F2A_d1-20_pw90_20250923120913
STANDARD FLUORINE PARAMETERS
-119.80 F-
4.84
95.16
f1 (ppm)

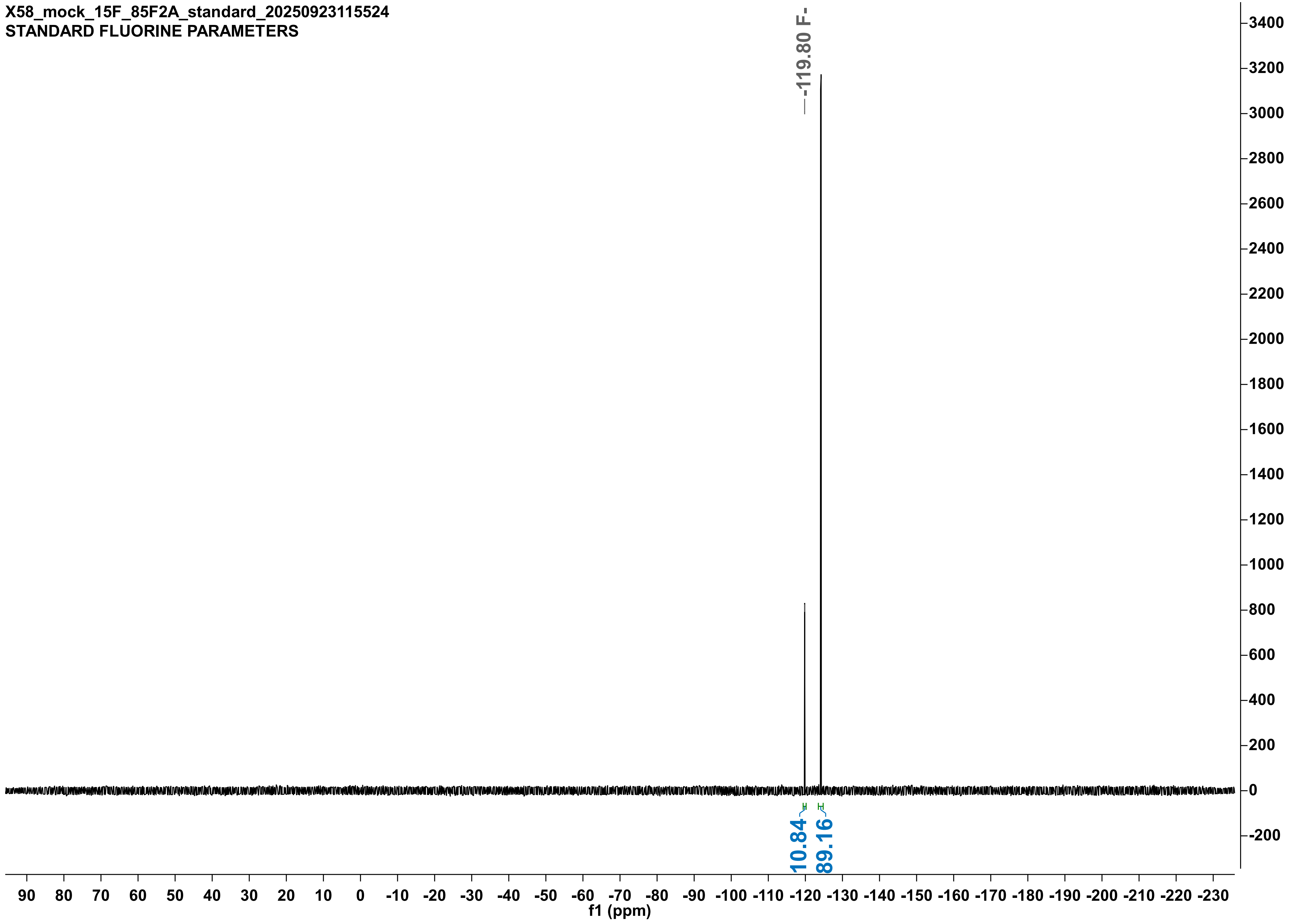

X58_mock_15F_85F2A_standard_20250923115524
STANDARD FLUORINE PARAMETERS
-119.80 F-
10.84
89.16
f1 (ppm)

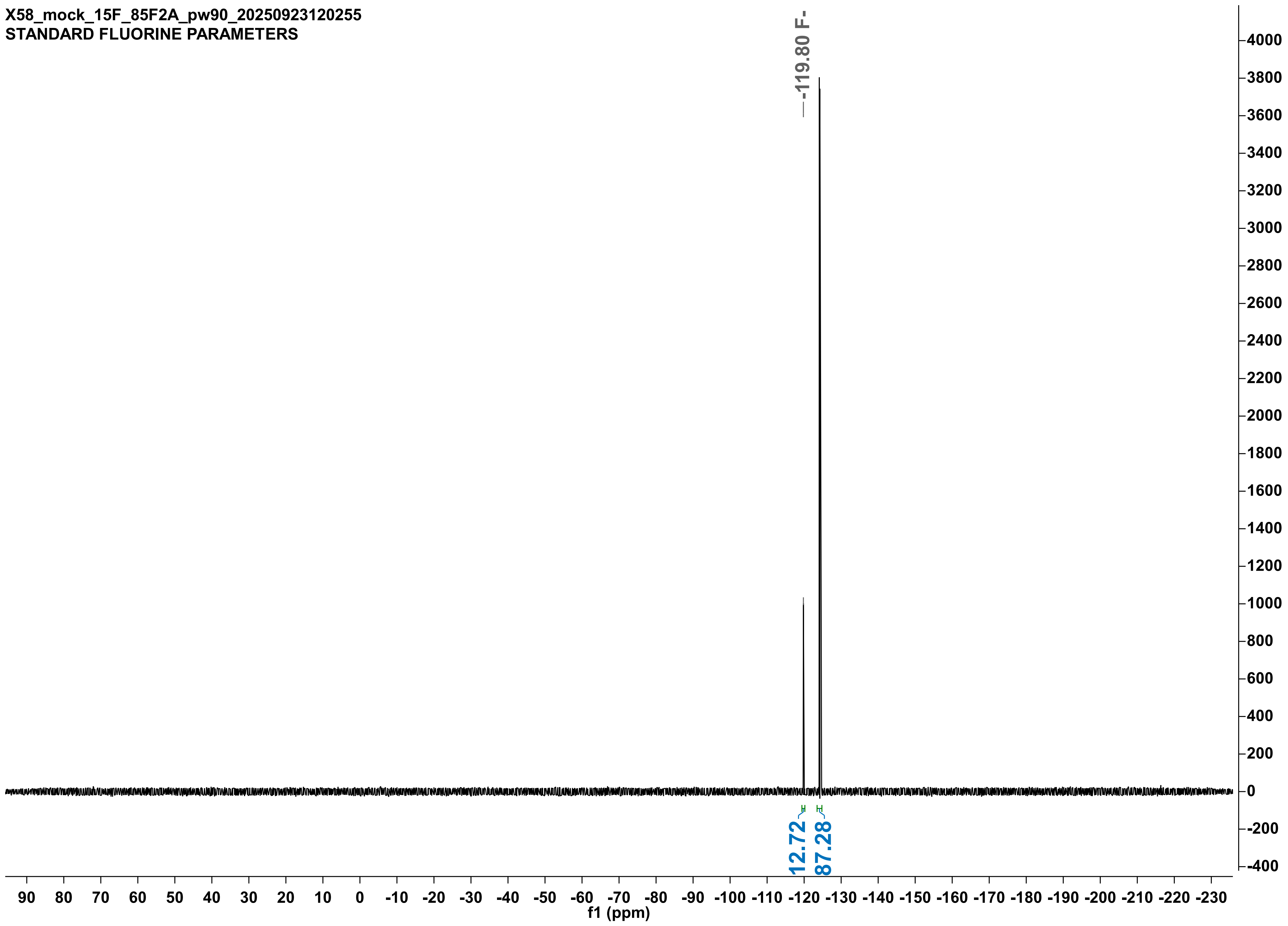

X58_mock_15F_85F2A_pw90_20250923120255
STANDARD FLUORINE PARAMETERS
-119.80 F-
12.72
87.28
f1 (ppm)

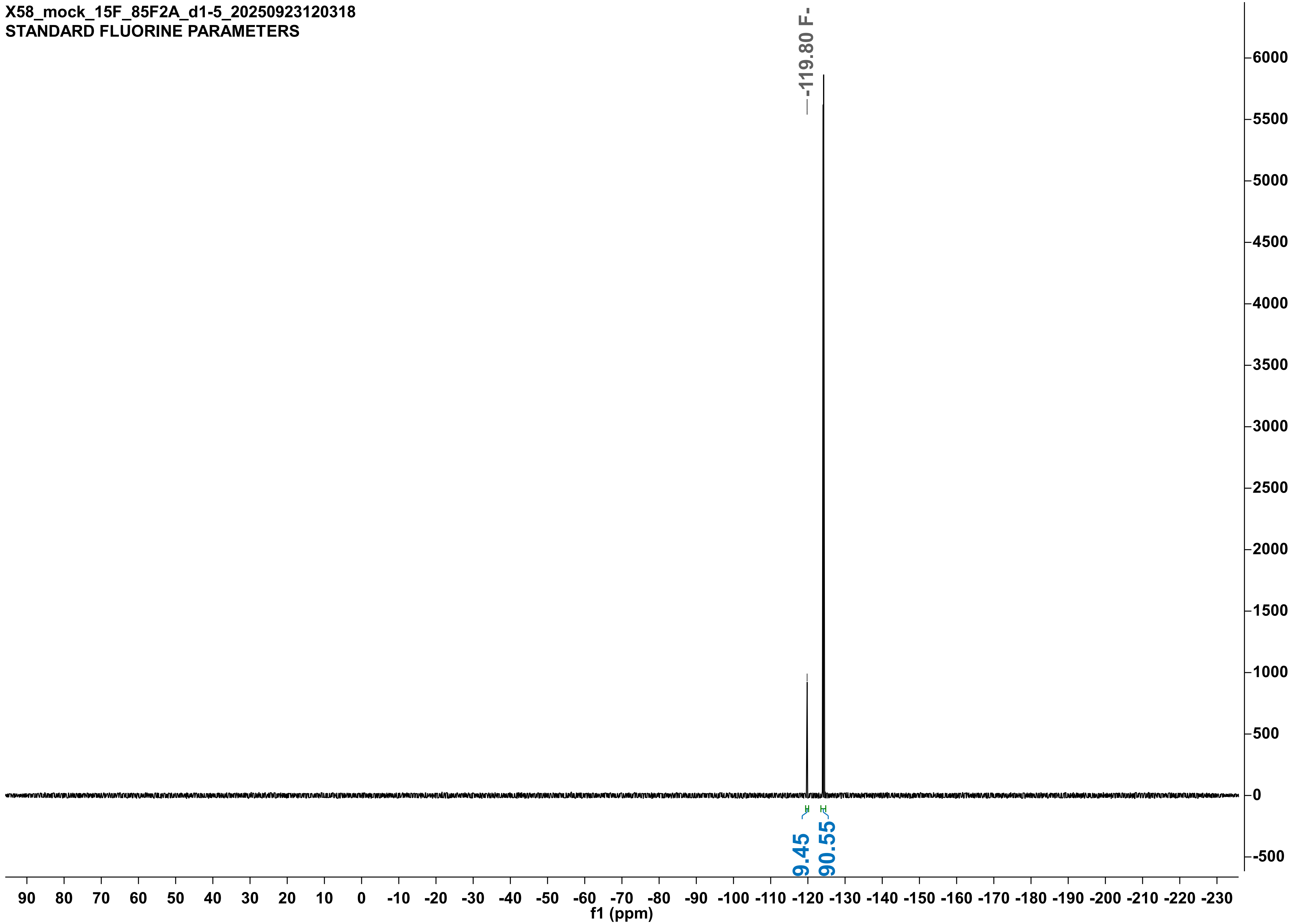
X58_mock_15F_85F2A_d1-5_20250923120318
STANDARD FLUORINE PARAMETERS
-119.80 F-
9.45
90.55
f1 (ppm)

X58_mock_15F_85F2A_d1-10_20250923120357
STANDARD FLUORINE PARAMETERS

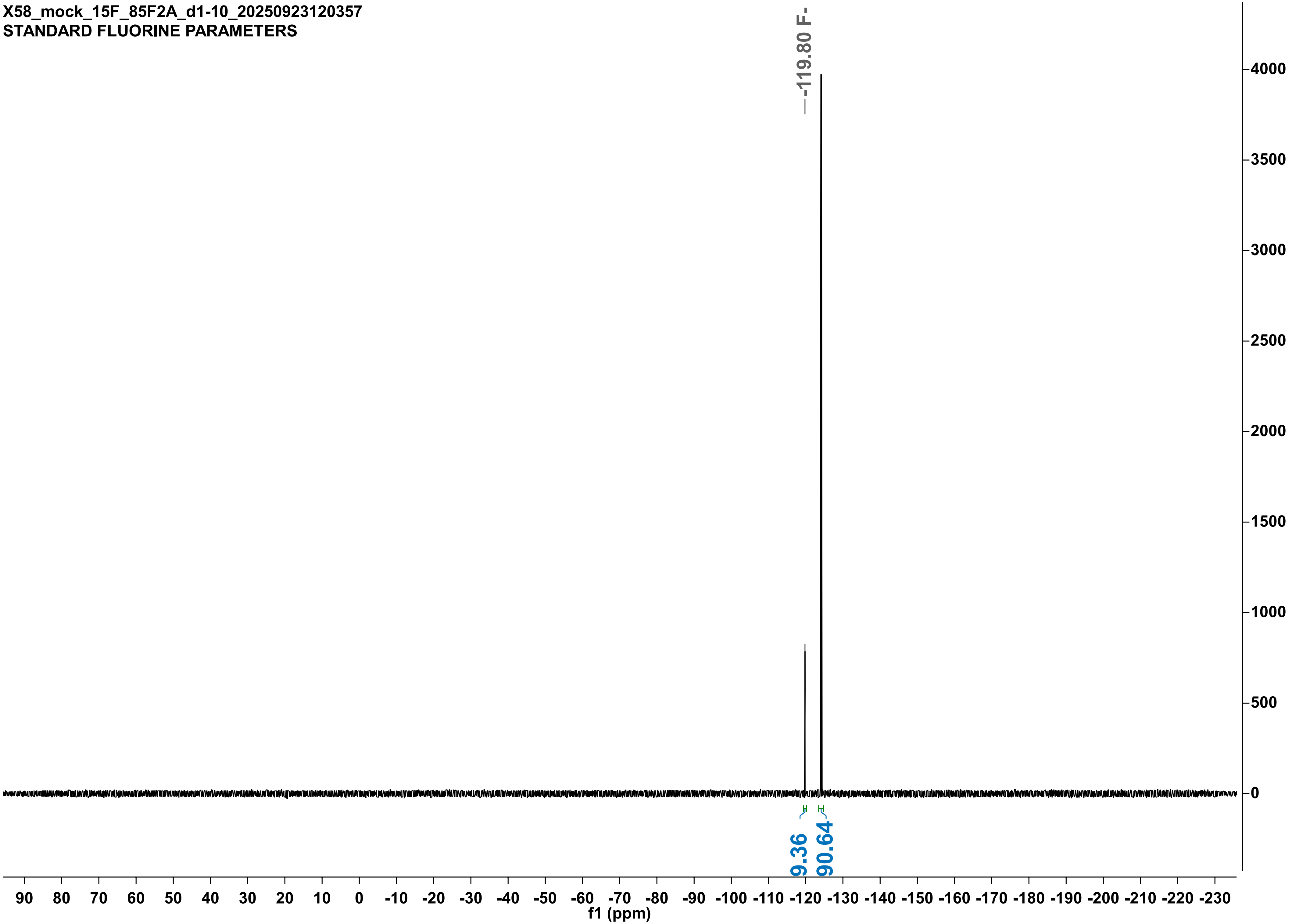

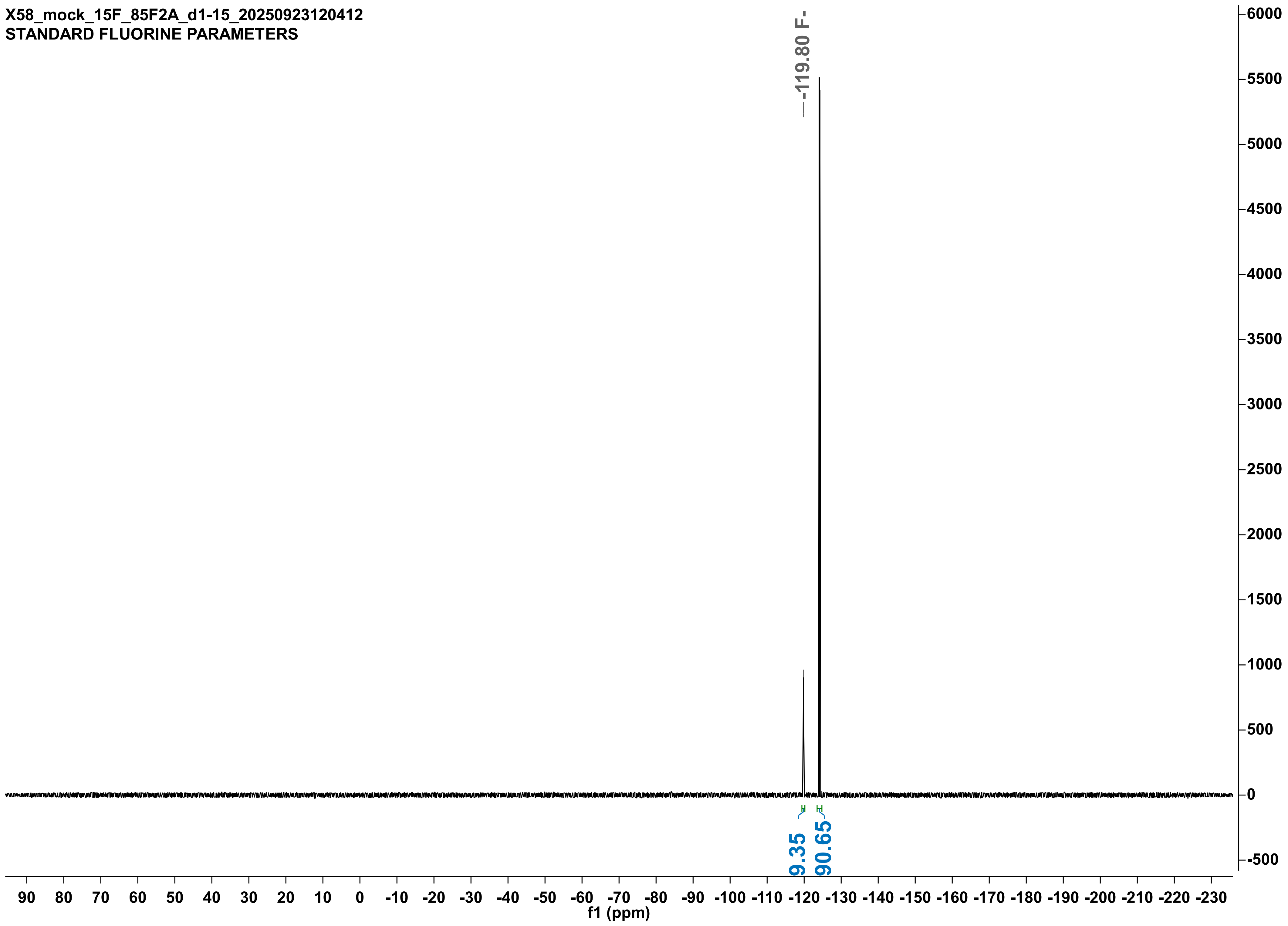
X58_mock_15F_85F2A_d1-15_20250923120412
STANDARD FLUORINE PARAMETERS
-119.80 F-
9.35
90.65
f1 (ppm)

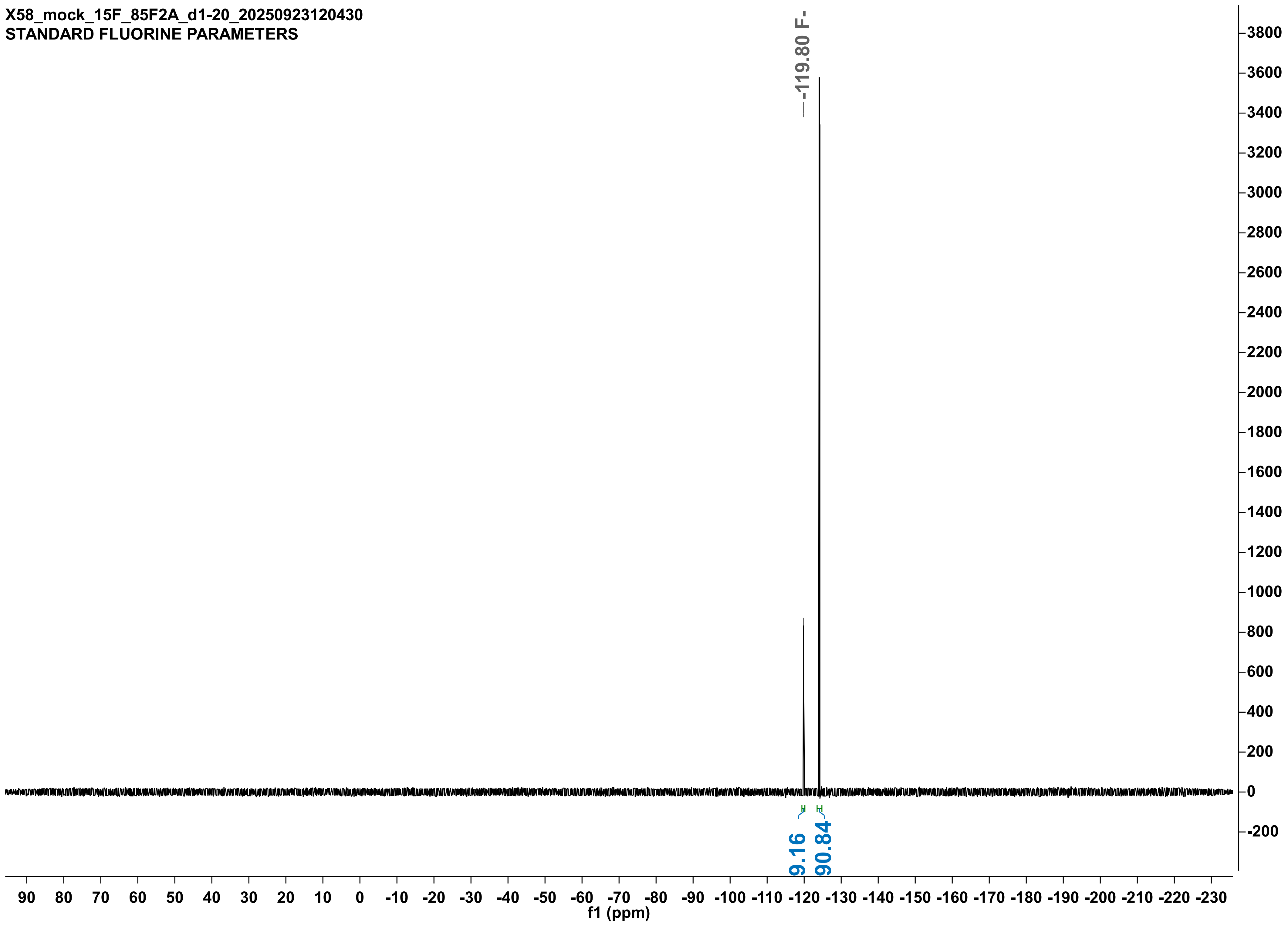
X58_mock_15F_85F2A_d1-20_20250923120430
STANDARD FLUORINE PARAMETERS
-119.80 F-
9.16
90.84
f1 (ppm)

X58_mock_15F_85F2A_d1-20_nt64_20250923120509
STANDARD FLUORINE PARAMETERS

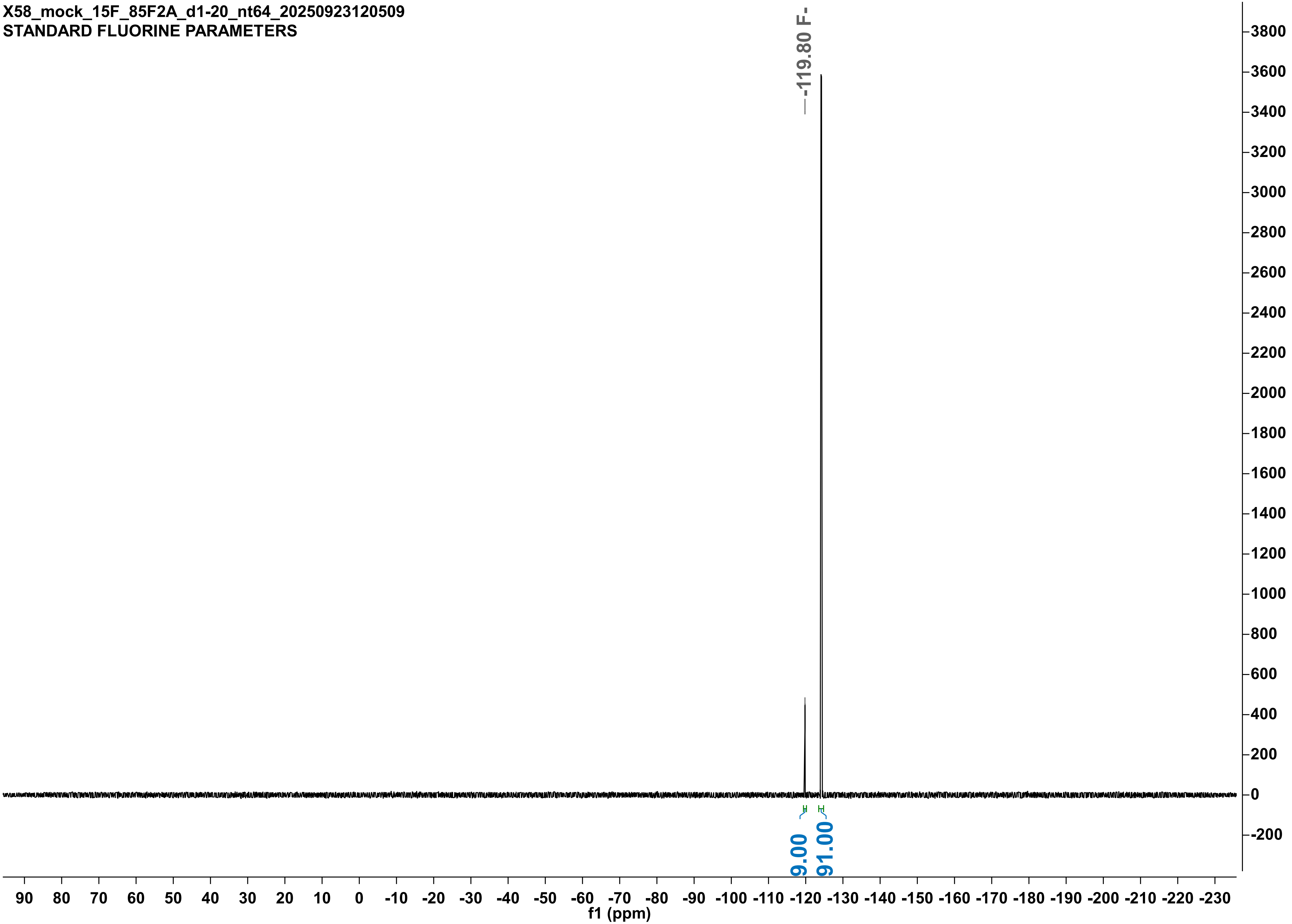

X58_mock_15F_85F2A_d1-20_pw90_20250923120538
STANDARD FLUORINE PARAMETERS

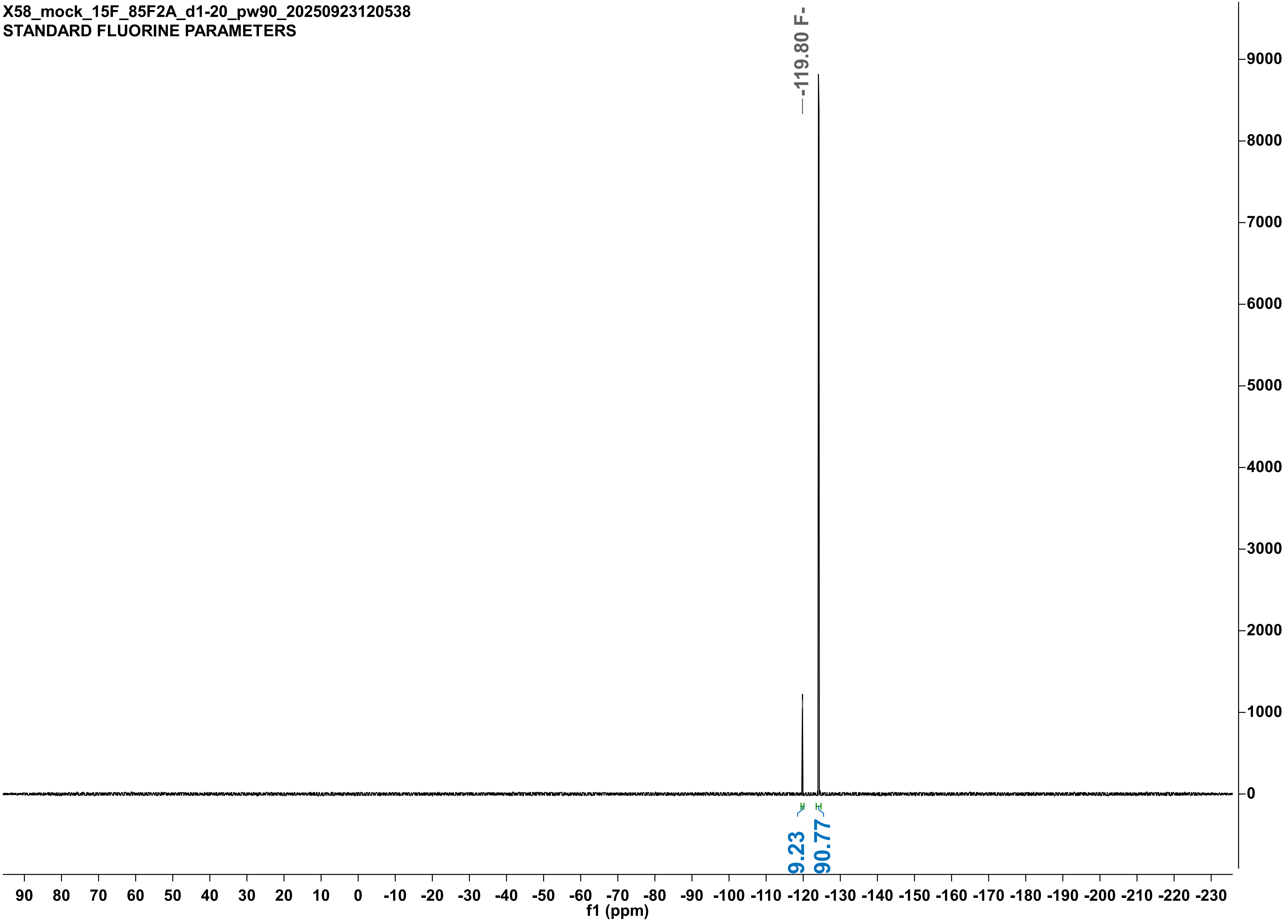

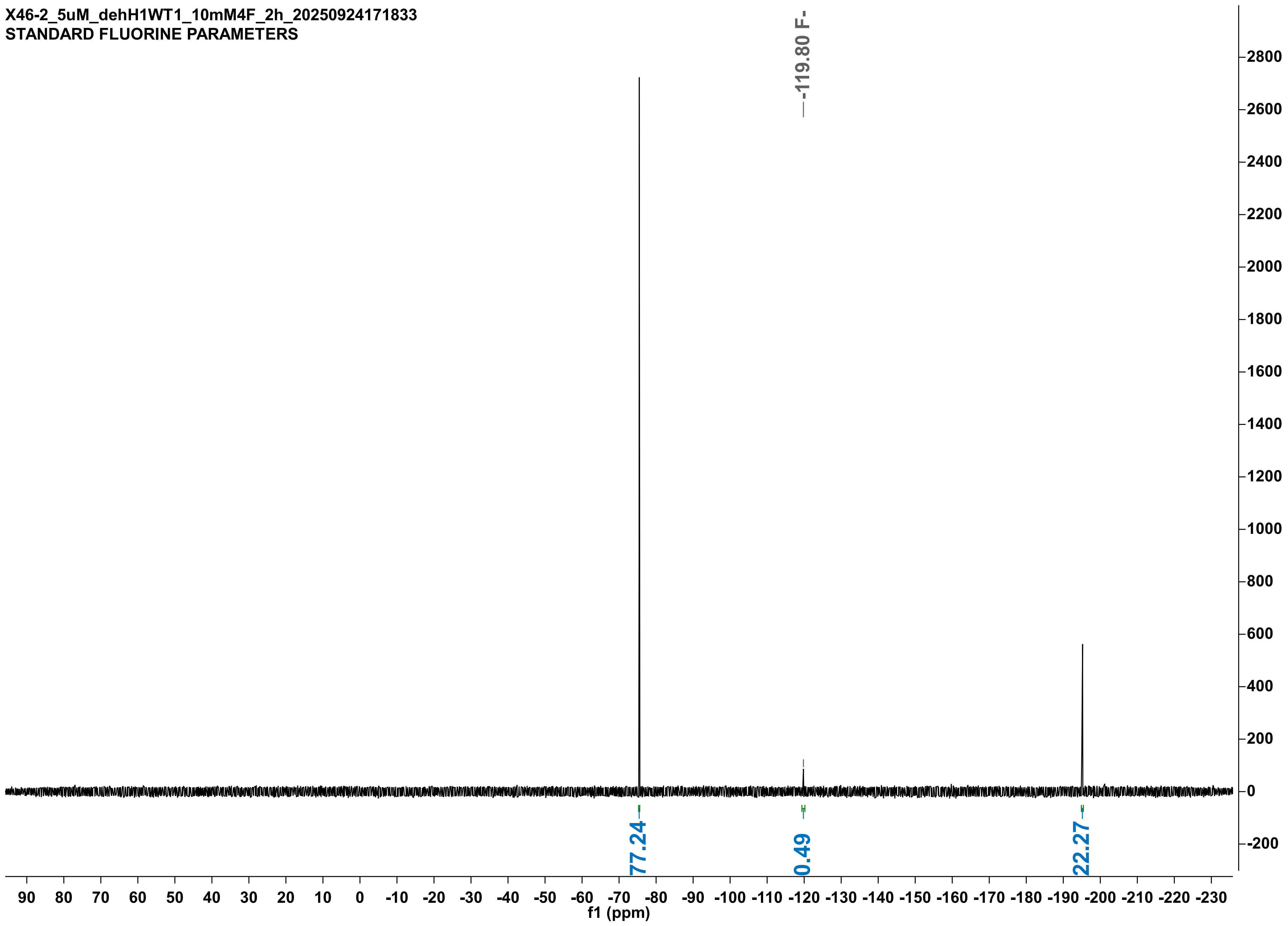

X46-2_5uM_dehH1WT1_10mM4F_2h_20250924171833
STANDARD FLUORINE PARAMETERS
-119.80 F-
77.24
0.49
22.27
f1 (ppm)

X46-2_5uM_dehH1WT2_10mM4F_2h_20250924171948
STANDARD FLUORINE PARAMETERS

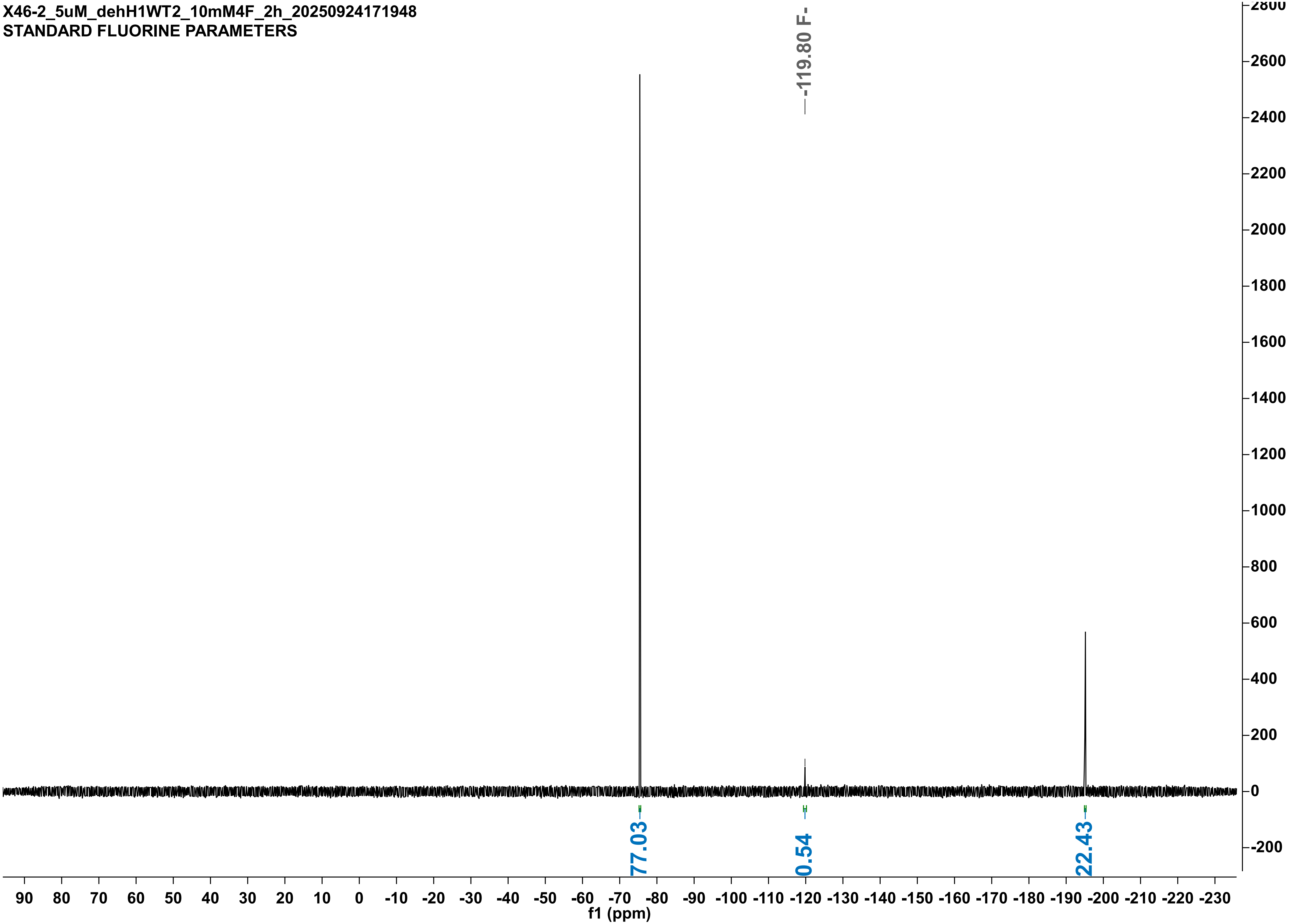

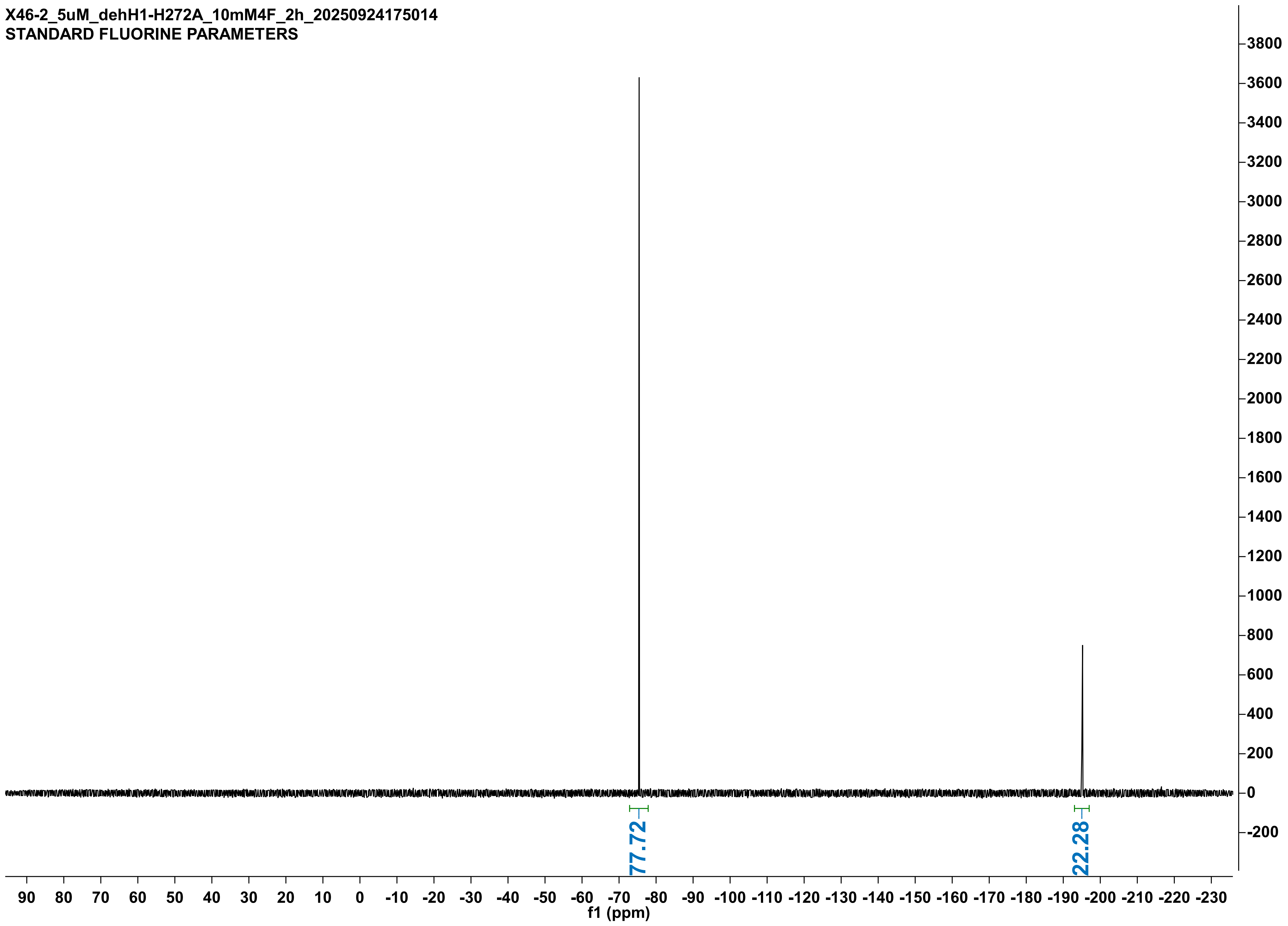

X46-2_5uM_dehH1-H272A_10mM4F_2h_20250924175014
STANDARD FLUORINE PARAMETERS
77.72
22.28
f1 (ppm)

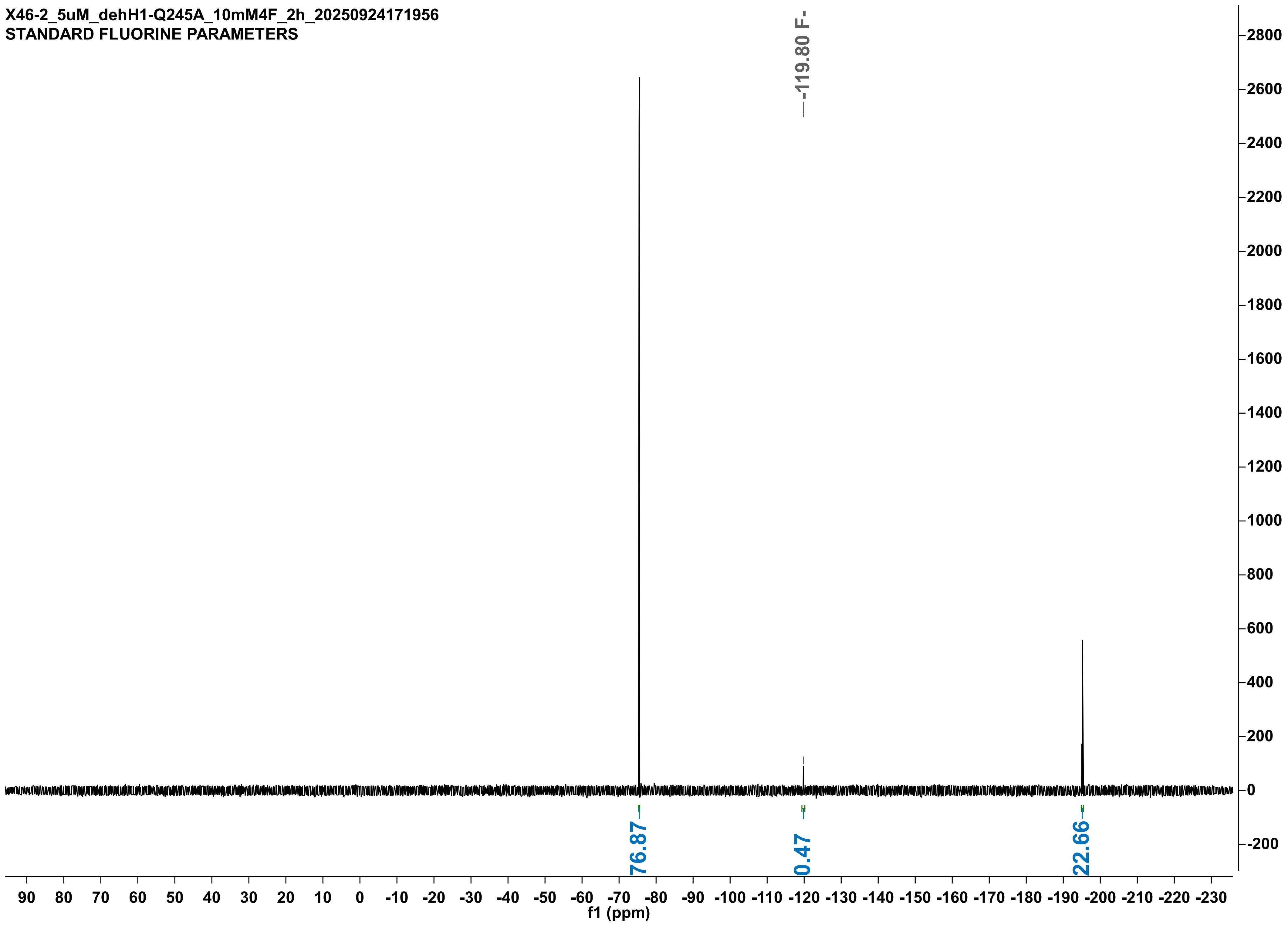
X46-2_5uM_dehH1-Q245A_10mM4F_2h_20250924171956
STANDARD FLUORINE PARAMETERS
-119.80 F-
76.87
0.47
22.66
f1 (ppm)

X46-2_5uM_dehH1-Q245A-DUP_10mM4F_2h_20250924172008
STANDARD FLUORINE PARAMETERS

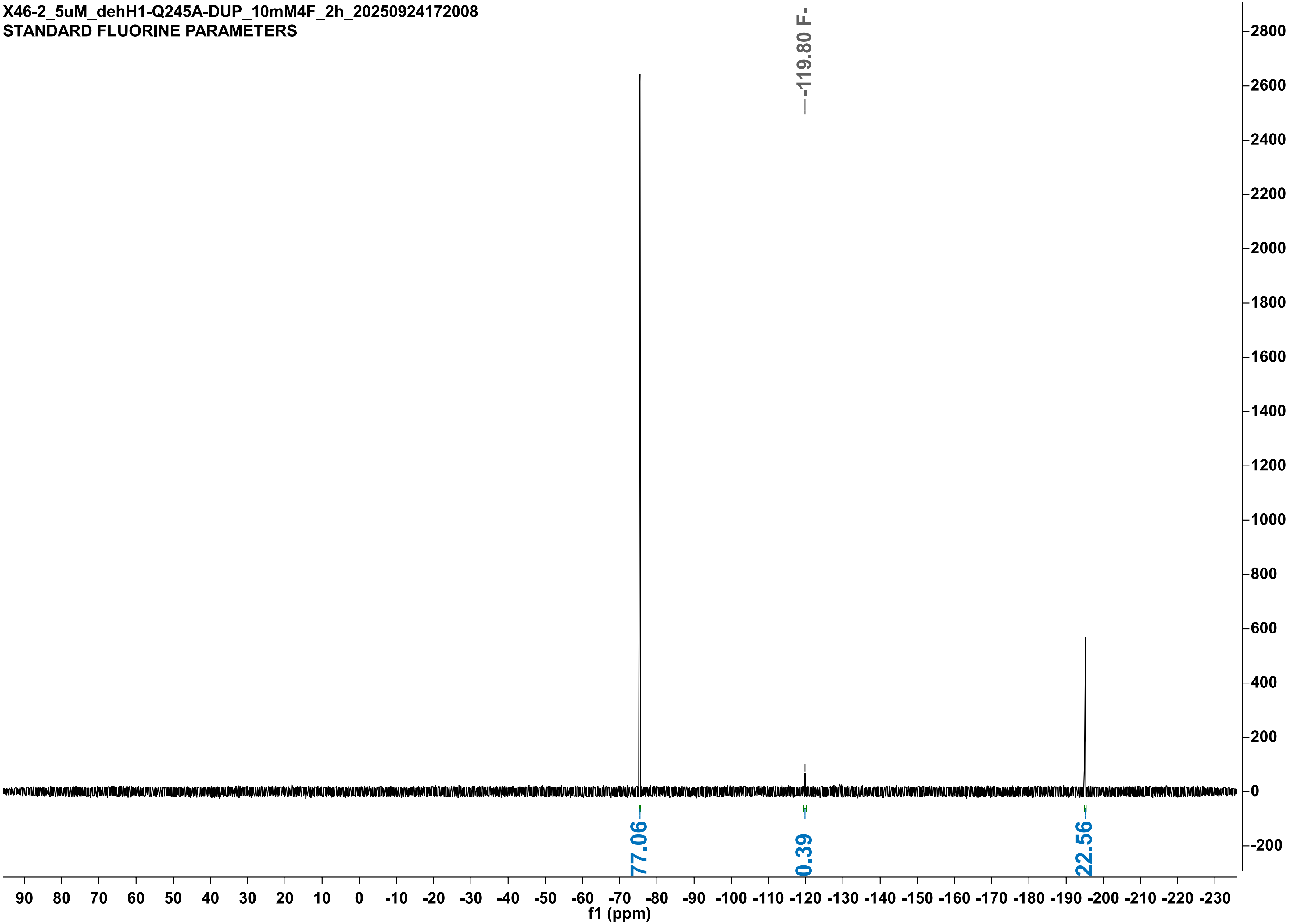

X46-2_5uM_dehH1-ML_10mM4F_2h_20250924173411
STANDARD FLUORINE PARAMETERS

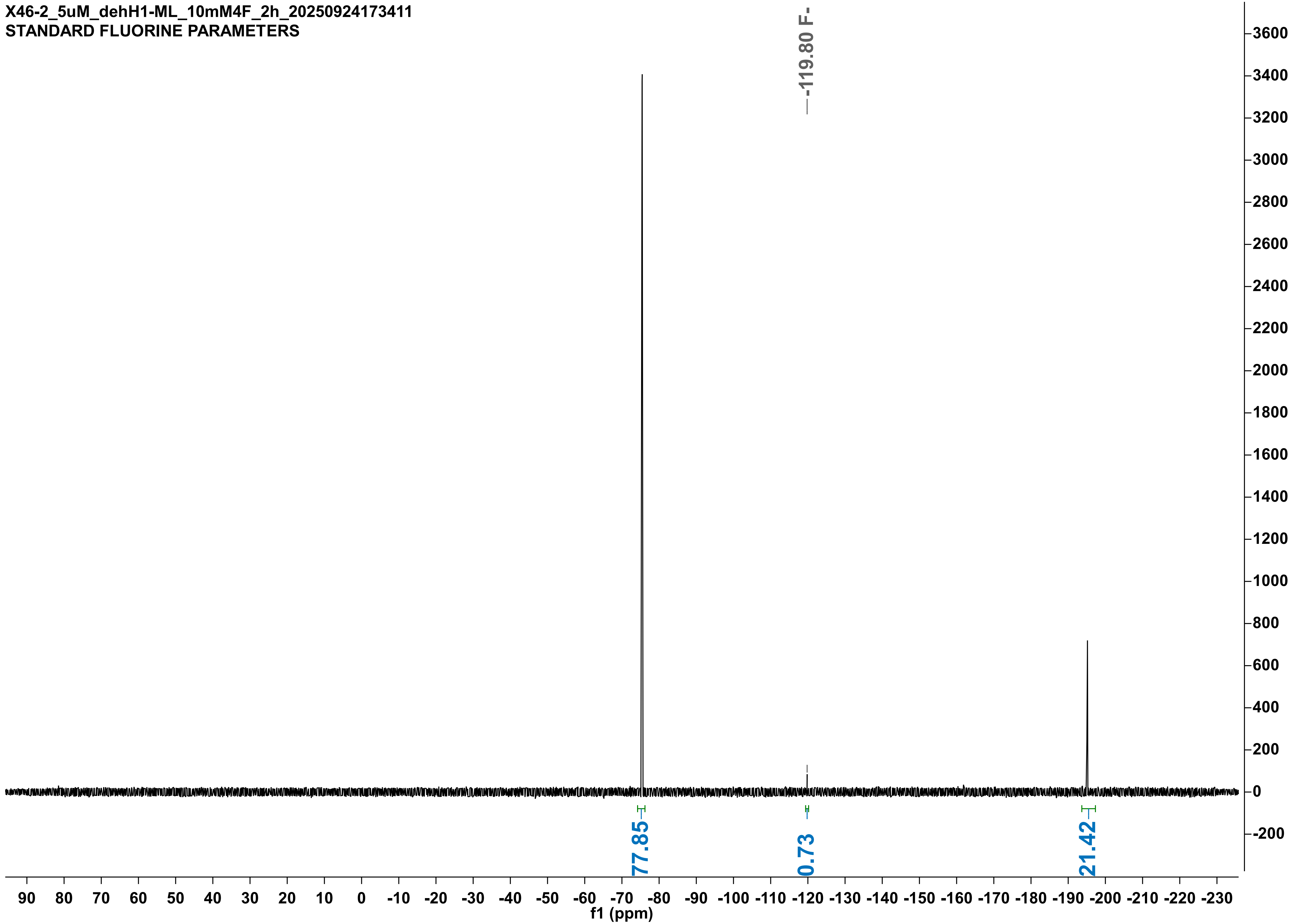

X46-2_5uM_dehH1-ML-DUP_10mM4F_2h_20250924174059
STANDARD FLUORINE PARAMETERS

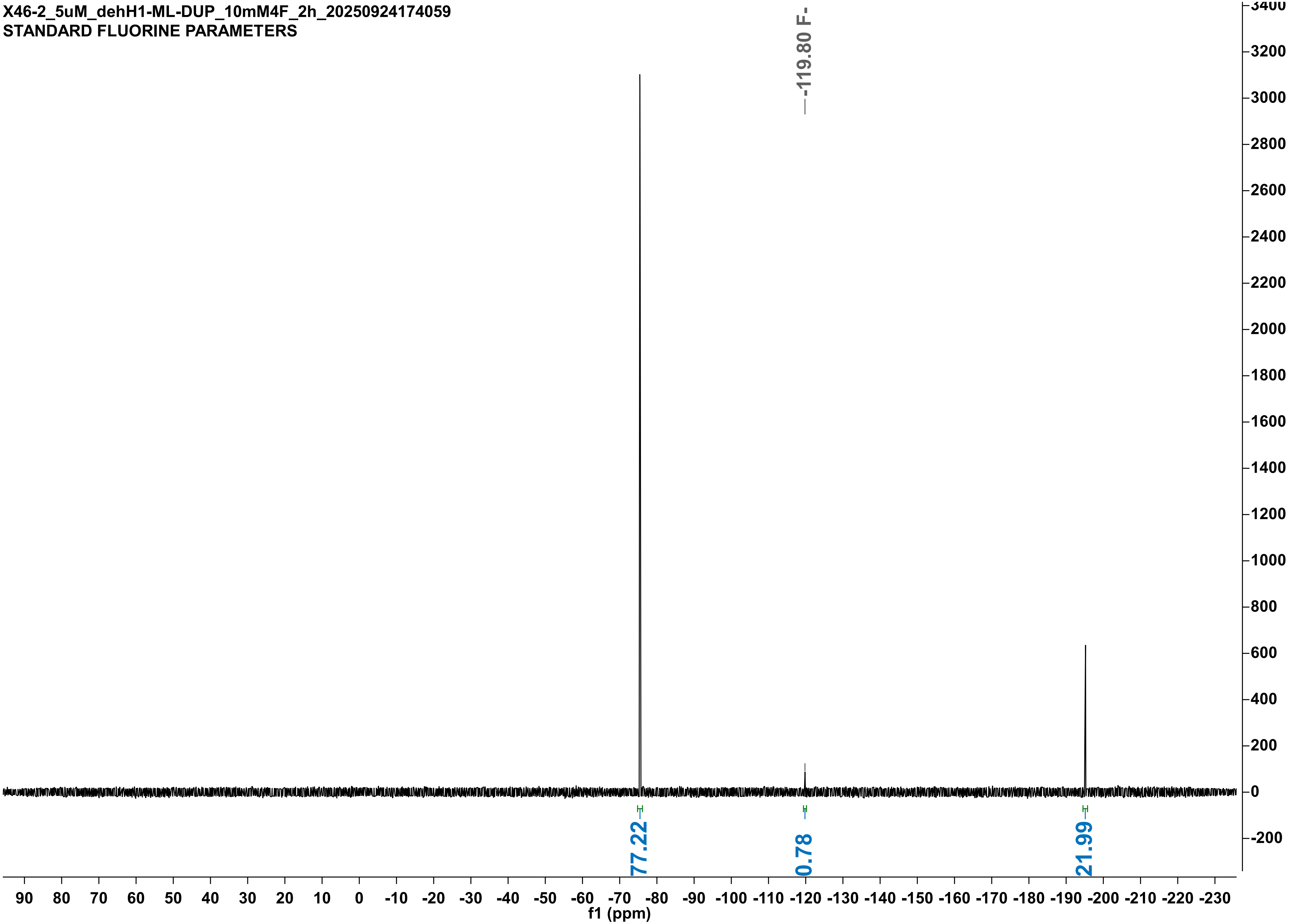

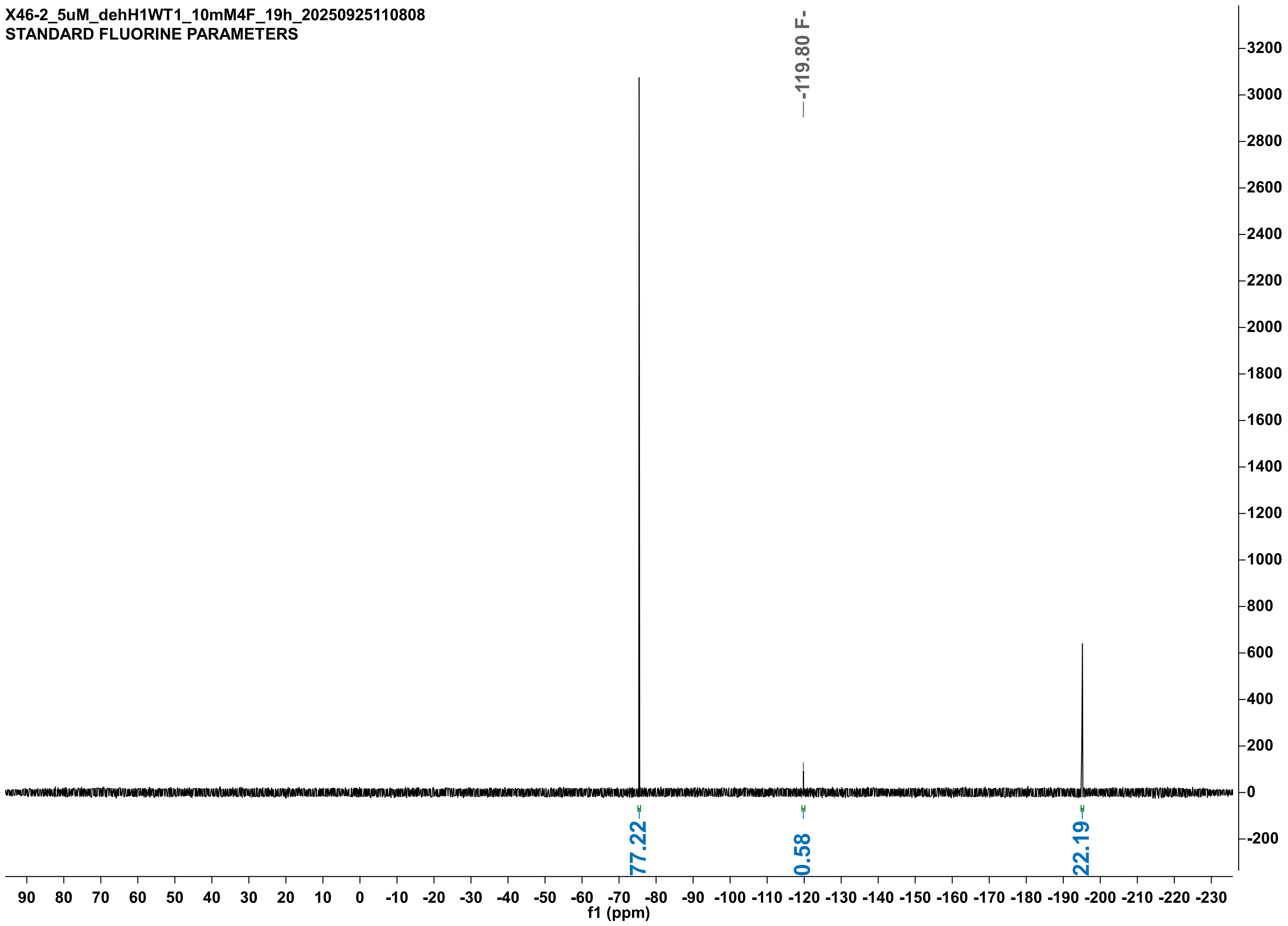
X46-2_5uM_dehH1WT1_10mM4F_19h_20250925110808
STANDARD FLUORINE PARAMETERS
-119.80 F-
77.22
0.58
22.19
f1 (ppm)

X46-2_5uM_dehH1WT2_10mM4F_19h_20250925110820
STANDARD FLUORINE PARAMETERS

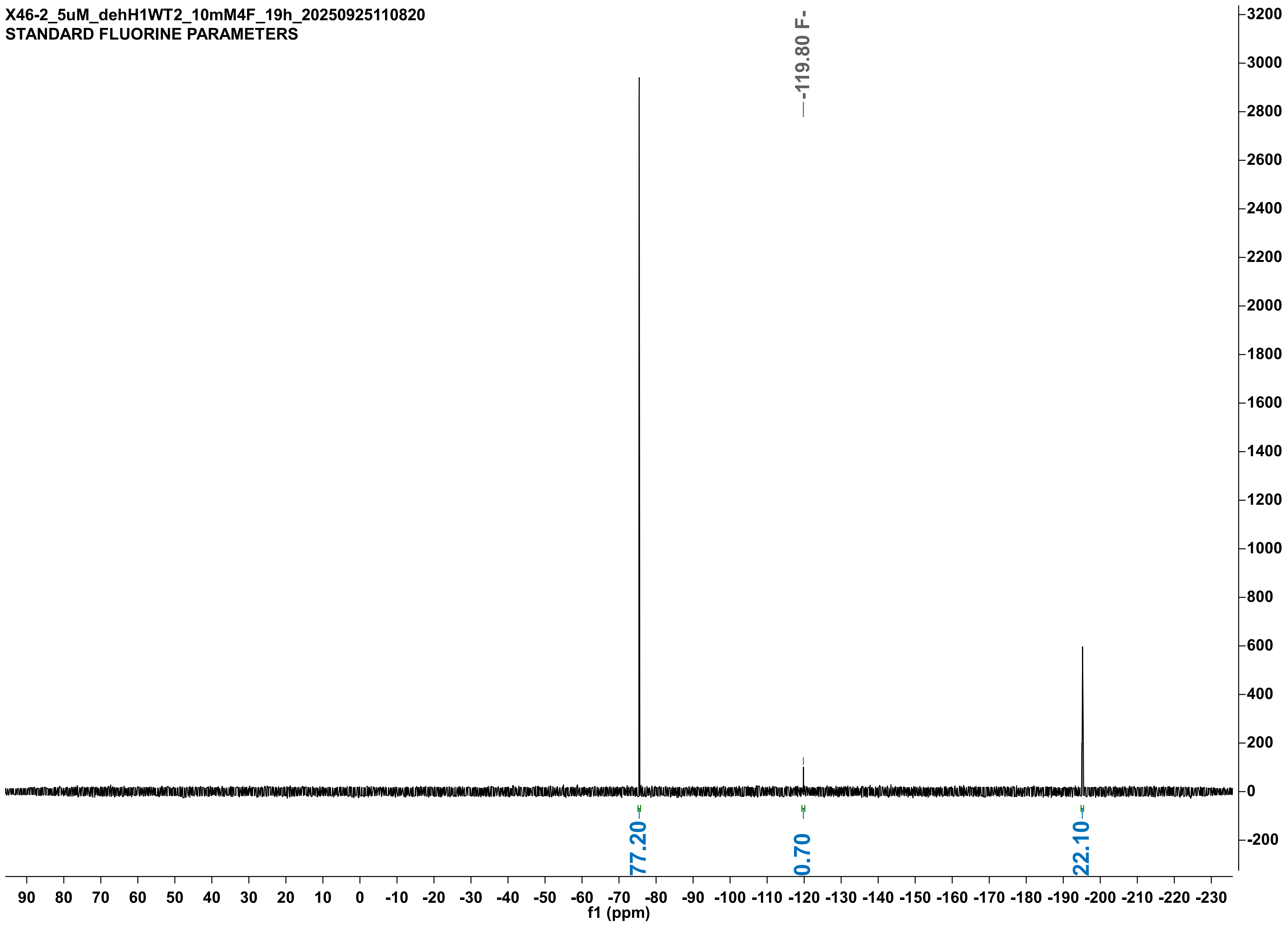

X46-2_5uM_dehH1-H272A_10mM4F_19h_20250925112105
STANDARD FLUORINE PARAMETERS

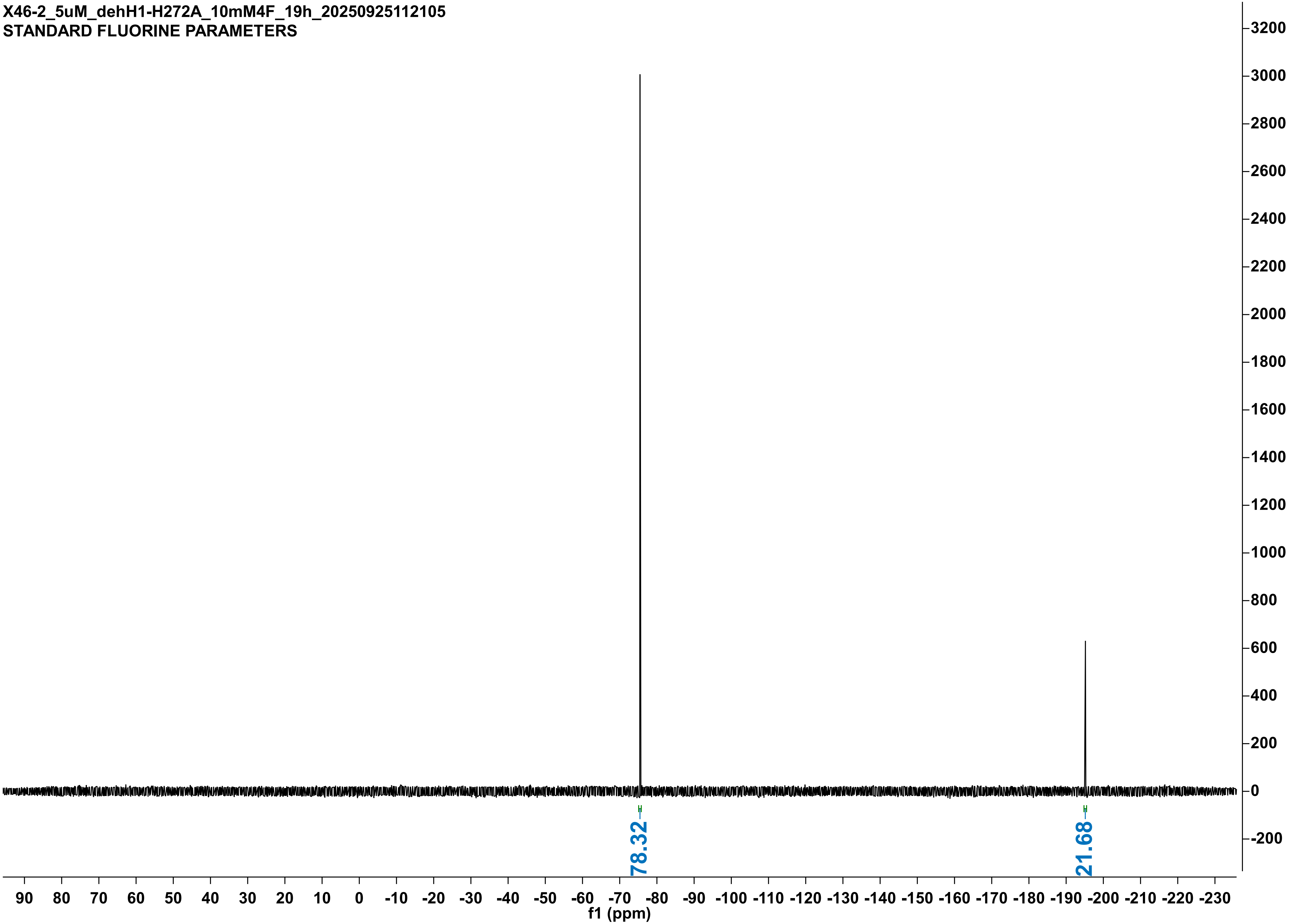

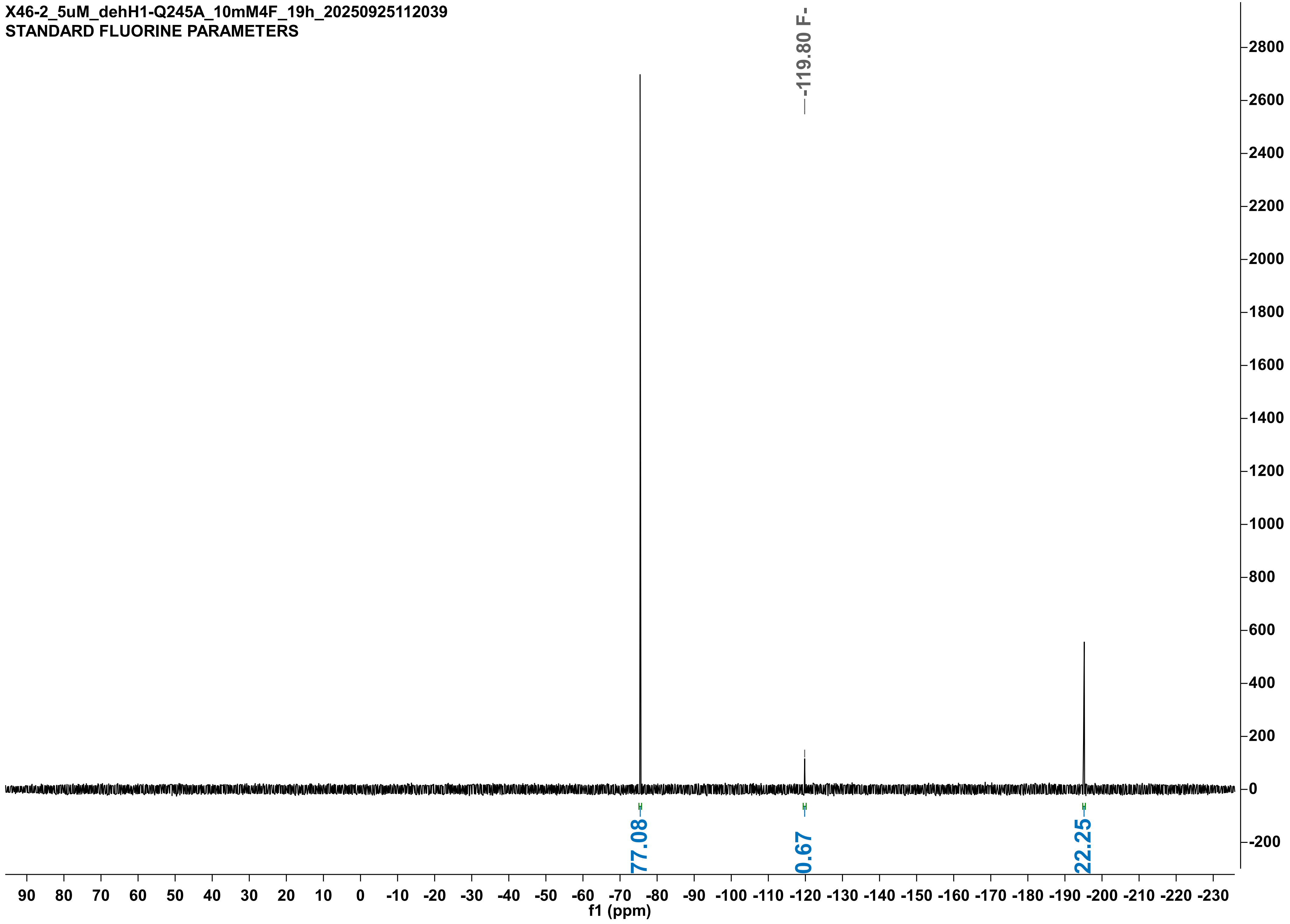

X46-2_5uM_dehH1-Q245A_10mM4F_19h_20250925112039
STANDARD FLUORINE PARAMETERS
-119.80 F-
77.08
0.67
22.25
f1 (ppm)

X46-2_5uM_dehH1-Q245A-DUP_10mM4F_19h_20250925112051
STANDARD FLUORINE PARAMETERS

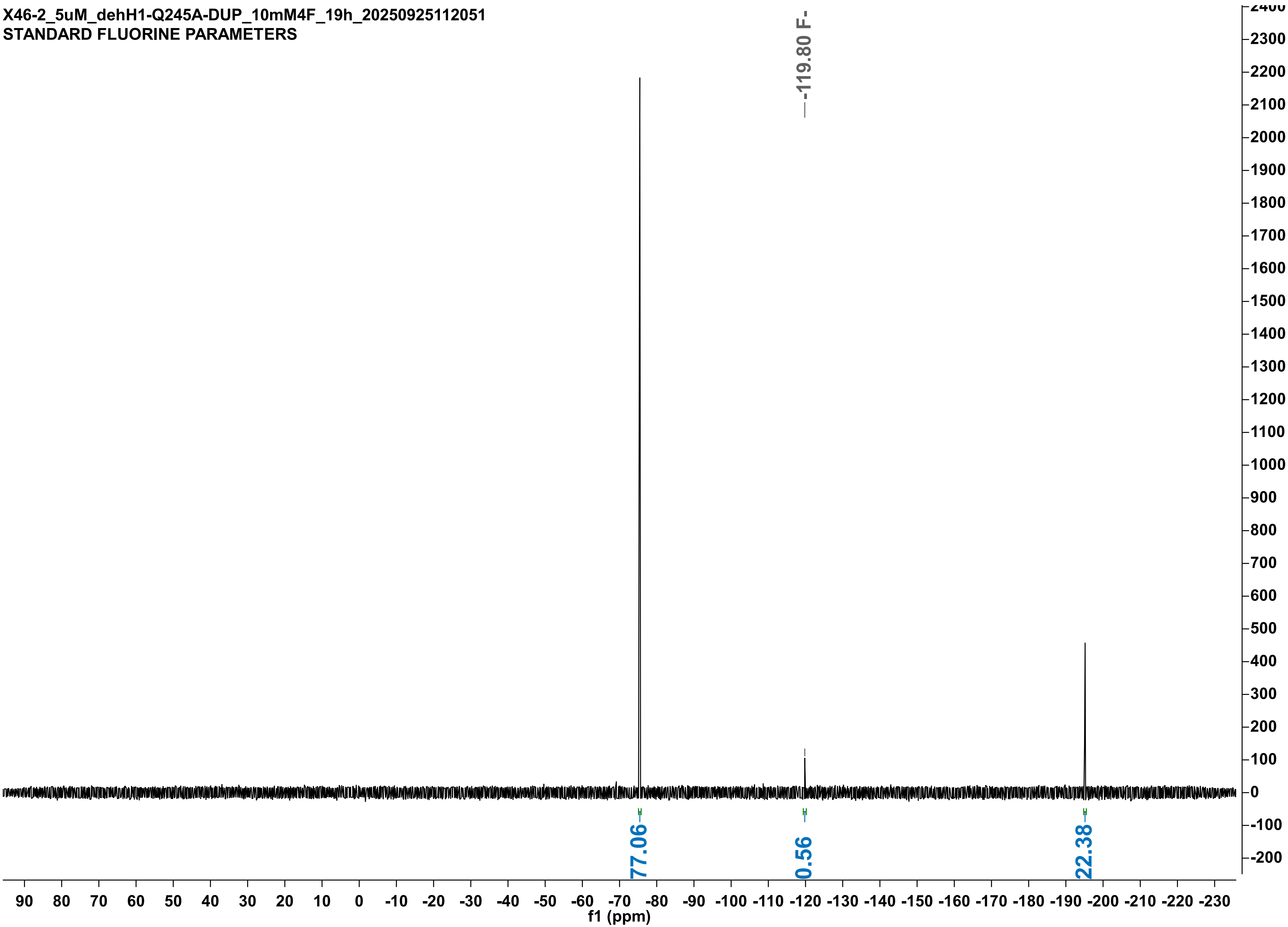

X46-2_5uM_dehH1-ML_10mM4F_19h_20250925110740
STANDARD FLUORINE PARAMETERS

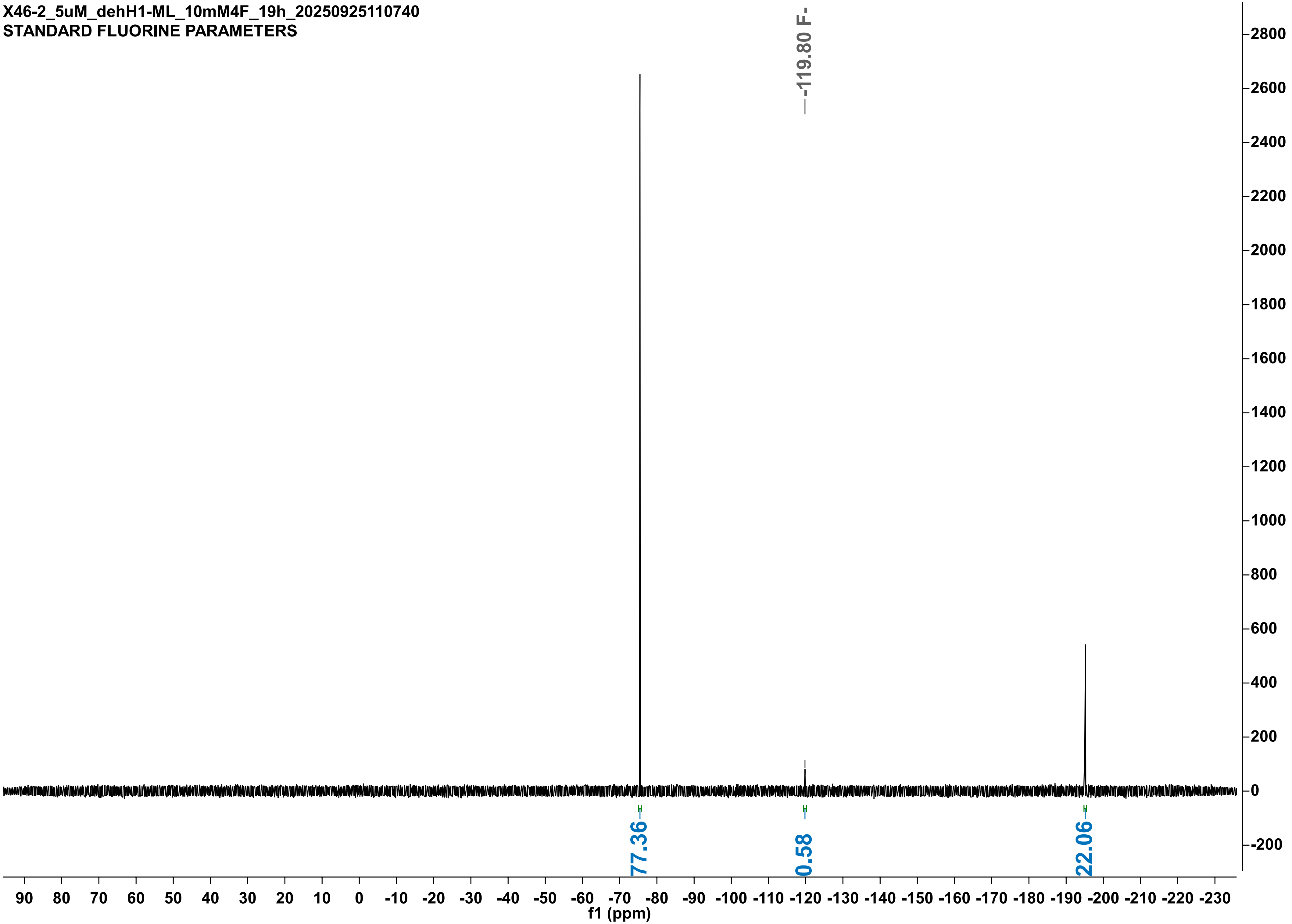

X46-2_5uM_dehH1-ML-DUP_10mM4F_19h_20250925110757
STANDARD FLUORINE PARAMETERS

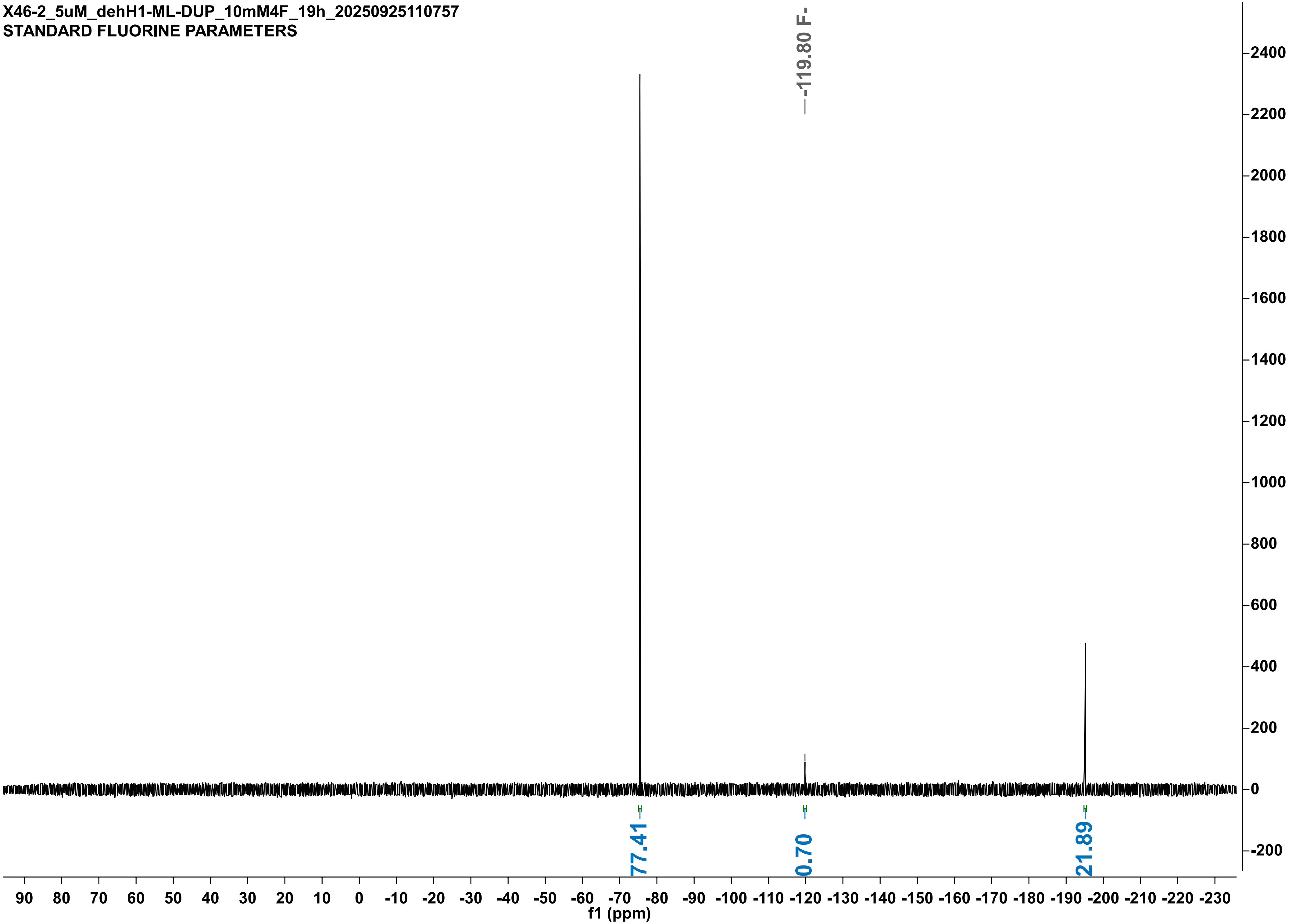

Supplement: Supplementary file 3 — Supporting File 3: anie71294‐sup‐0003‐19FNMR_supernatants, kinetics, eetest, d1, 4F.pdf. [file ANIE-65-e24234-s002.pdf]
